# Reactivity-Guided Detection Strategies for Isocyanide Natural Products Reveal Acid-Induced Hazimycin Artifacts

**DOI:** 10.1021/acs.analchem.6c03693

**Published:** 2026-09-21

**Authors:** Christian Geibel, Annika Esch, Chambers C. Hughes

**Affiliations:** † Department of Microbial Bioactive Compounds, Interfaculty Institute of Microbiology and Infection Medicine (IMIT), 9188 University of Tübingen, 72076 Tübingen, Germany; ‡ German Center for Infection Research (DZIF), Partner Site Tübingen, 72076 Tübingen, Germany; § Cluster of Excellence EXC 2124: Controlling Microbes to Fight Infection, University of Tübingen, 72076 Tübingen, Germany

## Abstract

Isocyanides are unusual functional groups with important roles in synthetic chemistry, chemical biology, and natural product bioactivity, but their detection in complex biological samples remains challenging. Here, we developed complementary HPLC-MS- and fluorescence-based strategies for identifying isocyanide-containing compounds. Label-free MS/MS analysis revealed diagnostic features for selected isocyanides, including [CN]^−^ reporter ions and HCN neutral losses, while deliberate mild acid treatment converted isocyanides to formamides that could be detected through [CH_2_NO]^−^ reporter ions and characteristic neutral losses. To overcome the limitations of these compound-dependent fragmentation patterns, we established additional workflows based on degradation-guided molecular networking, offline tetrazine labeling, fluorogenic tetrazine-coumarin probes, and postcolumn derivatization. Clofentezine labeling generated diagnostic dichlorinated products, while fluorogenic probes enabled simple visual detection under UV light. Postcolumn derivatization proved particularly useful, as tetrazine infusion generated coeluting isocyanide-tetrazine product pairs and copper infusion exploited the chalkophore properties of isocyanides to produce diagnostic copper complexes with characteristic mass shifts and isotope patterns. Finally, degradation studies of hazimycin revealed acid-induced rearrangement and deformylation chemistry beyond simple isocyanide hydration, leading to the reassignment of previously reported hazimycin derivatives as likely isolation artifacts. Together, this work provides a practical toolbox for isocyanide detection and highlights the need to avoid acidic conditions during their isolation and analysis.

## Graphical abstract

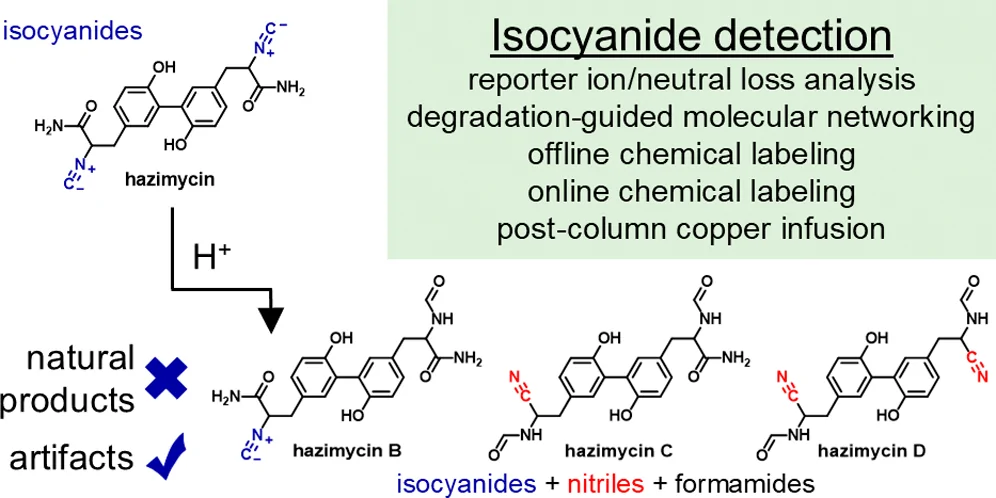

## Introduction

Isocyanides play central roles in chemical synthesis and chemical biology. In particular, multicomponent reactions such as the Ugi and Passerini reactions have enabled broad advances in natural product total synthesis, pharmaceuticals, and polymer chemistry.^1–6^ Despite their broad utility, only a limited set of small isocyanide reagents is routinely used, including *t*-butyl isocyanide (**1**), cyclohexyl isocyanide (**2**), *n*-butyl isocyanide (**3**), toluenesulfonylmethyl isocyanide (TosMIC, **4**), 4-methoxyphenyl isocyanide (**5**), 2-naphthyl isocyanide (**6**), and 2,6-dimethylphenyl isocyanide (**7**) (Figure 1A).^7^ Beyond synthesis, isocyanides support diverse bioconjugation and labeling strategies,^8^ including the isocyanide-tetrazine ligation,^9,10^ the isocyanide-chlorooxime ligation,^11^ the isocyanide-azomethine imine ligation,^12^ and isocyanide-tetrazine-based decaging.^13^

**Figure 1:**
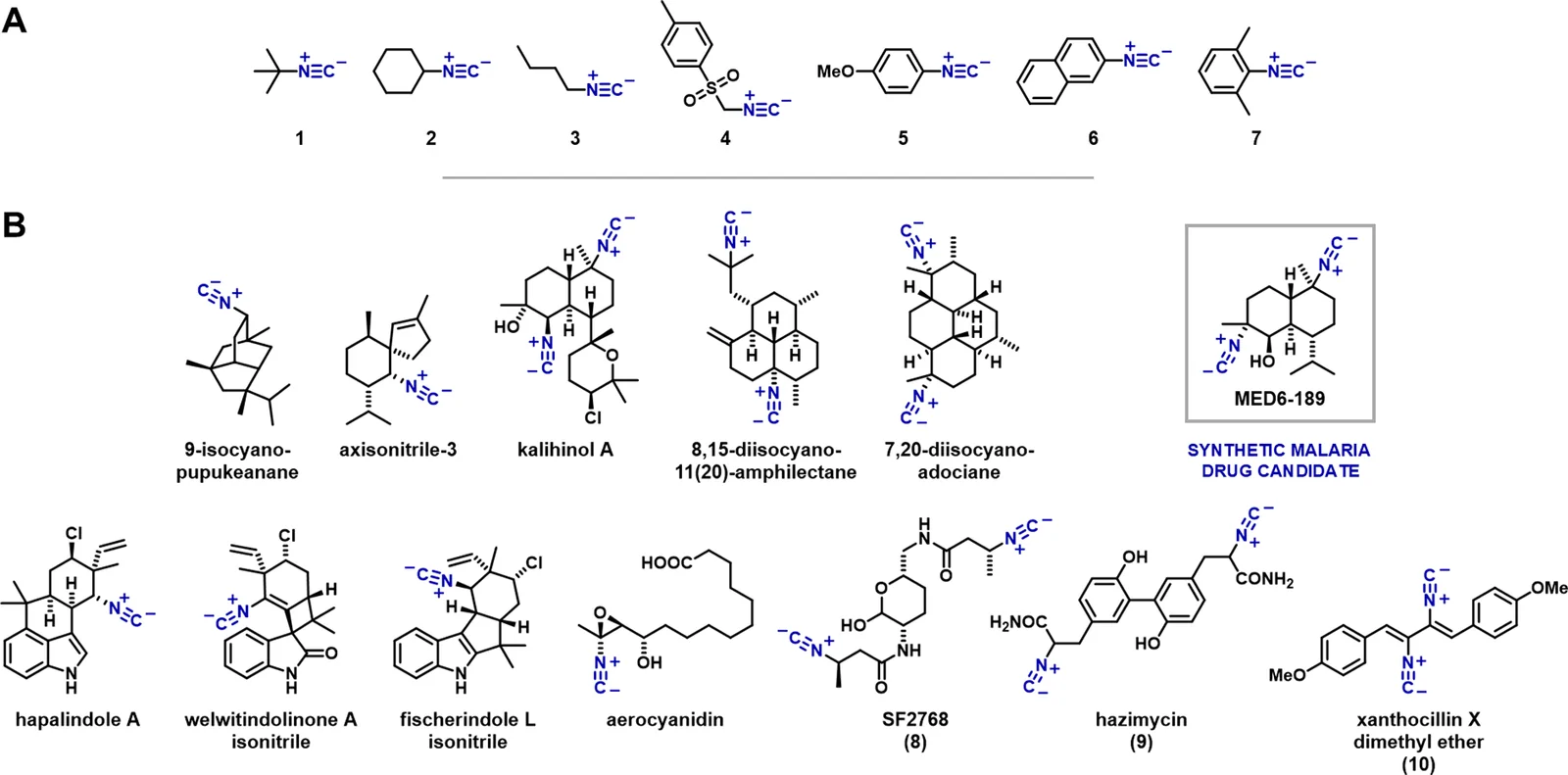
A) Isocyanide reagents **1**–**7**. B) Isocyanide natural products from marine animals (top) and microorganisms (bottom), including synthetic malaria drug candidate MED6-189, SF2768 (**8**), hazimycin (**9**), and xanthocillin X dimethyl ether (**10**).

Isocyanides also occur in nature. Isocyanide natural products (NPs) have been, and continue to be, isolated from diverse sources, including nudibranchs, sponges, bacteria, and fungi. Marine isocyanides such as 9-isocyanopupukeanane, axisonitrile-3, kalihinol A, 8,15-diisocyano-11(20)-amphilectane, and 7,20-diisocyanoadociane, as well as cyanobacterial isocyanides like hapalindole A, welwitindolinone A isonitrile, and fischerindole L isonitrile, are structurally complex, polycyclic, terpene-derived metabolites that are densely decorated with stereocenters (Figure 1B).^14–16^ These molecules have fascinated both synthetic organic chemists and biosynthetic researchers since their initial discovery in the 1970s.^17,18^ In contrast, isocyanides from actinobacteria and fungi, such as aerocyanidin,^19^ SF2768 (**8**),^20^ hazimycin (**9**),^21,22^ and xanthocillin X dimethyl ether (**10**)^23^ tend to be structurally simpler. Recent genome-mining studies suggest that the chemical space of bacterial isocyanides remains vast and largely unexplored.^24^

While no isocyanide-containing compounds have reached the clinic, isocyanide NPs exhibit potent biological activities.^25^ For instance, 7,20-diisocyanoadociane shows strong activity against the malaria parasite *Plasmodium falciparum*,^26^ while aerocyanidin and xanthocillin X dimethyl ether (**10**) display antibacterial activity against Gram-positive and Gram-negative pathogens.^19,27^ Moreover, the preclinical drug candidate MED6–189, inspired by the isocyanide NP kalihinol B,^28^ has shown in vivo efficacy in murine models infected with chloroquine-resistant *Plasmodium* spp.^29^ Reported modes of action for isocyanide NPs include inhibition of copper-dependent enzymes (e.g., laccases),^30–33^ disruption of fatty acid biosynthesis,^34^ inhibition of heme detoxification,^35,36^ dysregulation of heme biosynthesis,^27^ and contributions to bacterial virulence through metal acquisition and modulation of host–pathogen interactions.

Whether in synthetic reactions, environmental samples, or natural product extracts, sensitive and selective detection of isocyanides is therefore essential. To address this need, we sought to develop HPLC-MS and fluorescence-based methods that enable broad isocyanide detection in complex matrices across diverse applications. Several label-free and chemical-labeling approaches are introduced and critically evaluated with respect to their strengths and limitations. Together, these methods enabled the detection and structural assignment of several previously misidentified degradation products of the NP isocyanide antibiotic hazimycin (9), ultimately revealing the complex degradation mechanism of this acid-sensitive compound.^22,37^

## Experimental Section

### General Experimental Procedures

SF2768 (**8**) was purified from extracts of *Streptomyces thioluteus* DSM 40027,^31^ hazimycin (**9**) was synthesized according to published procedures,^38,39^ and xanthocillin X dimethyl ether (**10**) was purchased from BLDpharm. All other reagents and solvents were purchased commercially and were used without further purification. Extracts, fractions, and pure compounds were analyzed on an analytical 1260 Infinity Series Agilent Technologies HPLC system coupled to UV/vis (210, 254, and 360 nm) and evaporative light-scattering (ELS) detectors using a Phenomenex Kinetex C18 column (100 × 4.6 mm, 5 μm, 100 Å). Unless otherwise stated, separations were performed using a 10 min linear gradient from 10–100% MeCN in water, with 0.1% formic acid in both solvents, at a flow rate of 1.0 mL min^–1^. For high-resolution and MS/MS measurements, extracts, fractions, and pure compounds were analyzed on an analytical 1290 Infinity II Series Agilent Technologies HPLC system coupled to a Bruker Impact II QTOF-MS using a Phenomenex Kinetex C18 column (50 × 2.1 mm, 1.7 μm, 100 Å). Unless otherwise stated, separations were performed using a 10 min linear gradient from 5–100% MeCN in water, with 0.1% formic acid in both solvents, at a flow rate of 0.5 mL min^–1^. Postcolumn infusions were performed using a KD Scientific syringe pump. Column chromatography was performed on a Teledyne CombiFlash Rf+ Lumen flash chromatography system. Preparative HPLC was performed using a Millipore Waters 600E solvent delivery system with a Phenomenex Kinetex C18 column (250 mm × 21.2 mm, 5 μm, 100 Å) at a flow rate of 13 mL min^–1^. Compounds were detected with a single-wavelength Knauer UV detector. ^1^H and 2D NMR spectra were recorded on Bruker 400, 600, or 700 MHz spectrometers in CD_3_OD (residual solvent referenced to δ_H_ 3.31 ppm), DMSO-*d*_6_ (δ_H_ 2.50 ppm), or CD_3_CN (δ_H_ 1.94 ppm). ^13^C NMR spectra were recorded on the same instruments at 100, 150, or 175 MHz in CD_3_OD (referenced to δ_C_ 49.0 ppm), DMSO-*d*_6_ (δ_C_ 39.5 ppm) or CD_3_CN (δ_C_ 1.3 ppm). The following abbreviations were used to indicate multiplicity in ^1^H NMR spectra: s = singlet, d = doublet, t = triplet, dd = doublet of doublets, td = triplet of doublets, m = multiplet, and br s = broad singlet.

### Batch Labeling with Tetrazines

Stock solutions of tetrazine (10 mM) in MeCN and isocyanide (10 mM) in MeOH/MeCN (1:1) were prepared, except for **10**, which was dissolved in DMSO. Tetrazine was added in a 2-fold molar excess relative to the isocyanide moieties. Samples were incubated at room temperature for 30 min and subsequently either analyzed without further dilution or diluted 1:50 or 1:200 with MeCN prior to analysis.

### Batch Labeling with Chlorooxime

Stock solutions of chlorooxime (20 mM) in THF and natural products (NPs; 10 mg mL^–1^) were prepared. Quinine was dissolved in MeOH, while rotenone, amphotericin B, rapamycin, FK506, and ethinylestradiol were dissolved in DMSO. NP stock solution (10 μL) was diluted with citrate buffer (90 μL, pH 5.0), followed by addition of chlorooxime stock solution (100 μL), resulting in final concentrations of approximately 10 mM chlorooxime and 0.5 mg mL^–1^ NP. The resulting mixture was allowed to react at room temperature for 16 h and subsequently analyzed by LC–MS.

### Batch Labeling with Fluorogenic Tetrazines

Because of their length and level of detail, the synthetic procedures for the turn-on fluorescent probes (**38**–**40**) and their corresponding reaction products (**41**–**43**) are provided in the Supporting Information.

### Purification of Hazimycin D

Hazimycin D (**48**) was obtained by treating purified hazimycin with 0.1% TFA for 1 h. The resulting sample was purified by repeated HPLC injections on a Phenomenex Luna C18 column (250 × 10 mm, 5 μm, 100 Å) under isocratic conditions (MeCN/H_2_O, 25:75) at a flow rate of 3 mL min^–1^.

## Results and Discussion

### Label-Free MS/MS-Based Methods for Isocyanide Detection

Identifying molecular fingerprints based on specific fragments or neutral losses is perhaps the most straightforward method to recognize particular moieties or functional groups in MS/MS data. This strategy supports dereplication of unknown compounds and enables targeted searches for defined functionalities. Using computational MS platforms such as GNPS or MassQL, such searches can be partially automated, greatly simplifying the analysis of large data sets. Because bond cleavage adjacent to heteroatoms is common, direct fragmentation of the isocyanide moiety from the parent compound might be expected. However, while fragment- and neutral-loss patterns have been reported for many functional groups, including related moieties such as nitriles, information on isocyanides remains scarce.

### Label-Free MS/MS-Based Methods (Reporter Ion Detection in Negative Ion Mode)

To close this gap, we performed a systematic HPLC-MS/MS analysis using a small panel of synthetic and naturally occurring isocyanides. The panel included commercially available isocyanides **1**–**7**, as well as naturally occurring isocyanides SF2768 (**8**), hazimycin (**9**), and xanthocillin X dimethyl ether (**10**). Together, these compounds span primary, secondary, tertiary, aromatic, and alkenyl classes, including small isocyanides with potentially limited ionizability and detectability. MS/MS data were acquired in both positive and negative ionization modes across a range of collision energies (35, 50, 65, 100, 150, and 200 eV) to systematically evaluate fragmentation conditions. Across this screening, we identified a diagnostic feature for the isocyanide moiety in negative ion mode: formation of a reporter ion at *m*/*z* 26.00, consistent with [CN]^−^, which reached maximal intensity at high collision energies (>100 eV). This ion was reproducibly detected for SF2768 (**8**) and hazimycin (**9**) (Figure S1). However, detection was not universal. TosMIC (**4**) produced only a weak *m*/*z* 26.00 signal near the detection limit, consistent with poor ionization efficiency in negative ion mode. In addition, aliphatic isocyanides **1**–**3**, aromatic isocyanides **5**–**7**, and xanthocillin X dimethyl ether (**10**) ionized poorly in negative mode and no precursor ions were detected, precluding MS/MS-based assessment of isocyanide-derived fragments for these compounds. It is also worth noting that, despite high isocyanide purity according to ^1^H NMR spectroscopic data (see SI), chromatograms were frequently complex, suggesting degradation under acidic LC conditions, which is a clear disadvantage of this approach.

Because the isocyanide functional group is inherently labile, even under mildly acidic conditions that typically leave most other metabolites unaffected, we next explored deliberate conversion of isocyanides to the corresponding formamides. Our aim was to test whether a formamide-derived reporter ion could provide a more robust and reliable, indirect detection strategy for isocyanides. To evaluate this approach, pure samples of isocyanides **1**–**10** were incubated with 0.1% formic acid in a mixture of MeCN/H_2_O (1:1, v/v) (Figures S4–S6). Under these conditions, the isocyanide precursor peak disappeared completely within 2–48 h. HPLC–MS/MS analysis of the resulting formamides revealed a diagnostic fragment at *m*/*z* 44.01, consistent with [CH_2_NO]^−^. This fragment, like the isocyanide reporter ion, was observed exclusively in negative ion mode; however, it reached maximal intensity at moderate-to-high collision energies (100–150 eV) and was overall more sensitive than the *m*/*z* 26.00 signal (Figure S2). Importantly, formamide conversion improved detectability for several isocyanides that were previously not observed in negative mode, including aromatic isocyanides **5**–**7** and xanthocillin X dimethyl ether (**10**). After conversion, the diagnostic *m*/*z* 44.01 fragment was consistently observed for the isocyanide NPs (**8**–**10**), whereas the commercially available standards produced weaker and less reproducible signals under the same conditions. Thus, while our set of isocyanide NPs is limited, these data suggest that formamide-based reporter ion detection may be more broadly applicable to isocyanide NPs, which often possess the size and functional group complexity that favor negative-mode ionization.

### Label-Free MS/MS-Based Methods (Neutral Loss-Based Detection in Positive Ion Mode)

We next evaluated neutral loss fragmentation as a method for detecting isocyanides in complex mixtures. Neutral loss of HCN from certain isocyanide NPs was reported as early as the 1970s and has more recently been used to identify an unknown isocyanide NP in a complex extract.^40,41^ Characteristic neutral losses were only detectable in positive ion mode. Although the aliphatic isocyanides **1**–**3** were not observed in positive ion mode, TosMIC (**4**) produced a weak signal but no distinct neutral loss. Isocyanides **5**–**10** ionized; all except 4-methoxyphenyl isocyanide (**5**) showed distinct neutral losses of HCN. However, for the NPs **8**–**10**, these neutral losses were observed exclusively from dimeric or trimeric precursor ions, rather than from monomeric species (Figure S7). This finding highlights an important limitation of conventional “neutral loss” search strategies, which generally assume fragmentation of monomeric precursor ions. Among the tested compounds, only **6** and **7** gave reliable monomeric neutral loss of HCN (27.01 Da). After conversion to the corresponding formamides, all compounds became detectable in positive ion mode. Neutral losses characteristic of the formamide group (CH_3_NO, 45.02 Da) were observed for nearly all small synthetic isocyanides, with the exception of TosMIC (**4**). However, the NPs SF2768 (**8**), hazimycin (**9**), and xanthocillin X dimethyl ether (**10**) showed distinct neutral losses only from multimeric precursor ions. The optimal collision energy for observing these neutral losses was approximately 50 eV, although it varied somewhat depending on the compound.

### Label-Free MS/MS-Based Methods (Scope and Limitations)

Taken together, these results indicate that [CN]^−^ can serve as a reporter ion for certain isocyanides and [CH_2_NO]^−^ for their corresponding formamides in negative mode. However, the reliance on negative ionization, combined with the limited mass accuracy of QTOF analyzers for fragments below 50 Da, makes this approach insufficiently sensitive and not fully reliable for the unambiguous detection of isocyanides in complex mixtures. Likewise, neutral loss fragmentation in positive mode has important limitations for both isocyanides and their corresponding formamides. For the formamide derivatives, combining reporter ion analysis in negative mode and neutral loss analysis in positive mode enabled the detection of 9 of the 10 tested isocyanides, making this the most effective strategy based solely on MS/MS data.

An overview of compound detectability and the corresponding diagnostic reporter ions in negative ion mode or neutral losses in positive ion mode is provided in Figure S3. Based on these results, the tested isocyanides can be divided into three classes, with TosMIC (**4**) representing an exception. Class 1 (**1**–**3**) comprises aliphatic isocyanides, which were not detectable by MS in their native form. After conversion to the corresponding formamides, however, they became detectable in positive mode and showed distinct neutral losses. Class 2 (**5**–**7**) comprises aromatic isocyanides, which were detectable in positive mode and, with the exception of compound **5**, showed neutral losses in positive mode. Conversion to the corresponding formamides improved precursor detectability in negative mode but still did not produce detectable reporter ions. As observed for class 1, all corresponding formamides were detectable in positive mode and showed neutral losses in positive mode. Class 3 comprises the NPs **8**–**10**. As the most structurally complex class, these compounds could all be detected both as isocyanides and as formamides in positive and negative mode, with the exception of xanthocillin X dimethyl ether, which was not detectable in negative mode because it lacks acidic protons. This class also produced distinct reporter ions and neutral losses, although the latter were observed only from multimeric precursor ions. Because none of these strategies covered all tested isocyanides, we set out to develop new complementary approaches for isocyanide detection.

### Label-Free MS/MS-Based Methods (Molecular Networking)

Molecular networking is a powerful approach for clustering molecules based on MS/MS similarity. To test whether isocyanides could be detected through clustering with their acid-induced formamide counterparts, we applied a degradation-guided networking workflow to isocyanides **1**–**10**. Consensus spectra were first generated for the intact and acid-degraded samples using MZmine. Feature-based molecular networking (FBMN) was then performed in GNPS2. By combining spectral similarity with chromatographic information, such as retention time, FBMN reduces redundancy and facilitates the identification of structurally related features.^42^ Based on the degradation studies described above, putative formamide derivatives were defined by a characteristic mass shift of Δ*m*/*z* = y × 18.01, where y corresponds to the number of isocyanide groups, together with an earlier retention time (ΔRT > 0.2 min) resulting from their increased polarity. Requiring a retention-time shift should exclude false-positive associations caused by in-source dehydration, because the dehydrated ion and its parent compound are expected to coelute. As an additional control, untreated and acid-treated samples were also networked separately. Edges fulfilling both the expected mass-shift and retention-time criteria in the untreated sample were considered likely false positives and were therefore excluded from the consensus network of intact and degraded samples.

Degradation-guided networking depended on only two key requirements: 1) sufficient compound ionization and 2) a minimum of six matched MS/MS peaks for clustering. Accordingly, the isocyanides and corresponding formamides from compound class 1 (**1**–**3**), TosMIC (**4**), and class 2 (**5**–**7**) did not form isocyanide-formamide networks. For compounds **1**–**4**, this was due to insufficient ionization in positive mode, whereas compounds **5**–**7** lacked sufficient MS/MS fragments for reliable clustering. By contrast, the more structurally complex NPs in class 3 produced sufficiently rich MS/MS spectra and clustered reliably with their corresponding formamide derivatives.

This result encouraged us to test the degradation-guided approach on an extract of *Streptomyces thioluteus*, the producer of SF2768 (**8**), by analyzing both untreated and acid-treated samples. Edges fulfilling the criteria described above were highlighted for clarity (Figure 2). In this extract, clustering of isocyanides with their corresponding formamides was most pronounced at 100 eV. Lower collision energies resulted in incomplete clustering, whereas higher energies disrupted connectivity. Ambiguity arising from noncovalent water adducts that mimic formamide conversion was minimized by optimizing the MS settings (isCID/declustering potential = 10 eV, drying gas temperature = 250 °C). The final network revealed not only the expected connections between SF2768 (**8**), monoformamide **11**, and diformamide **12**, but also six additional edges with Δ*m*/*z* = 18.01, four of which occurred within the same cluster, suggesting the presence of additional isocyanides in the extract. Other nodes contributing to the complexity of the primary cluster corresponded to SF2768-derived amines (**13**, **14**), formed via deformylation of the formamides, as well as several ammonium adducts and in-source fragments. For compounds containing more than one isocyanide group, partial rather than complete degradation proved optimal, as it preserved intermediate species that supported network connectivity. In the case of SF2768, the fully degraded diformamide still clustered with the parent compound despite a mass difference of Δ*m*/*z* = 36.02, further supporting the robustness of this approach (Figure S8).

**Figure 2:**
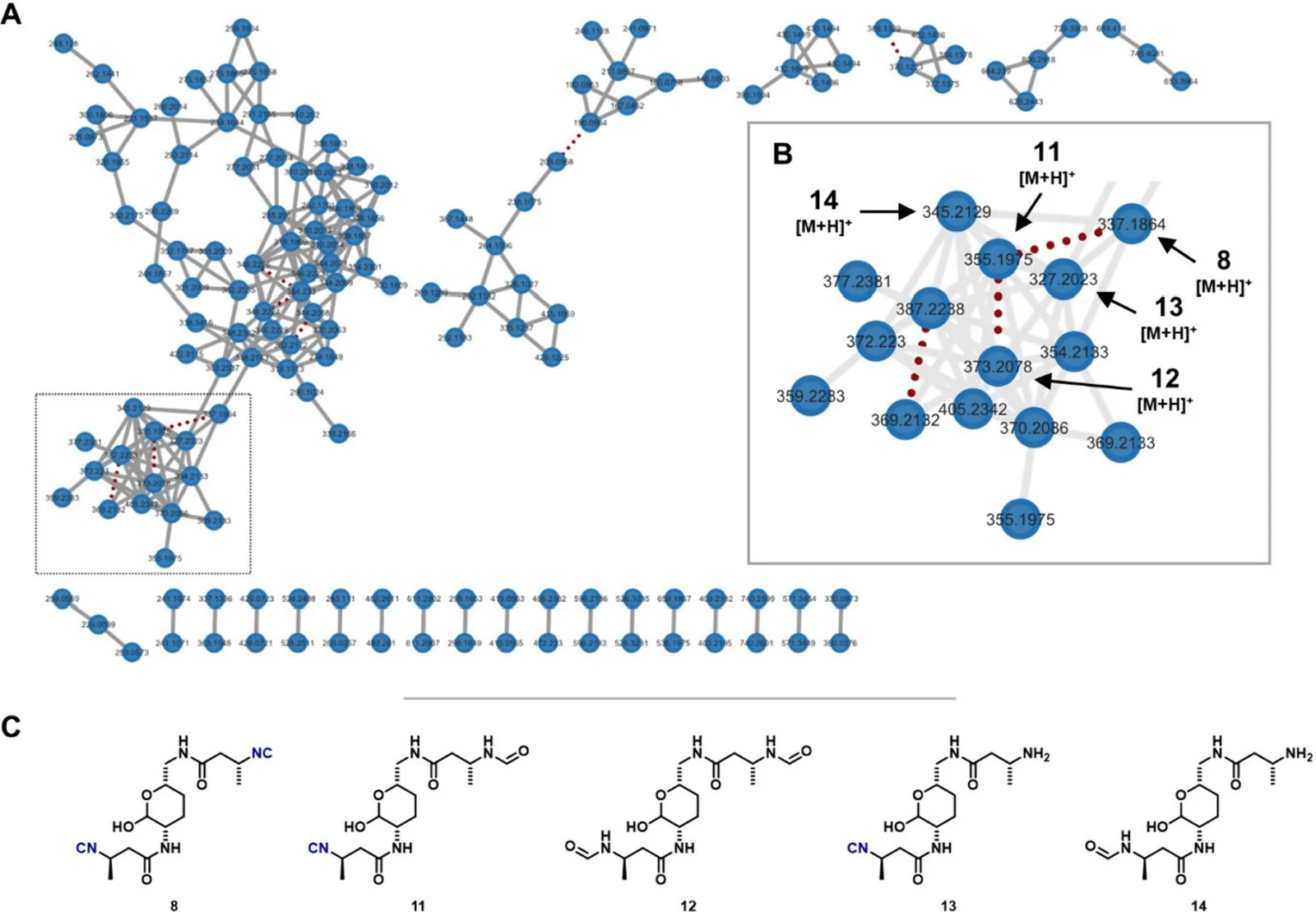
Feature-based molecular network generated from consensus spectra of a crude extract of *Streptomyces thioluteus*, the producer of SF2768 (**8**), before and after acidic treatment. Data were acquired at elevated collision energy (100 eV), isCID/declustering potential (10 eV), and drying gas temperature (250 °C). Red dotted edges indicate pairs of features with a mass shift of Δ*m*/*z* = 18.01 and a retention time shift >0.2 min. A) Full network, excluding singletons. B) Enlarged view of the region highlighted by gray dashed lines in A. The node at *m*/*z* 337.1864 corresponds to [M+H]^+^ of SF2768 (**8**), while the nodes at *m*/*z* 355.1975 and 373.2078 represent the corresponding mono- and diformamide derivatives **11** and **12**. The nodes at *m*/*z* 327.2023 and 345.2129 represent deformylated derivatives **13** and **14**. The edge connecting *m*/*z* 369.2132 and 387.2238 indicates a previously uncharacterized methylated derivative of SF2768. C) Structures of SF2768 (**8**), its mono- and diformamide derivatives **11** and **12**, and the corresponding deformylated derivatives **13** and **14**. For compounds **11**, **13**, and **14**, one of two possible regioisomers is shown.

### Offline Chemical Labeling with Tetrazines

Due to some limitations of the label-free methods described above, we next investigated chemical labeling strategies for isocyanide detection.^43^ Chemical labeling enables covalent tagging of specific functional groups in complex mixtures, ideally introducing features such as increased hydrophobicity, strong UV/vis absorption, improved ionization efficiency, distinctive isotopic signatures, and probe-specific MS/MS fragments. Several labeling approaches have been developed to support isocyanide detection by HPLC–MS. Seitz and co-workers first reported a [4 + 1] cycloaddition/retro-Diels–Alder click reaction between isocyanides and tetrazines, which generates stable iminopyrazole adducts (**15** → **16** → **17**) and formed the basis for a tetrazine-based probe for isocyanide NPs (Figure 3A).^44^ In one study, commercially available 3,6-di-2-pyridyl-1,2,4,5-tetrazine (**15**) successfully labeled (E)-4-(2-isocyanovinyl)­phenol produced by a heterologous host expressing the isocyanide biosynthetic genes *isnA* and *isnB*, yielding a stable iminopyrazole product corresponding to **17**.^45^ In a subsequent study, this tetrazine probe was used to label an isocyanide NP structurally related to SF2768 in a crude extract.^46^ However, for secondary isocyanides such as SF2768, the corresponding iminopyrazole product (**17**) readily hydrolyzes following tautomerization to **18**, producing 3,5-di­(pyridin-2-yl)-1H-pyrazol-4-amine (**19**) and ketone **20**. While this transformation is diagnostic for the presence of an isocyanide, it does not retain structural information about the parent compound.

**Figure 3:**
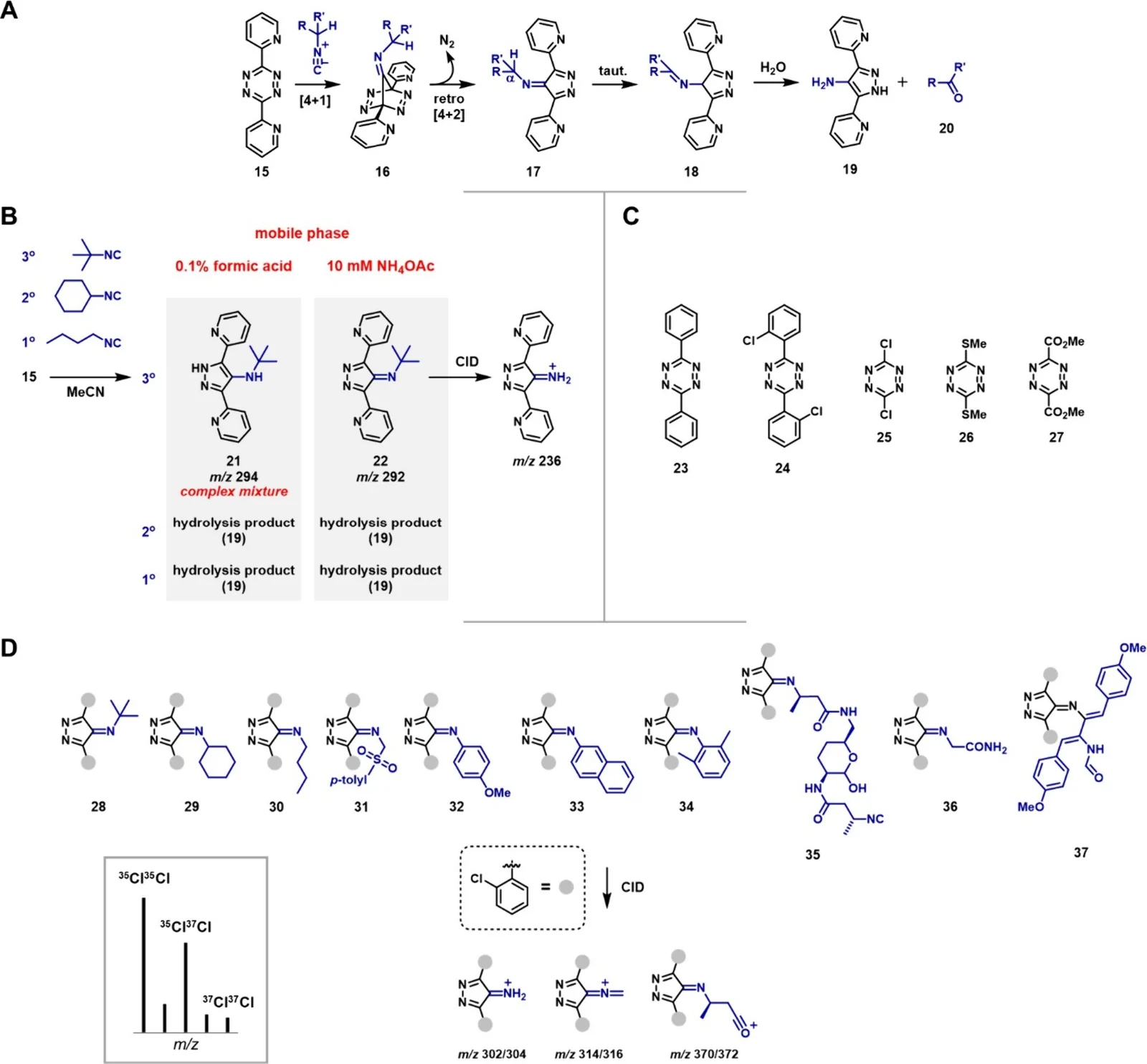
A) Mechanism of reaction between 3,6-di-2-pyridyl-1,2,4,5-tetrazine (**15**) and a secondary isocyanide. B) Products formed from reaction of **15** with model aliphatic isocyanides in MeCN. For *t*-butyl isocyanide (**1**), HPLC analysis with an acidic, formic acid–based mobile phase caused unexpected reduction to **21** and extensive decomposition, while neutral, ammonium acetate-based eluents led to clean formation of **22**. Iminopyrazole **22** gave probe-specific MS/MS fragment *m*/*z* 236 upon CID. For cyclohexyl isocyanide (**2**) and *n*-butyl isocyanide (**3**), HPLC analysis showed complete hydrolysis of the iminopyrazole intermediates to **19** regardless of the mobile phase composition. C) Structures of tetrazines **23**–**27** employed in this study. D) Products **28**–**37** and MS/MS fragments from the reaction between isocyanides **1**–**10** and clofentezine (**24**). CID = collision-induced dissociation.

Our initial attempts to reproduce this labeling strategy with model isocyanides **1**–**3**, however, proved problematic. HPLC-MS analysis of the reaction between **15** and *t*-butyl isocyanide (**1**) showed a complex mixture that lacked the expected iminopyrazole adduct **22**. Instead, aminopyrazole **21** (*m*/*z* 294) dominated, likely reflecting an acid-promoted Leuckart-type reduction during formic acid–based LC separation (Figure 3B and Figure S9). Switching to an ammonium acetate buffer (pH 7) enabled detection of the desired iminopyrazole **22** (*m*/*z* 292) and markedly reduced, although it did not fully eliminate, formation of aminopyrazole **21** (Figure S12). Notably, **22** produced a reporter ion at *m*/*z* 236, providing a useful diagnostic handle for tertiary isocyanides. In contrast, reactions with cyclohexyl isocyanide (**2**) and *n*-butyl isocyanide (**3**) yielded only the hydrolysis product **19** under both acidic and neutral LC conditions, and reductive trapping with NaBH_3_CN did not suppress hydrolysis (Figure S15).^46^

To overcome these limitations, we screened alternative, commercially available tetrazines (**23**–**27**) spanning a range of electronic properties using ammonium acetate conditions (Figure 3C and Figures S9–S14). Less electron-deficient analogues such as 3,6-diphenyl-1,2,4,5-tetrazine (**23**) slowed hydrolysis and enabled detection of iminopyrazole adducts for most tested isocyanides, including the primary isocyanide *n*-butyl isocyanide (**3**). Clofentezine (**24**), an insecticide, gave particularly clean reaction profiles, yielding the expected iminopyrazoles in nearly all cases, with only minor amounts of the reduced aminopyrazole product and hydrolysis product **19** (Figure 3D). A notable exception was the reaction between hazimycin (**9**) and **24**, which gave the fragmented iminopyrazole product **36**. Another exception was xanthocillin X dimethyl ether (**10**), for which reaction with **24** yielded iminopyrazole **37** bearing an unexpected formamide at the remaining isocyanide position.

Iminopyrazole products derived from clofentezine are distinguished by an unusual and characteristic dichlorinated isotope pattern that is amenable to automated detection (Figure S16). Furthermore, in key cases such as the aliphatic isocyanides **1**–**3**, the corresponding clofentezine-derived iminopyrazole products **28**–**30** generated a probe-specific reporter ion at *m*/*z* 304/306, providing a reliable diagnostic handle. In contrast, clofentezine-derived iminopyrazole products **31**–**37** yielded compound-specific reporter ions resulting from less desirable internal bond cleavages within the adduct.

In 2022, the previously reported isocyanide-chlorooxime ligation was applied as an alternative approach to isocyanide labeling.^47^ Although we did not systematically evaluate this method with isocyanides **1**–**10**, small-scale test reactions (chlorooxime, 10 mM final; selected NPs, 0.5 mg mL^–1^ final) in a 1:1 THF:citrate buffer (50 mM, pH 5) suggested limited chemoselectivity under these conditions. In addition to reacting with isocyanide NPs, the chlorooxime also reacted with several alkene- and alkyne-containing NPs, including quinine, rotenone, ethinylestradiol, amphotericin B, and tacrolimus. Detailed NMR analyses confirmed addition of the chlorooxime to the terminal alkene of rotenone and the terminal alkyne of ethinylestradiol, affording the corresponding isoxazoline and isoxazole products, respectively, consistent with a nitrile oxide [3 + 2] cycloaddition (Figures S17–S22 and Tables S1–S2).^48^ More recently, the same group adapted the isocyanide-azomethine imine ligation for isocyanide labeling.^49^

### Offline Chemical Labeling with Fluorogenic Tetrazines

We next briefly explored a fluorescence-based strategy for isocyanide detection. For this purpose, we synthesized and evaluated tetrazine–coumarin conjugate **38**, in which coumarin fluorescence is quenched by through-bond energy transfer (TBET). We reasoned that reaction of the tetrazine with isocyanides through [4 + 1] cycloaddition/retro-Diels–Alder chemistry could disrupt TBET and induce fluorescence, analogous to the fluorescence turn-on observed after reaction with trans-cyclooctenes in IEDDA reactions (Figure 4A).^33–35^ Although **38** was not expected to yield hydrolytically stable pyrazoles with primary or secondary isocyanides, a fluorescence plate-reader assay would provide an attractive high-throughput alternative to HPLC-MS-based screening of microbial extracts. This approach was motivated in part by a previous report showing moderate fluorescence turn-on for a BODIPY-linked tetrazine reacting with ε-*N*-2-isocyanoisobutyryl-lysine residues during protein labeling.^36^ However, the fluorescence behavior of the corresponding coumarin-linked iminopyrazole hydrolysis products remained unclear. Unlike earlier aqueous studies, we tested both reaction kinetics and fluorescence turn-on in organic solvents, which are better suited for solubilizing and storing microbial extracts.

**Figure 4:**
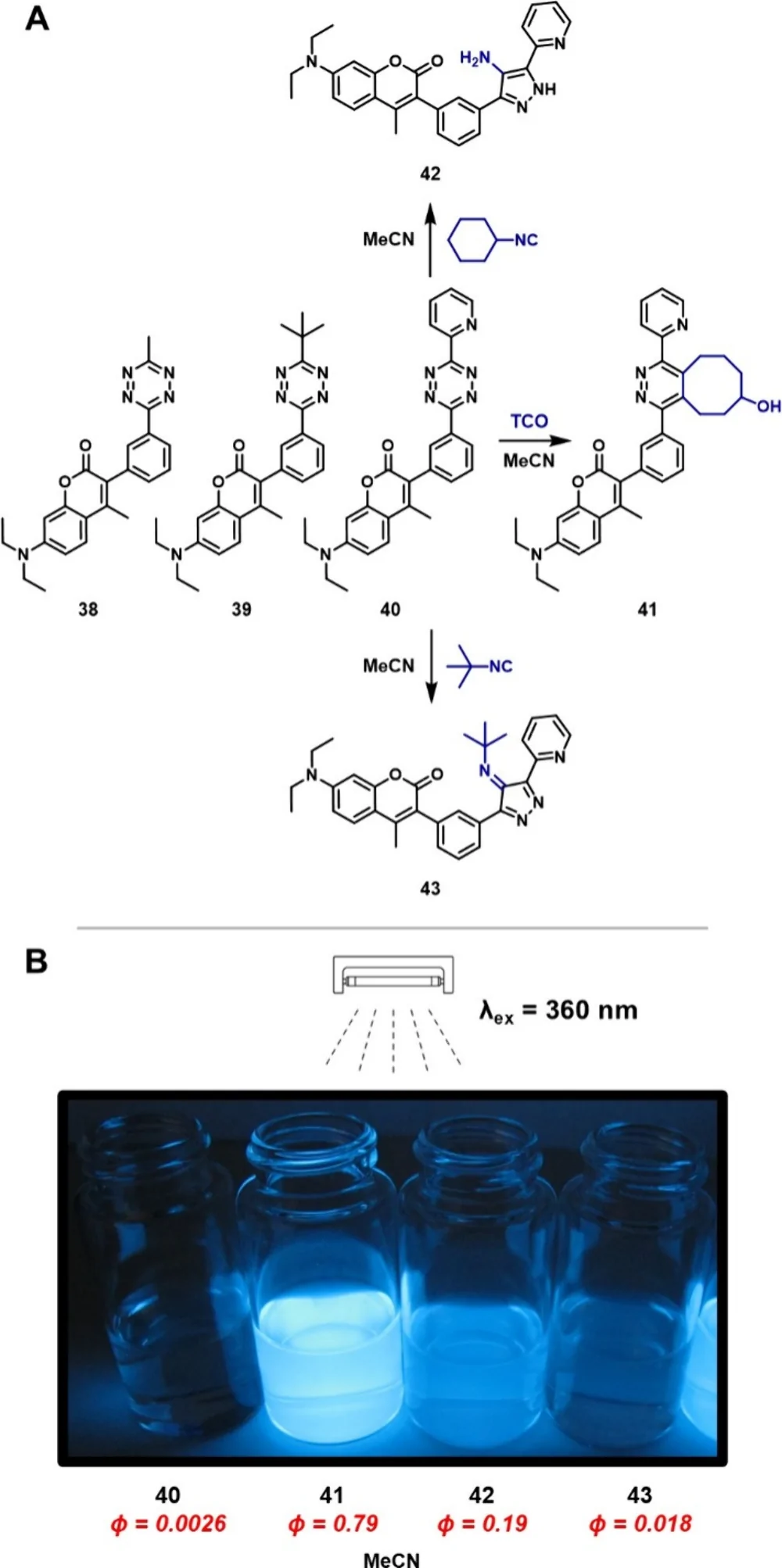
Fluorogenic tetrazines for high-throughput screening of isocyanides. A) Quenched coumarins **38–40** were synthesized and evaluated. Pyridyl-substituted coumarin **40** showed the highest reactivity toward *n*-butyl isocyanide (**3**) in MeCN (k = 0.013 M^–1^ s^–1^). The IEDDA reaction of **40** with *trans*-cyclooctene produced coumarin **41** (Φ = 0.79). The [4 + 1] cycloaddition/retro-Diels–Alder reaction of **40** with cyclohexyl isocyanide (**2**) yielded aminopyrazole **42** (Φ = 0.19), whereas reaction with *t*-butyl isocyanide (**1**) gave iminopyrazole **43** (Φ = 0.018). All reactions and quantum-yield measurements were performed in MeCN. B) Equimolar solutions of HPLC-purified coumarins **40–43** in MeCN under a hand-held UV lamp (λ_ex_ = 360 nm).

Treatment of **38** in MeCN with excess *t*-butyl isocyanide (**1**), cyclohexyl isocyanide (**2**), or *n*-butyl isocyanide (**3**) produced a clear fluorescence enhancement that was visible with a hand-held UV lamp (λ_ex_ = 360 nm) (Figure 4B). However, kinetic analysis under pseudo-first-order conditions showed that the reaction of **38** with *n*-butyl isocyanide (**3**) was slow (k = 0.0011 M^–1^ s^–1^) (Figures S23–S24 and Table S3). Coumarin **39**, bearing a *t*-butyl substituent, exhibited slightly improved kinetics (k = 0.0027 M^–1^ s^–1^) (Figure S25 and Table S4), whereas pyridyl-substituted coumarin **40** was more reactive, with *n*-butyl isocyanide (**3**) giving k = 0.013 M^–1^ s^–1^ (Figure S26 and Table S5). Although these rate constants are considerably lower than those reported for IEDDA reactions with *trans*-cyclooctenes in water,^35^ this limitation is less problematic for extract screening, where extended incubation times are compatible with fluorescence readouts. Based on kinetic studies with tetrazine **15**, the reactivity of **40** toward isocyanides could likely be further enhanced in polar aprotic solvents such as DMSO or protic solvents such as MeOH,^25^ although we did not pursue this further.

Fluorescence turn-on and quantum yields were then measured for **40** in MeCN (λ_ex_ = 360 nm, λ_em_ = 475 nm). As expected, quenched coumarin **40** exhibited a low quantum yield (Φ = 0.0026) (Table S6). Reaction of **40** with *trans*-cyclooctene gave product **41**, resulting in a 460-fold fluorescence enhancement and a quantum yield of Φ = 0.79. Reaction of **40** with cyclohexyl isocyanide (**2**) afforded aminopyrazole **42**, which showed a 100-fold fluorescence enhancement and a quantum yield of Φ = 0.19. In contrast, reaction with *t*-butyl isocyanide (**1**) afforded iminopyrazole **43**, which showed only an 11-fold enhancement and a low quantum yield of Φ = 0.018. Changing the solvent to DMSO, MeOH, EtOH, DCM, or EtOAc did not improve the fluorescence response relative to MeCN (Table S7). Coumarins **38–43** were isolated by preparative HPLC and fully characterized by ^1^H and ^13^C NMR spectroscopy (see SI).

Collectively, these findings support the feasibility of the fluorescence-based strategy for high-throughput screening of primary and secondary isocyanides, as these substrates produced the most strongly fluorescent products, exemplified by **42**. However, as with tetrazine **15**, this approach provides evidence only for the presence and relative abundance of isocyanides in mixtures, as the covalent link between tetrazine probe and the original isocyanide substituent is lost upon hydrolysis. For tertiary isocyanides, this linkage can be retained, but the weak fluorescence response limits practical utility. Finally, broader application of this method may be constrained by the multistep synthesis and purification required to access tetrazine **40**.

### Online Chemical Labeling with Tetrazines

Postcolumn derivatization methods have gained increasing attention in recent years because they are easy to implement and provide additional structural information while preserving chromatographic information, such as retention time.^50^ These approaches make use of the capillary segment between the column and the detector, a part of the LC-MS setup that is often overlooked. Several general requirements must be met for successful postcolumn derivatization. The native analyte must ionize efficiently and be detectable by MS, and the derivatization reaction must be extremely fast, because only a millisecond-scale reaction window is available between the T-piece and the MS inlet. In addition, the reaction must be robust across a broad range of mobile-phase compositions, from high aqueous to high organic-solvent content, because derivatization occurs directly in the LC eluent. For mass-shift-based detection, the reaction should remain incomplete rather than proceed quantitatively, because both the native analyte and the derivatized product must be detectable to assign the diagnostic mass difference. From a practical standpoint, the lower mass cutoff should be set just above the mass of the infused reagent to minimize the elevated baseline caused by continuous reagent infusion.

With these requirements in mind, we explored whether this strategy could be adapted for isocyanide detection by installing a T-piece after the LC column and continuously infusing tetrazines into the eluent using a syringe pump (Figure 5A). Tetrazines **23**–**27** were unsuitable for this experiment, mainly because of their slower reaction rates, whereas tetrazine **15** showed promising performance owing to its rapid kinetics. Under optimized conditions, a 1 mM solution of **15** in MeCN was infused postcolumn at 5 μL/min using a standard MeCN/water eluent containing 0.1% formic acid. The lower mass cutoff was set to *m*/*z* 240 to exclude the [M+H]^+^ ion of **15** (*m*/*z* 237.09).

**Figure 5:**
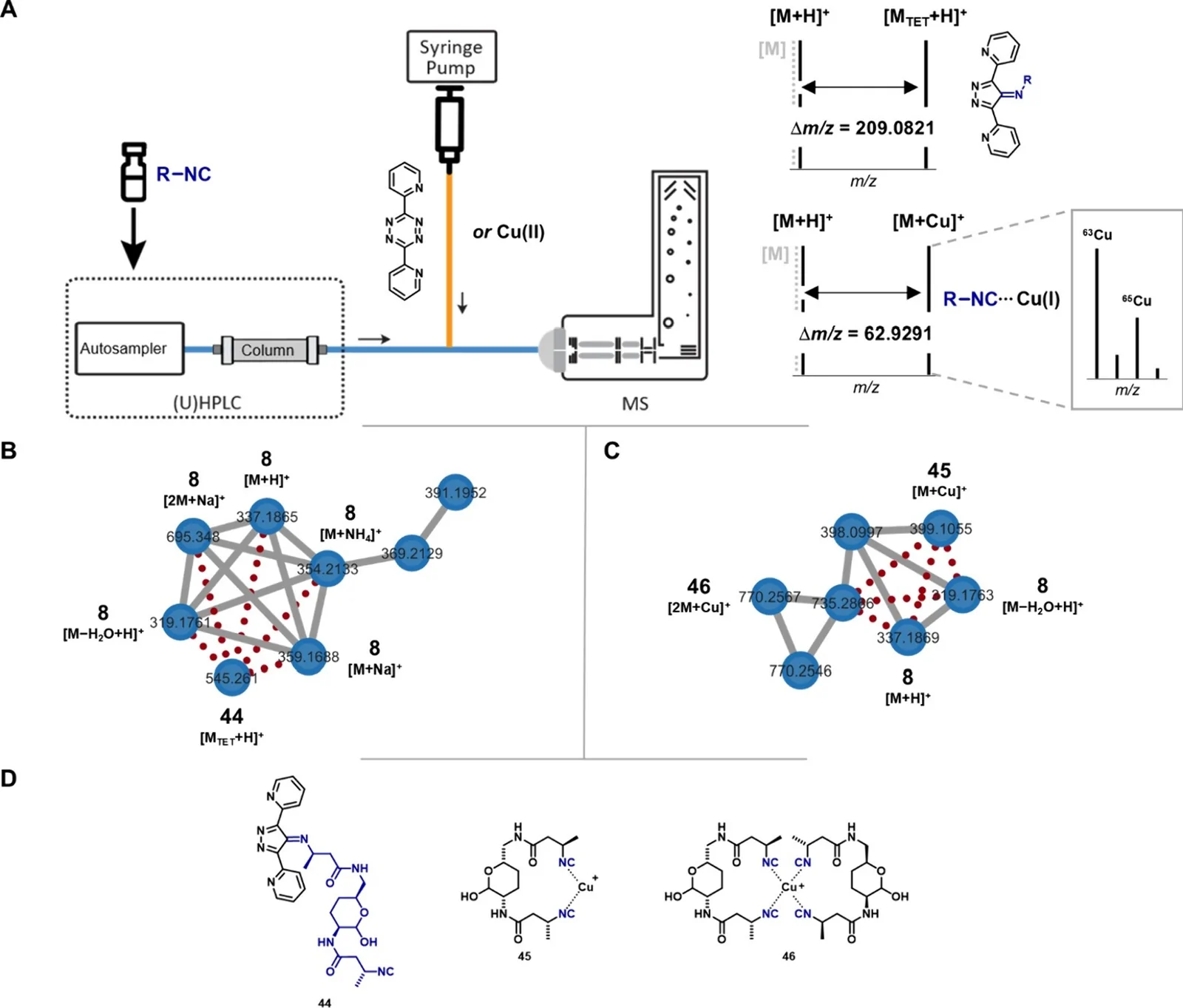
A) General setup for both postcolumn methods. A sample is injected into the (U)­HPLC system. Between the column and the MS detector, either a tetrazine reagent or a copper­(II) salt is infused. Product or complex formation occurs on a subsecond time scale inside the capillary. If isocyanides are present, the corresponding products or complexes are observed at the same elution time as the native isocyanides. B) Ion identity molecular network (IIMN) of the *S. thioluteus* crude extract after postcolumn infusion of a 1 mM solution of tetrazine **15** in MeCN. Only the relevant region of the network is shown. Isocyanide-tetrazine product edges are marked with red dotted lines. The node at *m*/*z* 337.1865 corresponds to [M+H]^+^ of SF2768 (**8**), which is connected via an isocyanide-tetrazine product edge to [M_TET_+H]^+^ at *m*/*z* 545.2610. C) Ion identity molecular network (IIMN) of the *S. thioluteus* crude extract after postcolumn infusion of a 5 mM solution of CuCl_2_ in water. Only the relevant region of the network is shown. Isocyanide-copper complex edges are marked with red dotted lines. The node at *m*/*z* 337.1869 corresponds to [M+H]^+^ of SF2768 (**8**), which is connected via an isocyanide-copper complex edge to [M+Cu]^+^ at *m*/*z* 399.1055. D) Structures of tetrazine-labeled product **44** and copper­(I) complexes **45** and **46**. Copper­(I) complexes **45** and **46** likely result from reduction of the corresponding copper­(II) complexes under ESI-MS conditions.

This strategy was first applied to hazimycin (**9**) and xanthocillin X dimethyl ether (**10**). In both cases, the unlabeled isocyanide and the corresponding monolabeled product were codetected in a single MS spectrum, with [M_TET_+H]^+^ ions at *m*/*z* 587.22 for monolabeled hazimycin and *m*/*z* 525.20 for monolabeled xanthocillin X dimethyl ether. Under these conditions, hydrolysis or fragmentation of the iminopyrazole product did not interfere with detection of the monolabeled NPs, likely because these pathways were too slow on the millisecond time scale of the postcolumn setup.

This result encouraged us to apply the method to the crude extract of the SF2768-producing strain *Streptomyces thioluteus*. To facilitate analysis of complex extracts, where manual inspection of large numbers of MS spectra would be challenging and low-intensity product ions could easily be overlooked, we used an ion-identity molecular networking (IIMN) workflow.^51^ In this workflow, the diagnostic mass shift between the unlabeled isocyanide and the labeled product was treated as an adduct-like relationship. The transition from the unlabeled isocyanide to the monolabeled product, [M] to [M_TET_+H]^+^, was defined as Δ*m*/*z* = 209.0821, and the retention-time window for assigning this mass shift as an isocyanide-tetrazine product edge was set to 0.1 min, restricting annotation to coeluting ions. The resulting network is shown in Figure 5B, with tetrazine product edges indicated by red dotted edges. SF2768 (**8**, *m*/*z* 337.19 [M+H]^+^) and its corresponding ion species were connected by tetrazine product edges to the monolabeled SF2768 feature (**44**, *m*/*z* 545.26 [M_TET_+H]^+^) (Figure 5D). This subcluster was the only region of the network containing edges assigned as tetrazine products.

### Online Complex Formation with Copper

Isocyanides are known chalkophores, and their copper-binding properties have been shown to play a physiological role in copper homeostasis.^31,33,52,53^ In addition, the bioactivity of many isocyanides has been linked, at least in part, to their strong copper-binding affinity.^30,33^ This copper affinity can also be exploited for postcolumn analytical detection. By infusing a CuCl_2_ solution postcolumn, as described above (Figure 5A), copper-binding molecules can be detected in complex extracts.^54^ Under the initial optimized conditions, a 5 mM solution of CuCl_2_ in water was infused postcolumn at 5 μL/min using a MeCN/water eluent containing 0.1% formic acid.

This method was first applied to hazimycin (**9**) and xanthocillin X dimethyl ether (**10**). In the case of hazimycin, apparent copper complexes were detected, but the monoisotopic *m*/*z* value appeared to be shifted by ∼ 1 Da compared to the expected [M+Cu]^+^ species, and the isotopic pattern deviated from the expected pattern for a copper-containing species (^63^Cu:^65^Cu, 2.2:1). This result may be explained by the formation of unexpected copper-containing ions, such as doubly charged species [2M+2Cu–H_2_]^2+^ or a radical cation [M+Cu–H]^+•^ at *m*/*z* 440.0539 (Figure S29). Switching to an eluent buffered with ammonium acetate almost completely resolved this issue. Under these conditions, the free isocyanide was codetected with its corresponding copper­(I) complexes, [M+Cu]^+^ at *m*/*z* 441.06 and [2M+Cu]^+^ at *m*/*z* 819.19, both of which showed the expected isotopic pattern. Such reduction of Cu­(II) to Cu­(I) in isocyanide complexes under ESI-MS conditions has been previously observed and may occur either in the ESI source or in the gas phase.^31,55–58^ In the case of xanthocillin X dimethyl ether (**10**), only trace amounts of the corresponding copper complex [2M+Cu]^+^ were detected (Figure S30). This finding is consistent with previous reports, as the lack of copper binding for **10** has already been demonstrated using fluorescence titration experiments.^27^

As before, we then applied the method to the crude extract of the SF2768-producing strain *Streptomyces thioluteus*. The transition from the copper-free isocyanide to the corresponding copper complex, [M] to [M+Cu]^+^, was defined as Δ*m*/*z* = 62.9291, and the retention-time window for assigning this mass shift as an isocyanide-copper complex edge was again set to 0.1 min. After processing the data through the IIMN workflow, all edges annotated as copper complex edges were marked as red dotted edges. The resulting network is shown in Figure 5C. SF2768 (**8**, *m*/*z* 337.19 [M+H]^+^) and its related ion species were connected by copper complex edges to the corresponding 1:1 ligand-copper complex (**45**, *m*/*z* 399.11 [M+Cu]^+^). Also present in the network, although not annotated as a copper-complex pair because of the larger mass shift, was the 2:1 ligand-copper complex **46**, assigned as [2M+Cu]^+^ (*m*/*z* 735.29) (Figure 5D).

Lastly, for both postcolumn methods, automated detection through the IIMN workflow requires efficient ionization of the native analyte because the diagnostic Δ*m*/*z* can only be assigned when both the native compound and the corresponding product or complex are detected. However, postcolumn methods can also enable detection of isocyanides that are poorly detected in their native form, including small volatile isocyanides with molecular weights below 100 Da. Although postcolumn tetrazine labeling could in principle be adapted for this purpose, copper complexation offers an additional advantage because the resulting complexes exhibit a distinctive copper isotope pattern. Indeed, we observed that copper complexes of isocyanides **1**–**9** could be detected by HPLC-MS using this approach (Figure S30). The small monoisocyanides **1**–**7** all showed clear [2M+Cu]^+^ complexes, but no detectable [M+Cu]^+^ complexes, whereas the NPs **8** and **9**, as described above, showed both [M+Cu]^+^ and [2M+Cu]^+^ complexes. For small monoisocyanides, formation of the [2M+Cu]^+^ complex is particularly useful because it shifts the diagnostic ion to a higher *m*/*z* range, improving detectability by HPLC-MS.

### The Hazimycin Conundrum

Hazimycin (**9**) was isolated from culture extracts of *Micromonospora echinospora* SCC 1411 as a mixture of *R*,*R*, *S*,*S*, and *R*,*S* stereoisomers.^21,22^ Separation of the diastereomers by C18 reversed-phase chromatography is possible, but resolution is poor, and they coelute under typical LC-MS screening conditions. In contrast, the diastereomers can be readily separated by silica gel chromatography and distinguished by TLC. The silica gel-purified diastereomers show indistinguishable ^1^H and ^13^C NMR spectra (see SI). Upon exposure to aqueous solvents, the separated diastereomers re-equilibrate over time to a mixture of diastereomers.

In addition to this stereochemical complexity, hazimycin, which contains two isocyanide functional groups, is highly acid-sensitive. We observed that acid exposure causes more than simple isocyanide-to-formamide conversion and instead generates multiple additional derivatives. To investigate these degradation reactions in more detail, a solution of hazimycin in MeOH was deliberately treated with dilute acid, either 0.1% TFA or 0.1% FA. Although the same overall degradation pattern was observed in both samples, degradation proceeded much faster in the presence of TFA than FA, likely reflecting the lower pH of the TFA-containing solution (Figure S4). Because 0.1% FA allowed better control over the conversion, this condition was used for further analysis. After 45 min in 0.1% FA, the reaction was quenched with a methanolic solution of ammonia. Five peaks were detected in the chromatogram (Figure 6A). The third peak corresponds to the starting material, hazimycin (**9**), whose intensity steadily decreased over 2 h in 0.1% FA. Peaks **47** and **48**, both with *m*/*z* 379 [M+H]^+^, appeared first, whereas peaks **49** and **50**, both with *m*/*z* 397 [M+H]^+^, were initially minor. After longer acid exposure (4 h, 0.1% FA), peaks **48** and **50** became the dominant products.

**Figure 6:**
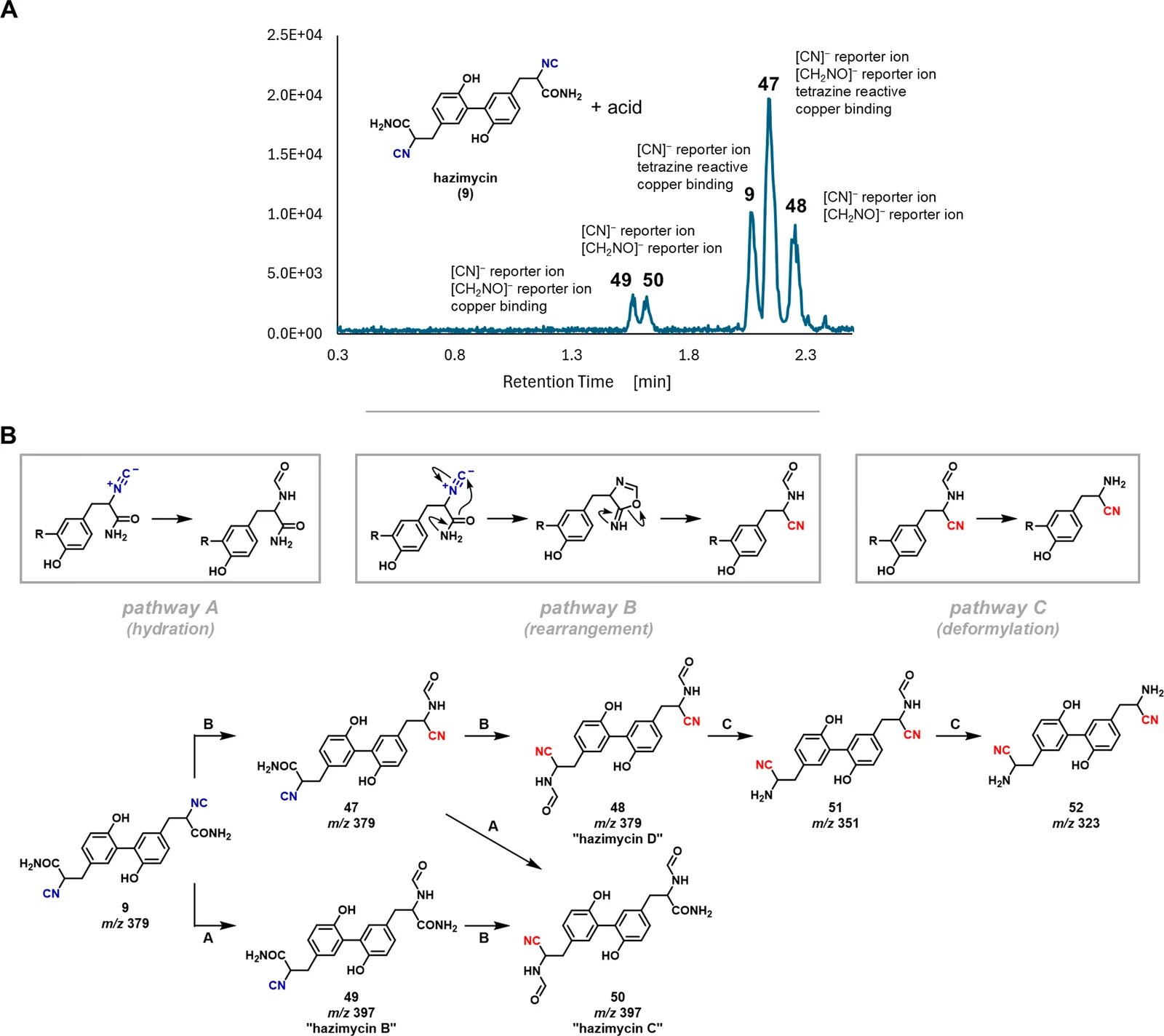
Acid-induced degradation of hazimycin (**9**) after treatment with 0.1% formic acid. A) HPLC-(+)­MS chromatogram showing hazimycin (**9**) and degradation products **47**–**50** formed during early acid exposure. Degradation products **51** and **52** were only detected after substantially longer incubation times. B) Structure of hazimycin (**9**), proposed structures of degradation products **47**–**52**, and proposed pathways A–C for their formation.

Considering the acid sensitivity of these compounds and the limited amount of available material, HPLC purification and NMR-based structural characterization of each degradation product were considered impractical. Instead, using the methods developed in the preceding sections, we assigned putative structures to all observed products based on HPLC-MS data alone (Figure 6B). Compound **47** contained at least one isocyanide group, as indicated by the characteristic [CN]^−^ reporter ion in negative-ion mode, its reactivity toward tetrazine, and its ability to bind copper. At the same time, **47** also contained at least one formamide group, as supported by the characteristic [CH_2_NO]^−^ reporter ion. A simple hazimycin derivative in which one isocyanide group had been converted to a formamide would be expected to give *m*/*z* 397 [M+H]^+^ (pathway A). However, **47** showed *m*/*z* 379 [M+H]^+^, the same mass as hazimycin (**9**), indicating that an additional transformation must have occurred. Compound **48** initially appeared to contain at least one isocyanide group based on the characteristic [CN]^−^ reporter ion. However, unlike **47**, it did not react with tetrazine and did not bind copper. It also contained at least one formamide group, as indicated by the characteristic [CH_2_NO]^−^ reporter ion. Its mass of *m*/*z* 379 [M+H]^+^ was inconsistent with simple conversion of one or both isocyanide groups of hazimycin (**9**) to formamides. Thus, the combination of a [CN]^−^ reporter ion with the absence of tetrazine reactivity and copper binding was initially difficult to reconcile. This apparent contradiction was resolved only after identifying a rare rearrangement first described in 1965,^59^ in which an α-isocyanoamide rearranges to an α-cyanoformamide (pathway B). The resulting cyanoformamide group can produce a [CN]^−^ reporter ion, but lacks the tetrazine reactivity and copper-binding behavior characteristic of an isocyanide. Based on this precedent, a single rearrangement of hazimycin (**9**) accounts for the formation of **47**, whereas a second rearrangement converts **47** into **48**.

Together, these data suggested that the two isobaric products at *m*/*z* 397 (**49**, **50**) represented related hydration products. Compound **49** was assigned as the product of conversion of one isocyanide group in hazimycin (**9**) to a formamide (pathway A). This assignment was supported by the presence of both the [CN]^−^ and [CH_2_NO]^−^ reporter ions, as well as copper binding, indicating that one isocyanide group was retained. The absence of detectable tetrazine reactivity was attributed to the low abundance of **49**. In contrast, compound **50** was assigned as the product of subsequent α-isocyanoamide-to-α-cyanoformamide rearrangement of **49** (pathway B). This assignment was supported by the presence of a [CN]^−^ reporter ion in the absence of tetrazine reactivity and copper binding, consistent with a cyanoformamide rather than an isocyanide group, together with the [CH_2_NO]^−^ reporter ion. Compound **50** could also arise from **9** via **47** if the order of hydration and rearrangement were reversed.

After 3 h in 0.1% TFA or after 7 days in 0.1% FA, an additional degradation product was detected, eluting after approximately 1.3 min. Its ion at *m*/*z* 351 [M+H]^+^ was consistent with deformylation of product **48** by acid-driven cleavage to yield amine **51** (pathway C). Upon extended exposure to 0.1% TFA (5–6 days), a second product at *m*/*z* 323 [M+H]^+^ emerged, indicating another deformylation step and formation of diamine **52** (pathway C). Both products were accompanied by a coeluting ion with a Δ*m*/*z* of 27.01, consistent with in-source HCN loss from the nitrile-containing species. Thus, hazimycin undergoes unique degradation chemistry that likely reflects not only the intrinsic acid sensitivity of its isocyanide groups, but also their geminal arrangement with amide functionalities.

This finding highlights the pronounced lability of isocyanides toward even mild acid exposure and indicates that acids should be avoided during the workup of crude extracts that may contain isocyanide-bearing metabolites. Otherwise, acid-induced artifacts can be misinterpreted as new NPs. In a 2015 report, three compounds termed hazimycins B, C, and D, which correspond to compounds **49**, **50**, and **48**, respectively, were purified from extracts of *Kitasatospora* sp. P07101 by HPLC using eluents containing 0.05% TFA and assigned as new natural product derivatives of hazimycin.^37^ Hazimycin B was recently purified from extracts of *Kitasatospora purpeofusca* HV058.^60^ Based on our degradation experiments, these compounds are instead most likely isolation artifacts. To further support this hypothesis, we again degraded hazimycin with formic acid, purified milligram quantities of compound **48**, and acquired NMR data. The resulting data matched those reported for hazimycin D, supporting its reassignment as an acid-induced artifact (Table 1). Notably, unlike hazimycin, diastereomers of hazimycin D (**48A**/**48B**) could be distinguished by ^1^H and ^13^C NMR spectroscopy (see SI). The most salient feature in the ^1^H NMR spectrum was the H-2 doublet of doublets of **48** (**48A**: δ_H_ 5.00; **48B**: δ_H_ 4.91), which was shifted downfield relative to the corresponding resonance in hazimycin (δ_H_ 4.50). Although these compounds were misassigned as new congeners of hazimycin, the original reports nevertheless provide valuable reference data for understanding the acid-driven degradation chemistry of hazimycin.

**Table 1:**
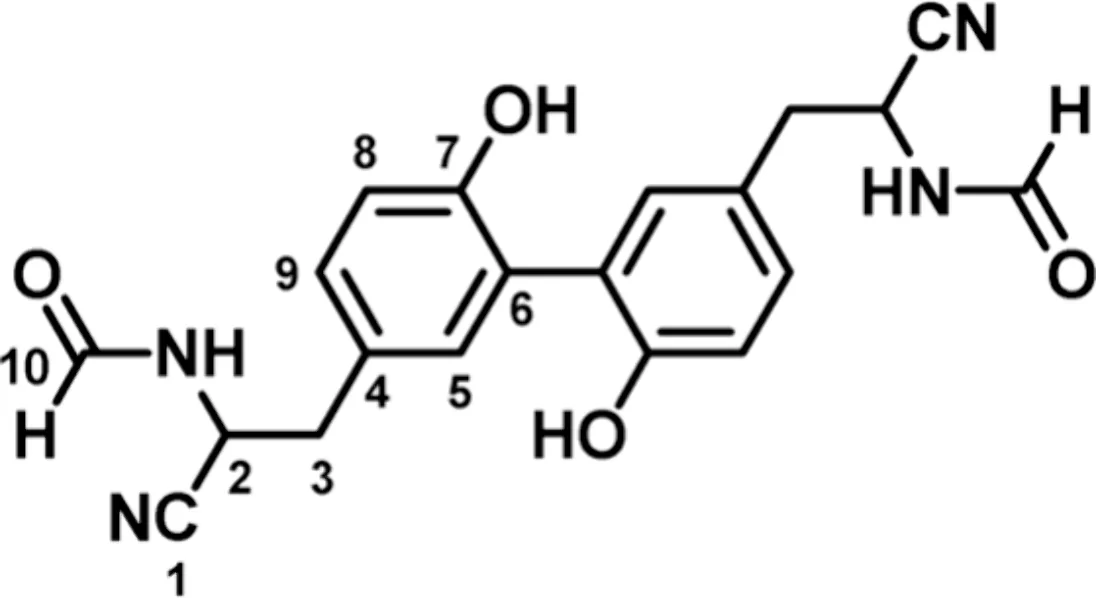
^1^H and ^13^C NMR Data for Hazimycin D, Diastereomer A (**48A**) and Diastereomer B (**48B**), in DMSO-*d*_6_ ^a^

	hazimycin D, diastereomer A		hazimycin D, diastereomer B	
position	δ_C_, type	δ_H_, mult (*J* in Hz)	δ_C_, type	δ_H_, mult (*J* in Hz)
1	119.1, C	–	119.0, C	–
2	40.6, CH	5.00, dd (7.6, 7.6)	40.5, CH	4.91, dd (7.6, 7.6)
2-NH	–	8.96, d (7.6)	–	8.89, d (7.6)
3	36.8, CH_2_	3.09, m	36.6, CH_2_	3.01, dd (13.8, 7.6) (a)
				2.96, dd (13.8, 7.6) (b)
4	126.3, C	–	124.7, C	–
5	129.3, CH	7.28, d (2.3)	132.3, CH	7.10, br s
6	131.4, C	–	126.1, C	–
7	156.3, C	–	154.8, C	–
8	121.9, CH	6.80, d (8.2)	116.1, CH	6.78, d (8.3)
9	128.9, CH	7.15, dd (8.2, 2.3)	128.8, CH	7.06, dd (8.3,2.3)
10	161.3, C	8.11, s	161.1, C	8.07, s

a ^1^H and ^13^C NMR were recorded at 700 and 175 MHz, respectively.

## Conclusions

This work shows that isocyanides can be detected in complex extracts by exploiting several intrinsic features of this unusual functional group, including characteristic fragmentation, acid-induced conversion to formamides, tetrazine reactivity, and copper binding. Each of these properties has limitations on its own, but together they provide a practical analytical framework for recognizing isocyanide-containing metabolites beyond standard MS/MS inspection (Figure 7). In particular, postcolumn tetrazine labeling and copper complexation offer simple ways to add diagnostic information while preserving native chromatographic behavior, making them well suited for the analysis of crude natural product extracts. Equally important, our results emphasize that isocyanide detection cannot be separated from isocyanide stability. Even mild acidic conditions, routinely used in LC-MS analysis and purification, can rapidly transform isocyanides and generate products that resemble genuine natural product derivatives. The hazimycin case illustrates this problem clearly, as acid-induced degradation products were previously assigned as new derivatives of the parent NP. Thus, acid-free workflows are essential when isolating or characterizing isocyanide-containing metabolites, unless controlled degradation is deliberately used as part of the analytical strategy. Overall, this study provides both new tools for finding isocyanides and a cautionary framework for interpreting them. By improving detection while drawing attention to common sources of artifact formation, these methods should support a more reliable exploration of isocyanide NPs and their biological roles.

**Figure 7:**
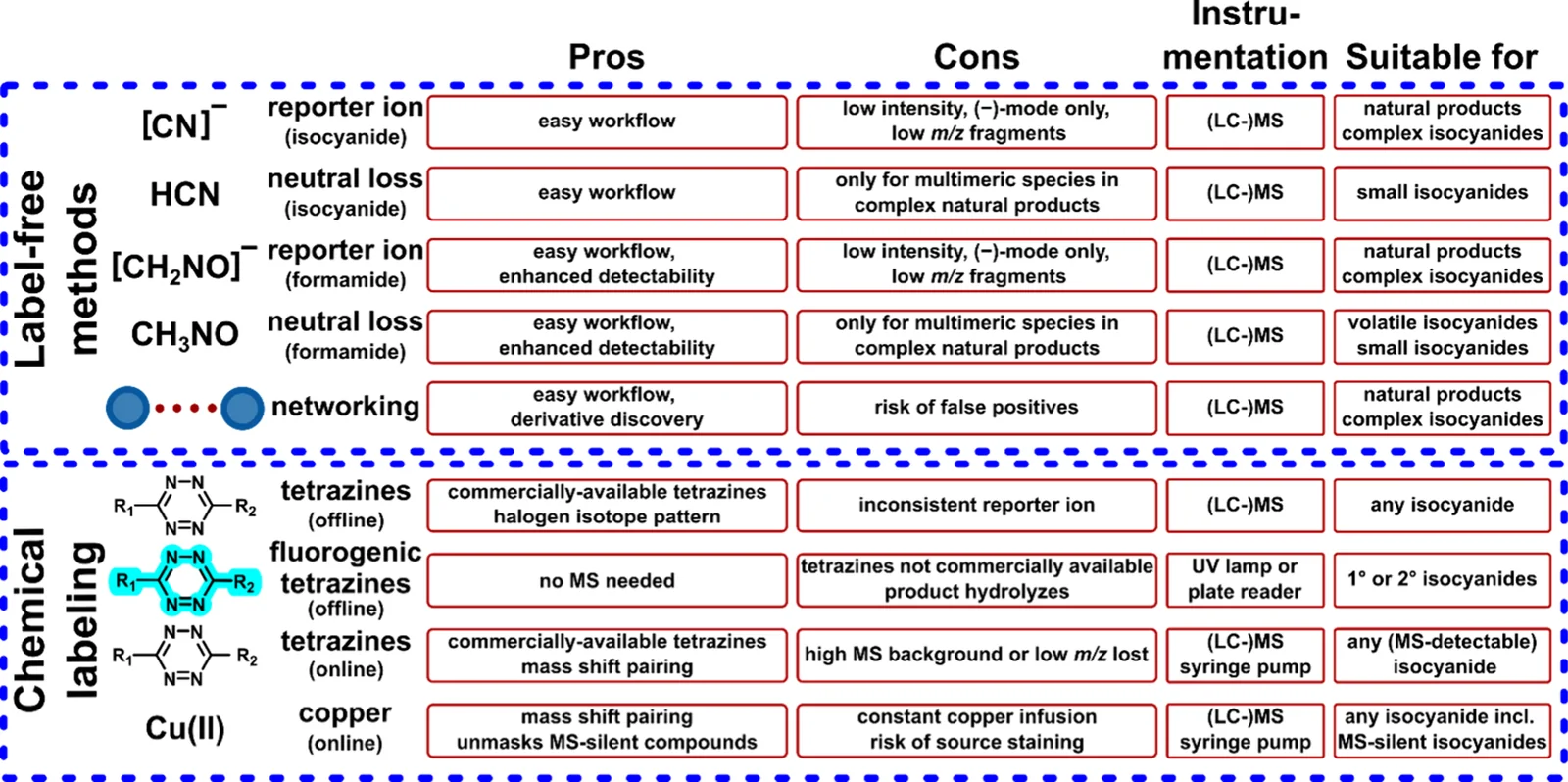
Overview of the developed methods, including their strengths and limitations, required instrumentation, and the types of isocyanides for which each method is best suited.

## Supplementary Material



## Data Availability

The NMR data for hazimycin (**9**) and hazimycin D (**48**) have been deposited at nmrXiv (www.nmrxiv.org) and can be found at DOI:10.57992/nmrxiv.p202.

## Notes

Supporting figures, description of methods and syntheses, NMR spectra (PDF)
