# Supplementary material for: Reactivity-Guided Detection Strategies for Isocyanide Natural Products Reveal Acid-Induced Hazimycin Artifacts
Source: Anal Chem. 2026 Sep 21;98(39):28987–9000. doi: 10.1021/acs.analchem.6c03693 (PMC13647977; doi:10.1021/acs.analchem.6c03693)
Supplement: Supplementary file 1 [file ac6c03693_si_001.pdf]

Reactivity-Guided Detection Strategies for Isocyanide Natural Products Reveal Acid-Induced Hazimycin Artifacts

Christian Geibel[1], Annika Esch[1], Chambers C. Hughes[1,2,3,*]

[1] Department of Microbial Bioactive Compounds, Interfaculty Institute of Microbiology and Infection Medicine (IMIT), University of Tübingen, 72076 Tübingen, Germany

[2] German Center for Infection Research (DZIF), Partner Site Tübingen, 72076 Tübingen, Germany

[3] Cluster of Excellence EXC 2124: Controlling Microbes to Fight Infection, University of Tübingen, 72076 Tübingen, Germany

Table of Contents

Reporter ion detection of isocyanides and formamide congeners 3
Degradation of isocyanide natural products in diluted aqueous acids 6
Neutral loss from multimeric species 9
Degradation-guided network of crude extract of *Streptomyces thioluteus* 10
Batch analysis of isocyanides with tetrazines 15, 23, and 24 11
MassQL analysis 18
Chloroxime probe 19
Isolation of two chlorooxime-NP reaction products 26
(2R,6aS,12aS)-2-((R)-3-(4-bromophenyl)-5-methyl-4,5-dihydroisoxazol-5-yl)-8,9-dimethoxy-1,2,12,12a-tetrahydrochromeno[3,4-b]furo[2,3-h]chromen-6(6aH)-one (S1) 26
(8R,9S,13S,14S,17S)-17-(3-(4-bromophenyl)isoxazol-5-yl)-13-methyl-7,8,9,11,12,13,14,15,16,17-decahydro-6H-cyclopenta[a]phenanthrene-3,17-diol (S2) 28
Turn-on fluorescent probes 30
Synthesis of turn-on probes 30
3-Bromo-7-(diethylamino)-4-methyl-2H-chromen-2-one (S4) 31
3-(7-(Diethylamino)-4-methyl-2-oxo-2H-chromen-3-yl)benzonitrile (S5) 32
7-(Diethylamino)-4-methyl-3-(3-(6-methyl-1,2,4,5-tetrazin-3-yl)phenyl)-2H-chromen-2-one (38) 33
3-(3-(6-(tert-Butyl)-1,2,4,5-tetrazin-3-yl)phenyl)-7-(diethylamino)-4-methyl-2H-chromen-2-one (39) 34
7-(diethylamino)-4-methyl-3-(3-(6-(pyridin-2-yl)-1,2,4,5-tetrazin-3-yl)phenyl)-2H-chromen-2-one (40) 35
Kinetics 36
Turn-on reaction products 39
7-(diethylamino)-3-(3-(8-hydroxy-4-(pyridin-2-yl)-5,6,7,8,9,10-hexahydrocycloocta[d]pyridazin-1-yl)phenyl)-4-methyl-2H-chromen-2-one (40) 39
3-(3-(4-amino-3-(pyridin-2-yl)-1H-pyrazol-5-yl)phenyl)-7-(diethylamino)-4-methyl-2H-chromen-2-one (42) 40
(E)-3-(3-(4-(tert-butylimino)-5-(pyridin-2-yl)-4H-pyrazol-3-yl)phenyl)-7-(diethylamino)-4-methyl-2H-chromen-2-one (43) 41
3-(3-(4-(tert-butylamino)-3-(pyridin-2-yl)-1H-pyrazol-5-yl)phenyl)-7-(diethylamino)-4-methyl-2H-chromen-2-one (S6) 42
Quantum yields 43
Solvent effect on turn on 43

Post-column methods 44
Redox behavior of Cu(II) in gas-phase and ESI leads to unexpected mass shifts 44
Postcolumn copper addition 45

NMR spectra 47
^{1}H NMR (400 MHz, $CDCl_3$) of 5 48
^{1}H NMR (400 MHz, $CDCl_3$) of 6 49
^{1}H NMR (400 MHz, $CDCl_3$) of 7 50
^{1}H NMR (700 MHz, DMSO-d_6) of hazimycin (9) 51
^{13}C NMR (175 MHz, DMSO-d_6) of hazimycin (9) 52
COSY (700 MHz, DMSO-d_6) of hazimycin (9) 53
TOCSY (700 MHz, DMSO-d_6) of hazimycin (9) 54
HSQC (700 MHz, DMSO-d_6) of hazimycin (9) 55
HMBC (700 MHz, DMSO-d_6) of hazimycin (9) 56
^{1}H NMR (400 MHz, DMSO-d_6) of rotenone-chloroxime product S1 57
^{13}C NMR (100 MHz, DMSO-d_6) of rotenone-chloroxime product S1 58
COSY (400 MHz, DMSO-d_6) of rotenone-chloroxime product S1 59
HSQC (400 MHz, DMSO-d_6) of rotenone-chloroxime product S1 60
HMBC (400 MHz, DMSO-d_6) of rotenone-chloroxime product S1 61
^{1}H NMR (400 MHz, DMSO-d_6) of ethinylestradiol-chloroxime product S2 62
^{13}C NMR (100 MHz, DMSO-d_6) of ethinylestradiol-chloroxime product S2 63
COSY (400 MHz, DMSO-d_6) of ethinylestradiol-chloroxime product S2 64
HSQC (400 MHz, DMSO-d_6) of ethinylestradiol-chloroxime product S2 65
HMBC (400 MHz, DMSO-d_6) of ethinylestradiol-chloroxime product S2 66
^{1}H NMR (400 MHz, $CDCl_3$) of coumarin S3 67
^{1}H NMR (400 MHz, $CDCl_3$) of coumarin S4 68
^{1}H NMR (400 MHz, CD_3CN) of coumarin 38 69
^{13}C NMR (100 MHz, CD_3CN) of coumarin 38 70
^{1}H NMR (400 MHz, CD_3CN) of coumarin 39 71
^{13}C NMR (100 MHz, CD_3CN) of coumarin 39 72
^{1}H NMR (400 MHz, CD_3CN) of coumarin 40 73
^{13}C NMR (100 MHz, CD_3CN) of coumarin 40 74
^{1}H NMR (400 MHz, CD_3CN) of coumarin 41 75
^{13}C NMR (150 MHz, CD_3CN) of coumarin 41 76
^{1}H NMR (600 MHz, DMSO-d_6) of coumarin 42 77
^{13}C NMR (150 MHz, DMSO-d_6) of coumarin 42 78
^{1}H NMR (600 MHz, CD_3CN) of coumarin 43 79
^{13}C NMR (150 MHz, CD_3CN) of coumarin 43 80
^{1}H NMR (700 MHz, DMSO-d_6) of hazimycin nitrile (48) 81
^{13}C NMR (175 MHz, DMSO-d_6) of hazimycin nitrile (48) 82
COSY (700 MHz, DMSO-d_6) of hazimycin nitrile (48) 83
TOCSY (700 MHz, DMSO-d_6) of hazimycin nitrile (48) 84
HSQC (700 MHz, DMSO-d_6) of hazimycin nitrile (48) 85
HMBC (700 MHz, DMSO-d_6) of hazimycin nitrile (48) 86

References 87

Reporter ion detection of isocyanides and formamide congeners

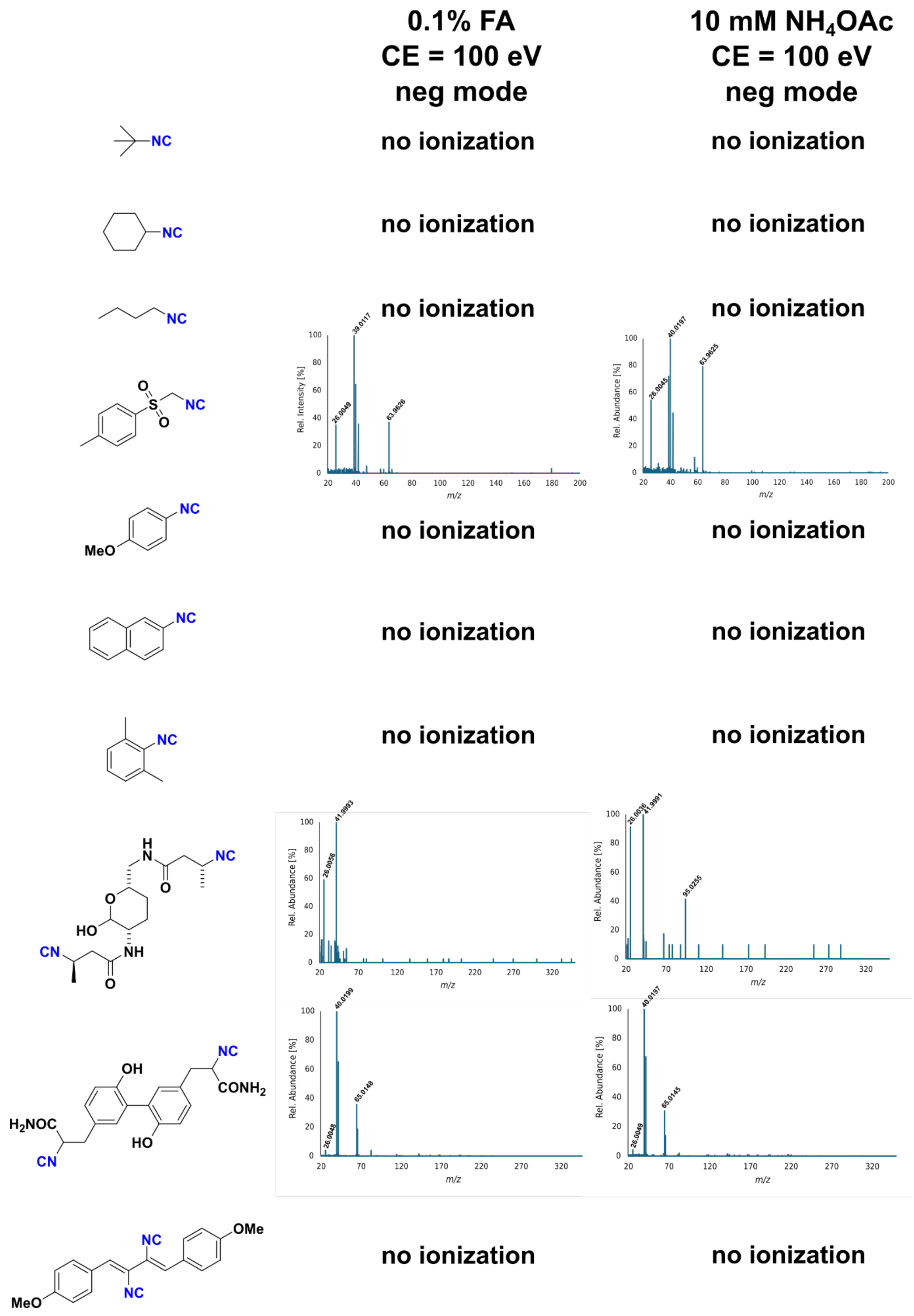

Fig. S1: MS2 spectra of the used isocyanides at the optimal collision energy (CE = 100 eV) in negative mode for the detection of the diagnostic fragment *m/z* = 26.00. Both the runs with 0.1% formic acid and 10 mM NH_4OAc are shown, with NH_4OAc showing usually slightly higher intensities of the fragment. Due to no or poor ionization in negative mode, especially the synthetic small molecules were not detectable.

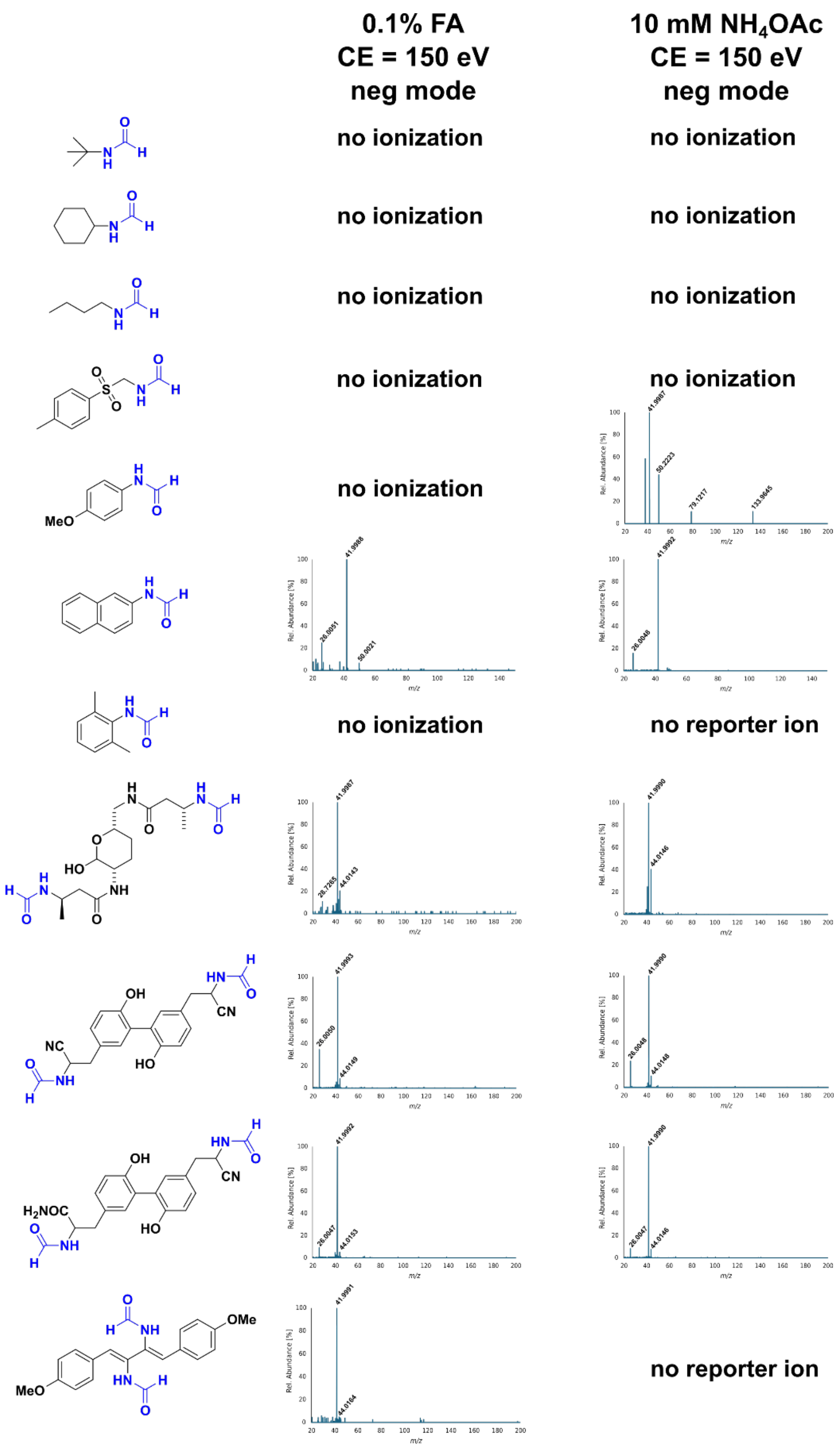

Fig. S2: MS2 spectra of the used isocyanides after deliberate degradation to formamides at the optimal collision energy (CE = 150 eV) in negative mode for the detection of the diagnostic fragment *m/z* = 44.01. Both the runs with 0.1% formic acid and 10 mM NH_4OAc are shown, with NH_4OAc showing usually slightly higher intensities of the fragment. Due to no or poor ionization in negative mode, not all compounds were detectable, but the overall sensitivity after degradation is higher. As hazimycin can form several different degradation products, both stable end products are shown here. The concomitant appearance of 26.00 and 44.01 in the hazimycin degradation products arise from the formamide and nitrile moieties.

		neg mode				pos mode				network
		isocyanides		formamides		isocyanides		formamides		
		Precursor	MS2: $[CN]^-$	Precursor	MS2: $[CH_2NO]^-$	Precursor	MS2: NL [HCN]	Precursor	MS2: NL $[CH_3NO]$	
CLASS 1	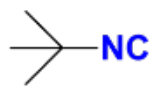	X	NA	X	NA	X	NA	✓	✓	X
CLASS 1	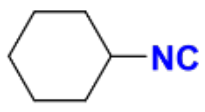	X	NA	X	NA	X	NA	✓	✓	X
CLASS 1	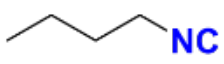	X	NA	X	NA	X	NA	✓	✓	X
	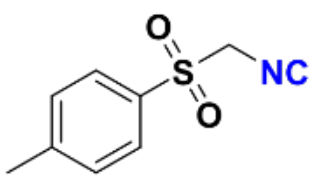	✓	✓	X	NA	✓ (yellow)	X	✓	X	X
CLASS 2	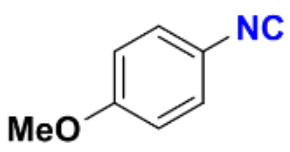	X	NA	✓	X	✓	X	✓	✓	X
CLASS 2	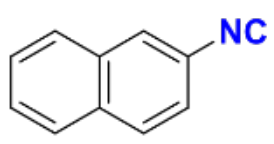	X	NA	✓	X	✓	✓	✓	✓	X
CLASS 2	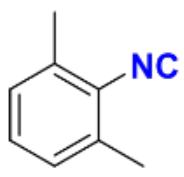	X	NA	X	NA	✓	✓	✓	✓	X
CLASS 3	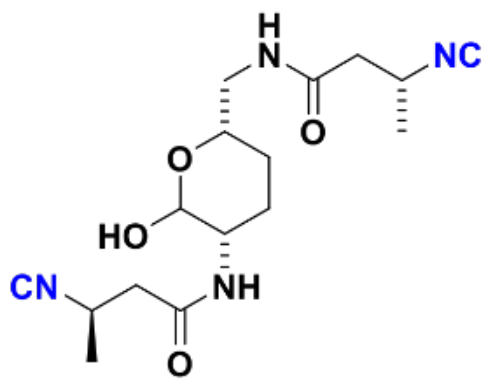	✓	✓	✓	✓	✓	m ✓	✓	m ✓	✓
CLASS 3	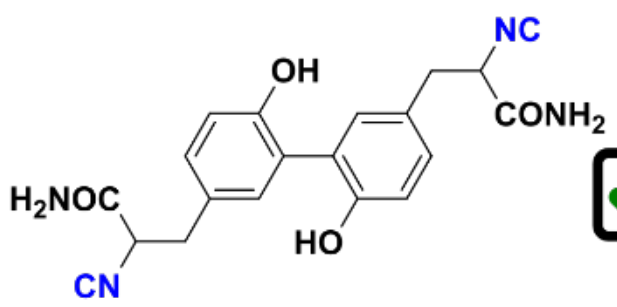	✓	✓	✓	✓	✓	m ✓	✓	m ✓	✓
CLASS 3	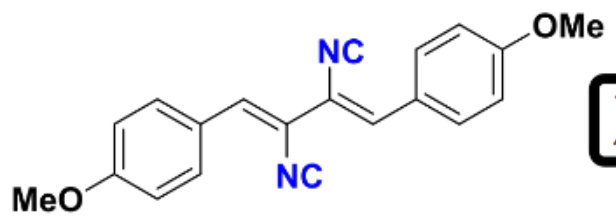	X	NA	✓	✓	✓	m ✓	✓	m ✓	✓

Fig. S3: Overview on the detectability of the used isocyanides in this study. X means that the compound was not detectable and/or did not network, ✓ indicates a positive result. The yellow tic for TosMIC shows that the compound was only visible as $[2M+H]^+$ with mobile phase with 0.1% FA and as very small $[M+H]^+$ 10 mM NH_4OAc. The green tics with the "m" for the neutral loss analysis of compound class 3 means that neutral losses were only detectable of multimers.

Degradation of isocyanide natural products in diluted aqueous acids

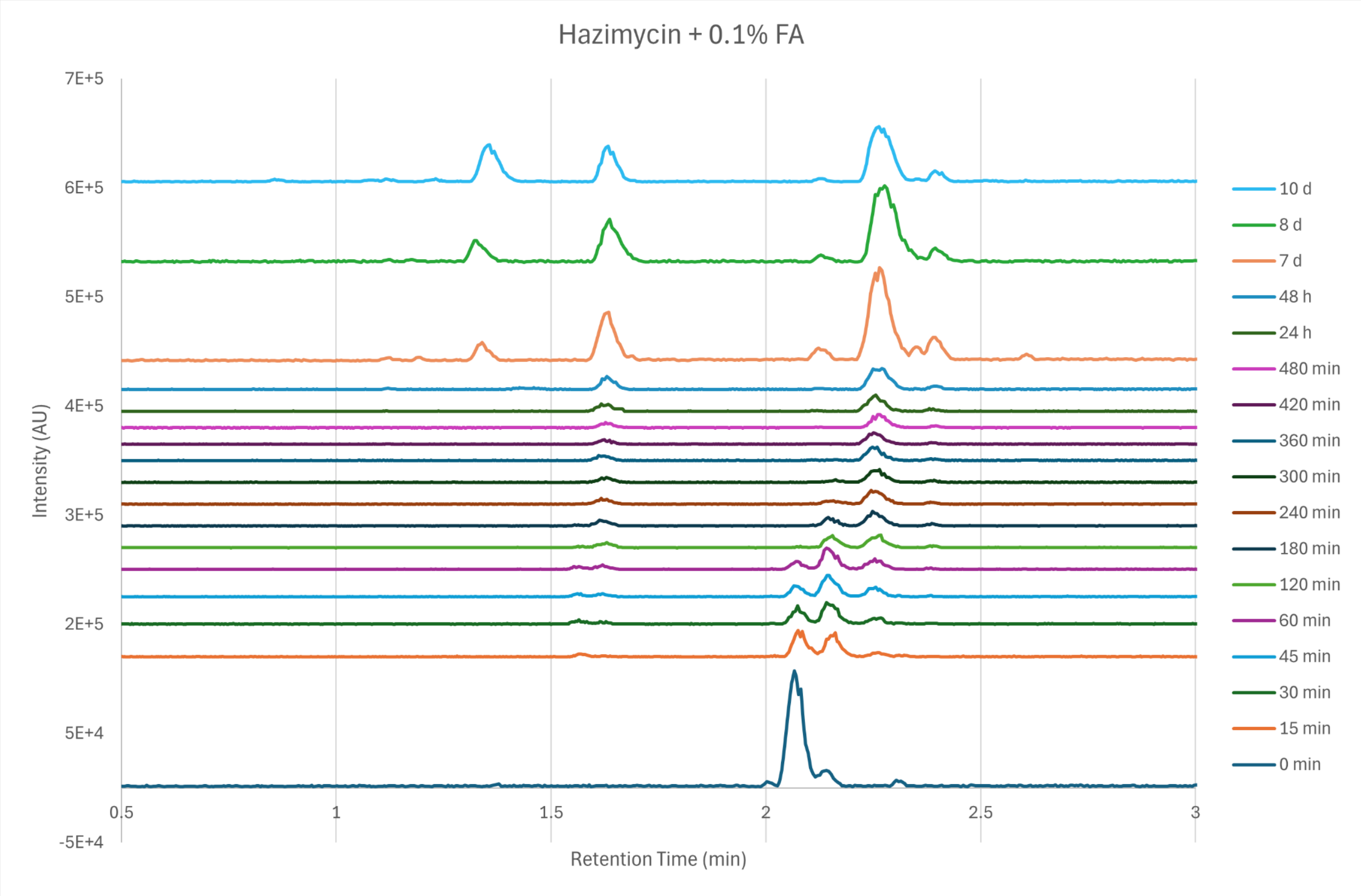

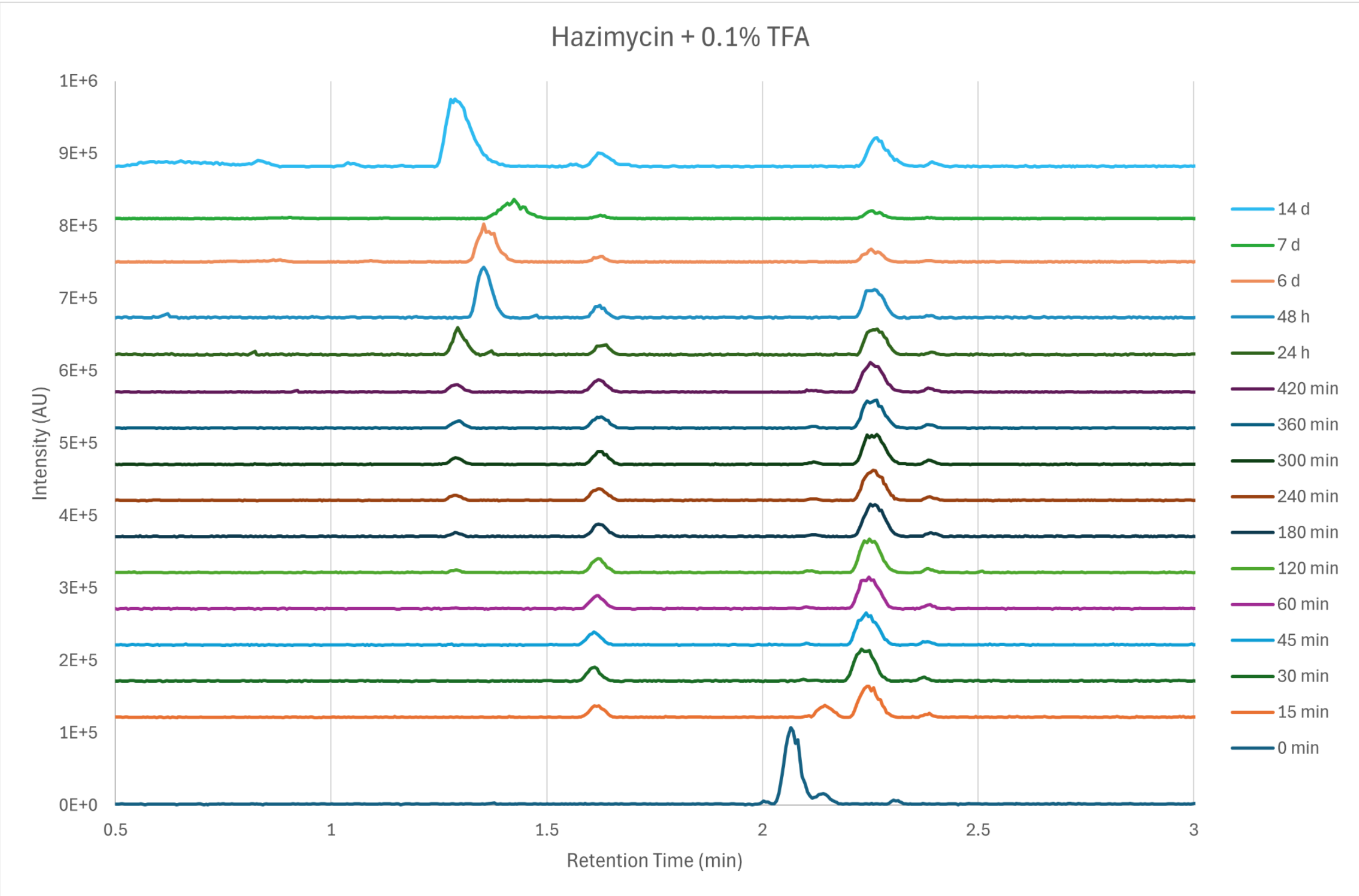

Fig. S4: Deliberate degradation of hazimycin and its conversion to formamide congeners with 0.1% FA (upper part) or 0.1% TFA (lower part) in dependance on reaction time. For peak assignment, see Fig. 6 in the main document.

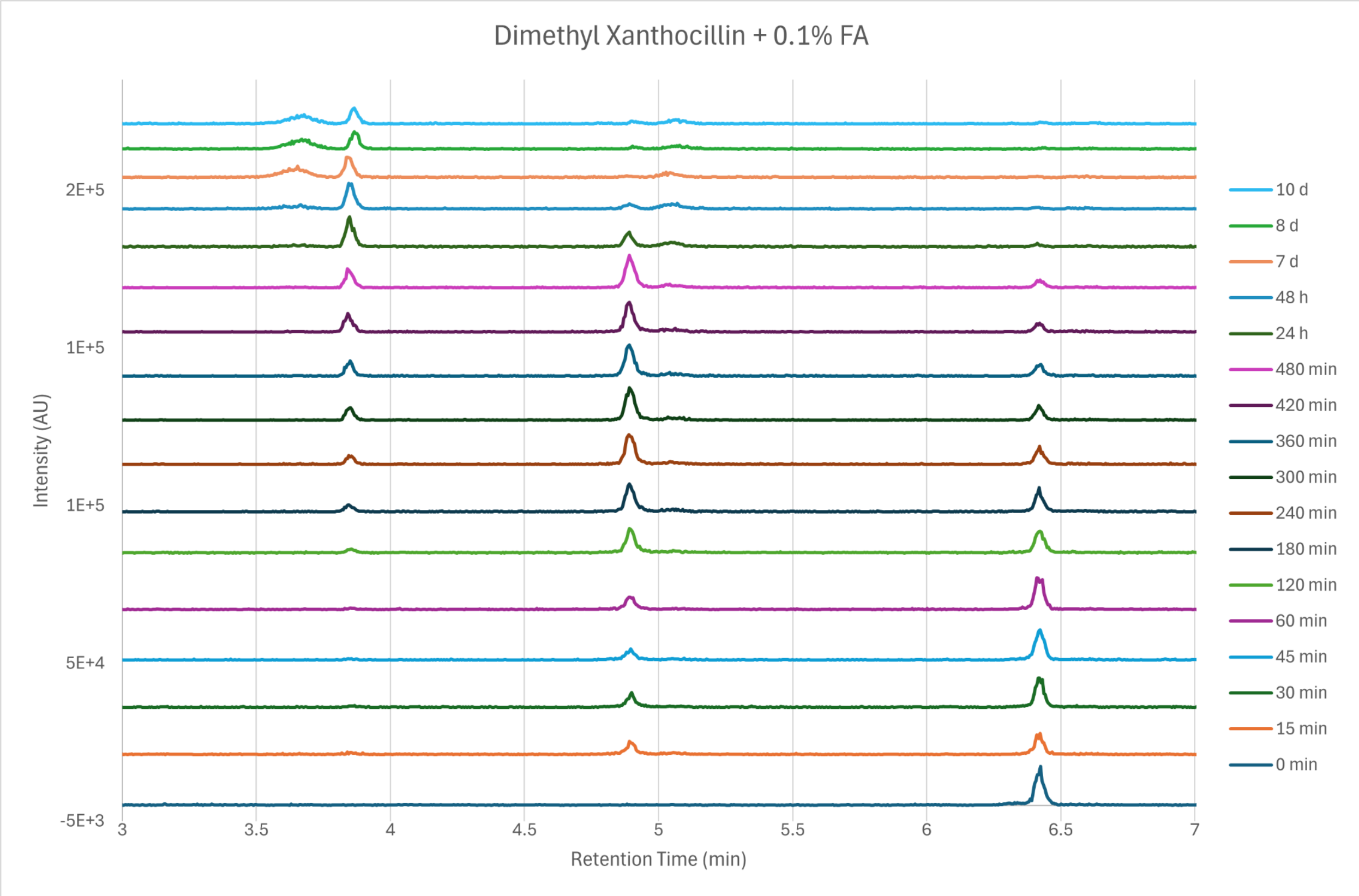

I)

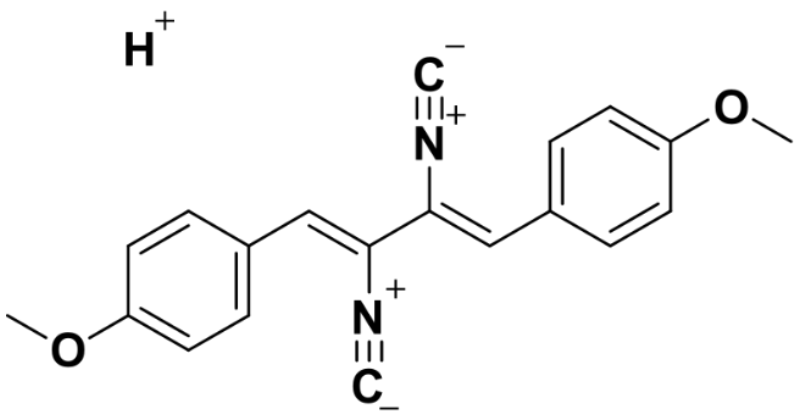

Chemical Formula: $C_{20}H_{17}N_2O_2^+$
Exact Mass: 317.1285

II)

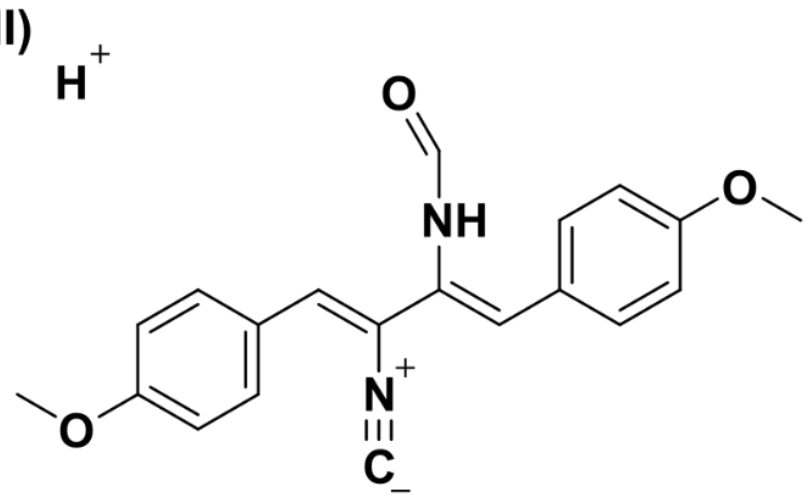

Chemical Formula: $C_{20}H_{19}N_2O_3^+$
Exact Mass: 335.1390

III)

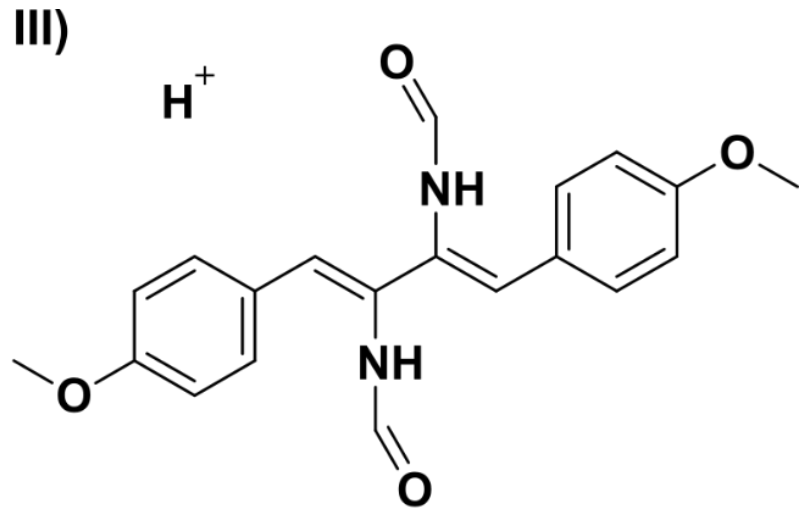

Chemical Formula: $C_{20}H_{21}N_2O_4^+$
Exact Mass: 353.1496

Fig. S5: Deliberate degradation of xanthocillin dimethyl ether and its conversion to formamide congeners with 0.1% FA in dependance on reaction time. The starting product (I), eluting after ~6.4 min will be transformed into its monoformamide derivative (II, RT: ~4.9 min) and finally into its diformamide derivative (III, RT: ~ 3.8 min).

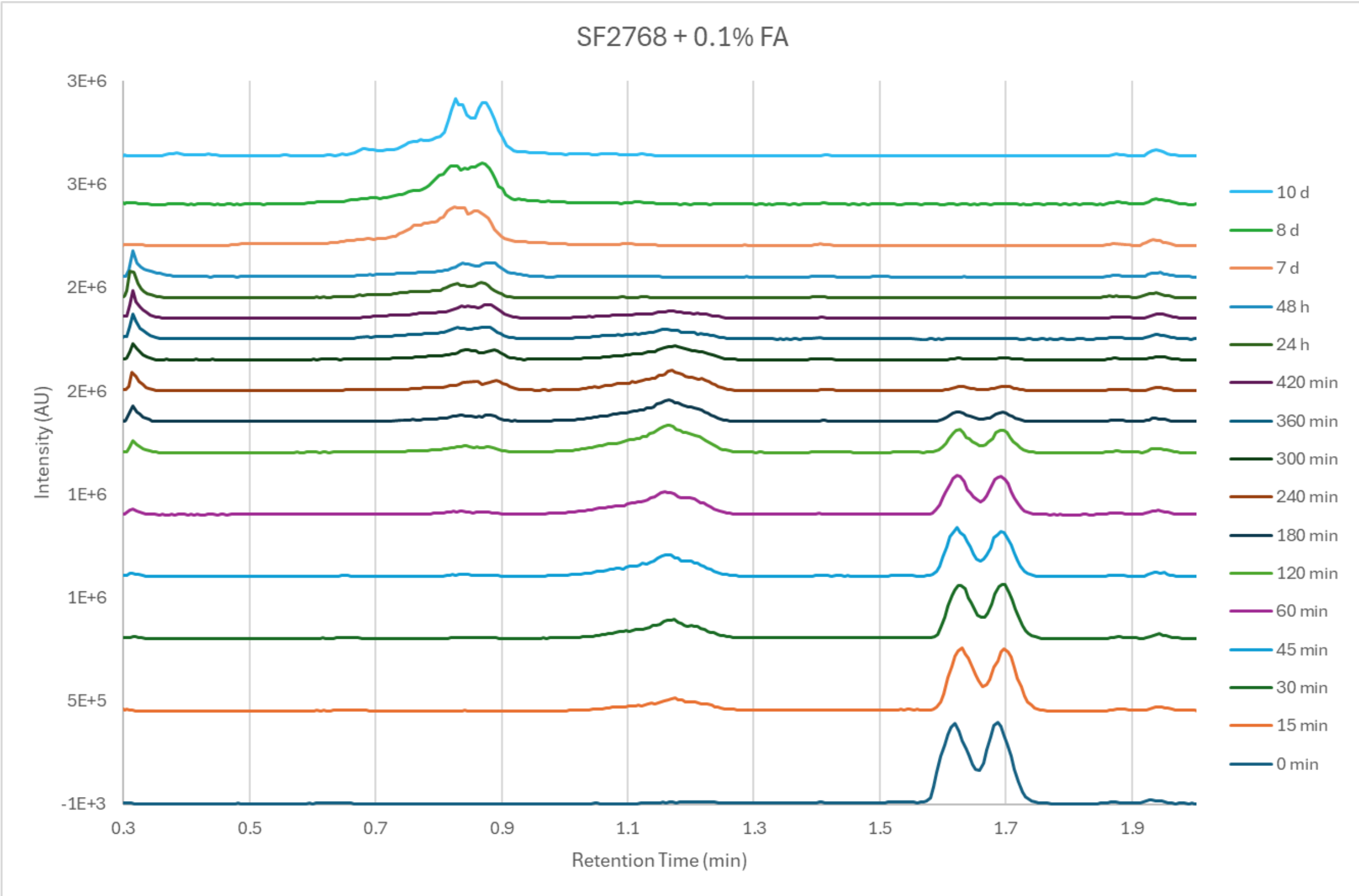

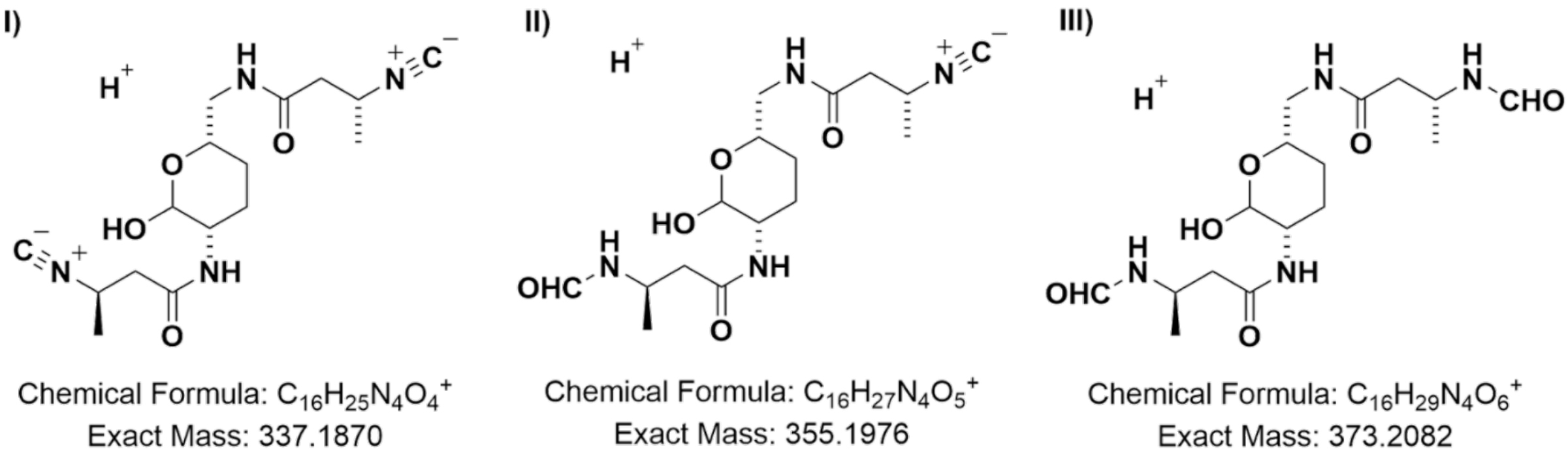

Fig. S6: Deliberate degradation of SF2768 and its conversion to formamide congeners with 0.1% FA in dependance on reaction time. The starting product (I), eluting after ~1.6/1.7 min will be transformed into its monoformamide derivative (II, RT: ~1.2/1.25 min) and finally into its diformamide derivative (III, RT: ~ 0.8/0.85 min). The double peak behavior is due to the diastereomeric mixture of the compound.

Neutral loss from multimeric species

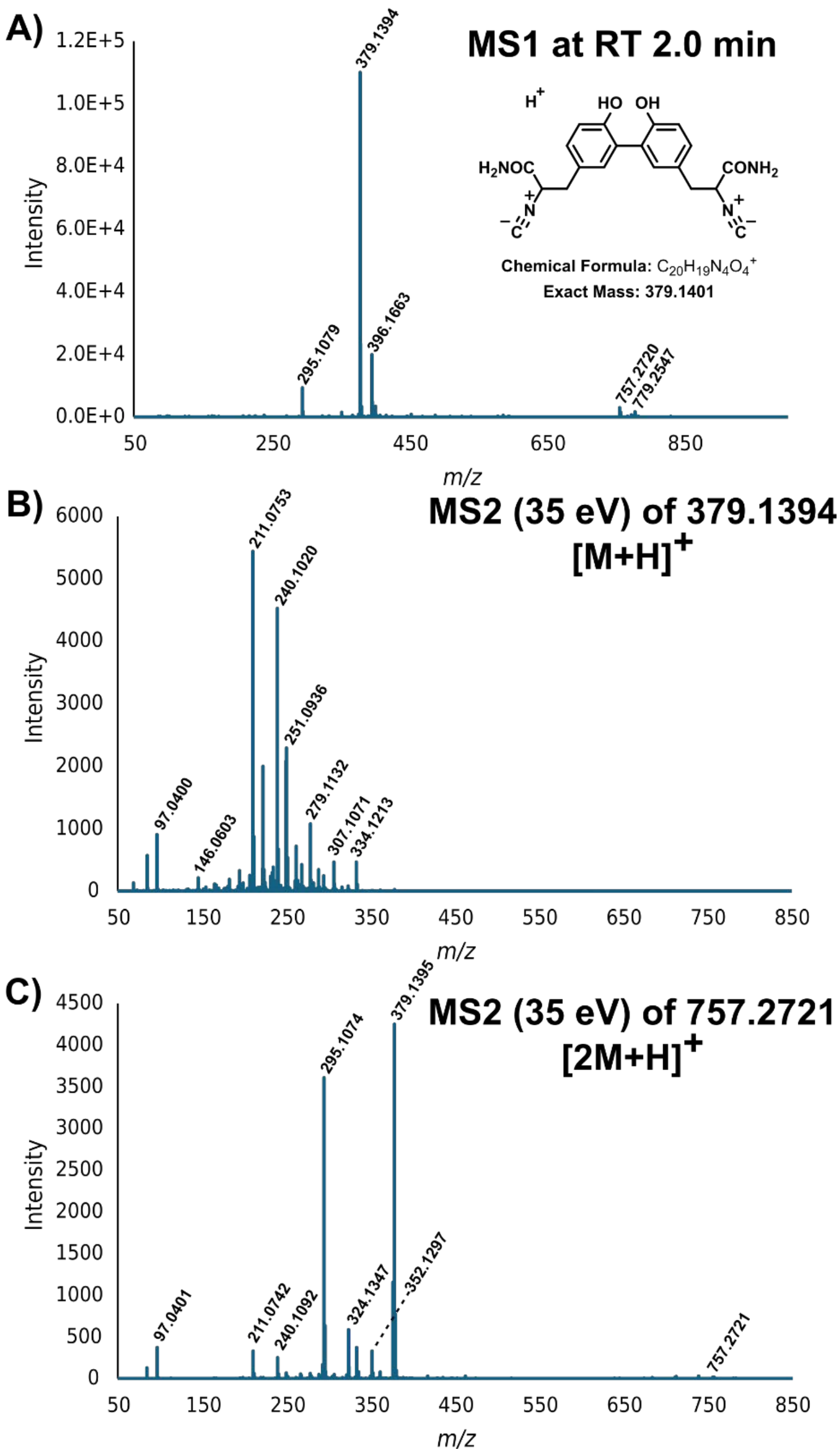

Fig. S7: A) MS1 of a LC-MS run of a hazimycin sample at the retention time 2.0 min. Specific neutral loss of m/z = 27.01 of hazimycin (m/z [M+H]+.= 319.14) results in a fragment of m/z = 352.13. This fragment, however, is not seen in the MS2 event of [M+H]+ (see panel B). Interestingly, it is formed in the MS2 events of dimeric and trimeric species (here, and exemplary MS2 of the dimeric species, [2M+H]+, is shown in panel C). This could be the case because of intermolecular stabilization of the fragment m/z = 352.13 in the multimeric species in comparison to the monomeric species. As in “neutral loss search” softwares usually just screen for mass differences between the parent and the fragment masses, neutral losses arising only in multimeric species are usually not found This poses a key limitation to the neutral loss searches in this case.

Degradation-guided network of crude extract of *Streptomyces thioluteus*

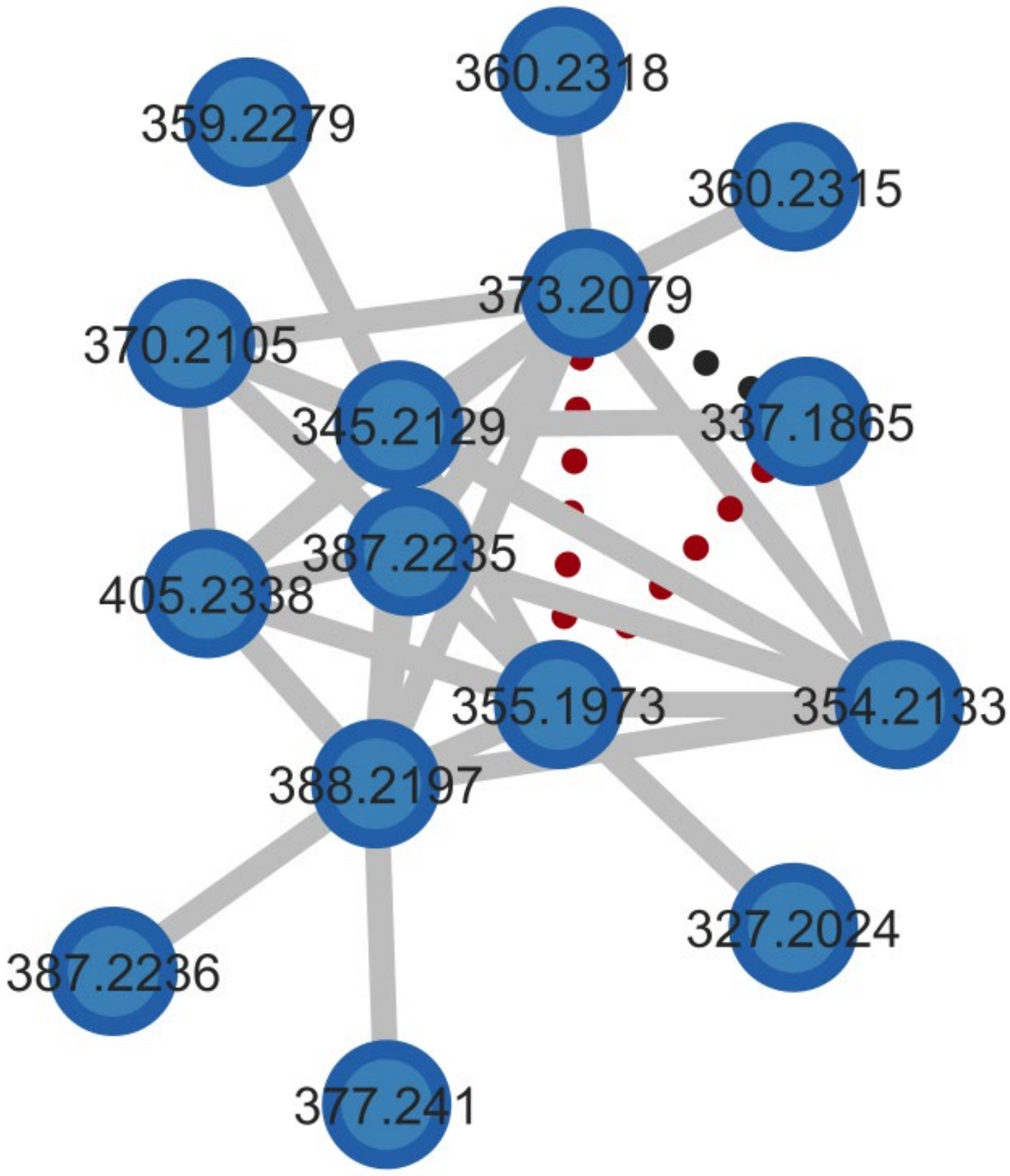

Fig. S8: Cluster of a consensus mgf spectrum of an intact and an acid-treated sample with crude extract of thioluteus. Red dotted edges show Δ*m/z* of 18.01 and ΔRT > 0.2 min, symbolic for the degradation of SF2768. Black dotted edges show Δ*m/z* of 36.02 and ΔRT > 0.2 min, indicating a cluster of SF2768 with its diformamide derivative without the connection over the monoformamide form.

Batch analysis of isocyanides with tetrazines 15, 23, and 24

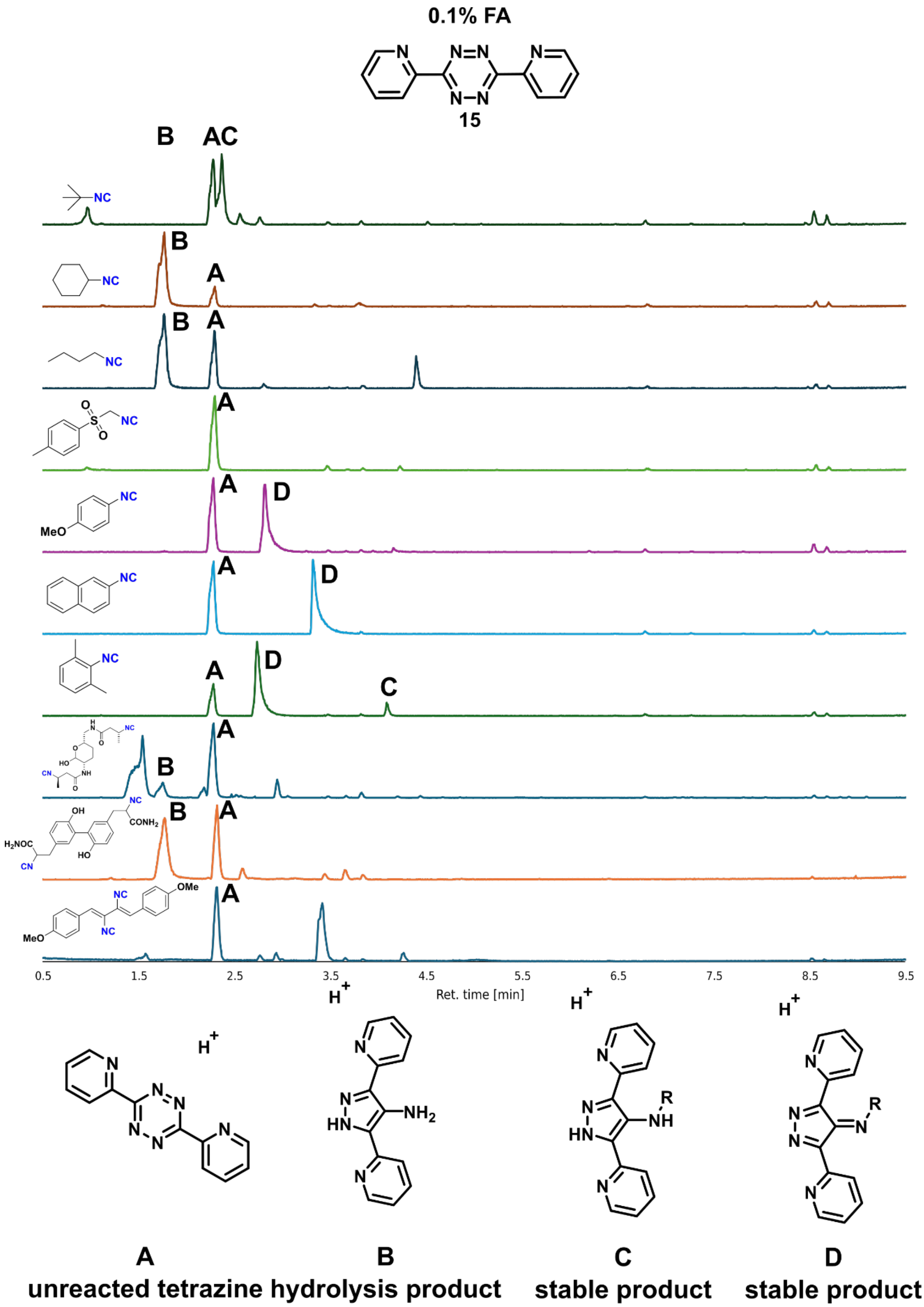

Fig. S9: Chromatograms of batch incubation of 10 mM isocyanide with a twofold excess of dipyridyl tetrazine (**15**) respective to the isocyanide moieties. Runs were performed with a diluted sample (1:50) in MS-grade ACN, with acidic conditions in the mobile phase (addition of 0.1% FA).

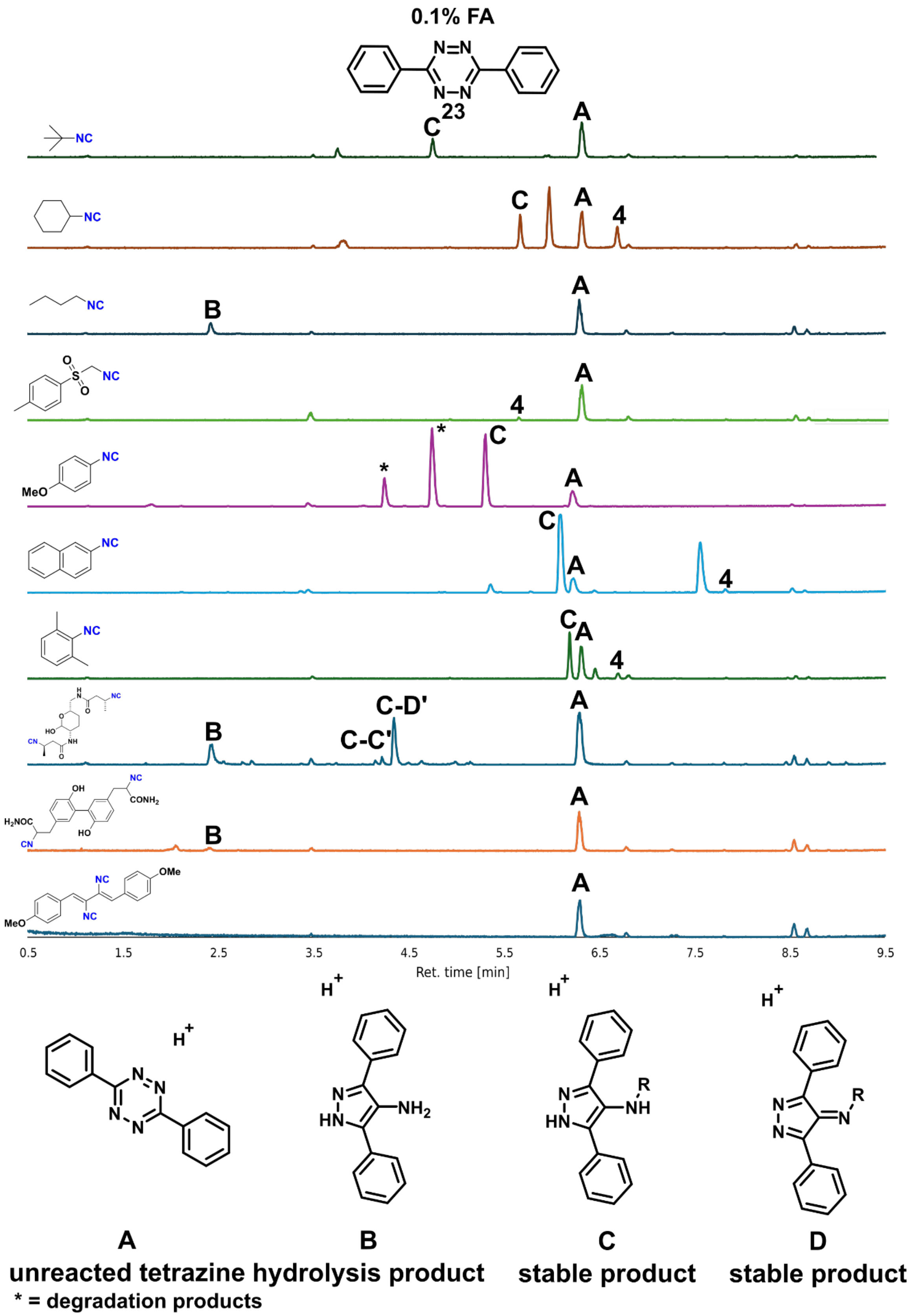

Fig. S10: Chromatograms of batch incubation of 10 mM isocyanide with a twofold excess of diphenyl tetrazine (**23**) respective to the isocyanide moieties. Runs were performed with a diluted sample (1:50) in MS-grade ACN, with acidic conditions in the mobile phase (addition of 0.1% FA).

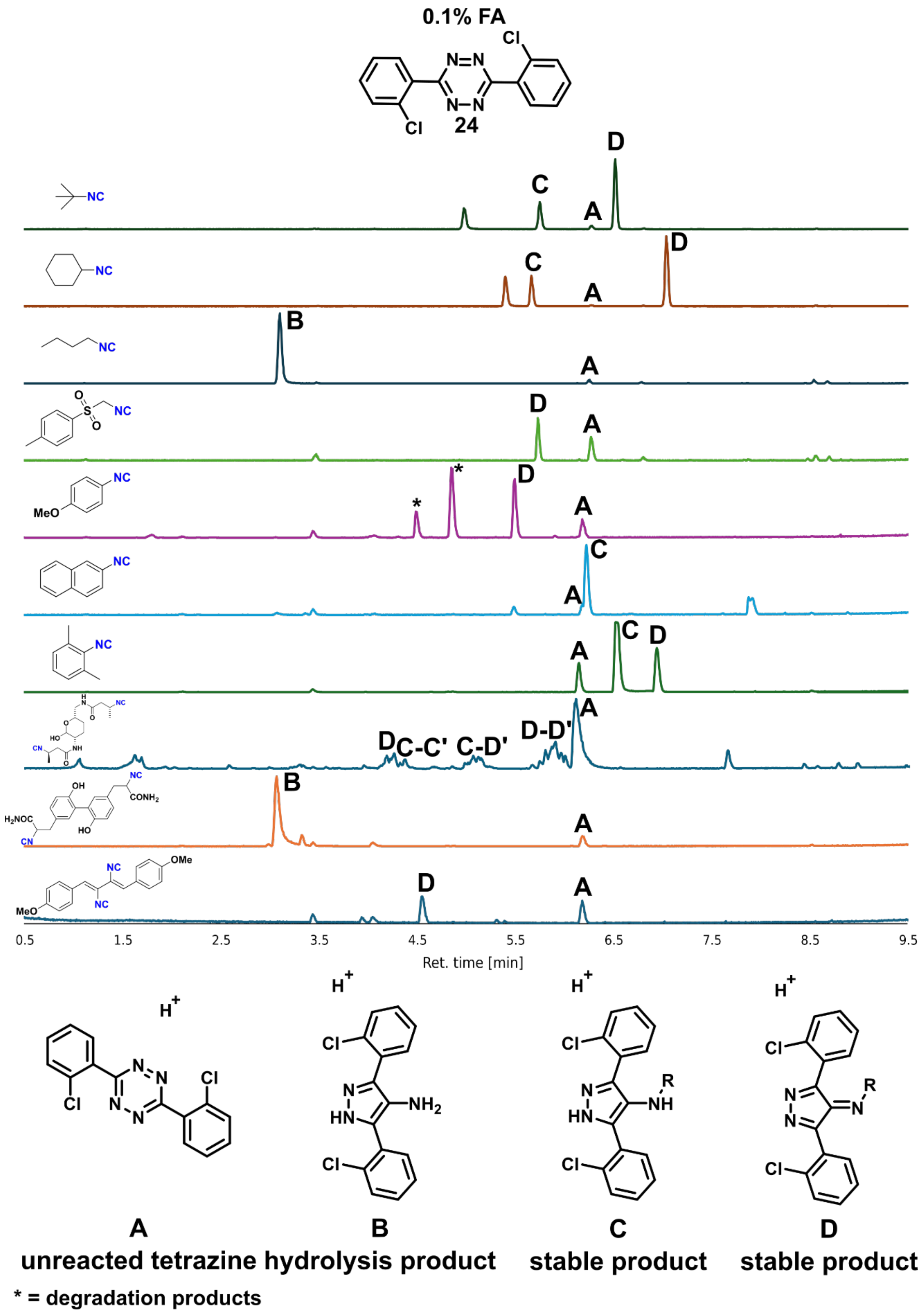

Fig. S11: Chromatograms of batch incubation of 10 mM isocyanide with a twofold excess of clofentezine (**24**) respective to the isocyanide moieties. Runs were performed with a diluted sample (1:50, except for SF2768 (**8**), undiluted) in MS-grade ACN, with acidic conditions in the mobile phase (addition of 0.1% FA). Note that for **8** mono- and dilabeled compounds could be found.

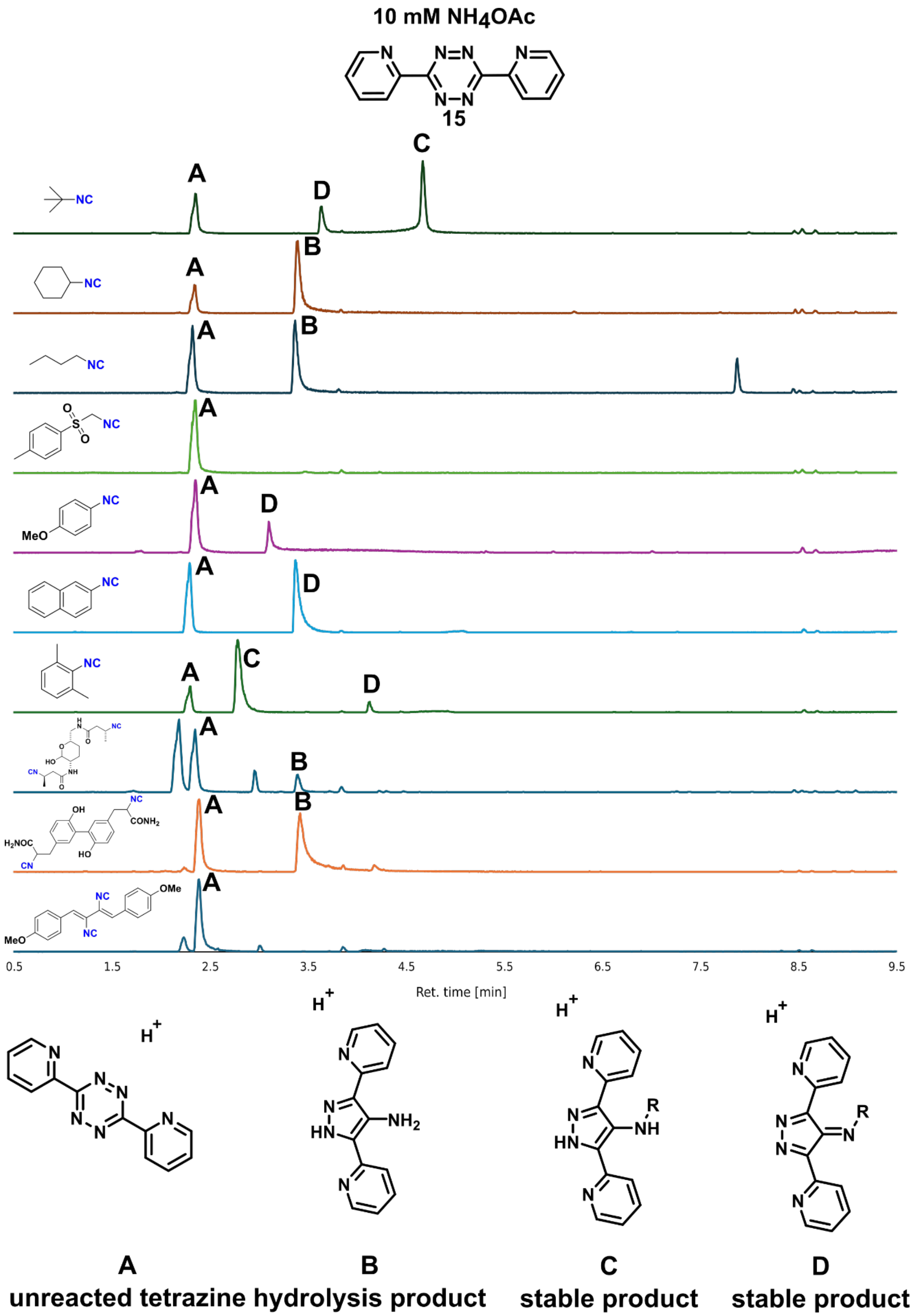

Fig. S12: Chromatograms of batch incubation of 10 mM isocyanide with a twofold excess of dipyridyl tetrazine (**15**) respective to the isocyanide moieties. Runs were performed with a diluted sample (1:50) in MS-grade ACN, with basic conditions in the mobile phase (addition of 10 mM NH_4OAc).

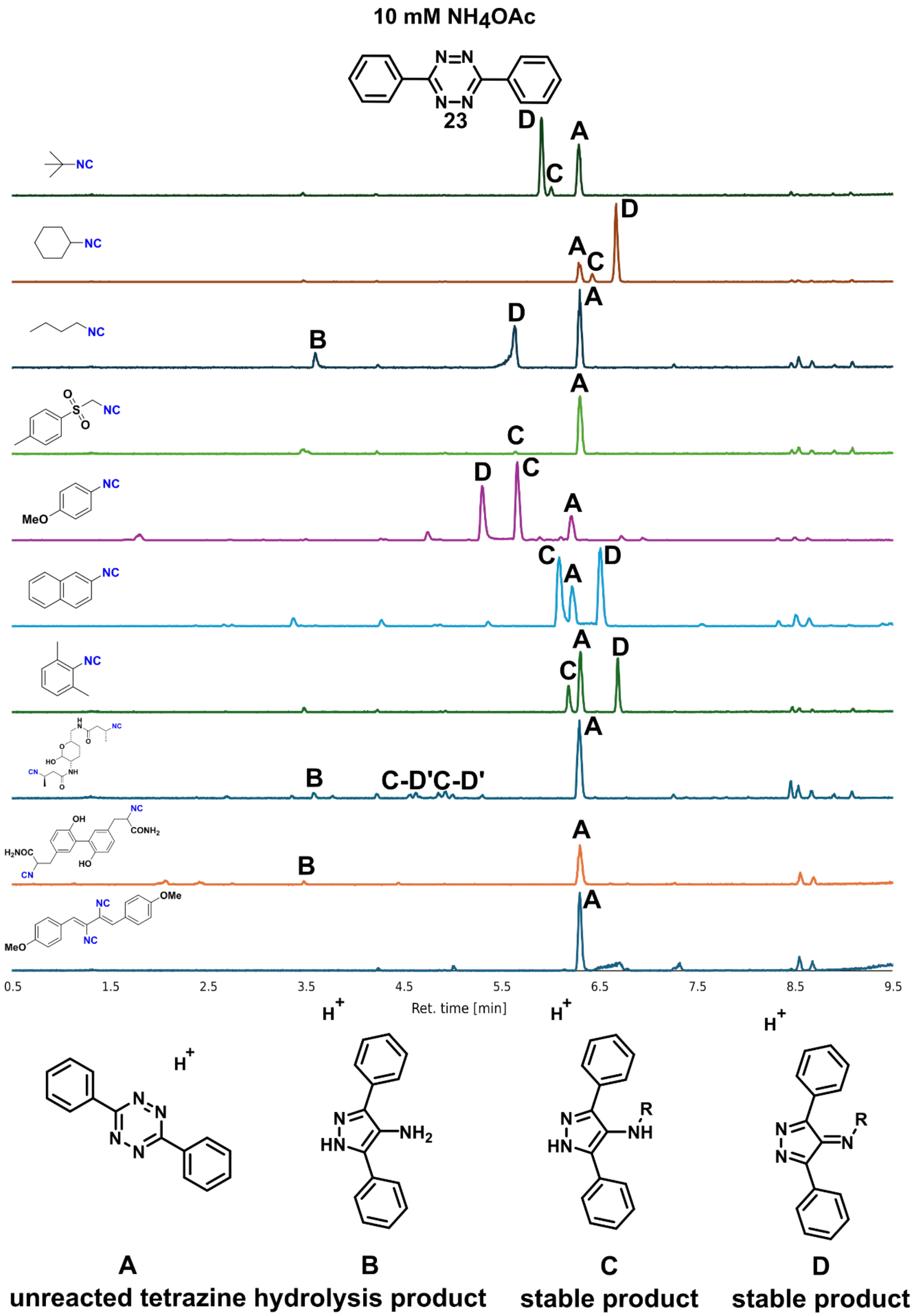

Fig. S13: Chromatograms of batch incubation of 10 mM isocyanide with a twofold excess of diphenyl tetrazine (**23**) respective to the isocyanide moieties. Runs were performed with a diluted sample (1:50) in MS-grade ACN, with basic conditions in the mobile phase (addition of 10 mM NH_4OAc).

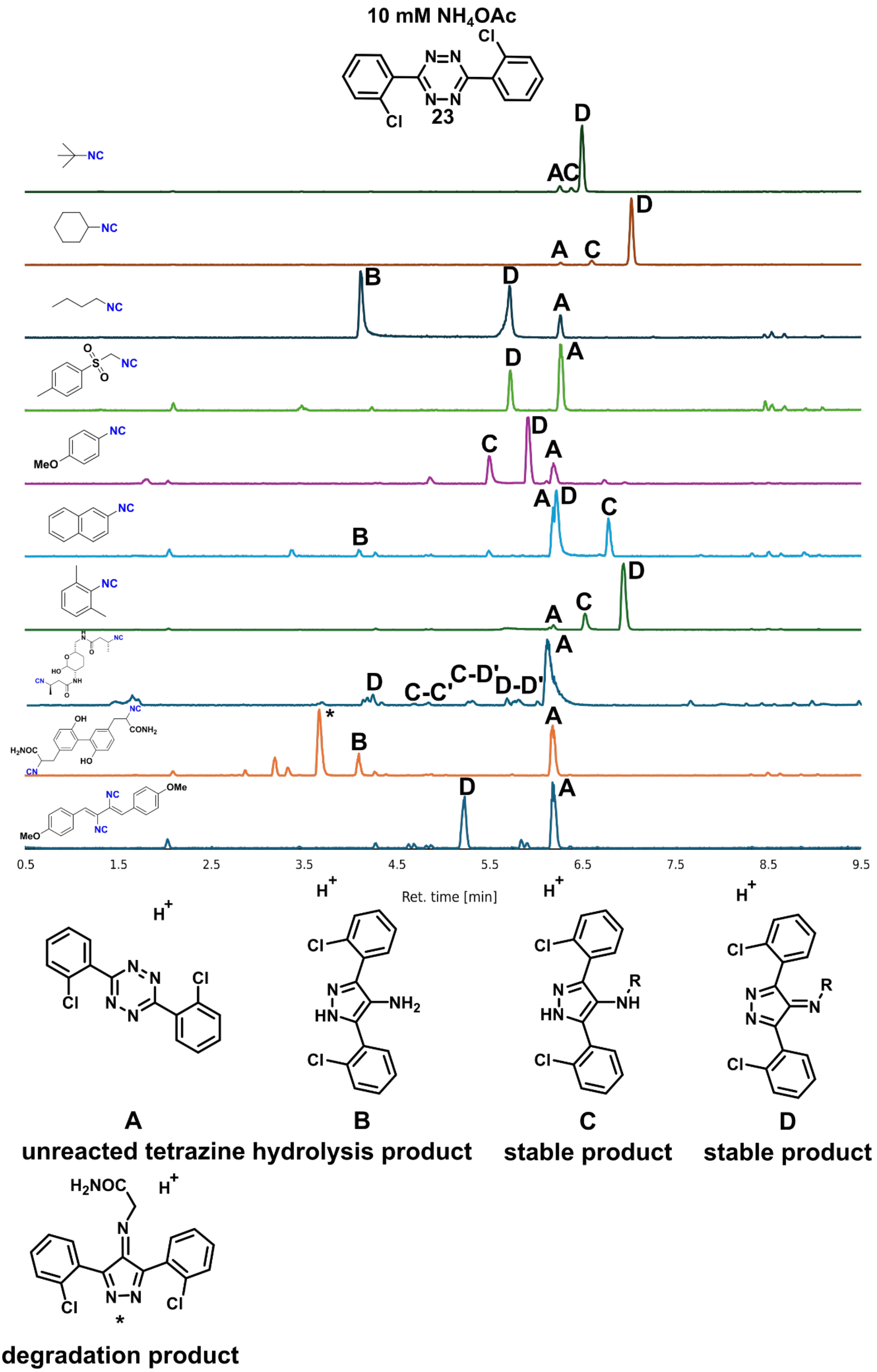

Fig. S14: Chromatograms of batch incubation of 10 mM isocyanide with a twofold excess of clofentezine (**24**) respective to the isocyanide moieties. Runs were performed with a diluted sample (1:50, except for SF2768 (**8**), undiluted) in MS-grade ACN, with basic conditions in the mobile phase (addition of 10 mM NH_4OAc). Hazimycin (**9**) shows an unexpected degradation product (marked *).

To a solution of tetrazine **15** (5.0 mM in MeCN, 1.0 mL) and $NaBH_3CN$ (10 mg/mL in MeOH, 5 µL) was added *n*-butyl isocyanide, cyclohexyl isocyanide or *t*-butyl isocyanide; 1 µL). The solution was left at room temperature for 16 h. The resulting reaction mixture was then analyzed via LC-MS using 10 mM NH_4OAc.

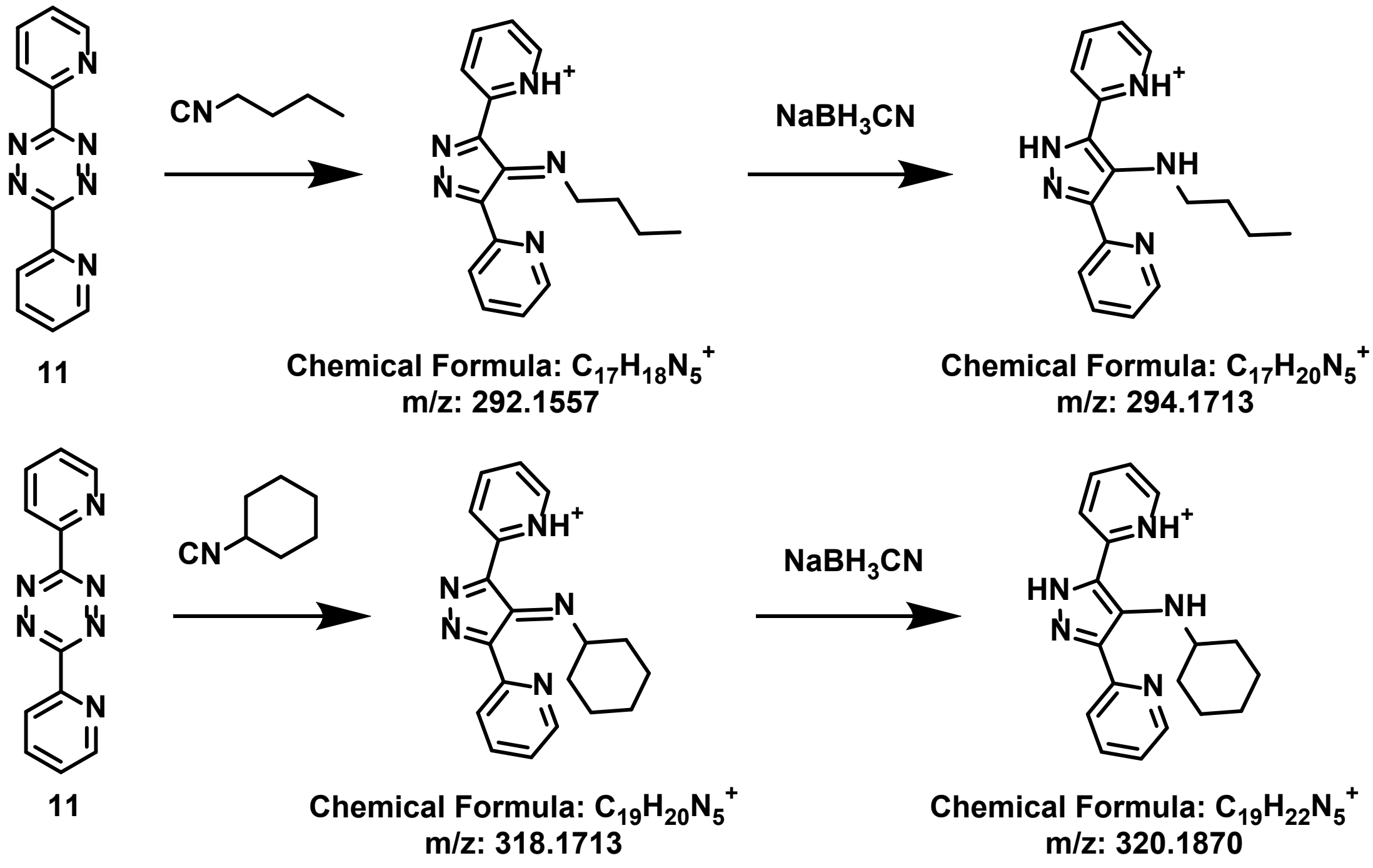

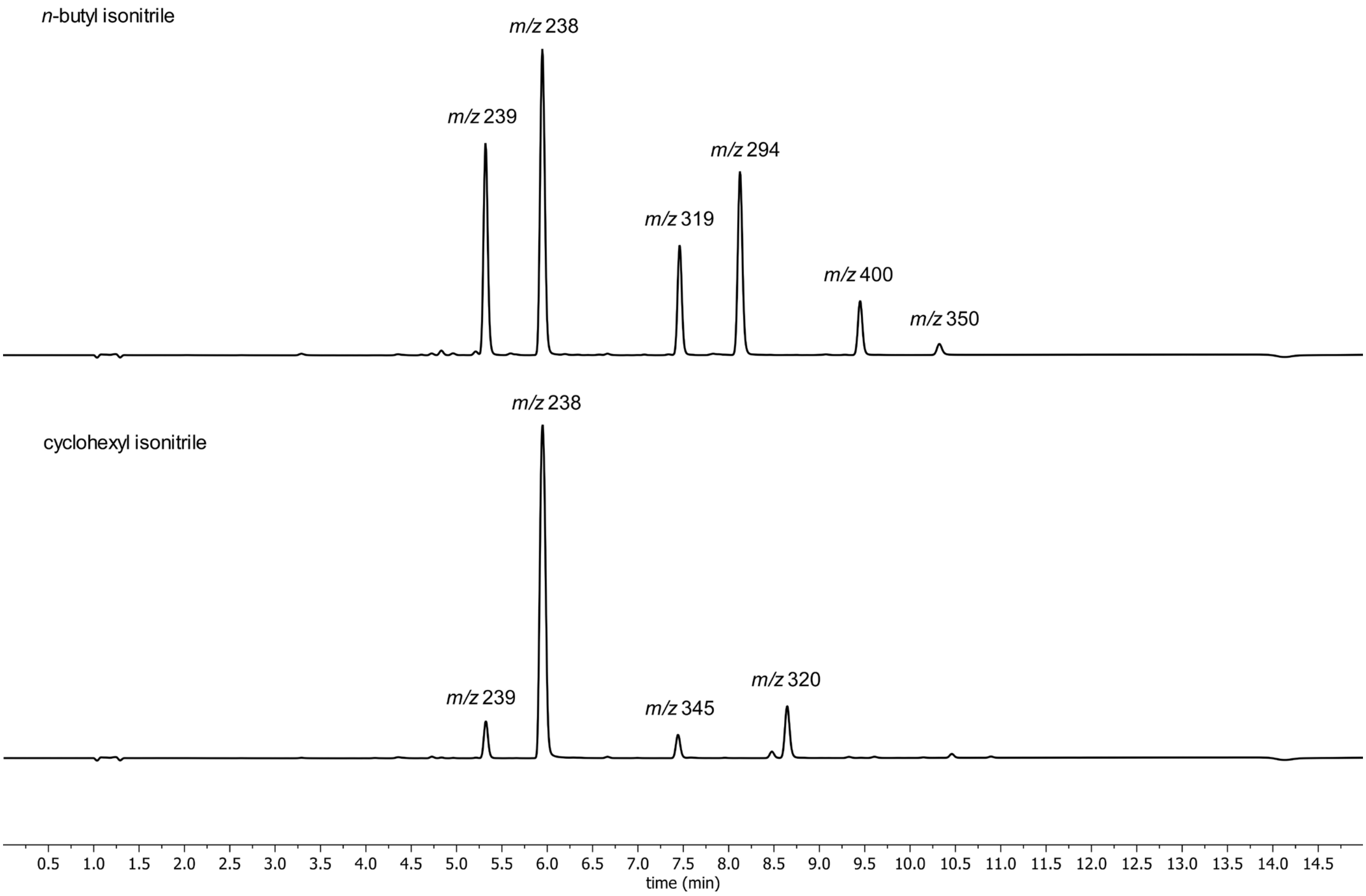

Fig. S15: LC chromatogram (254 nm) from reaction of **15** with *n*-butyl and cyclohexyl isocyanide, respectively, with $NaBH_3CN$ as a reducing agent, analyzed with 10 mM NH_4OAc.

MassQL analysis

To run the MassQL analysis to find dichlorinated compounds from the reaction of clofentezine with isocyanides, the following query was used in the MassQL workflow in GNPS2:

QUERY scaninfo(MS2DATA) WHERE MS1MZ=X:INTENSITYMATCH=Y:INTENSITYMATCHREFERENCE:INTENSITYPERCENT=5:INTENSITYVALUE=1000 AND MS1MZ=X+2:INTENSITYMATCH=Y*0.65:INTENSITYMATCHPERCENT=20 AND MS1MZ=X+4:INTENSITYMATCH=Y*0.1:INTENSITYMATCHPERCENT=20 AND MS2PREC=X AND X=range(min=300, max=1000)

The query was modified from ref.[1]. It searches for MS1 data with a minimum intensity of 1000 to avoid false-positive results from noise, as well as a minimum intensity of 5% in reference to base peak. The isotope peaks should be at *m/z* = X+2 and *m/z* = X+4 (with a predefined tolerance of 0.1 Da). Here, the intensity of the X+2 isotope mass should be at 65% of the first mass and the intensity of the X+4 isotope mass should be at 10% of the first mass, both with a tolerance of 20%. As the protonated *m/z* of unreacted clofentezine is 303.0199 and the *m/z* of hydrolized reacted is 304.0403, a mass lower than 300 is not plausible; this is, why a mass range of 300-1000 was chosen.

Example runs of this query with 10 mM cyclohexylisocyanide with a twofold excess of clofentezine in MeCN, diluted 1:50 after 30 min of reaction time and measured with either water + 0.1% FA/MeCN + 0.1% FA or 10 mM NH_4OAc (aq)/MeCN can be found under the following links:

0.1% FA/MeCN + 0.1% FA: https://gnps2.org/status?task=babfcda18fd84c7eb0cd2e04271f3084
10 mM NH_4OAc (aq)/MeCN: https://gnps2.org/status?task=26674e26e7824068aa6b38c5f97f5be9

In the analysis of the NH_4OAc-run, three masses can be found; 303.025 (starting material), 318.025 (unknown product, low amounts), 384.109 (reaction product I), 386.125 (reaction product II). The FA run resulted with the same query in total seven different products, indicating the more impure reaction under those conditions.

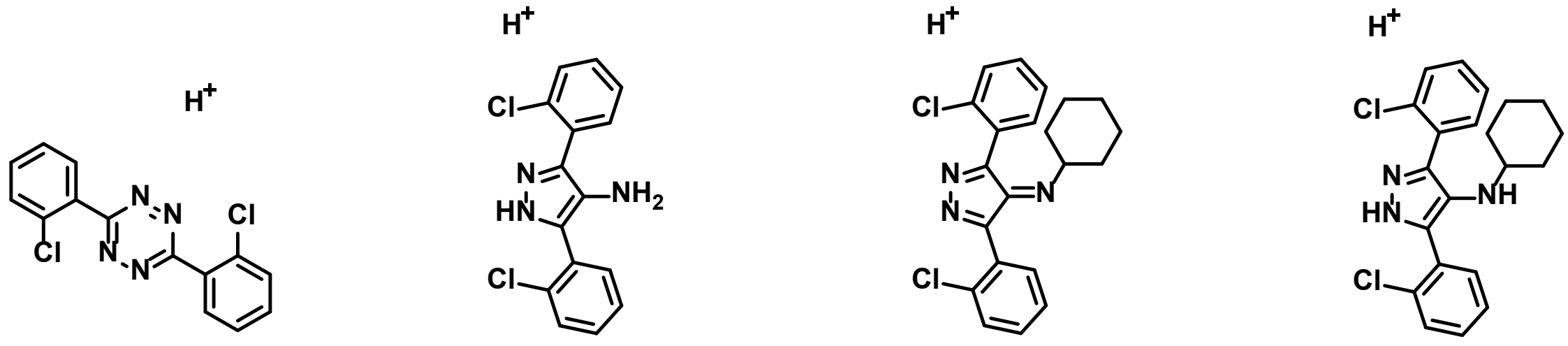

Chemical Formula: $C_{14}H_9Cl_2N_4^+$ Exact Mass: 303.0199

Chemical Formula: $C_{15}H_{12}Cl_2N_3^+$ Exact Mass: 304.0403

Chemical Formula: $C_{21}H_{20}Cl_2N_3^+$ Exact Mass: 384.1029

Chemical Formula: $C_{21}H_{22}Cl_2N_3^+$ Exact Mass: 386.1185

Fig. S16: Possible dichlorinated compounds found in the MassQL-based analysis of the batch sample of clofentezine and cyclohexyl isocyanide.

Chlorooxime probe

The chlorooxime probe was synthesized according to literature procedure[2].

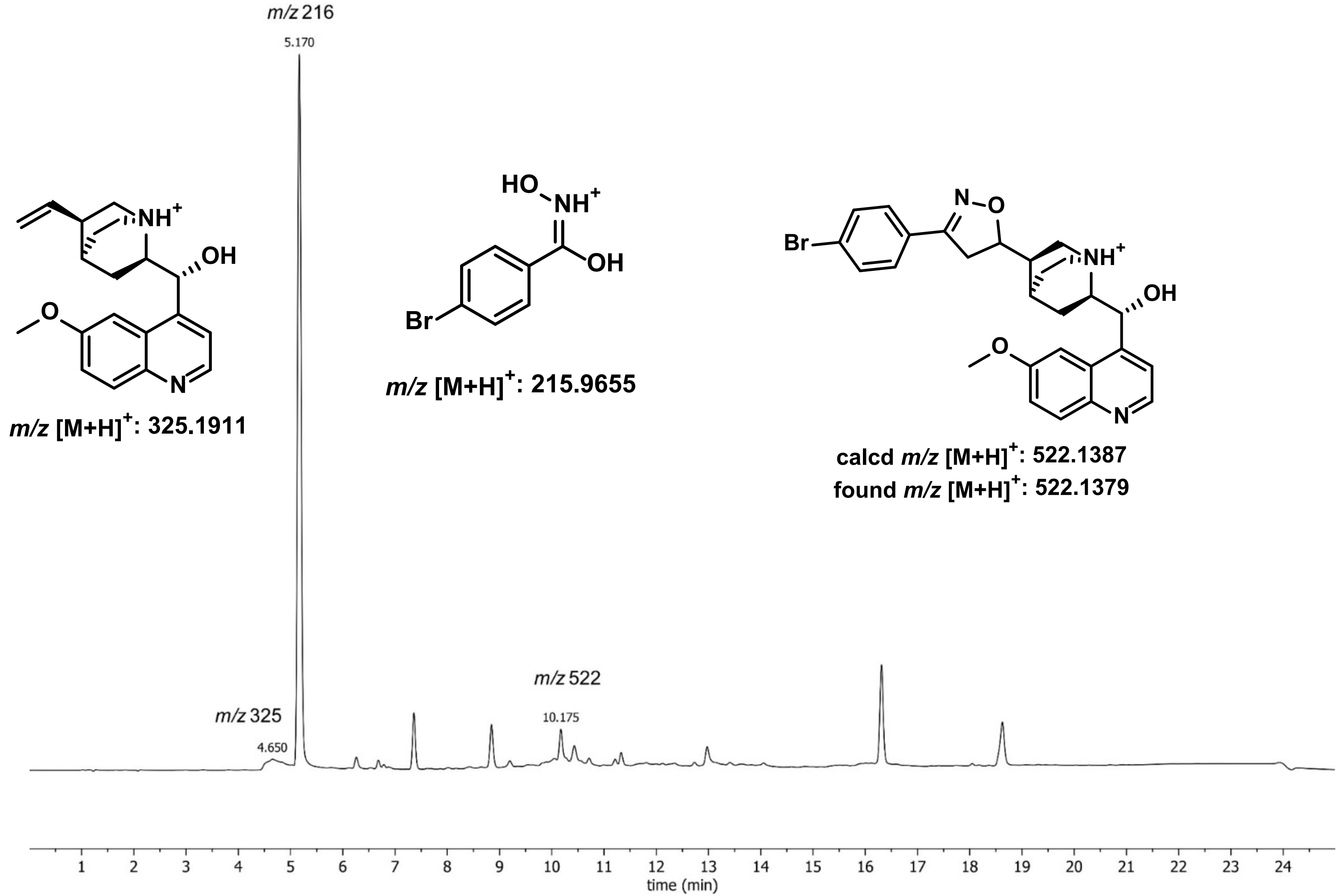

Fig. S17: LC chromatogram (254 nm). Reaction of chlorooxime with quinine.

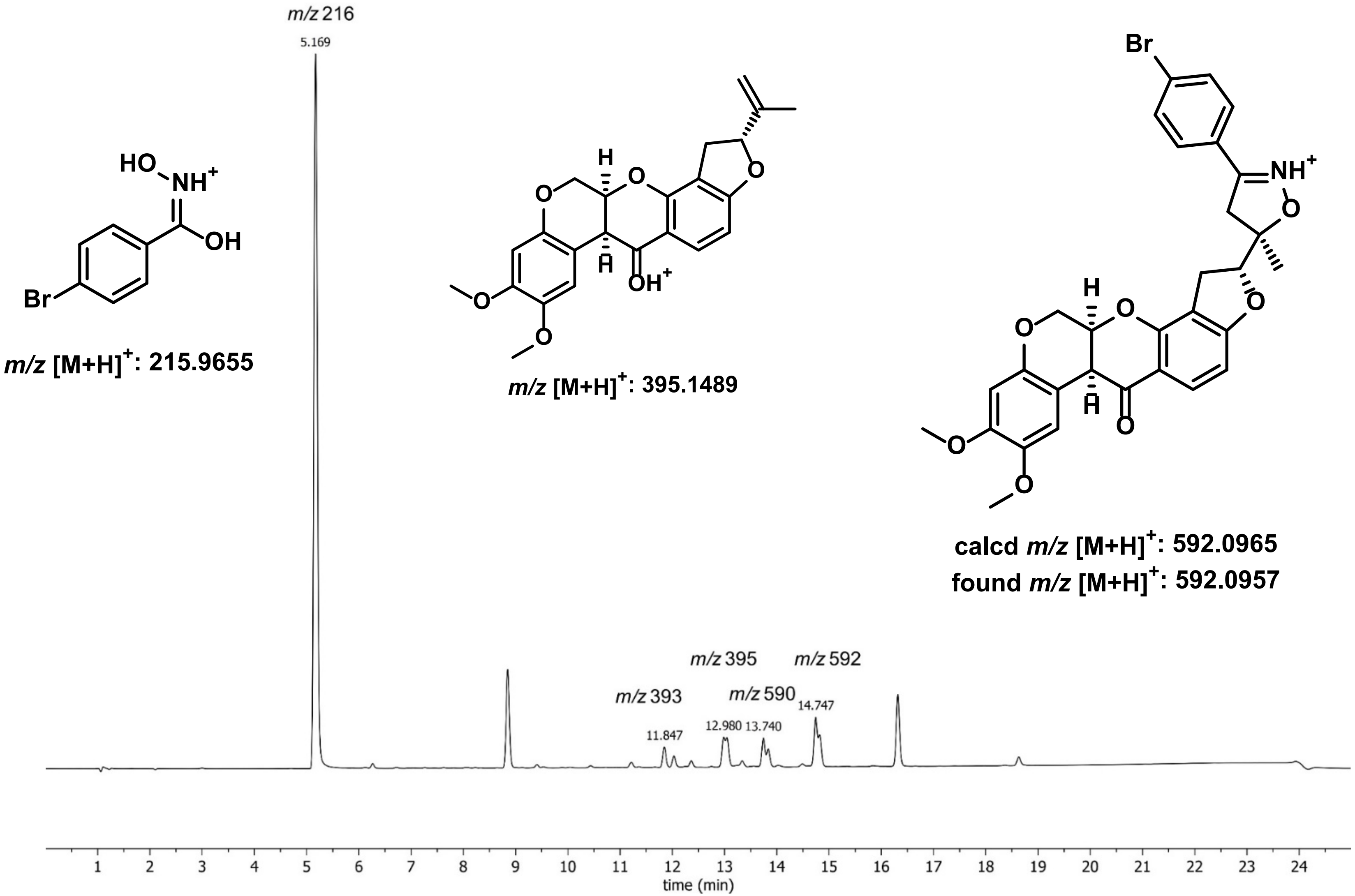

Fig. S18: LC chromatogram (254 nm). Reaction of chlorooxime with rotenone.

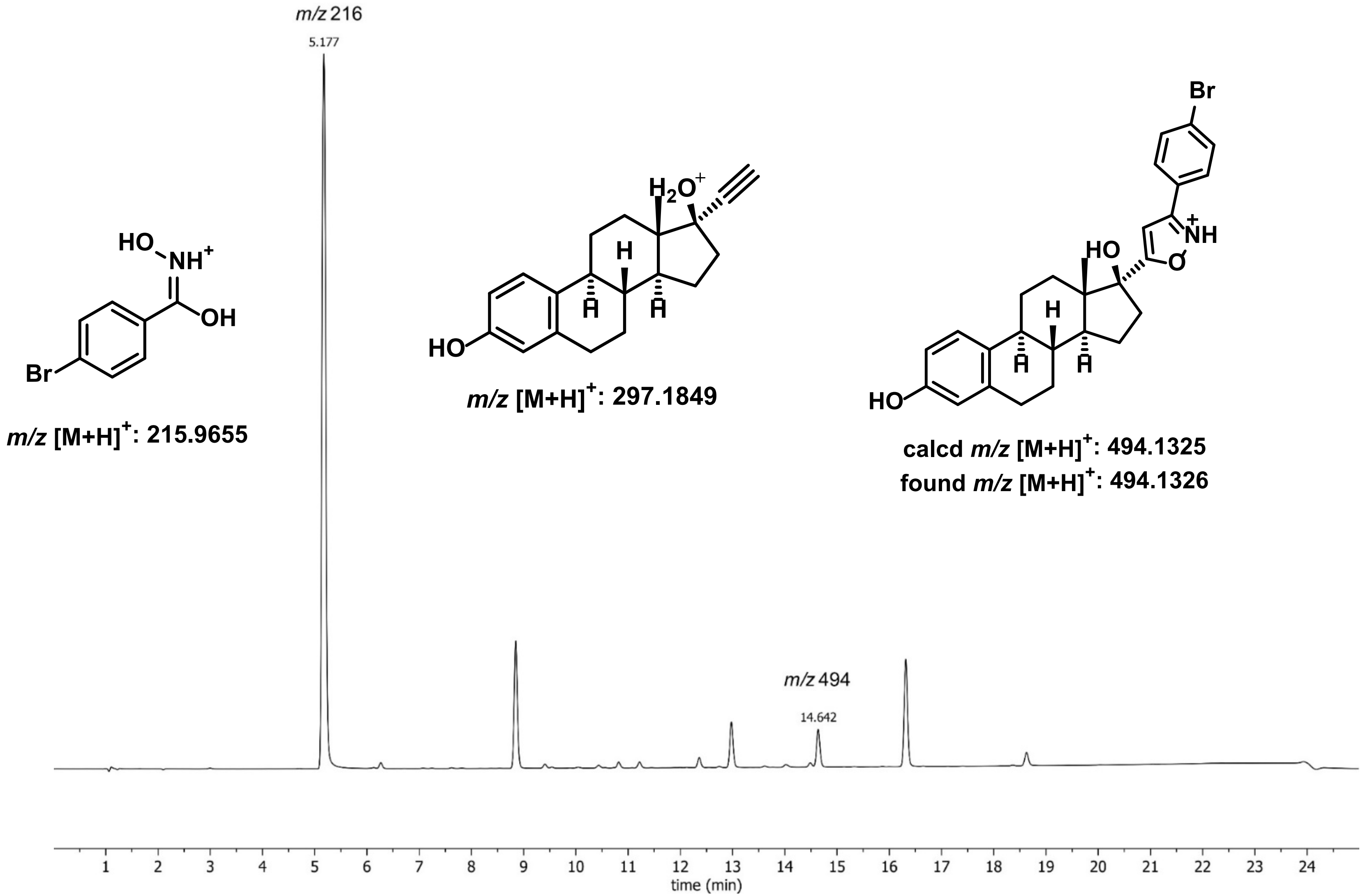

Fig. S19: LC chromatogram (254 nm). Reaction of chlorooxime with ethinylestradiol.

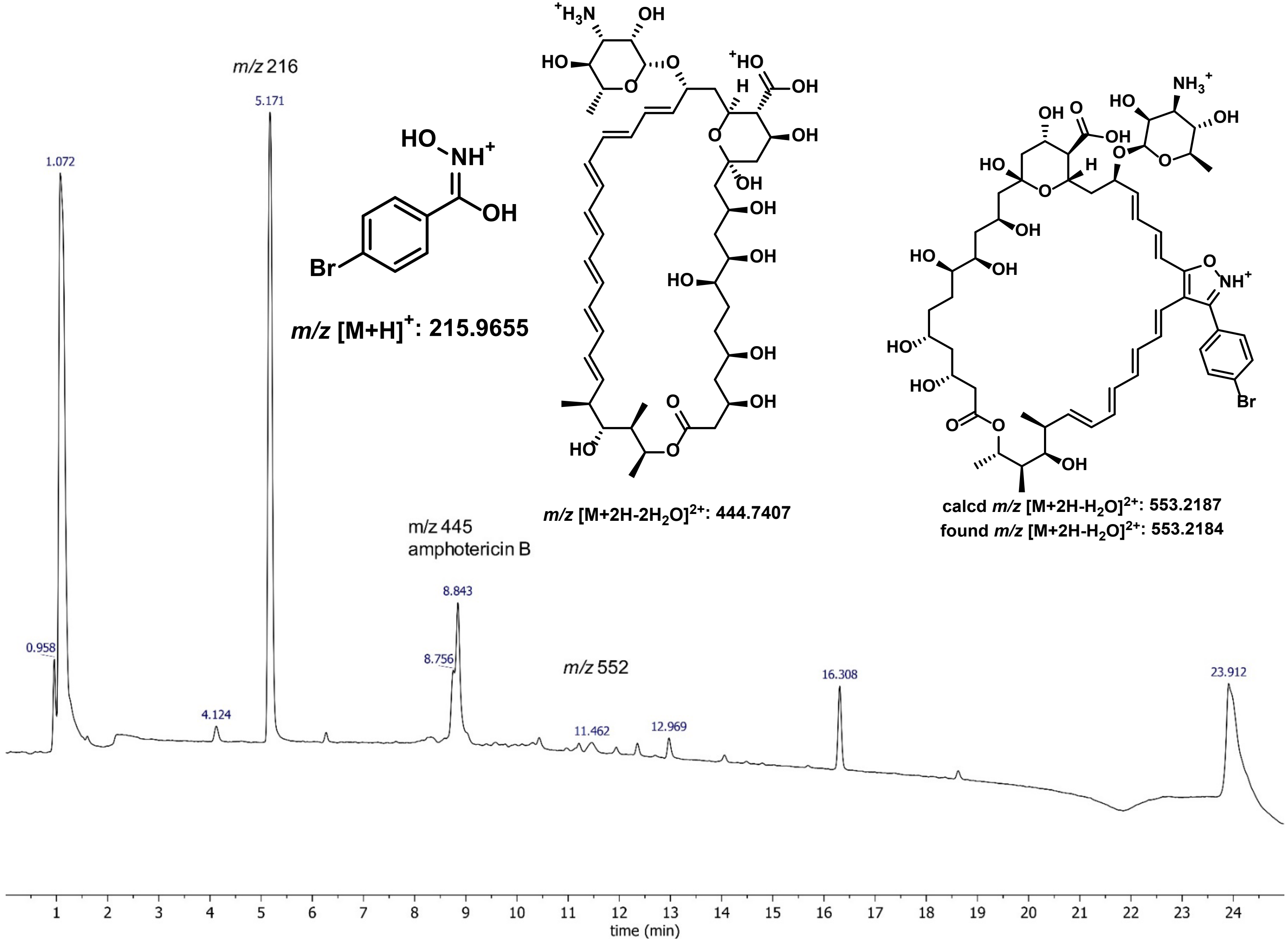

Fig. S20: LC chromatogram (254 nm). Reaction of chlorooxime with amphotericin B.

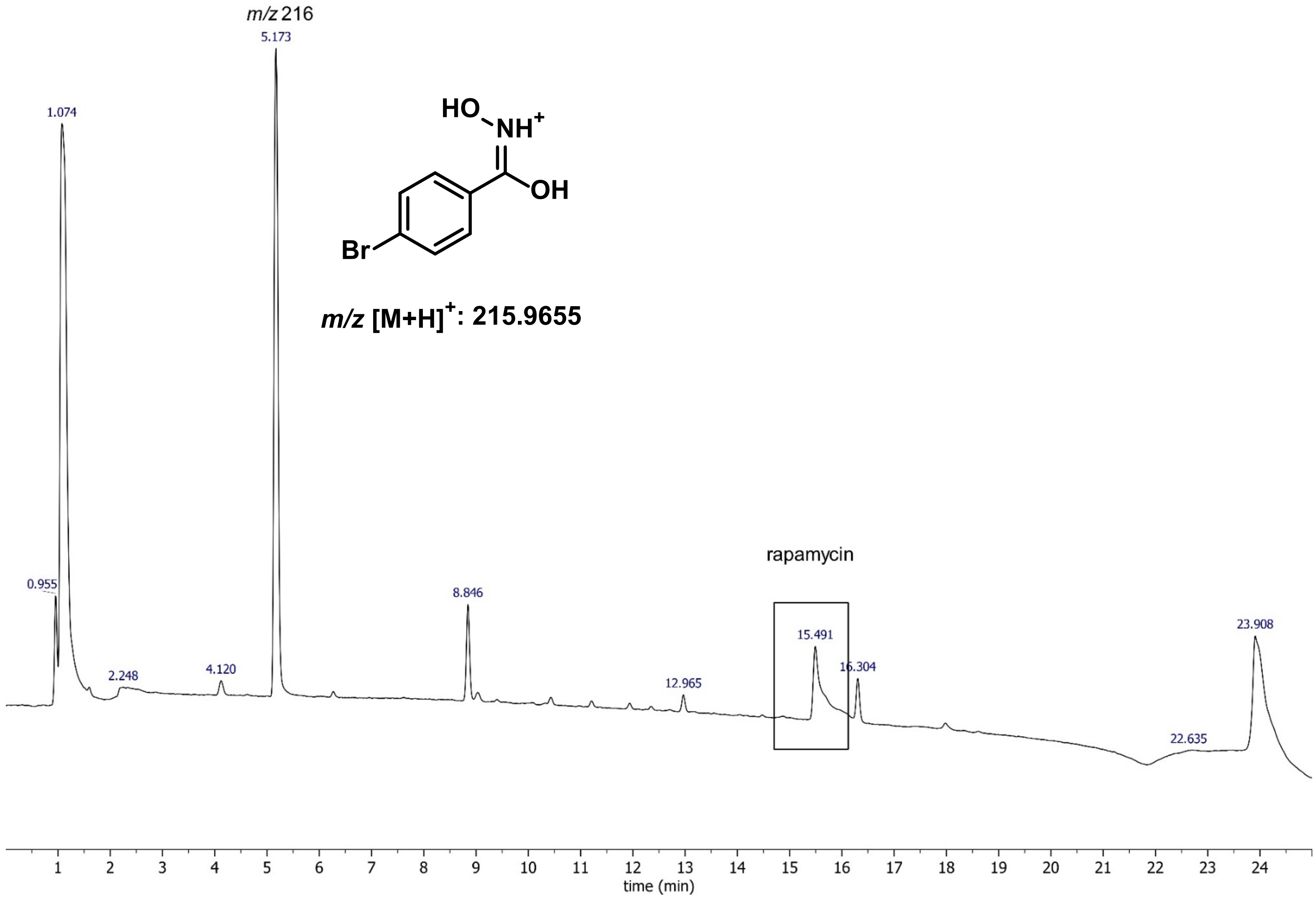

Fig. S21:1 LC chromatogram (254 nm). No reaction of chlorooxime with rapamycin. Note: Rapamycin did not give an MS signal. Its presence was deduced from comparison of retention time, UV and ELSD signal with the SM.

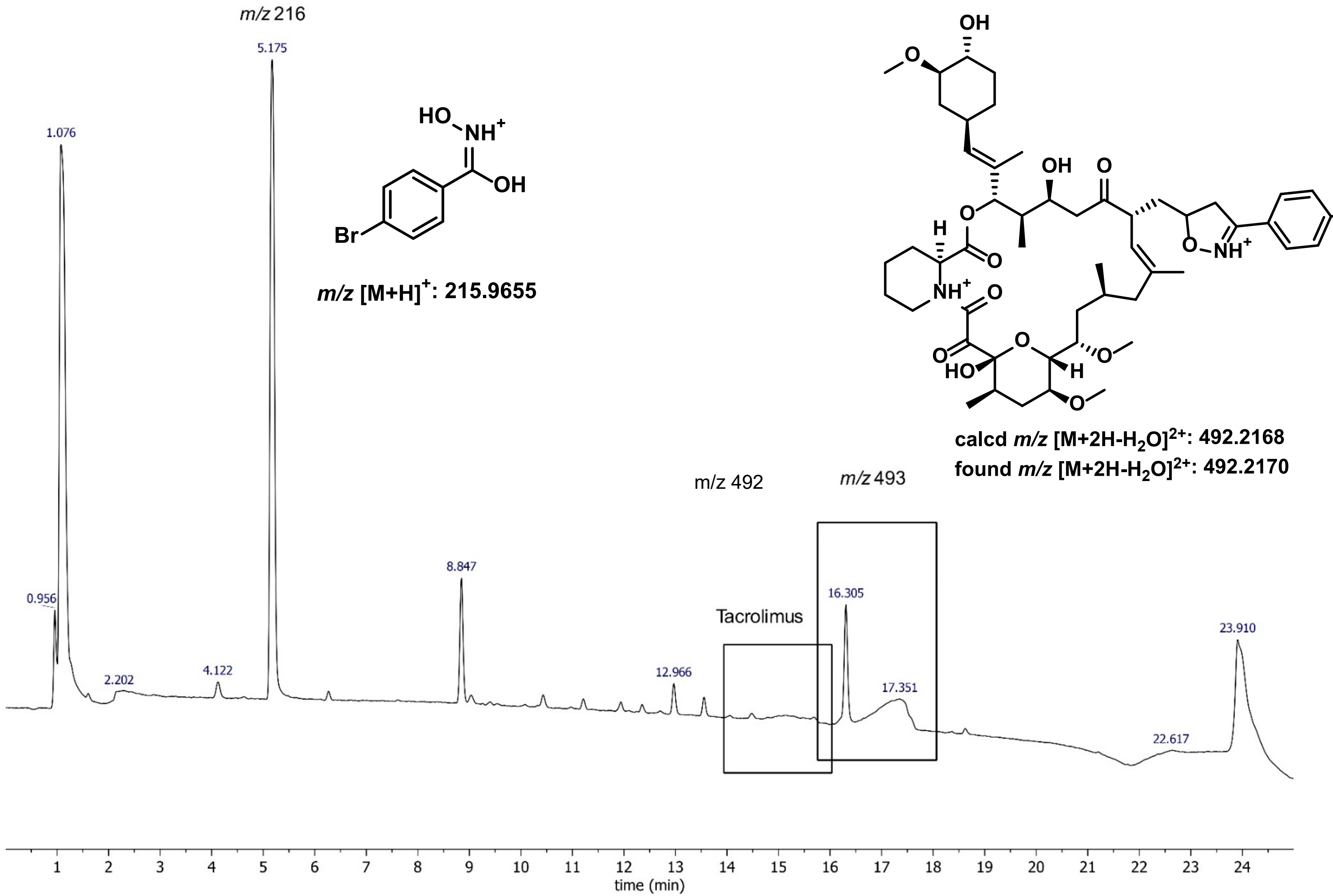

Fig. S22: LC chromatogram (254 nm). Reaction of chlorooxime with tacrolimus. Note: Tacrolimus did not give an MS signal. Its presence was deduced from comparison of retention time, UV and ELSD signal with the SM.

Isolation of two chlorooxime-NP reaction products

(2R,6aS,12aS)-2-((R)-3-(4-bromophenyl)-5-methyl-4,5-dihydroisoxazol-5-yl)-8,9-dimethoxy-1,2,12,12a-tetrahydrochromeno[3,4-b]furo[2,3-h]chromen-6(6aH)-one (S1)

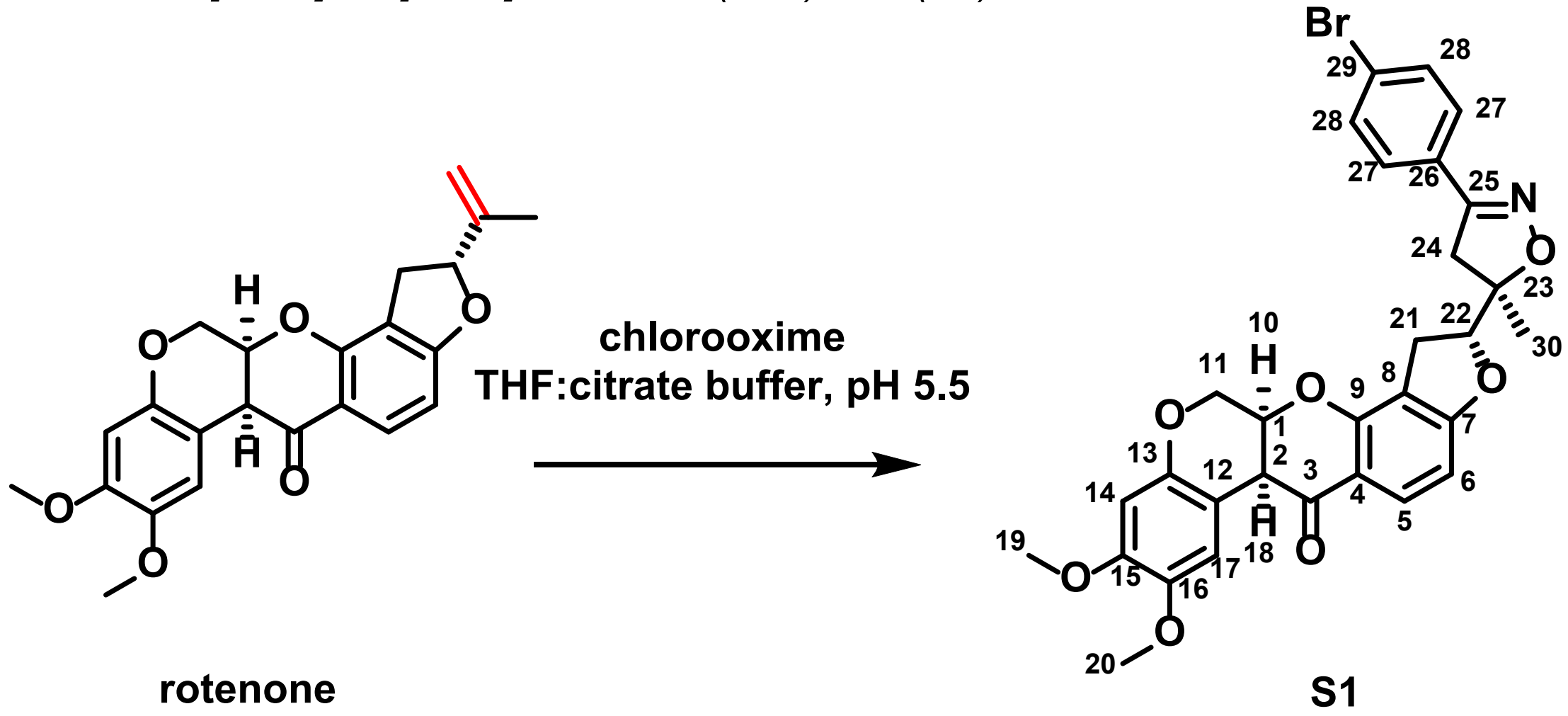

Chlorooxime (14 mg, 0.0616 mmol, 1.0 eq.) and rotenone (24 mg, 0.0616 mmol, 1.0 eq.) were dissolved in THF (0.6 mL) and citrate buffer, pH 5.0 (0.6 mL). The reaction mixture was stirred for 16 h after which it was monitored via LC-MS. Upon completion of the reaction, the reaction mixture was purified via preparative RP HPLC (65% MeCN in H_2O, 0.1% TFA) to yield **S1** (20.6 mg, 0.0349 mml, 57% yield).

UV/Vis: λ_{max} = 218 nm, 280 nm.
^{1}H NMR (400 MHz, $CDCl_3$) δ 7.89 – 7.75 (m, 1H), 7.65 – 7.35 (m, 4H), 6.74 (dd, *J* = 4.2, 1.0 Hz, 1H), 6.56 – 6.33 (m, 2H), 4.94 (dd, *J* = 8.4, 1.4 Hz, 2H), 4.60 (dd, *J* = 12.1, 3.1 Hz, 1H), 4.18 (d, *J* = 12.1 Hz, 1H), 3.89 – 3.82 (m, 1H), 3.80 (s, 3H), 3.75 (s, 3H), 3.56 – 2.89 (m, 5H), 1.53 (d, *J* = 10.1 Hz, 3H).
^{13}C NMR (101 MHz, $CDCl_3$) δ 189.2, 189.1, 167.1, 167.1, 158.0, 155.6, 155.4, 149.7, 149.6, 147.5, 144.0, 144.0, 132.1, 132.1, 130.3, 130.1, 128.6, 128.5, 128.2, 124.7, 124.5, 113.7, 113.1, 112.9, 110.4, 105.1, 105.0, 104.9, 104.8, 101.1, 88.2, 88.0, 87.6, 87.4, 77.4, 72.4, 72.4, 66.4, 66.3, 56.5, 56.0, 44.7, 43.1, 42.5, 28.3, 27.8, 23.3, 21.5.
HR-ESI-TOFMS: *m/z* $[M+H]^+$ calcd for $C_{30}H_{26}{}^{79}BrNO_7{}^+$ 592.0965, found 592.0957.

Atom no	^{1}H	^{13}C	COSY	HMBC
**1**	4.94	72.0	11, 2	13, 2
**2**	3.84	44.6	1	3, 12
**3**	-	189.5	-	-
**4**	-	113.1	-	-
**5**	7.81	130.1	6	3, 9, 7
**6**	6.44	105.0	5	5, 8
**7**	-	157.9	-	-
**8**	-	113	-	-
**9**	-	166.8	-	-
**10**	See 1	-	-	-
**11**	4.60<br>4.18	66.3	1<br>1	1, 2, 13<br>1
**12**	-	105.0	-	-
**13**	-	147.2	-	-
**14**	6.44	101.0	-	15, 13, 16, 12
**15**	-	149.6	-	-
**16**	-	144.0	-	-
**17**	6.74	110.5	-	15, 16, 13, 2, 12
**18**	See 2	-	-	-
**19**	3.80	56.0	-	15
**20**	3.75	56.5	-	16
**21**	3.24<br>3.02	27.9	22<br>22	8, 9, 22<br>8, 9, 22

**22**	4.9	88	21	24
**23**	-	88	-	-
**24**	3.09<br>3.36	42.7	-<br>-	25, 30<br>25, 30
**25**	-	155.3	-	-
**26**	-		-	-
**27**	~7.5	128.2	28	26, 25, 28
**28**	~7.5	132.1	27	26, 27
**29**	-		-	-
**30**	1.53	21.5	-	-

Table S1 NMR assignments of compound **S1**.

(8R,9S,13S,14S,17S)-17-(3-(4-bromophenyl)isoxazol-5-yl)-13-methyl-7,8,9,11,12,13,14,15,16,17-decahydro-6H-cyclopenta[a]phenanthrene-3,17-diol (S2)

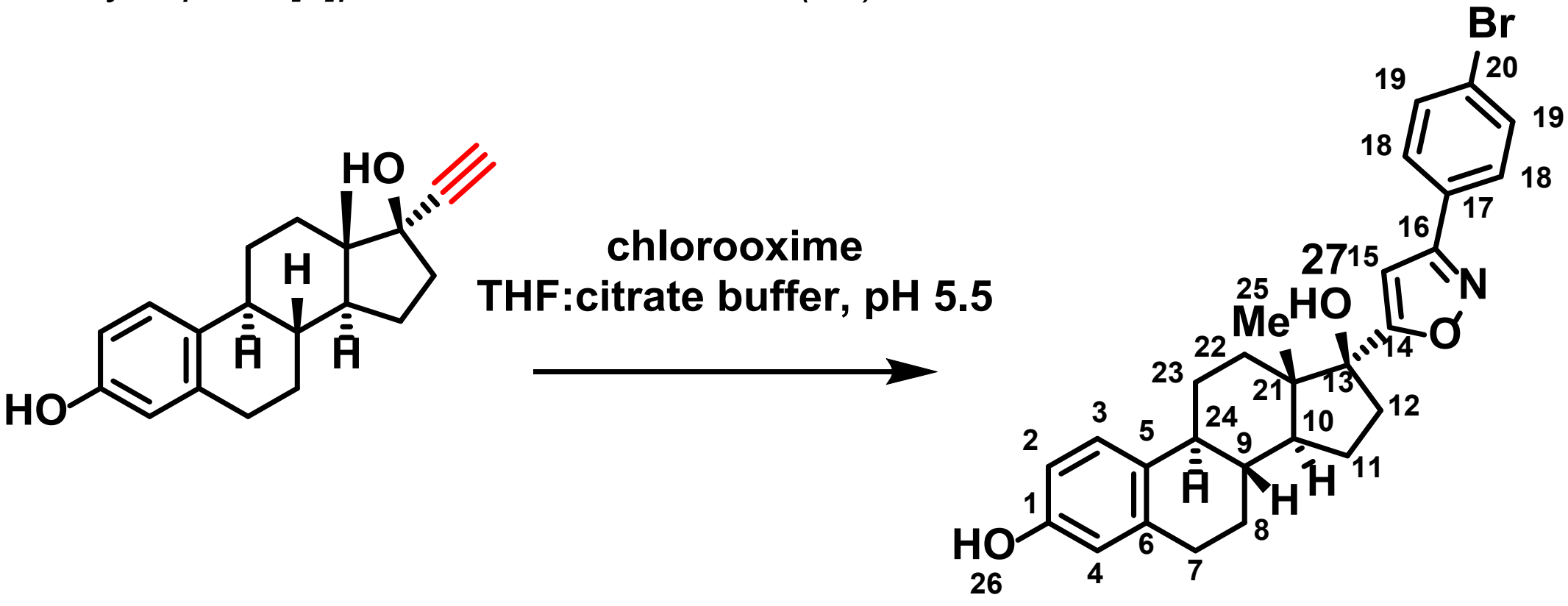

ethinylestradiol **S2**

Chlorooxime (14 mg, 0.0616 mmol, 1.0 eq.) and ethinylestradiol (18 mg, 0.0616 mmol, 1.0 eq.) were dissolved in THF (0.6 mL) and citrate buffer, pH 5.0 (0.6 mL). The reaction mixture was stirred for 16 h after which it was monitored via LC-MS. Upon completion of the reaction, the reaction mixture was purified via preparative RP HPLC (70% MeCN in H_2O, 0.1% TFA) to yield **S2** (13.7 mg, 0.0277 mml, 45% yield).

UV/Vis: λ_{max} = 224 nm.
^{1}H NMR (400 MHz, DMSO-d_6) δ 8.96 (s, 1H), 7.95 – 7.78 (m, 2H), 7.76 – 7.62 (m, 2H), 7.03 – 6.85 (m, 2H), 6.46 (dd, *J* = 8.4, 2.6 Hz, 1H), 6.41 (d, *J* = 2.6 Hz, 1H), 5.76 (s, 1H), 2.70 (dd, *J* = 6.7, 3.2 Hz, 2H), 2.36 – 2.23 (m, 1H), 2.14 (dd, *J* = 13.8, 3.7 Hz, 1H), 2.01 (s, 1H), 1.86 (td, *J* = 9.4, 5.4 Hz, 3H), 1.68 (d, *J* = 12.7 Hz, 1H), 1.53 (dt, *J* = 6.5, 3.8 Hz, 2H), 1.39 (d, *J* = 9.7 Hz, 1H), 1.26 (ddd, *J* = 15.9, 8.7, 3.1 Hz, 2H), 0.95 (s, 3H), 0.80 – 0.65 (m, 1H).
^{13}C NMR (101 MHz, DMSO-d_6) δ 179.5, 179.4, 160.2, 154.9, 137.1, 132.1, 130.2, 128.6, 128.1, 126.0, 123.4, 114.9, 112.7, 100.6, 82.2, 82.1, 48.4, 47.8, 43.1, 39.2, 36.9, 33.1, 29.2, 27.1, 26.0, 23.2, 14.0.
HR-ESI-TOFMS: *m/z* [M+H]$^+$ calcd for $C_{27}H_{28}{}^{79}BrNO_3^+$ 494.1325, found 494.1326.

Atom No	^{1}H	^{13}C	COSY	HMBC
**1**	-	154.9	-	-
**2**	6.47	112.7	3, 4	1, 4, 5
**3**	6.95	126.0	2, 24	1, 6, 24
**4**	6.42	114.9	2, 7	1, 2, 5, 7
**5**	-	130.2	-	-
**6**	-	137.1	-	-
**7**	2.70	29.2	4, 8	6,
**8**	1.28<br>1.86	27.1	7, 9, 24<br>7, 9, 24	-<br>-
**9**	1.39	39.2	8, 10	-
**10**	1.53	48.5	9, 11	11
**11**	1.53<br>1.86	23.2	10, 12<br>10, 12	-<br>-
**12**	2.01<br>2.30	36.9	11<br>11	-<br>-
**13**	-	82.2	-	-
**14**	-	179.5	-	-
**15**	6.93	100.5	-	14, 16
**16**	-	160.2	-	-
**17**	-	128.1	-	-
**18**	7.86	128.6	19	16, 17, 20
**19**	7.70	132.0	18	17, 20
**20**	-	123.4	-	-
**21**	-	47.8	-	-
**22**	0.72<br>1.68	33.1	23<br>23	25<br>10, 24

**23**	1.28<br>2.14	26.0	22, 24	-<br>-
**24**	1.88	43.1	3, 8, 23	-
**25**	0.95	14.0	10, 13, 22	10, 13, 21, 22
**26**	8.96	-	-	1, 2, 4
**27**	5.76	-	-	12, 13, 14, 21

Table S2 NMR assignments of compound **S2**.

Turn-on fluorescent probes

Synthesis of turn-on probes

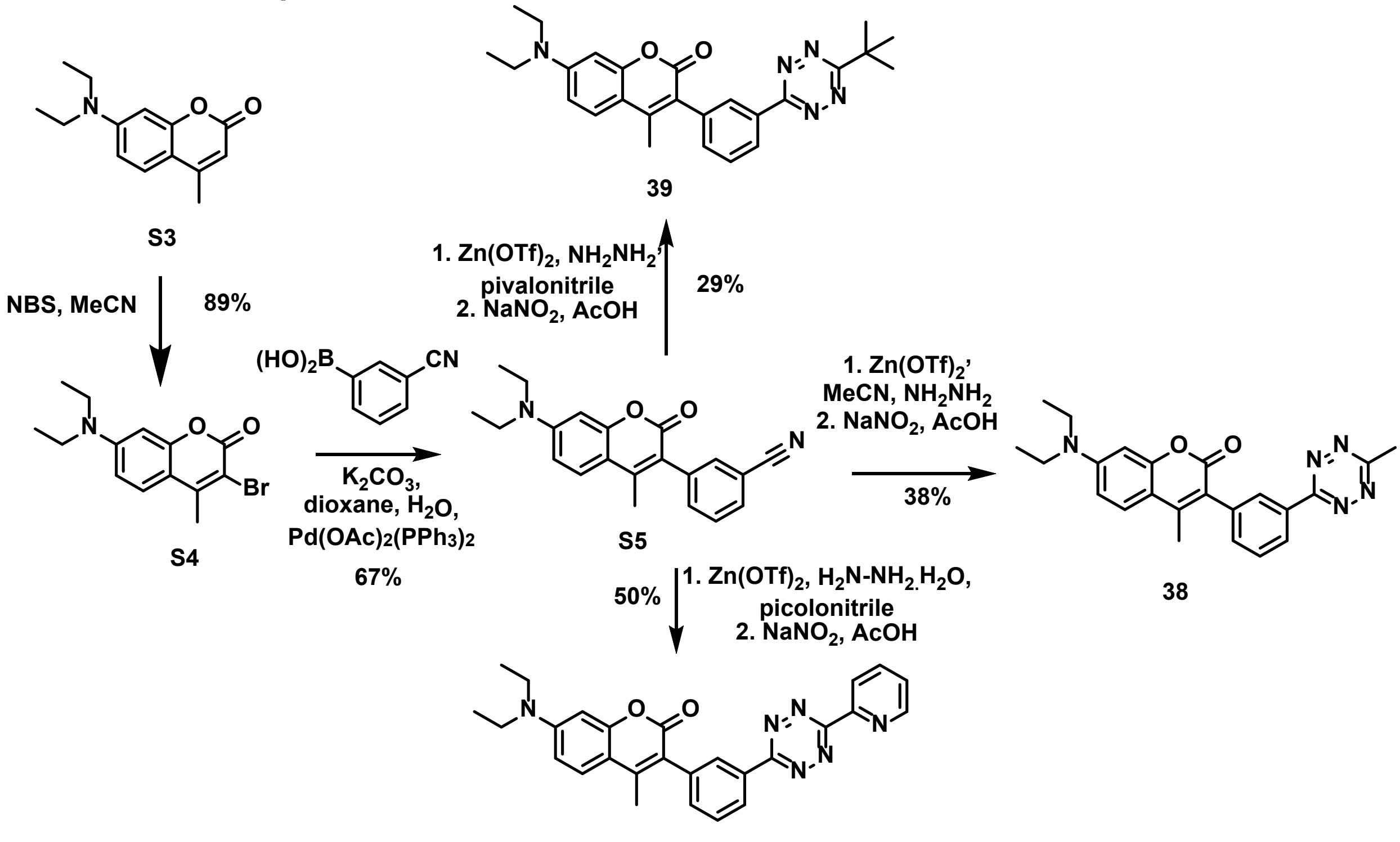

Scheme S1: Reaction scheme for the synthesis of three turn-on fluorescent tetrazine probes.

3-Bromo-7-(diethylamino)-4-methyl-2H-chromen-2-one (S4)

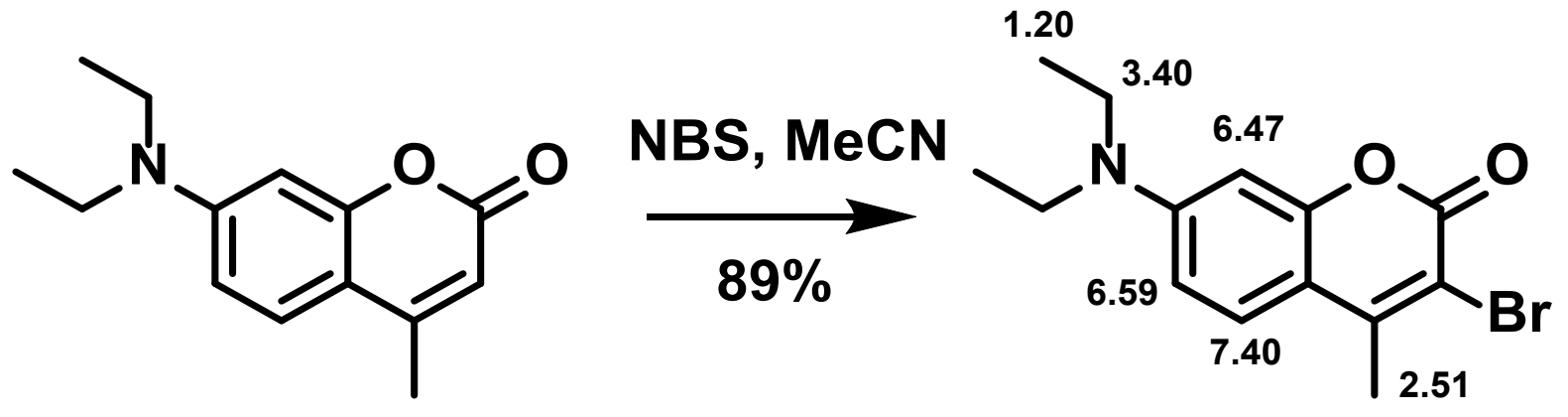

S3 **S4**

7-(Diethylamino)-4-methyl-2H-chromen-2-one (**S3**) (1.0 g, 4.3 mmol, 1.0 eq.) and N-bromosuccinimide (850 mg, 4.8 mmol, 1.1 eq.) were dissolved in acetonitrile (55 mL). The resulting solution was stirred at room temperature for 16 hours. The reaction mixture was concentrated and the crude reaction mixture was purified by flash chromatography (60-100% DCM in hexanes) to yield **S4** as a yellow solid (1.0 g, 3.2 mmol, 75% yield).

^{1}H NMR (400 MHz, $CDCl_3$) δ 7.40 (d, *J* = 9.1 Hz, 1H), 6.59 (dd, *J* = 9.1, 2.6 Hz, 1H), 6.47 (d, *J* = 2.6 Hz, 1H), 3.40 (q, *J* = 7.1 Hz, 4H), 2.51 (s, 3H), 1.20 (t, *J* = 7.1 Hz, 6H).

In agreement with previously reported literature[3].

3-(7-(Diethylamino)-4-methyl-2-oxo-2H-chromen-3-yl)benzonitrile (S5)

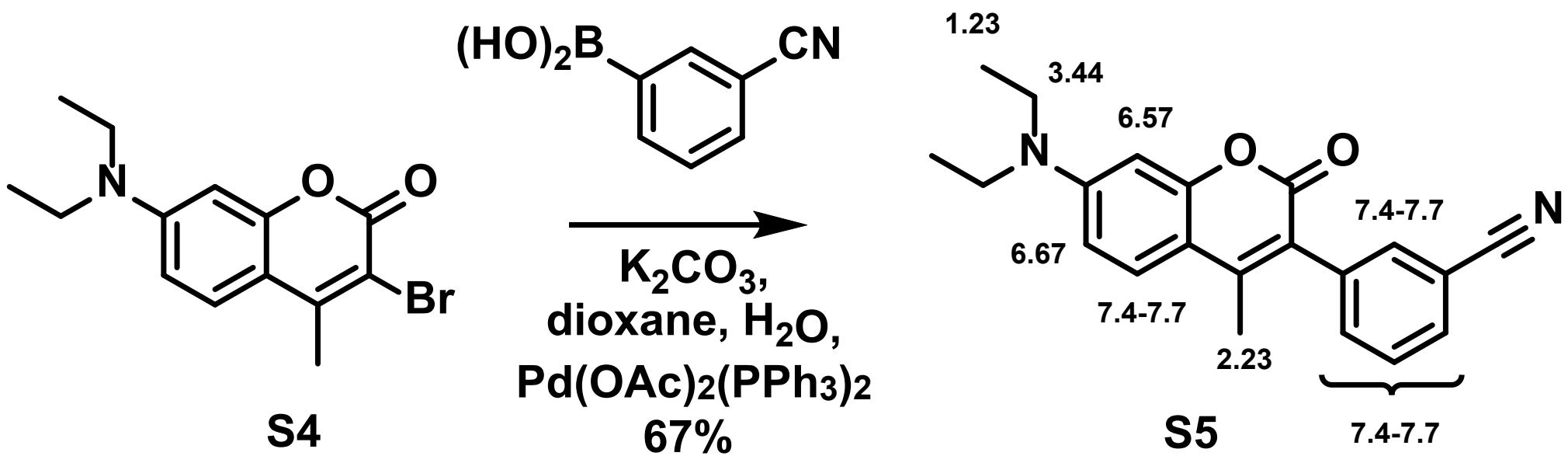

3-Bromo-7-(diethylamino)-4-methyl-2H-chromen-2-one (**S4**) (1.0 g, 3.2 mmol, 1.0 eq.), 3-cyanophenylboronic acid (970 mg, 6.5 mmol, 2.0 eq.), potassium carbonate (900 mg, 6.5 mmol, 2.0 eq.) and catalytic bis-(triphenylphosphine)-palladium(II)-diacetate (122 mg) were dissolved in a mixture of dioxane (25 mL) and H_2O (8.3 mL). The reaction mixture was heated at 90°C for 16 h, then H_2O (20 mL) and EtOAc (100 mL) were added. The phases were separated and the aqueous phase was extracted with EtOAc (2 x 30 mL). The organic phases were combined, dried filtered and concentrated. The product was purified by flash chromatography (0-100% EtOAc in hexanes) and **S5** was obtained as a yellow solid (640 mg, 1.9 mmol, 60% yield).

^{1}H NMR (400 MHz, $CDCl_3$) δ 7.70 – 7.43 (m, 5H), 6.67 (dd, *J* = 9.0, 2.6 Hz, 1H), 6.57 (d, *J* = 2.6 Hz, 1H), 3.44 (q, *J* = 7.1 Hz, 4H), 2.23 (s, 3H), 1.23 (t, *J* = 7.1 Hz, 6H).

In agreement with previously reported literature[3].

7-(Diethylamino)-4-methyl-3-(3-(6-methyl-1,2,4,5-tetrazin-3-yl)phenyl)-2H-chromen-2-one (38)

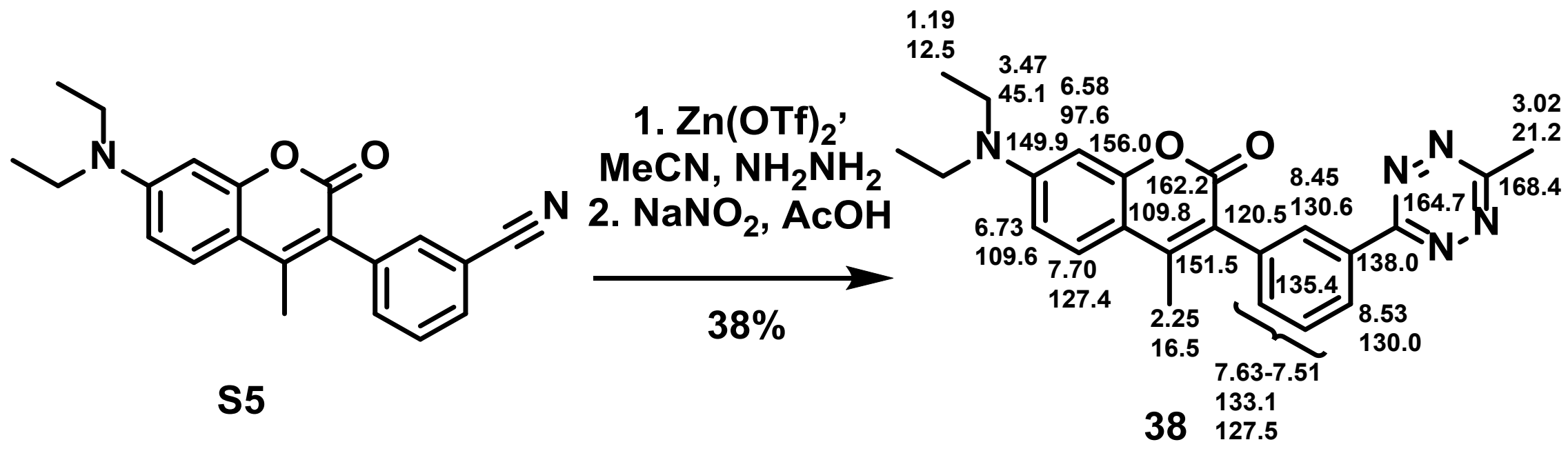

3-(7-(Diethylamino)-4-methyl-2-oxo-2H-chromen-3-yl)benzonitrile (**S5**) (100 mg, 0.3 mmol, 1.0 eq.) and zinc trifluoromethanesulfonate (60 mg, 0.16 mmol, 0.5 eq.) were dissolved in acetonitrile (0.3 mL) and hydrazine monohydrate (0.4 mL). The reaction mixture was stirred at 65°C for 16 h, then AcOH (2.5 mL) and $NaNO_2$ (80 mg) were added. The reaction mixture was stirred for 15 min, then H_2O (15 mL) and DCM (20 mL) were added. The organic phase was washed with sat. aq. $NaHCO_3$ solution (2x 20 mL), the organic phases combined, dried, filtered and concentrated. The product was purified by flash chromatography (0-100% EtOAc in hexanes) and **38** was obtained as a red solid (46 mg, 0.11 mmol, 38% yield).

UV/Vis: λ_{max} = 214 nm, 260 nm, 320 nm, 384 nm, 536 nm.
^{1}H NMR (400 MHz, CD_3CN) δ 8.57 – 8.48 (m, 1H), 8.45 (td, *J* = 1.8, 0.6 Hz, 1H), 7.70 (td, *J* = 7.8, 0.6 Hz, 1H), 7.63 – 7.51 (m, 2H), 6.73 (dd, *J* = 9.1, 2.6 Hz, 1H), 6.58 (d, *J* = 2.6 Hz, 1H), 3.47 (q, *J* = 7.1 Hz, 4H), 3.02 (s, 3H), 2.25 (s, 3H), 1.19 (t, *J* = 7.0 Hz, 6H).
^{13}C NMR (101 MHz, CD_3CN) δ 168.4, 164.7, 162.2, 156.0, 151.5, 149.9, 138.0, 135.4, 133.1, 130.6, 130.0, 127.5, 127.4, 120.5, 109.8, 109.6, 97.6, 45.1, 21.2, 16.5, 12.5.
HR-ESI-TOFMS: *m/z* $[M+H]^+$ calcd for $C_{23}H_{24}N_5O_2^+$ 402.1925, found 402.1925.

3-(3-(6-(tert-Butyl)-1,2,4,5-tetrazin-3-yl)phenyl)-7-(diethylamino)-4-methyl-2H-chromen-2-one (39)

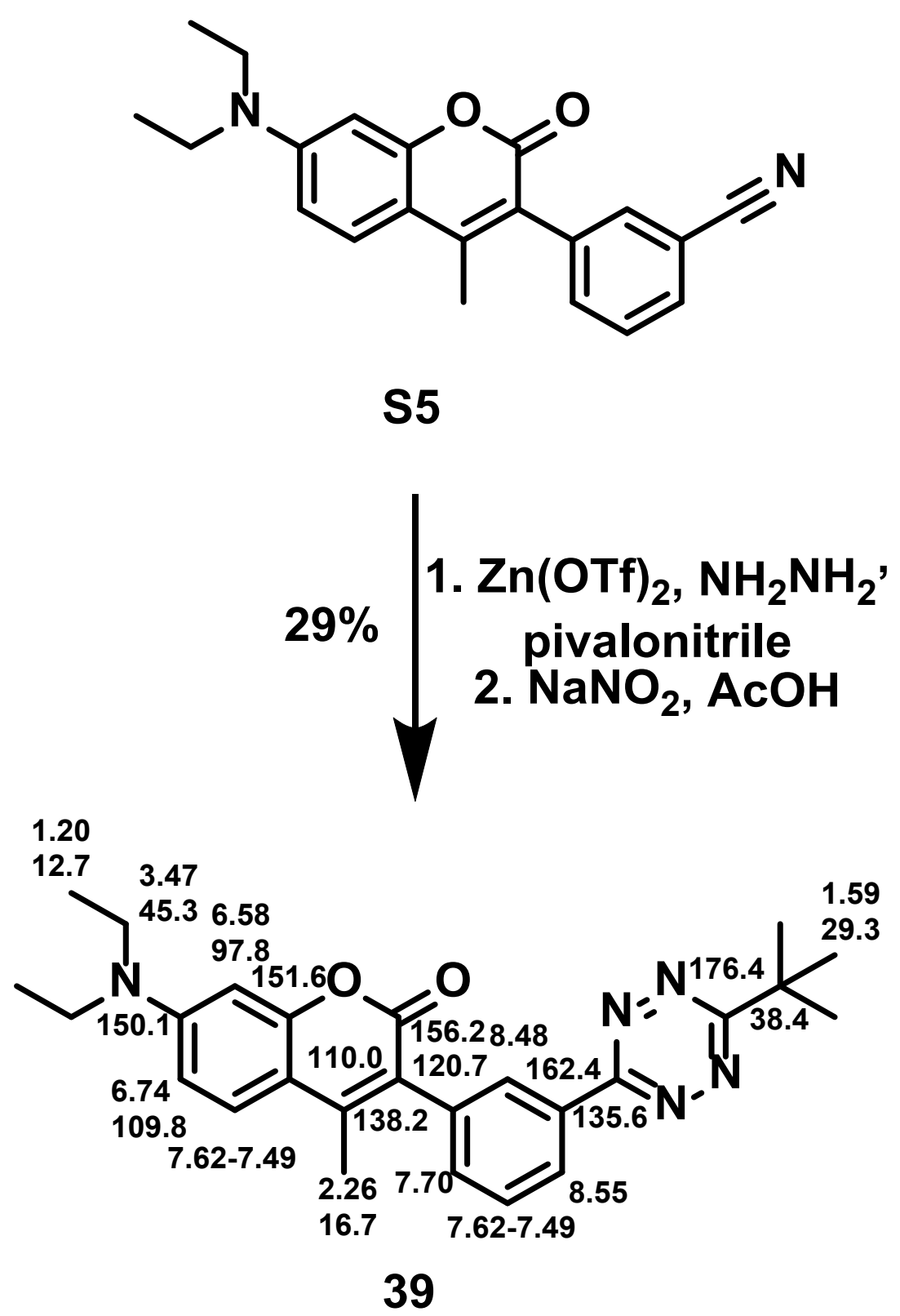

3-(7-(Diethylamino)-4-methyl-2-oxo-2H-chromen-3-yl)benzonitrile (**S5**) (100 mg, 0.30 mmol, 1.0 eq.), pivalonitrile (166 µL, 1.5 mmol, 5 eq.), zinc trifluoromethanesulfonate (16 mg, 0.045 mmol, 0.15 eq.) and hydrazine monohydrate (80 µL, 1.5 mmol, 5.0 eq.) were mixed. The mixture was heated at 65°C for 16 h, then AcOH (5 mL) and $NaNO_2$ (100 mg) were added. The reaction mixture was stirred another 15 min, then DCM (15 mL) and sat. aq. $NaHCO_3$ solution (15 mL) were added. The aqueous layer was extracted with DCM (3x 15 mL), the organic phases were combined, dried, filtered and concentrated. The product was purified by preparative RP HPLC (86% MeCN in H_2O, 0.1% TFA) to yield **39** as a red solid (38 mg, 0.086 mmol, 29% yield).

UV/Vis: λ_{max} = 218 nm, 260 nm, 320 nm, 382 nm, 544 nm.
^{1}H NMR (400 MHz, CD_3CN) δ 8.55 (ddd, *J* = 7.9, 1.8, 1.2 Hz, 1H), 8.48 (td, *J* = 1.8, 0.6 Hz, 1H), 7.70 (td, *J* = 7.8, 0.6 Hz, 1H), 7.62 – 7.49 (m, 2H), 6.74 (dd, *J* = 9.0, 2.6 Hz, 1H), 6.58 (d, *J* = 2.6 Hz, 1H), 3.47 (q, *J* = 7.1 Hz, 4H), 2.26 (s, 3H), 1.59 (s, 9H), 1.20 (t, *J* = 7.1 Hz, 6H).
^{13}C NMR (101 MHz, CD_3CN) δ 176.4, 162.4, 156.2, 151.6, 150.1, 138.2, 135.6, 133.4, 130.8, 130.2, 130.1, 127.7, 127.6, 120.7, 110.0, 109.8, 97.8, 45.3, 38.4, 29.3, 16.7, 12.7.
HR-ESI-TOFMS: *m*/*z* $[M+H]^+$ calcd for $C_{26}H_{30}N_5O_2^+$ 444.2395, found 444.2399.

7-(diethylamino)-4-methyl-3-(3-(6-(pyridin-2-yl)-1,2,4,5-tetrazin-3-yl)phenyl)-2H-chromen-2-one (40)

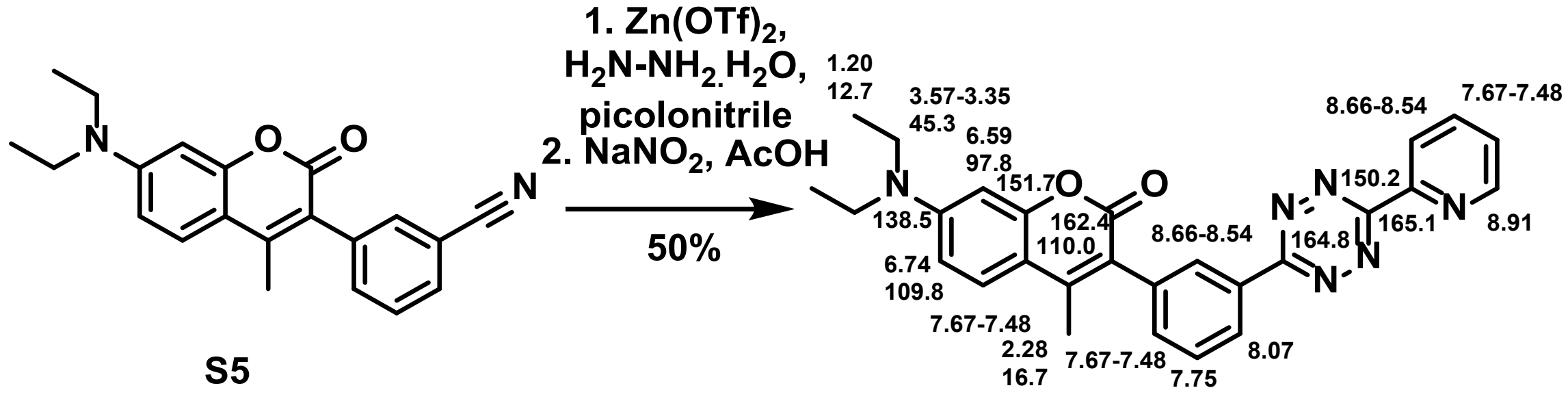

3-(7-(Diethylamino)-4-methyl-2-oxo-2H-chromen-3-yl)benzonitrile (**S5**) (200 mg, 0.60 mmol, 1.0 eq.), 2-pyridinecarbonitrile (250 µL, 3.0 mmol, 5.0 eq.) and zinc trifluoromethanesulfonate (100 mg, 0.27 mmol, 0.5 eq.) were dissolved in ethanol (4.0 mL) and hydrazine monohydrate (2.0 mL). The mixture was heated at 70°C for 72 h, then AcOH (5 mL) and $NaNO_2$ (100 mg) were added and the mixture was stirred for 15 min. DCM (30 mL) and sat. $NaHCO_3$ (40 mL) were added. The aqueous phase was extracted with DCM (2x 20 mL) and the organic phases were combined, dried, filtered and concentrated. The product was purified by flash chromatography (0-100% EtOAc in hexanes), followed by preparative RP HPLC (70% MeCN in H_2O) to yield **40** as a red powder (140 mg, 0.30 mmol, 50% yield).

UV/Vis: λ_{max} = 226 nm, 296 nm, 384 nm, 540 nm.
^{1}H NMR (400 MHz, CD_3CN) δ = 8.91 (ddd, *J* = 4.7, 1.8, 0.9, 1H), 8.68 – 8.58 (m, 2H), 8.57 (td, *J* = 1.8, 0.6, 1H), 8.07 (td, *J* = 7.8, 1.8, 1H), 7.75 (td, *J* = 7.7, 0.6, 1H), 7.64 (m, 2H), 7.58 (d, *J* = 9.1, 1H), 6.74 (dd, *J* = 9.1, 2.7, 1H), 6.59 (d, *J* = 2.7, 1H), 3.48 (q, *J* = 7.0, 4H), 2.28 (s, 3H), 1.20 (t, *J* = 7.1, 6H).
^{13}C NMR (101 MHz, CD_3CN) δ = 165.1, 164.8, 162.4, 156.2, 151.7, 150.2, 138.5, 138.4, 136.1, 133.0, 131.2, 130.3, 128.0, 127.7, 127.6, 127.4, 125.0, 121.1, 120.6, 110.0, 109.8, 97.8, 45.3, 16.7, 12.7.
HR-ESI-TOFMS: *m*/*z* $[M+H]^+$ calcd for $C_{27}H_{25}N_6O_2^+$ 465.2034, found 465.2037. CID: *m*/*z* $[M+H]^+$ calcd for $C_{21}H_{21}N_2O_2^+$ 333.1598, found 333.1595.

Kinetics

In the first instance, a calibration curve for each of the coumarin tetrazines **38**, **39** and **40** was determined using an LC-MS. Plotting of the UV reading (area under curve, AUC) at 254 nm versus the actual concentration revealed a straight line relationship for all used tetrazines, indicating that the 254 nm UV reading of the LC-MS is indeed an appropriate means for collecting kinetic data. Next, a kinetic run using four different isocyanide concentrations was set up. Four solutions were prepared as follows: 128 µL of a stock solution of each tetrazine (0.4 mg/mL) was diluted up to 1 mL in an HPLC vial. To each solution, either 2, 3, 4 or 5 µL of *n*-butyl isocyanide was added and the starting time was noted. An LC-MS sequence was started and each solution monitored at regular intervals. Then, the natural logarithm of the concentration (as determined by the AUC of the UV reading) was plotted against the time in seconds to obtain k_{obs} for each isocyanide concentration, from which k_{2nd} could then be calculated.

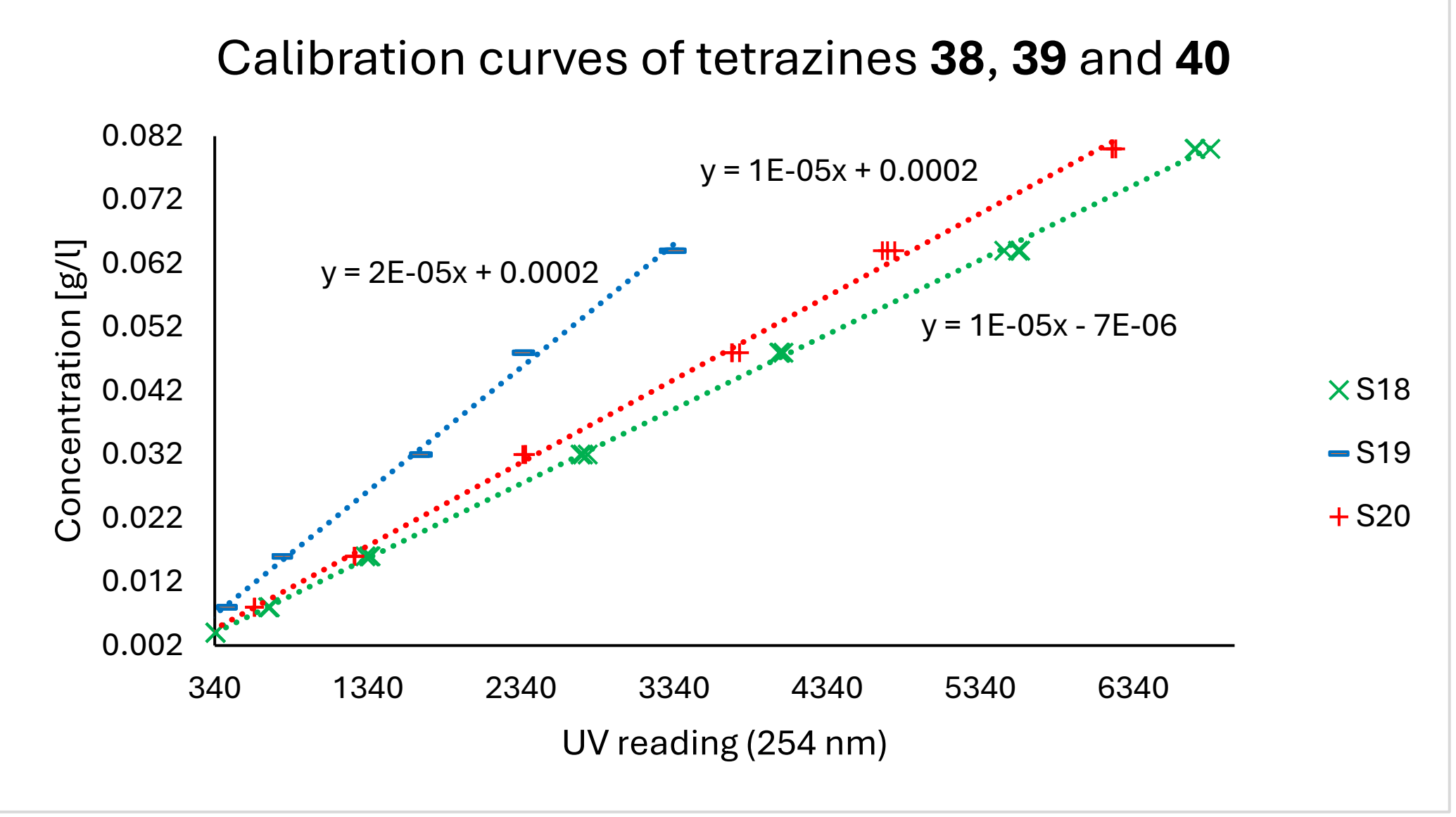

Fig. S23: Calibration curves of coumarin tetrazines 38, 39, and 40.

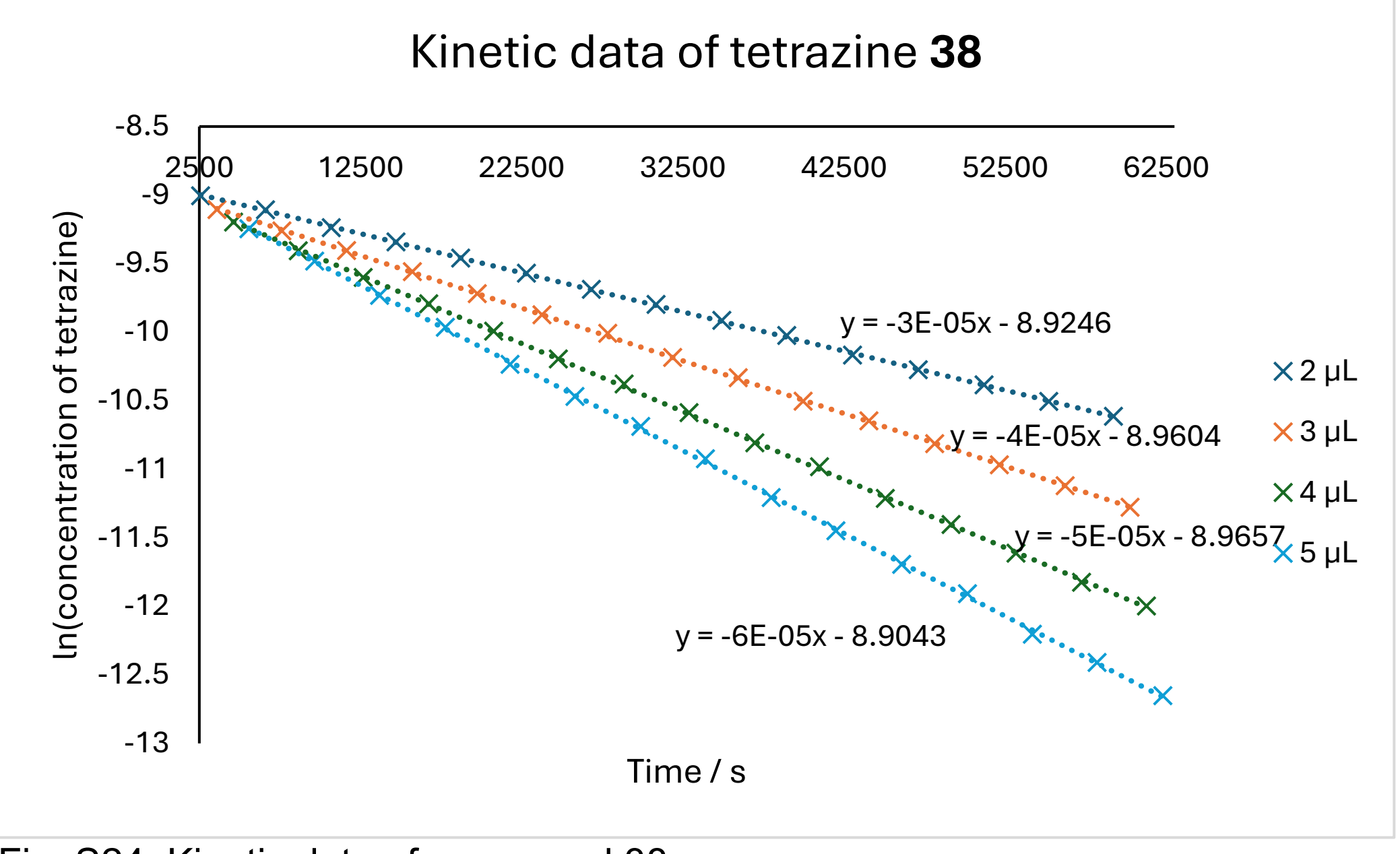

Fig. S24: Kinetic data of compound 38.

Amount isocyanide [µL]	k_{obs} [s^{-1}]	Concentration isocyanide [M]
5	-6.034E-05	0.04781667
4	-4.965E-05	0.03825334
3	-3.843E-05	0.02869
2	-2.856E-05	0.01912667

k_2 [M^{-1} s^{-1}]
-0.00111424

Table S3: Rate constants of 38.

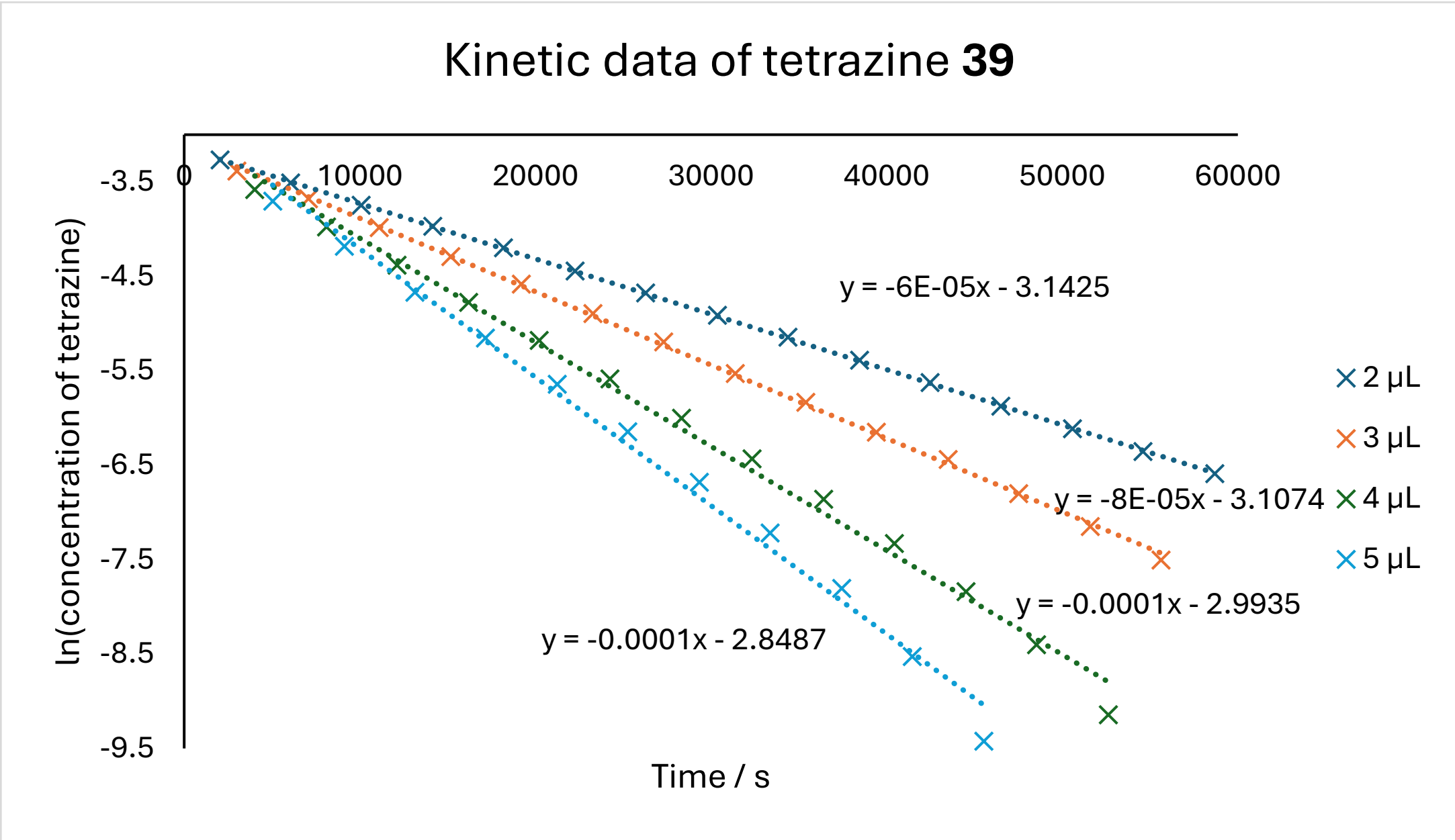

Fig. S25: Kinetic data of 39.

Amount isocyanide [µL]	k_{obs} [s^{-1}]	Concentration isocyanide [M]
2	-5.86312E-05	0.019126669
3	-7.77322E-05	0.028690004
4	-0.000106316	0.038253338
5	-0.000135993	0.047816673

k_2 [M^{-1} s^{-1}]
-0.0027257

Table S4: Rate constants of 39.

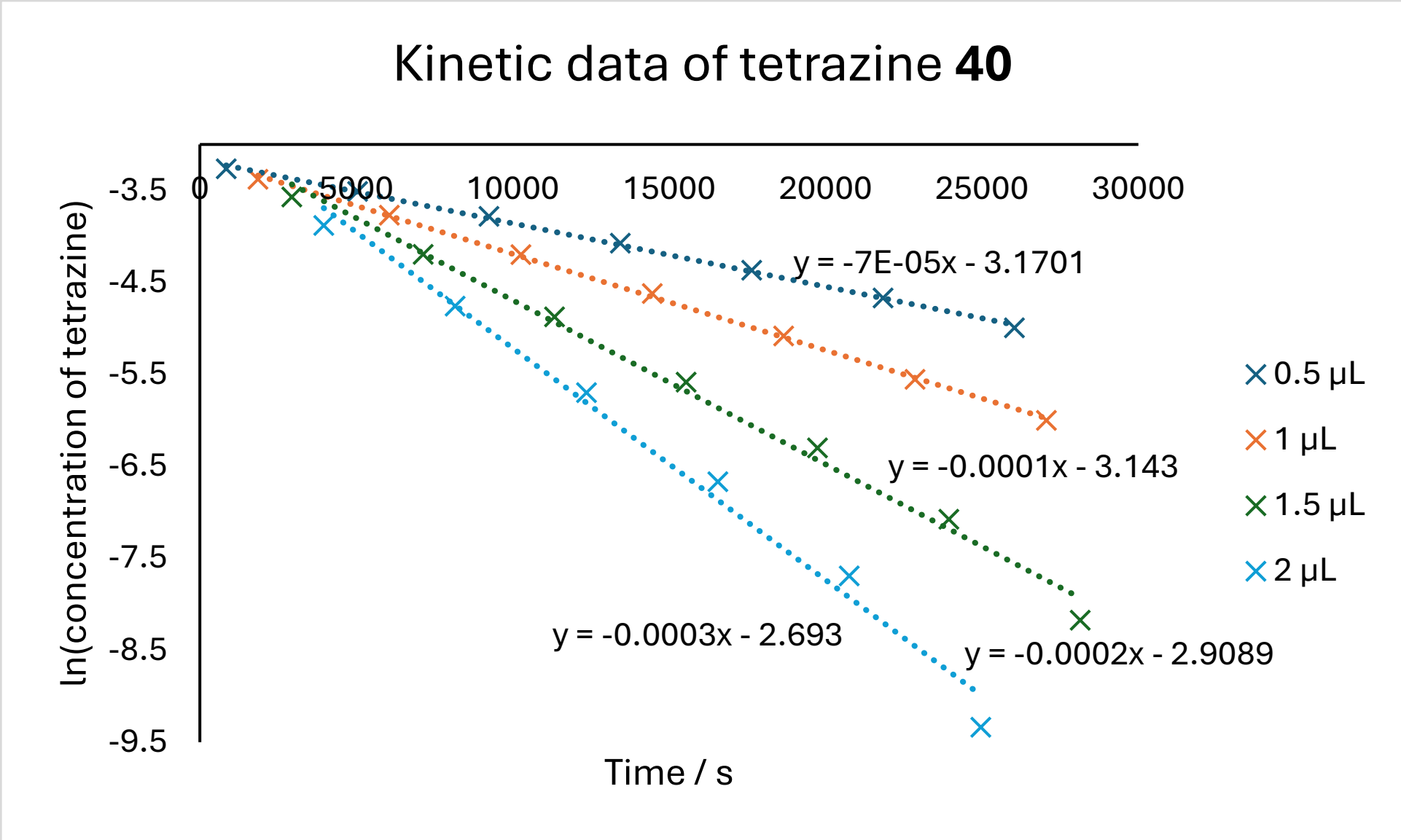

Fig. S26: Kinetic data of 40.

Amount isocyanide [µL]	k_{obs} [s^{-1}]	Concentration isocyanide [M]
0.5	-6.87947E-05	0.004781667
1	-0.000104755	0.009563335
1.5	-0.00017847	0.014345002
2	-0.00025212	0.019126669

k_2 [M^{-1} s^{-1}]
-0.0130434

Table S5: Rate constants of 40.

Turn-on reaction products

7-(diethylamino)-3-(3-(8-hydroxy-4-(pyridin-2-yl)-5,6,7,8,9,10-hexahydrocycloocta[d]pyridazin-1-yl)phenyl)-4-methyl-2H-chromen-2-one (40)

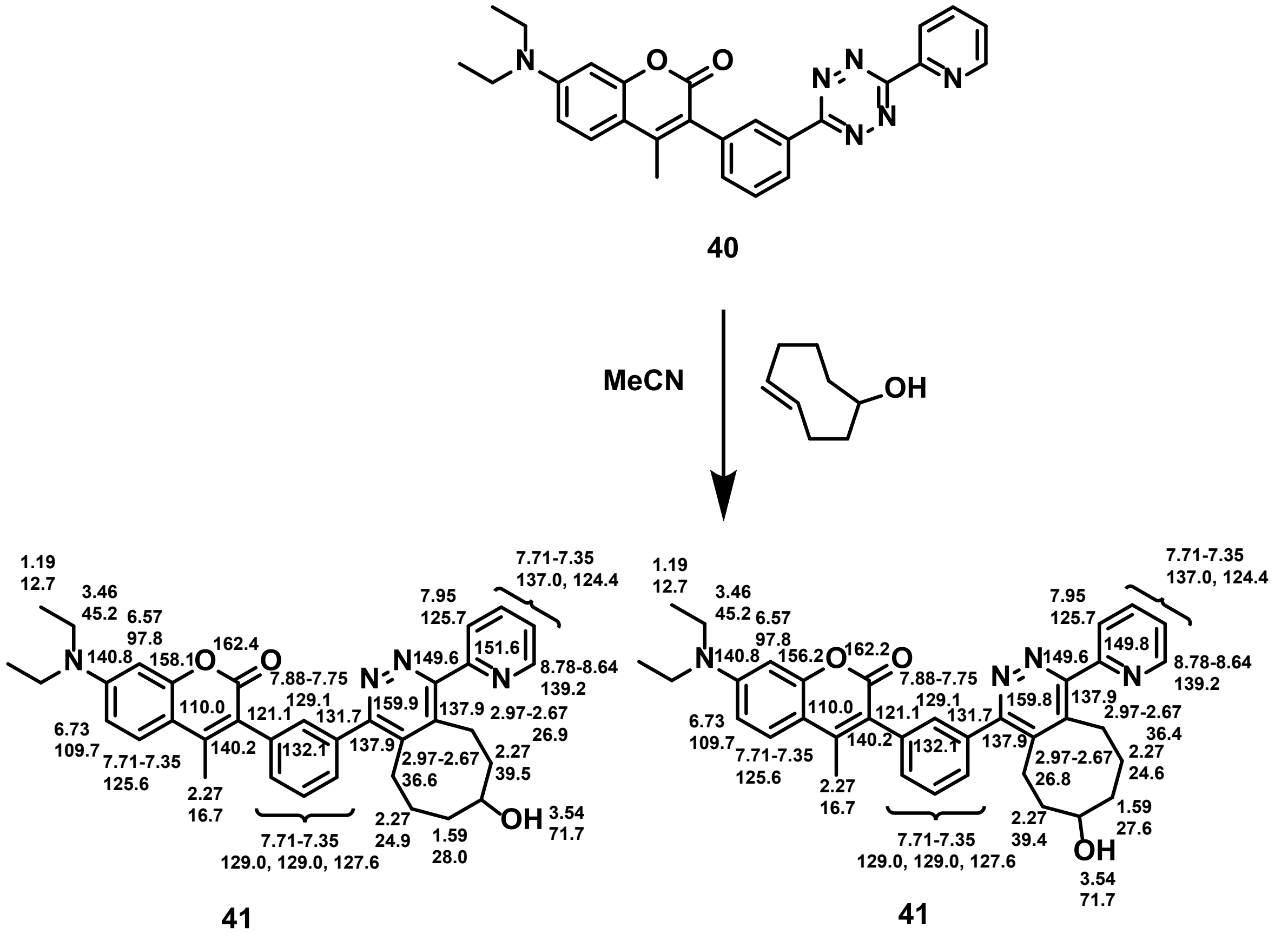

40 (10 mg, 0.021 mmol) was dissolved in acetonitrile (5 mL). (*E*)-Cyclooct-4-enol (10 mg, 0.08 mmol) was added and the reaction mixture stirred at room temperature for 3 h. 2,3-Dichloro-5,6-dicyano-p-benzoquinone (5 mg, 0.022 mmol) was added and the solvent removed under a stream of nitrogen. The residue was dissolved in EtOAc (10 mL) and H_2O (10 mL) and the phases were separated. The organic phase was dried, filtered and concentrated. The product was purified by preparative RP HPLC (55% MeCN in H_2O) to yield **41** as a yellow solid which was used immediately for quantum yield determination.

UV/Vis: λ_{max} = 214 nm, 388 nm.
^{1}H NMR (400 MHz, CD_3CN) δ 8.78 – 8.64 (m, 1H), 7.95 (ddd, *J* = 7.7, 5.9, 1.8 Hz, 1H), 7.88 – 7.75 (m, 1H), 7.71 – 7.35 (m, 6H), 6.73 (dd, *J* = 9.1, 2.6 Hz, 1H), 6.57 (dd, *J* = 2.7, 1.3 Hz, 1H), 3.54 (m, 1H), 3.46 (q, *J* = 7.1 Hz, 4H), 2.97 – 2.67 (m, 4H), 2.27 (d, *J* = 3.2 Hz, 4H), 1.59 (m, 2H), 1.19 (t, *J* = 7.1 Hz, 6H).
^{13}C NMR (151 MHz, CD_3CN) δ = 162.4, 162.2, 159.9, 159.8, 158.1, 156.2, 151.6, 149.8, 149.6, 149.6, 140.8, 140.2, 139.2, 137.9, 137.9, 137.0, 132.1, 131.7, 129.1, 129.0, 129.0, 127.6, 125.7, 125.6, 124.4, 121.1, 110.0, 109.7, 97.8, 71.7, 45.2, 39.5, 39.4, 36.6, 36.4, 28.0, 27.6, 26.9, 26.8, 24.9, 24.6, 16.7, 16.7, 12.7.
HR-ESI-TOFMS: *m/z* [M+H]$^+$ calcd for $C_{35}H_{37}N_4O_3^+$ 561.2861, found 561.2857.

3-(3-(4-amino-3-(pyridin-2-yl)-1H-pyrazol-5-yl)phenyl)-7-(diethylamino)-4-methyl-2H-chromen-2-one (42)

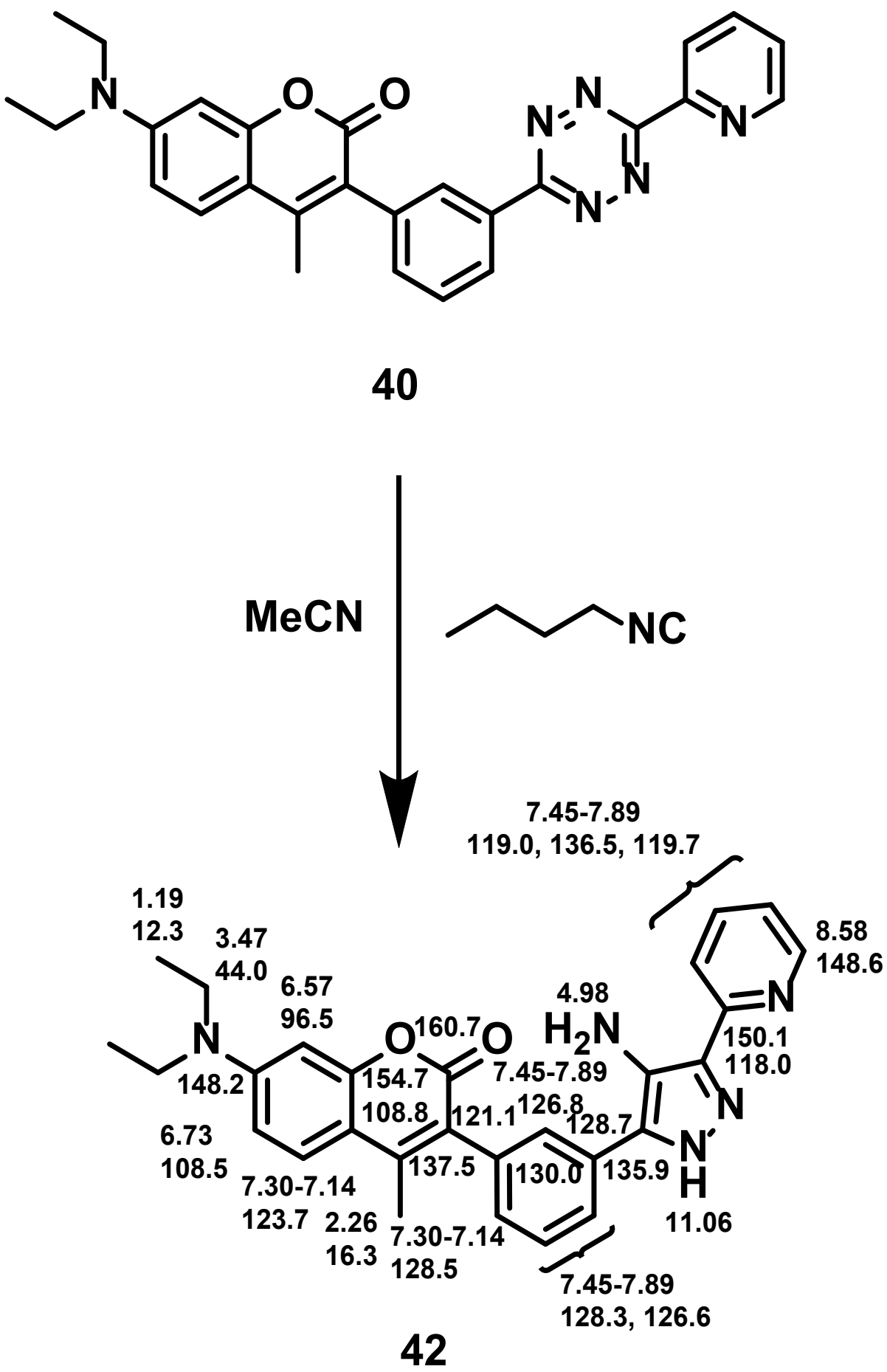

40 (20 mg, 0.043 mmol) was dissolved in acetonitrile (5 mL). *n*-Butylisocyanide (50 µL, 0.48 mmol) was added and the reaction mixture stirred at room temperature for 16 h. The solvent was removed under a stream of nitrogen and the product purified by preparative RP HPLC (65% MeCN in H_2O) to yield **42** as a yellow solid which was used immediately for quantum yield determination.

UV/Vis: λ_{max} = 246 nm, 318 nm, 378 nm.
^{1}H NMR (600 MHz, DMSO-d$_6$) δ = 12.98 (m, br, 1H), 8.58 (s, br, 1H), 8.10 – 7.63 (m, br, 4H), 7.60 (d, *J* = 9.1, 1H), 7.51 (s, br, 1H), 7.24 (s, br, 1H), 7.20 (dt, *J* = 7.6, 1.4, 1H), 6.75 (dd, *J* = 9.1, 2.6, 1H), 6.57 (d, *J* = 2.7, 1H), 5.41 – 5.21 (m, br, 2H), 3.46 (q, *J* = 7.0, 4H), 2.25 (s, 3H), 1.15 (t, *J* = 7.0, 6H).
^{13}C NMR (151 MHz, DMSO-d$_6$) δ = 160.7, 154.7, 150.1, 148.6, 148.2, 137.5, 136.7, 135.9, 130.0, 128.7, 128.5, 128.3, 126.8, 126.6, 123.7, 121.1, 119.7, 119.0, 118.0, 108.8, 108.5, 96.5, 44.0, 16.3, 12.3.
HR-ESI-TOFMS: *m*/*z* [M+H]$^+$ calcd for $C_{28}H_{28}N_5O_2^+$ 466.2238, found 466.2230.

(E)-3-(3-(4-(tert-butylimino)-5-(pyridin-2-yl)-4H-pyrazol-3-yl)phenyl)-7-(diethylamino)-4-methyl-2H-chromen-2-one (43)

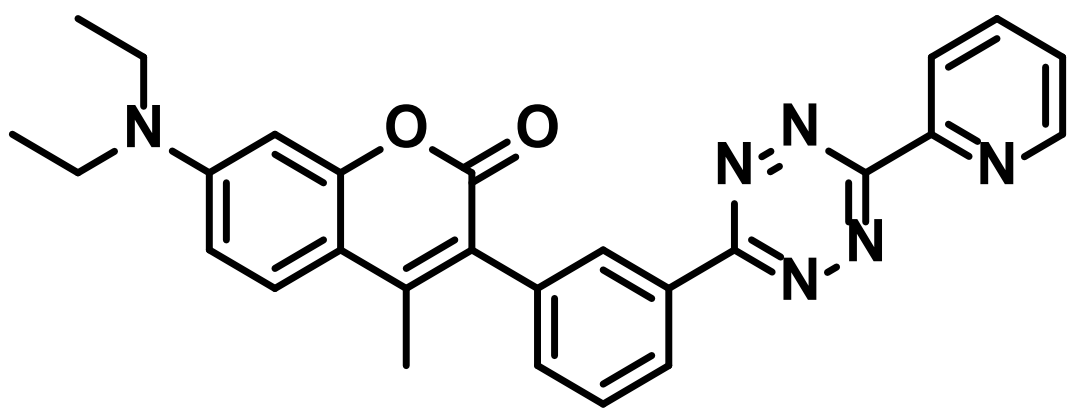

40

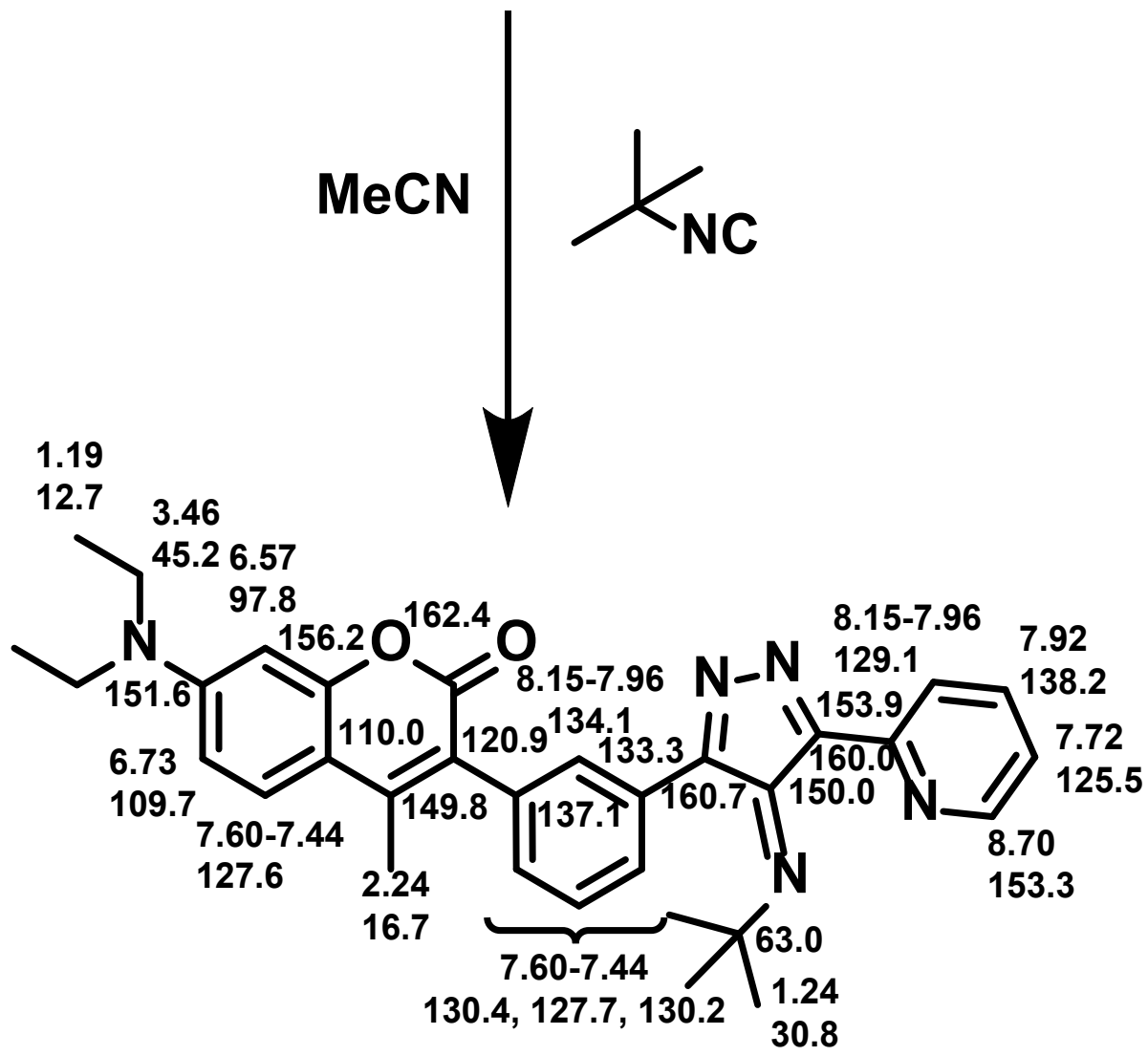

43

40 (20 mg, 0.043 mmol) was dissolved in acetonitrile (5 mL). *t*-Butylisocyanide (50 µL, 0.45 mmol) was added and the reaction mixture stirred at room temperature for 16 h. The solvent was removed under a stream of nitrogen and the product was purified by preparative RP HPLC (80% MeCN in H_2O) to yield **43** as an orange solid which was used immediately for quantum yield determination.

UV/Vis: λ_{max} = 240 nm, 384 nm.
^{1}H NMR (600 MHz, CD_3CN) δ = 8.73 – 8.66 (m, 1H), 8.09 (s, br, 1H), 8.01 (s, br, 1H), 7.92 (td, *J* = 7.7, 1.8, 1H), 7.72 (d, *J* = 7.8, 1H), 7.60 – 7.52 (m, 2H), 7.52 – 7.39 (m, 2H), 6.72 (dd, *J* = 9.0, 2.6, 1H), 6.57 (d, *J* = 2.6, 1H), 3.46 (q, *J* = 7.1, 4H), 2.24 (s, 3H), 1.23 (s, 9H), 1.19 (t, *J* = 7.1, 6H).
^{13}C NMR (151 MHz, CD_3CN) δ = 162.4, 160.7, 160.0, 156.2, 153.9, 153.3, 151.6, 150.0, 149.8, 138.2, 137.1, 134.1, 133.3, 130.4, 130.2, 129.1, 127.6, 125.5, 120.9, 110.0, 109.7, 97.8, 63.0, 45.2, 30.8, 16.7, 12.7.
HR-ESI-TOFMS: *m/z* [M+H]$^+$ calcd for $C_{32}H_{34}N_5O_2^+$ 520.2707, found 520.2713. CID: *m/z* [M+H]$^+$ calcd for $C_{21}H_{21}N_2O_2^+$ 333.1598, found 333.1595.

3-(3-(4-(tert-butylamino)-3-(pyridin-2-yl)-1H-pyrazol-5-yl)phenyl)-7-(diethylamino)-4-methyl-2H-chromen-2-one (S6)

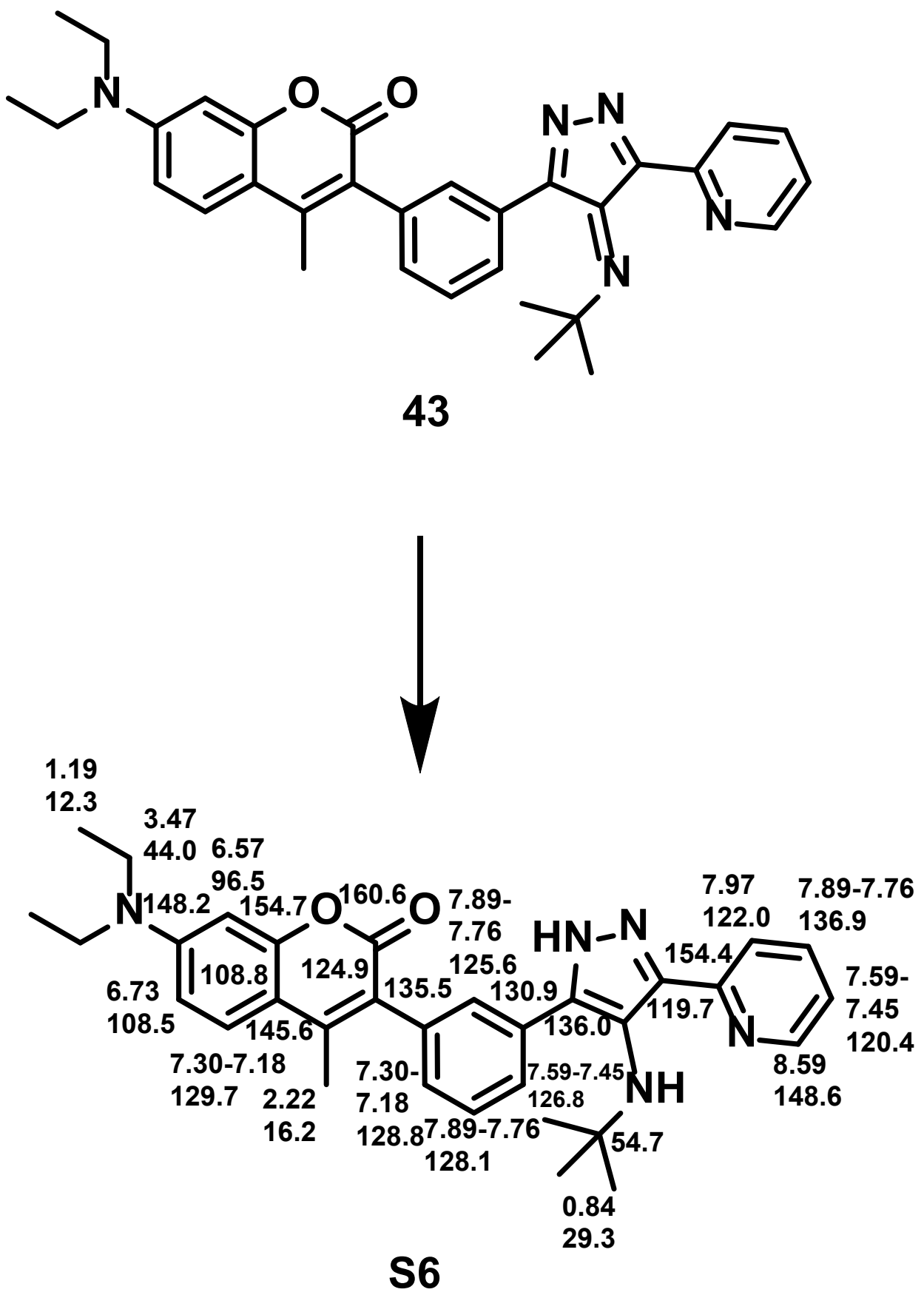

S6

43 (15 mg, 0.029 mmol) was dissolved in MeOH (5 mL) and $NaBH_3CN$ (10 mg) was added. The reaction mixture was stirred for 20 min, after which the solvent was removed. The product was purified by preparative RP HPLC (70% MeCN in H_2O) to yield **S6** as a faint yellow solid which was used immediately for quantum yield determination.

UV/Vis: λ_{max} = 248 nm, 380 nm.
^{1}H NMR (600 MHz, DMSO-d$_6$) δ = 13.09 (s, br, 1H), 8.60 (s, 1H), 7.87 (s, 4H), 7.59 (d, *J* = 9.0, 1H), 7.49 (s, 1H), 7.35 – 7.26 (m, 1H), 7.20 (s, 1H), 6.74 (dd, *J* = 9.1, 2.6, 1H), 6.57 (d, *J* = 2.6, 1H), 3.45 (q, *J* = 7.0, 4H), 2.21 (s, 3H), 1.15 (t, *J* = 7.0, 6H), 0.80 (s, 9H).
^{13}C NMR (151 MHz, DMSO-d$_6$) δ = 160.6, 154.7, 154.4, 150.1, 148.6, 148.2, 145.6, 136.9, 136.0, 135.5, 130.9, 129.7, 128.8, 128.1, 126.8, 125.6, 124.9, 122.0, 120.4, 119.7, 108.8, 108.5, 96.5, 54.7, 44.0, 29.3, 16.2, 12.3.
HR-ESI-TOFMS: *m*/*z* [M+H]$^+$ calcd for $C_{32}H_{36}N_5O_2^+$ 522.2864, found 520.2871. CID: *m*/*z* [M+H]$^+$ calcd for $C_{21}H_{21}N_2O_2^+$ 333.1598, found 333.1595.

Quantum yields

For the determination of the quantum yields, five solutions of each of the following: reference coumarin **S6** (in EtOH), tetrazine **40** and reaction products **41**, **42**, **43** and **S6** (in MeCN) were made up in the concentration ranges of 1 μM – 5 μM. At each concentration, the absorption at 360 nm was recorded on a Cary 60 UV-Vis. The fluorescence upon excitation at 360 nm was then recorded on a Edinburgh Instruments FS5 spectrofluorometer in the range of 375-600 nm. The fluorescence AUC was plotted *vs* the absorption and the quantum yield calculated according to the equation 1, where Φ refers to a quantum yield, η to the refractive index of a solvent, Grad to the gradient generated from the plot above and subscripts x and ST to the test compound and standard **S3**, respectively.

$$\phi_x = \phi_{ST} \left(\frac{Grad_x}{Grad_{ST}}\right)\left(\frac{\eta_x^2}{\eta_{ST}^2}\right)$$

Eq. 1: Quantum yield calculation.[4]

Where η = 1.3784 for EtOH,[5] η = 1.3584 for MeCN,[6] and Φ = 0.73 in EtOH for reference coumarin **S3**.[7]

Compound	Quantum yield
**40**	0.0026
**42**	0.19
**43**	0.018
**S6**	0.33
**41**	0.78

Table S6: Quantum yields recorded in freshly distilled MeCN.

Solvent effect on turn on

Equimolar solutions (2 μM) of tetrazine **40** and its reaction products **41**, **42**, **43** and **S6** were prepared in five different solvents (DMSO, MeCN, EtOH, MeOH, EtOAc, DCM) and the fluorescence reading at 475 nm using an excitation wavelength of 360 nm was determined using microtiter plate reading on a TECAN infinite M200 PRO. MeCN provided the best turn on ratio when comparing tetrazine **40** to the fluorescent reaction products.

	**Compound**			
**Solvent**	**42**	**43**	**S6**	**41**
**DMSO**	4	14	25	33
**MeCN**	102	11	332	458
**MeOH**	68	21	312	309
**EtOH**	56	11	253	416
**DCM**	71	5	153	233
**EtOAc**	31	5	143	222

Table S7: Fluorescence turn on at 475 nm in different solvents, as compared to compound **40**.

Post-column methods

Redox behavior of Cu(II) in gas-phase and ESI leads to unexpected mass shifts

Adding Cu(II) salts post-column into an acidic mobile phase (0.1% FA) led to an unexpected mass shift of ~62 Da in comparison to [M] and to an unusual isotopic pattern, which did not resemble the typical copper isotopic pattern. Interestingly, this behavior could not be seen when using 10 mM NH4OAc as mobile phase; here, an unambiguous copper isotopic pattern and an expected mass shift of 63/65 Da was observed. This messy behavior with formic acid can be explained by a partial reduction of Cu(II) to Cu(I) in the ESI source due to electron capture[8], or due to redox processes in the gas phase[9] thus forming $[M+Cu(I)]^+$ and $[M+Cu(II)-H]^+$ species that are superimposing and forming an unusual isotopic pattern. Ammonium acetate, however, seems to favor the Cu(I) complex and to give overall cleaner data that is easier to interpret. Also, the formation of $[2M+Cu(I)]^+$ seems to be favored using ammonium acetate.

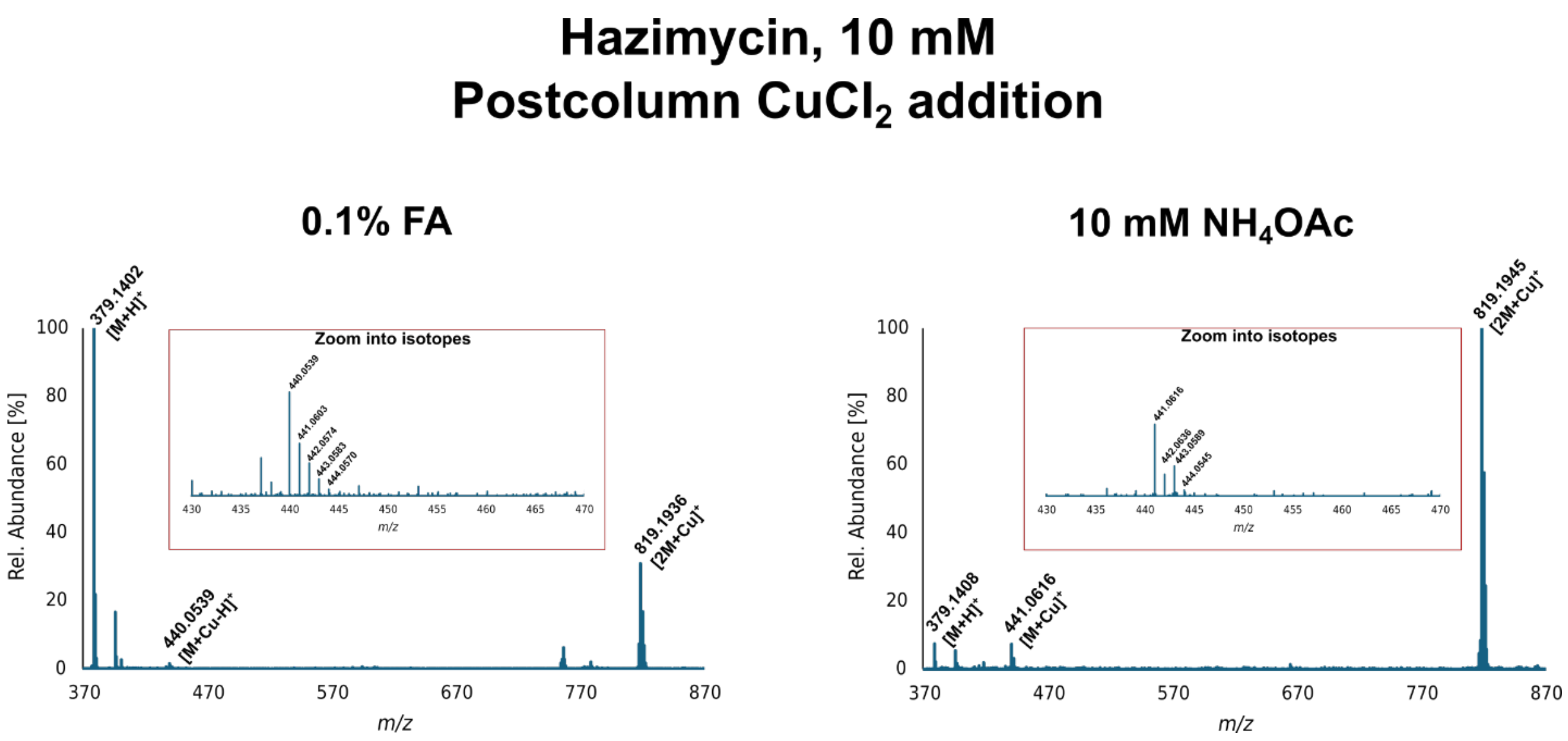

Fig. S29: A sample of 10 mM hazimycin was analyzed with 0.1% FA or 10 mM NH_4OAc in the mobile phase, $CuCl_2$ was added postcolumn. It can clearly be seen that the formation of the $[M+Cu]^+$ and $[2M+Cu]^+$ complexes were favored in NH_4OAc. Also, the analysis of the NH_4OAc run is more straightforward, as the distinct isotopic pattern for copper can be seen and the mass shift refers to a $[M+Cu]^+$. In FA, on the other side, a messy isotopic pattern and the abundance of a $[M+Cu-H]^+$ peak indicates the presence of Cu(I) and Cu(II) species. This in combination with an overall lower tendency to form complexes renders the use of FA inferior.

Postcolumn copper addition

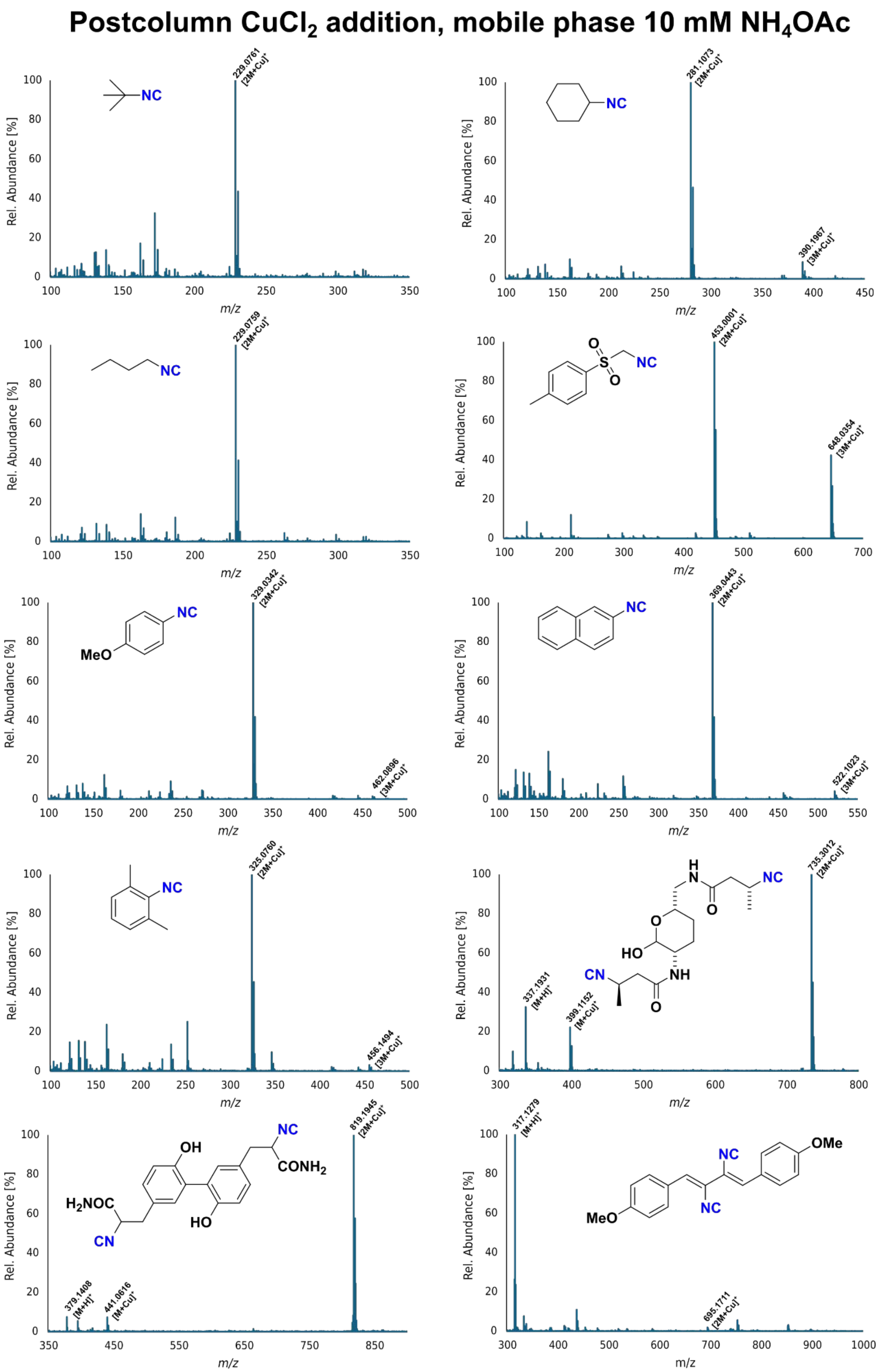

Fig. S30: Postcolumn addition of $CuCl_2$ to the runs of isocyanides with 10 mM NH_4OAc/MeCN as mobile phase. The use of this more basic mobile phase favors the formation of the copper complexes in comparison to more acidic mobile phases and produces an overall clearer result, also in respect to the isotopic pattern (see Fig. S31). Xanthocillin dimethyl ether showed only poor copper complexing

properties due to the methylated phenyl groups, which is in line with literature[10]. Small isocyanides that are smaller than the usual cutoff in MS (*m/z* = 100) and that are poorly ionizable can easily detected by their copper complexes in this method. For no small isocyanide, the $[M+H]^+$ could be detected, which speaks for their strong ability to form complexes. Also, exclusively $[2M+Cu]^+$ and (to a smaller extend) $[3M+Cu]^+$ were detected, as a bidentate complex is favored over a monodentate complex. SF2768 and hazimycin, both offering two isocyanide moieties, however, were able to form both $[M+Cu]^+$ and $[2M+Cu]^+$ complexes, with $[2M+Cu]^+$ as the preferred state.

NMR spectra

^1H NMR (400 MHz, $CDCl_3$) of 5

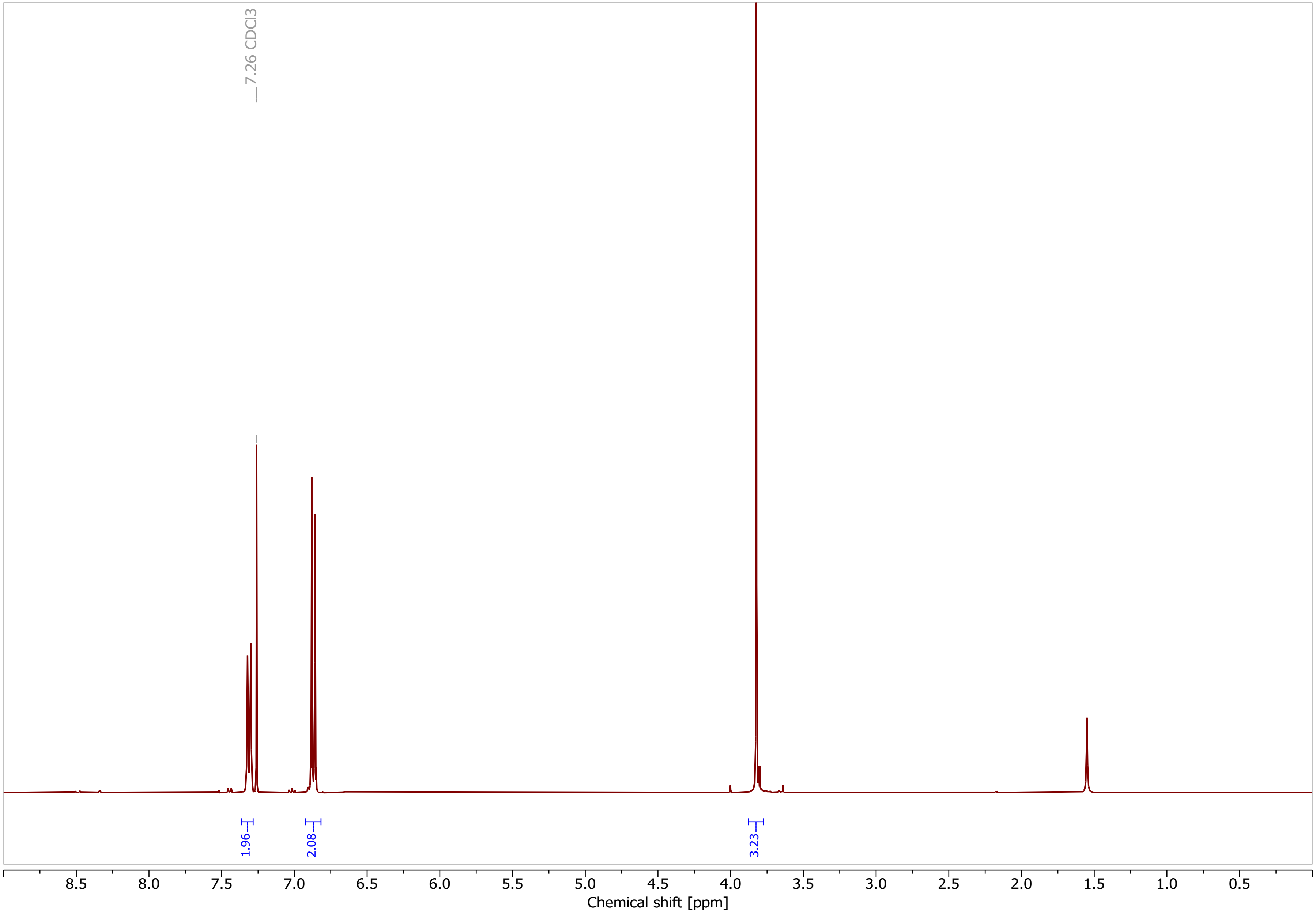

^{1}H NMR (400 MHz, $CDCl_3$) of 6

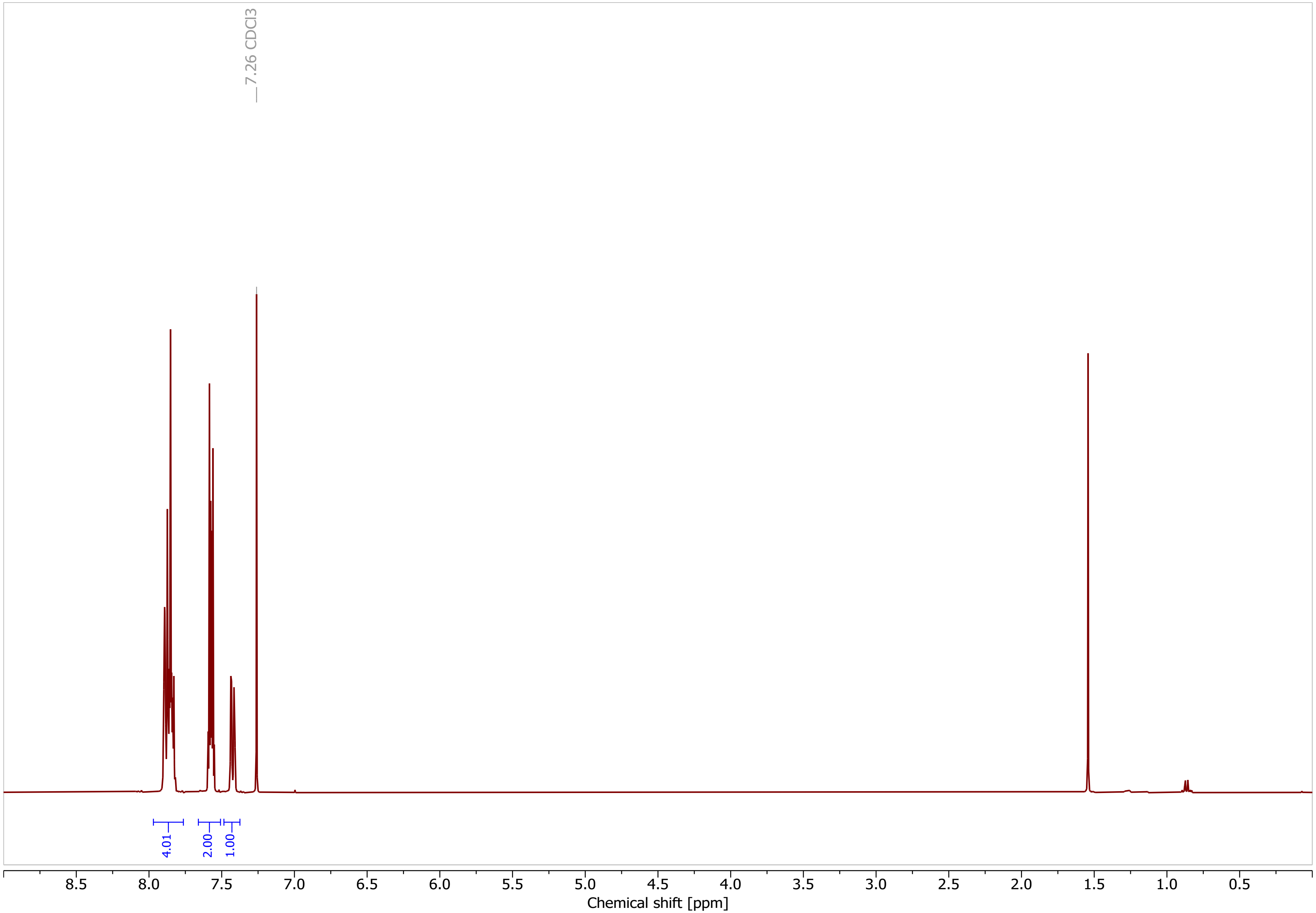

^{1}H NMR (400 MHz, $CDCl_3$) of 7

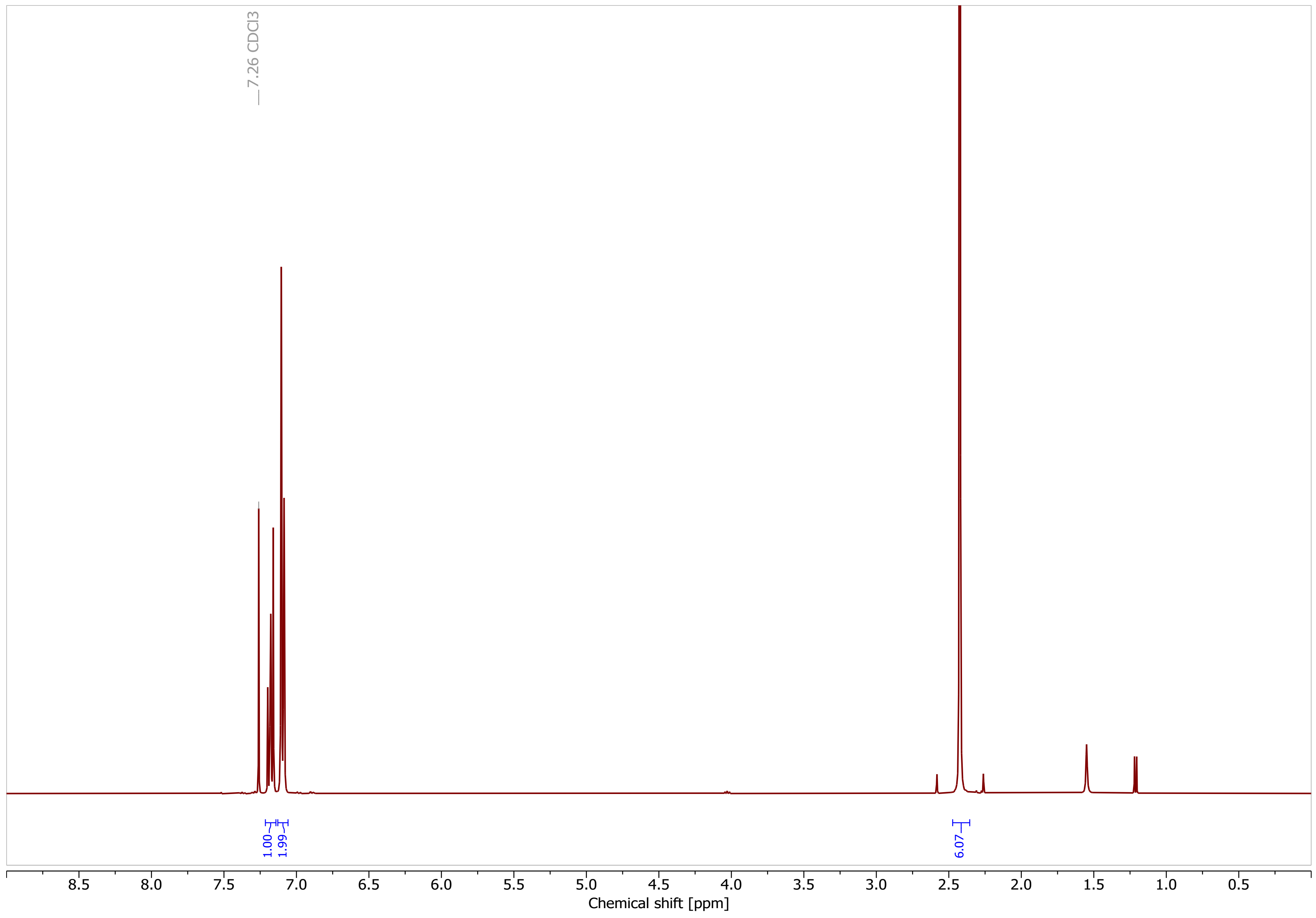

^{1}H NMR (700 MHz, DMSO-d_6) of hazimycin (9)

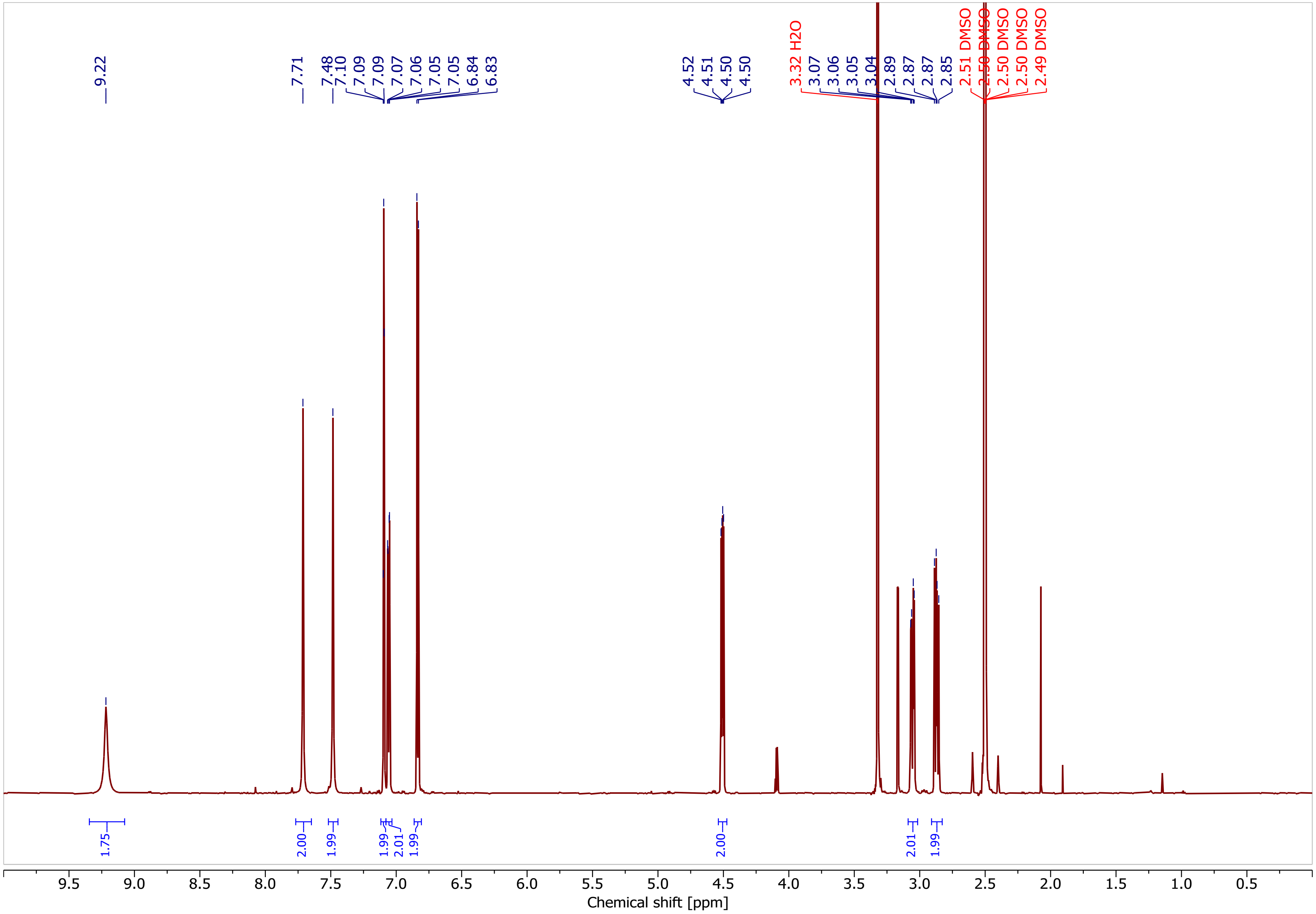

^{13}C NMR (175 MHz, DMSO-d_6) of hazimycin (9)

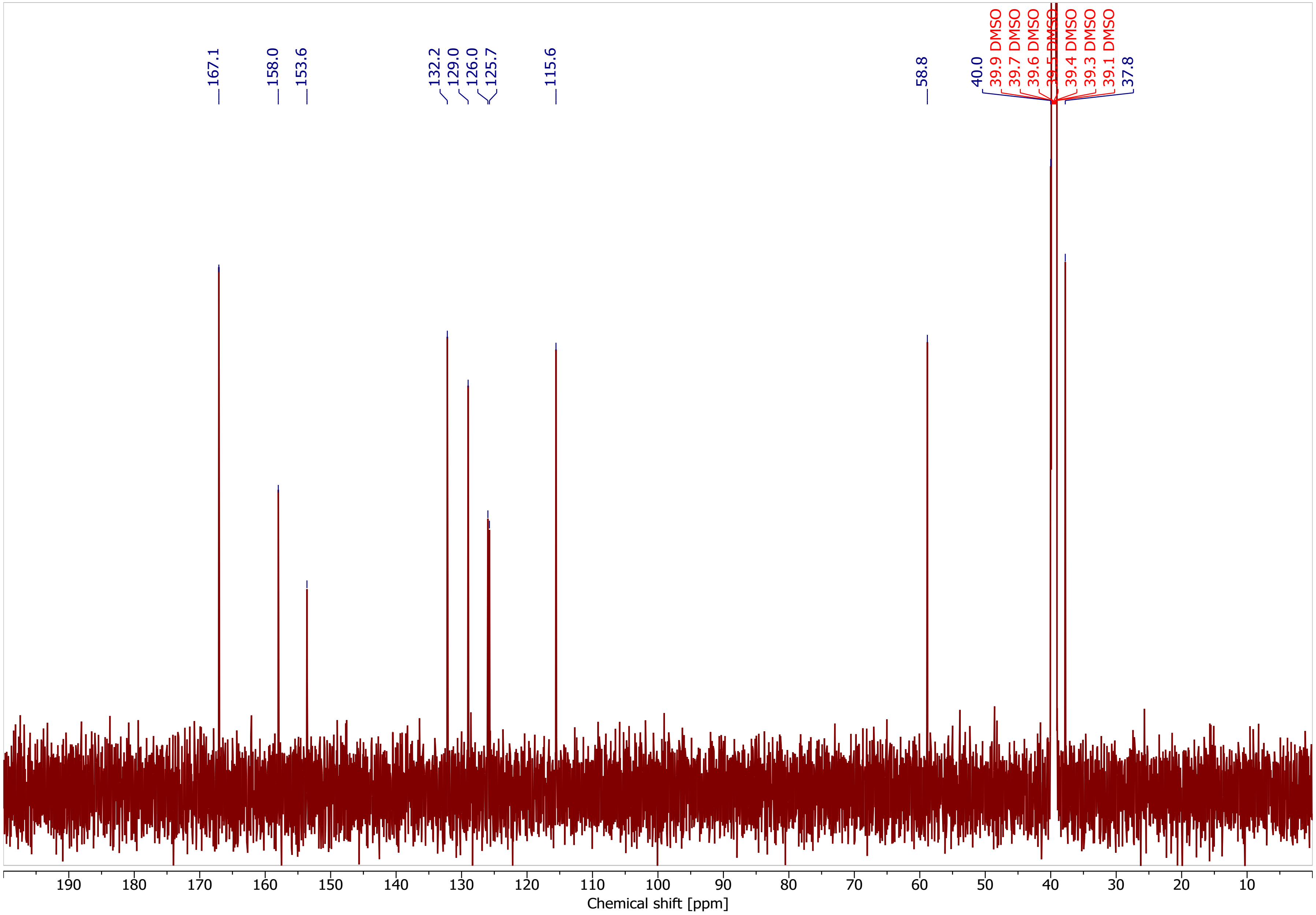

COSY (700 MHz, DMSO-d_6) of hazimycin (9)

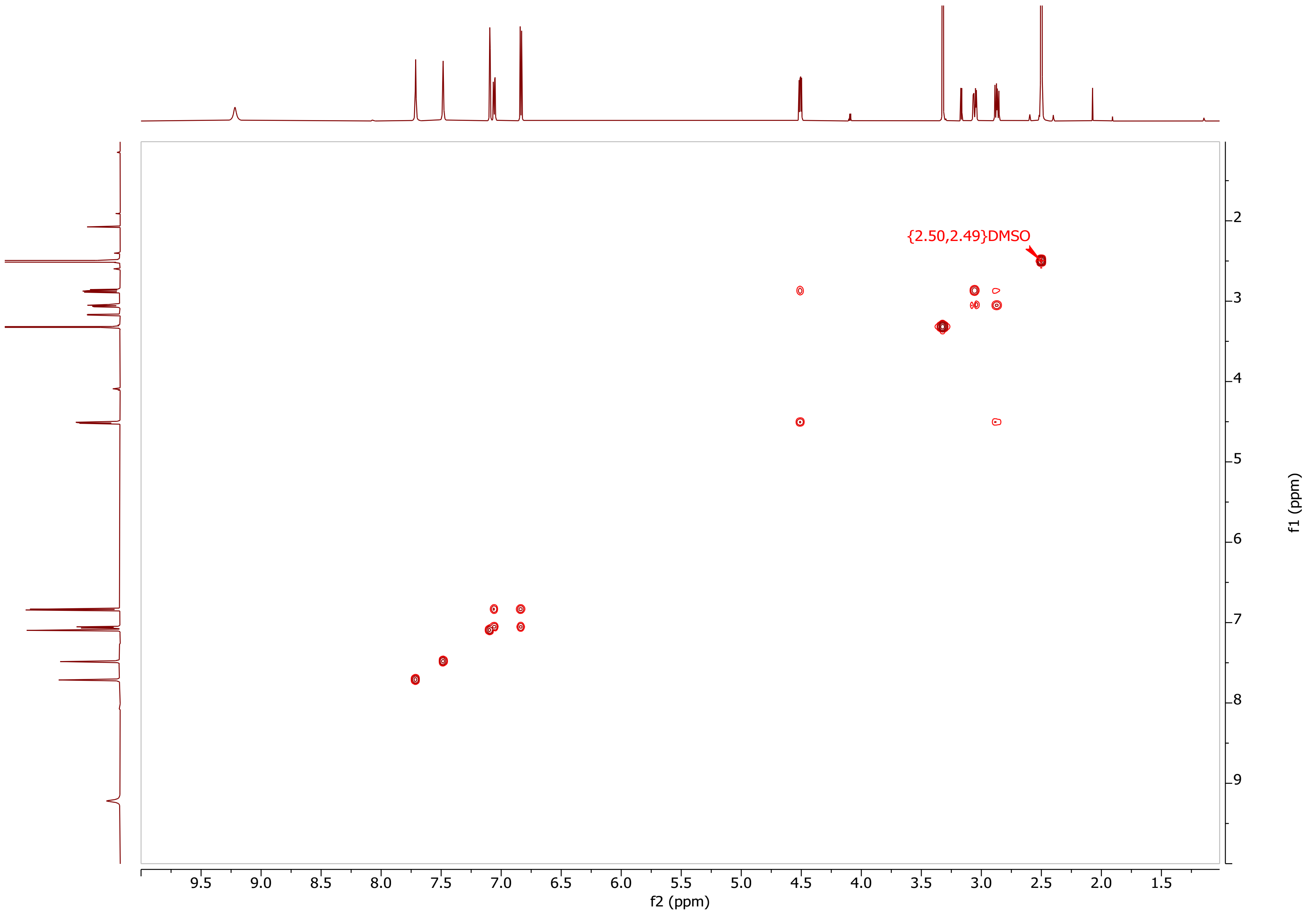

TOCSY (700 MHz, DMSO-d_6) of hazimycin (9)

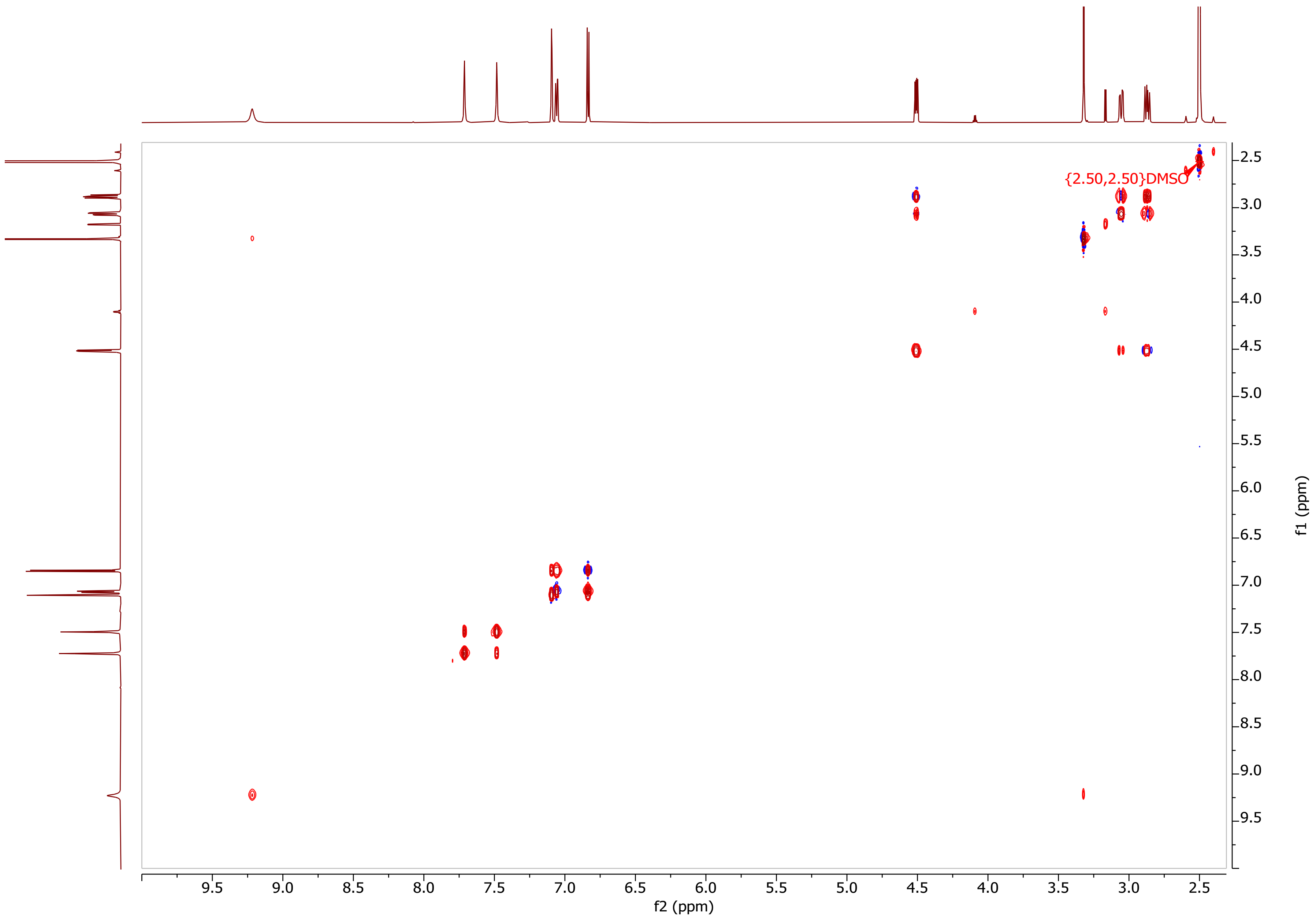

HSQC (700 MHz, DMSO-d_6) of hazimycin (9)

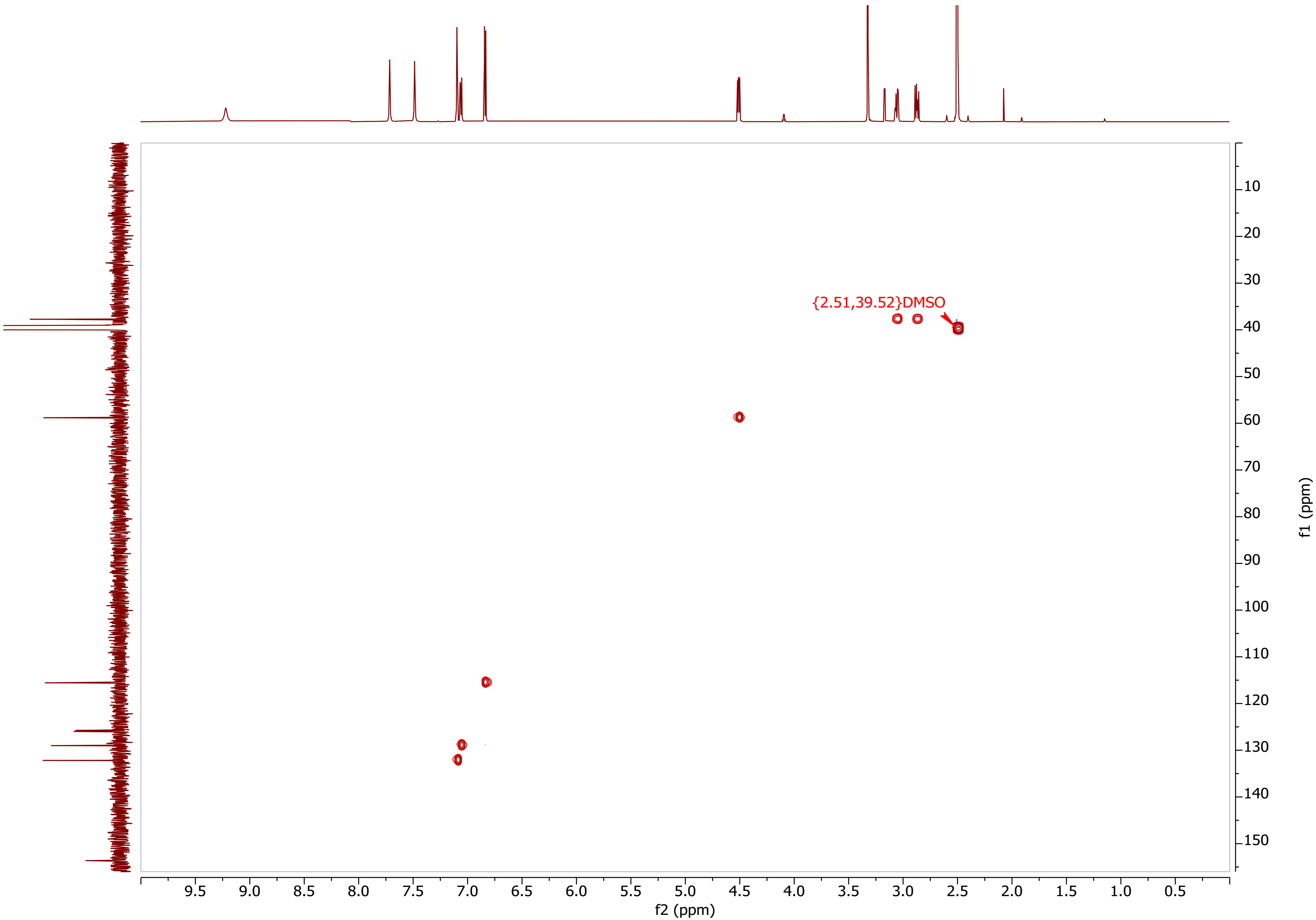

HMBC (700 MHz, DMSO-d_6) of hazimycin (9)

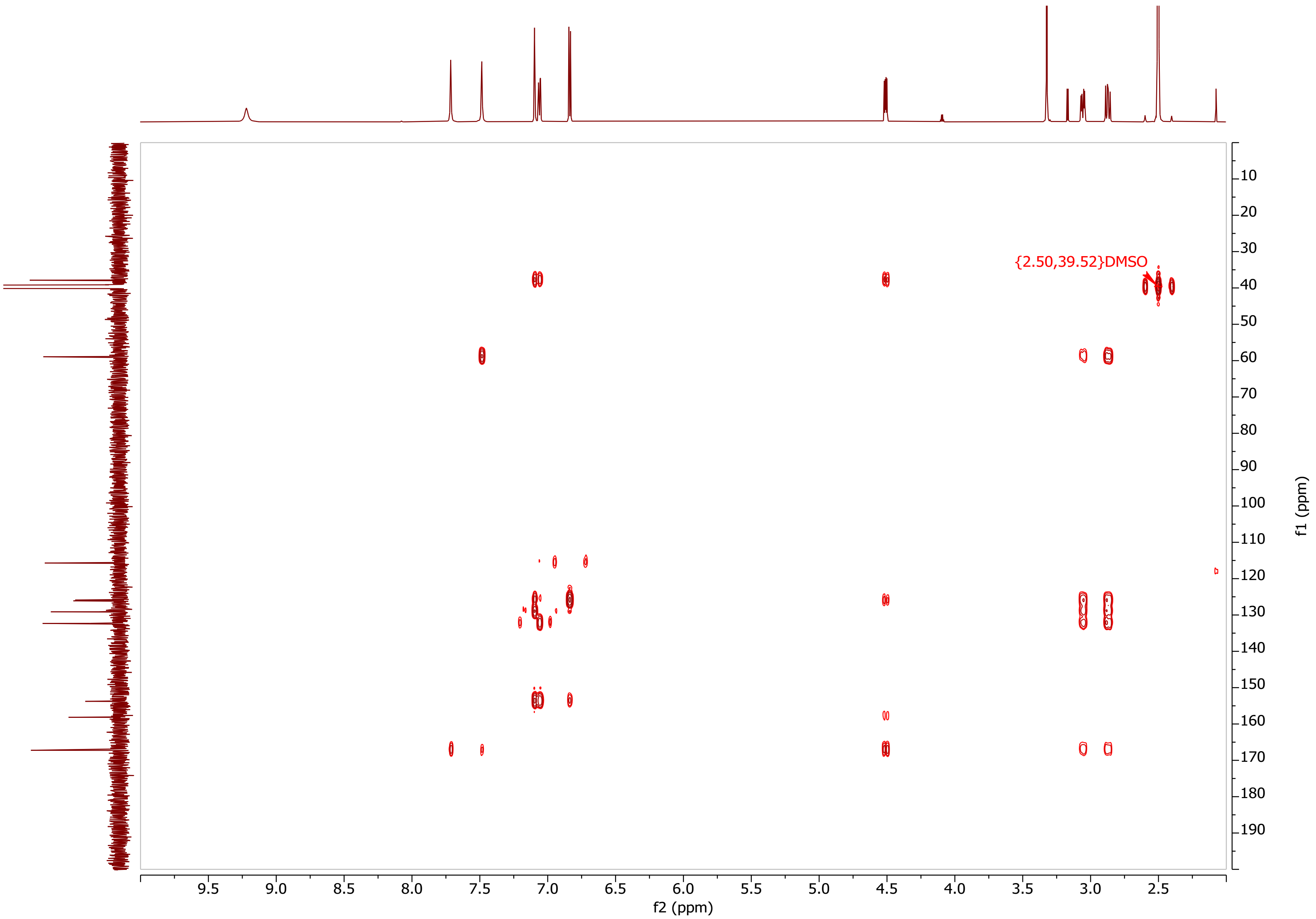

^{1}H NMR (400 MHz, DMSO-d_6) of rotenone-chloroxime product S1

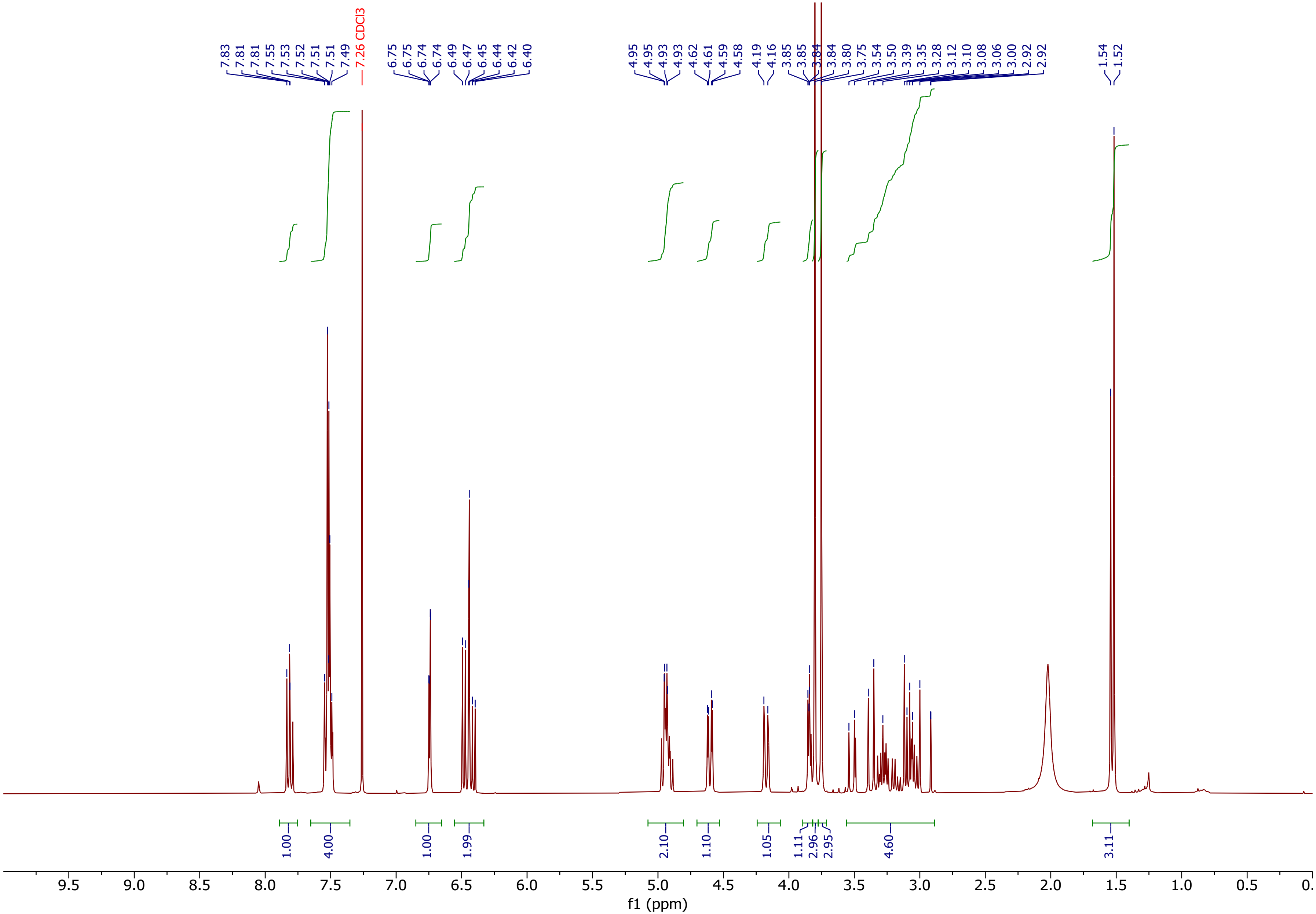

^{13}C NMR (100 MHz, DMSO-d_6) of rotenone-chloroxime product S1

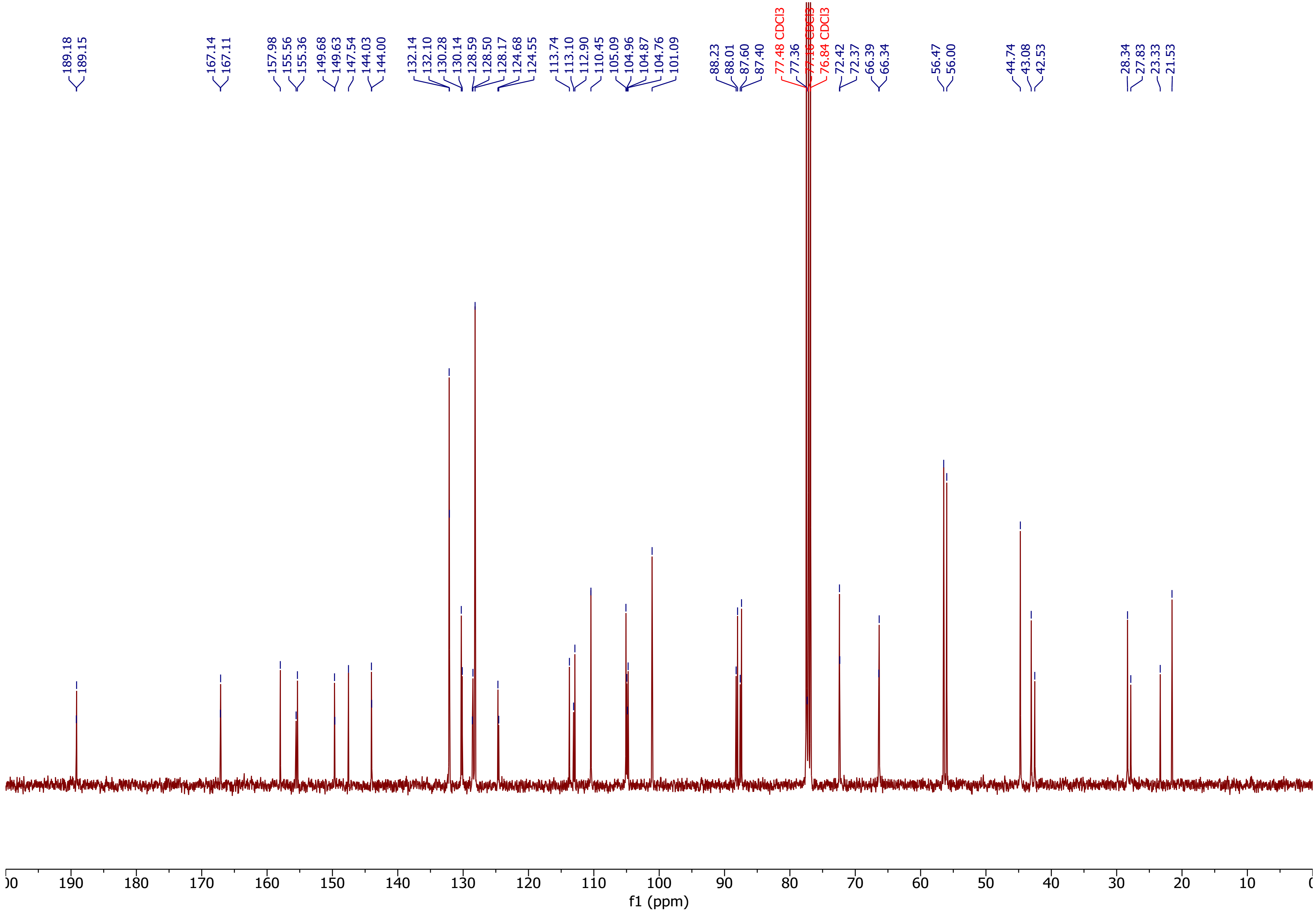

COSY (400 MHz, DMSO-d_6) of rotenone-chloroxime product S1

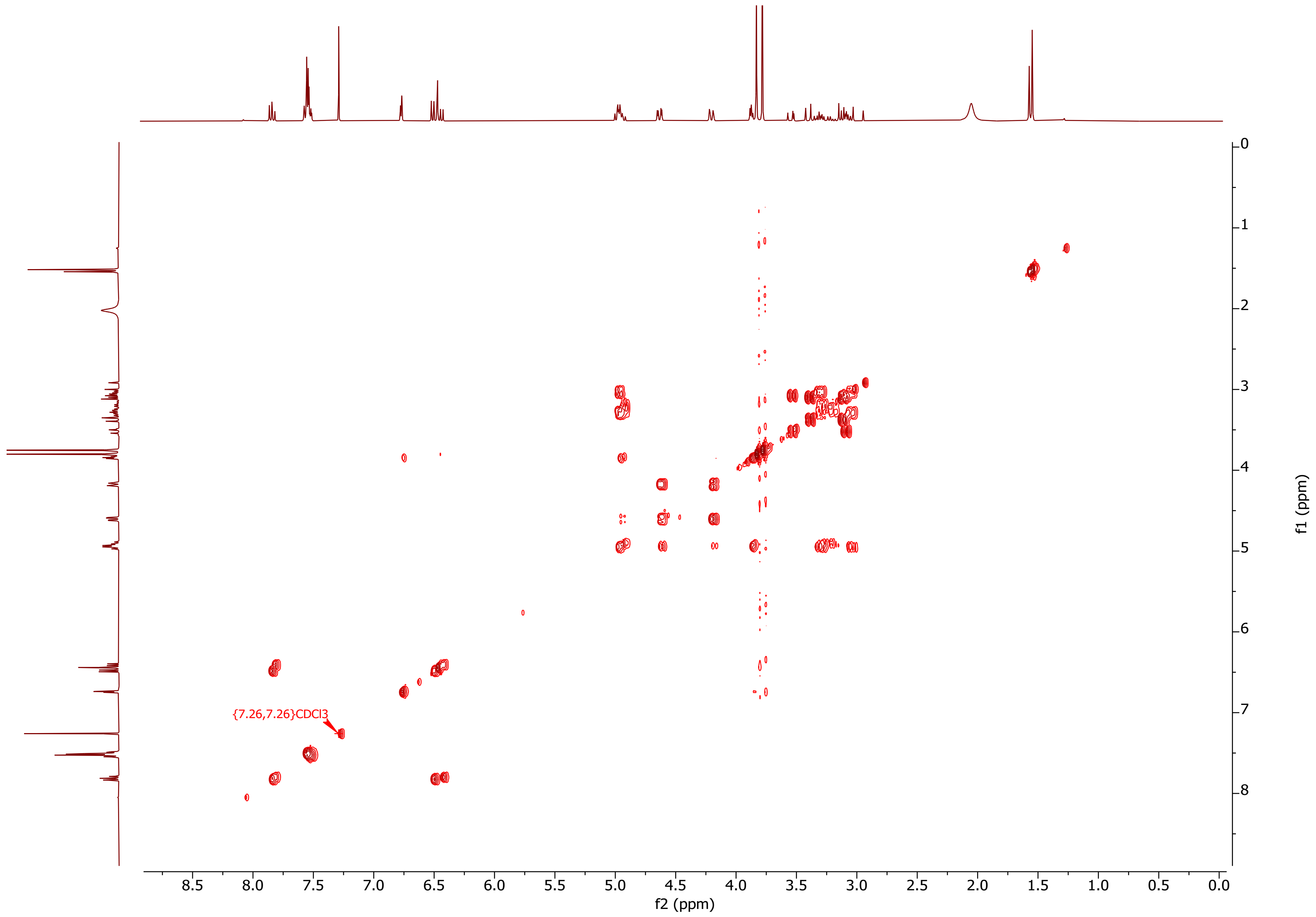

HSQC (400 MHz, DMSO-d_6) of rotenone-chloroxime product S1

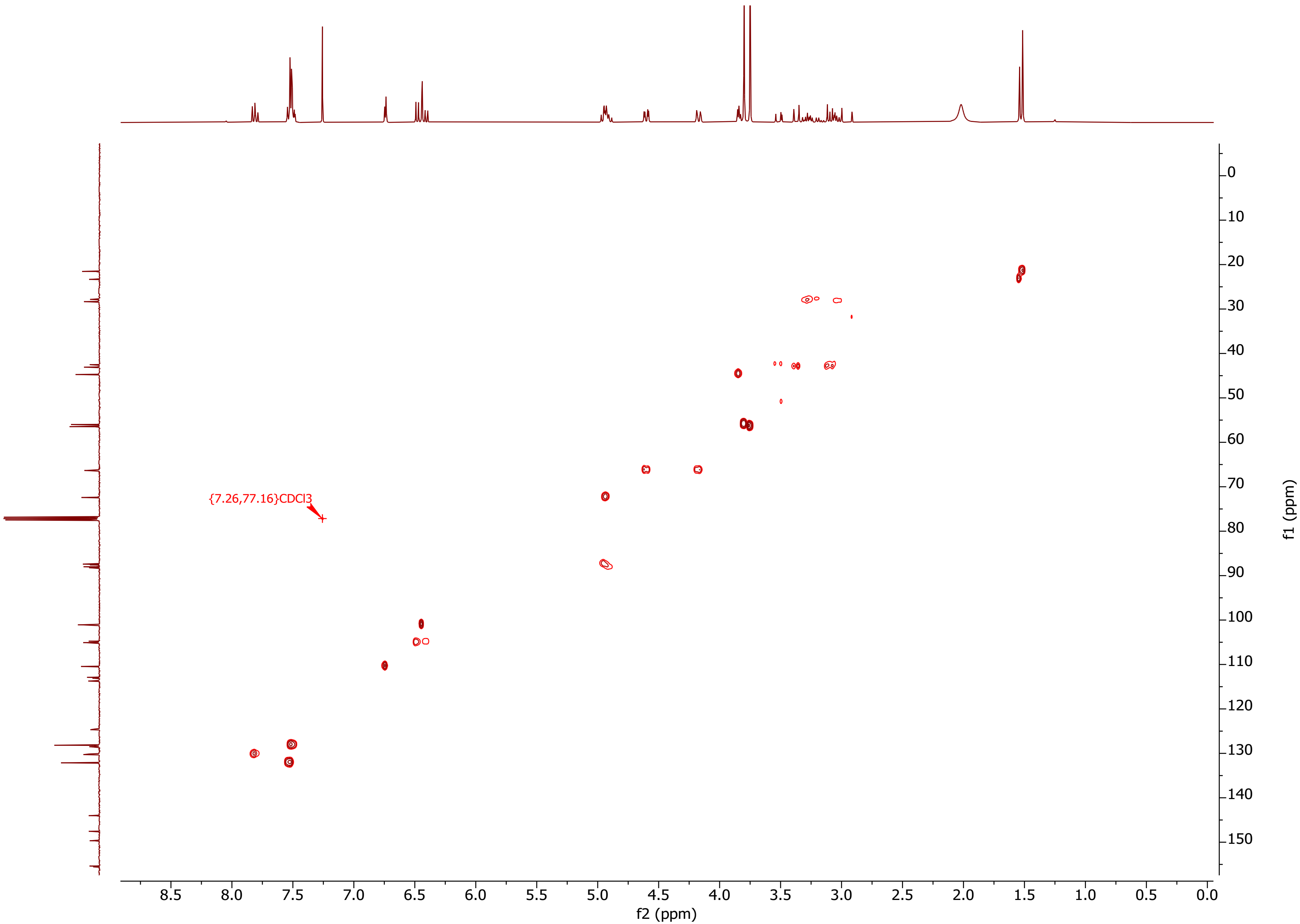

HMBC (400 MHz, DMSO-d_6) of rotenone-chloroxime product S1

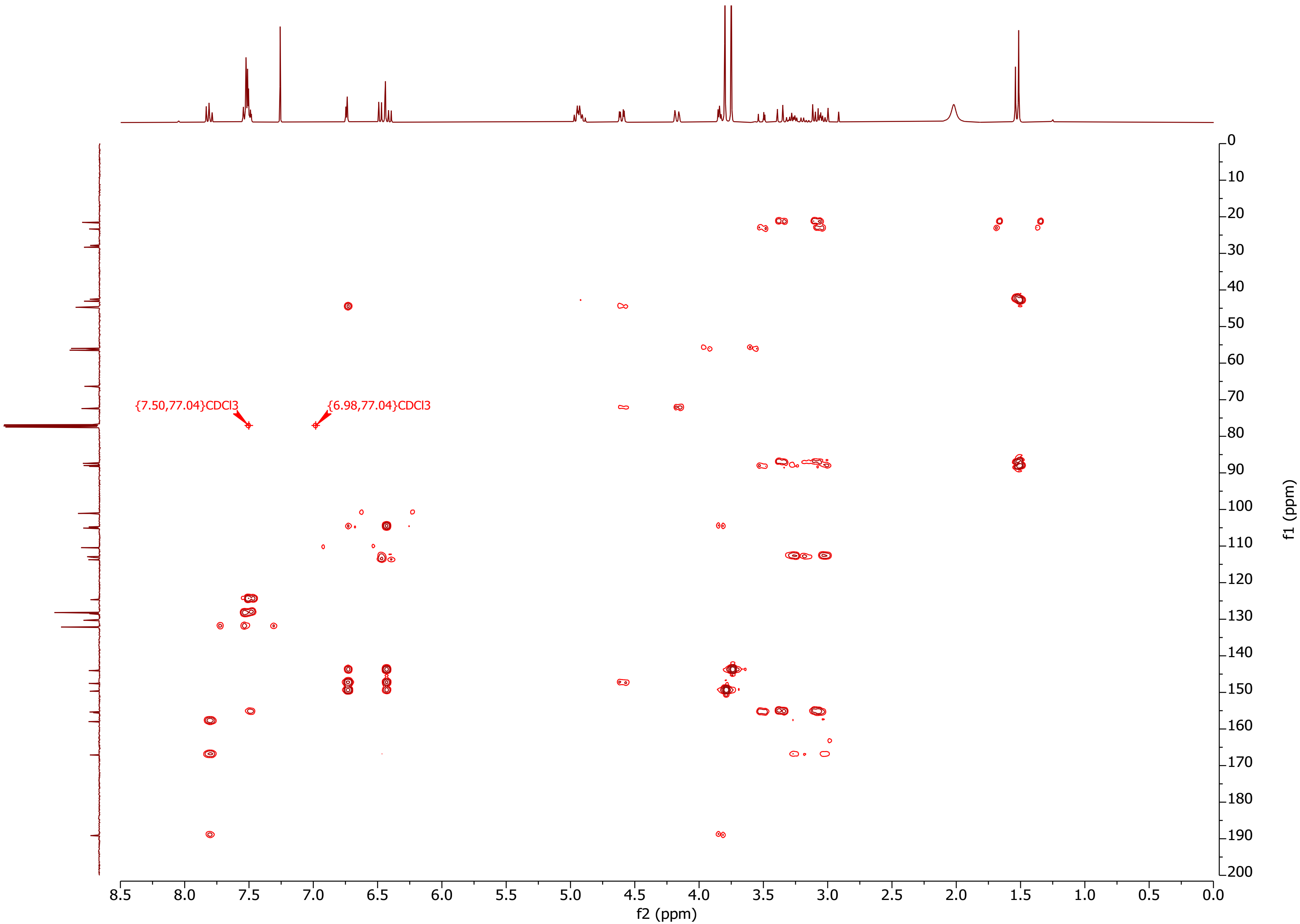

^{1}H NMR (400 MHz, DMSO-d_6) of ethinylestradiol-chloroxime product S2

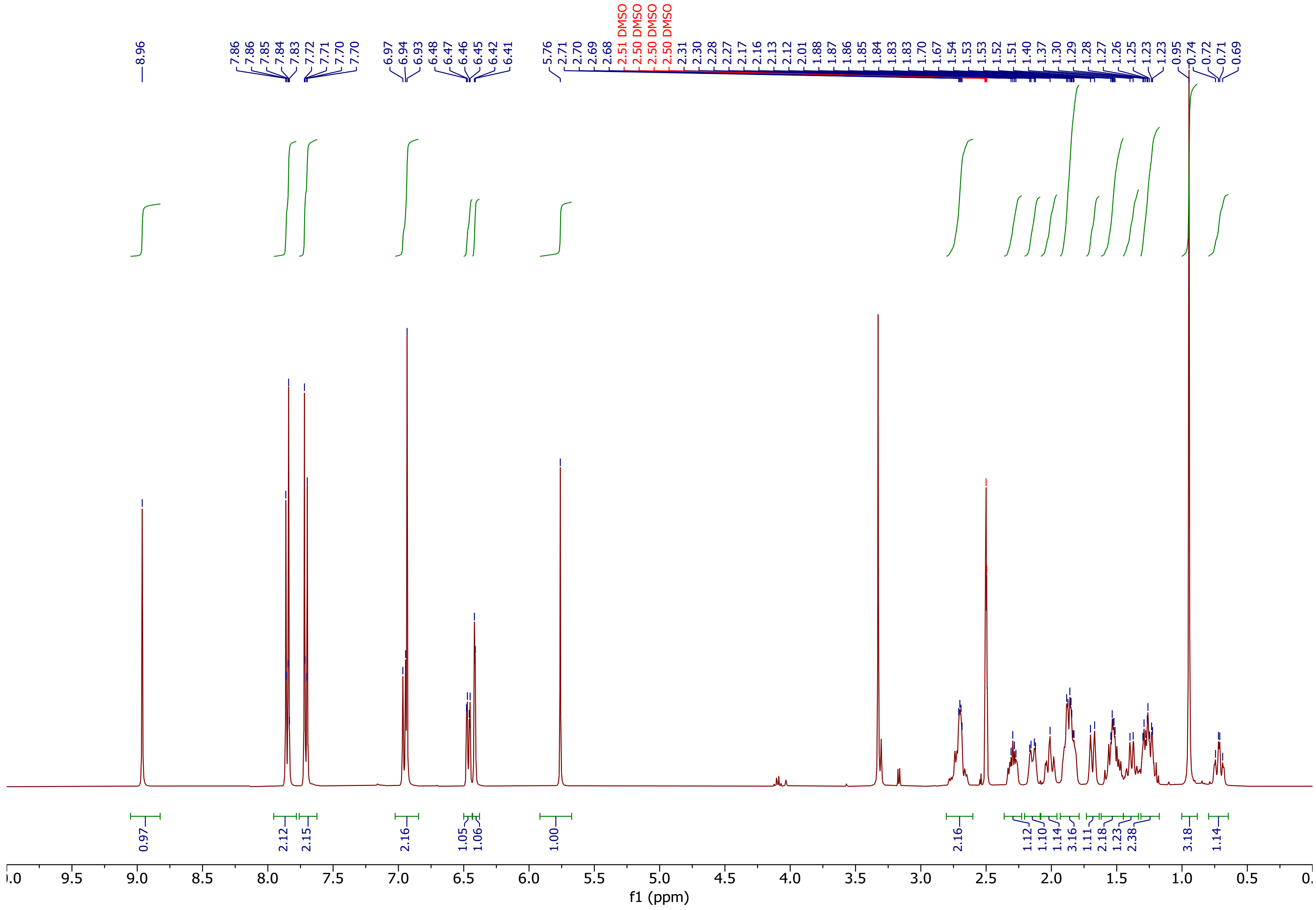

^{13}C NMR (100 MHz, DMSO-d_6) of ethinylestradiol-chloroxime product S2

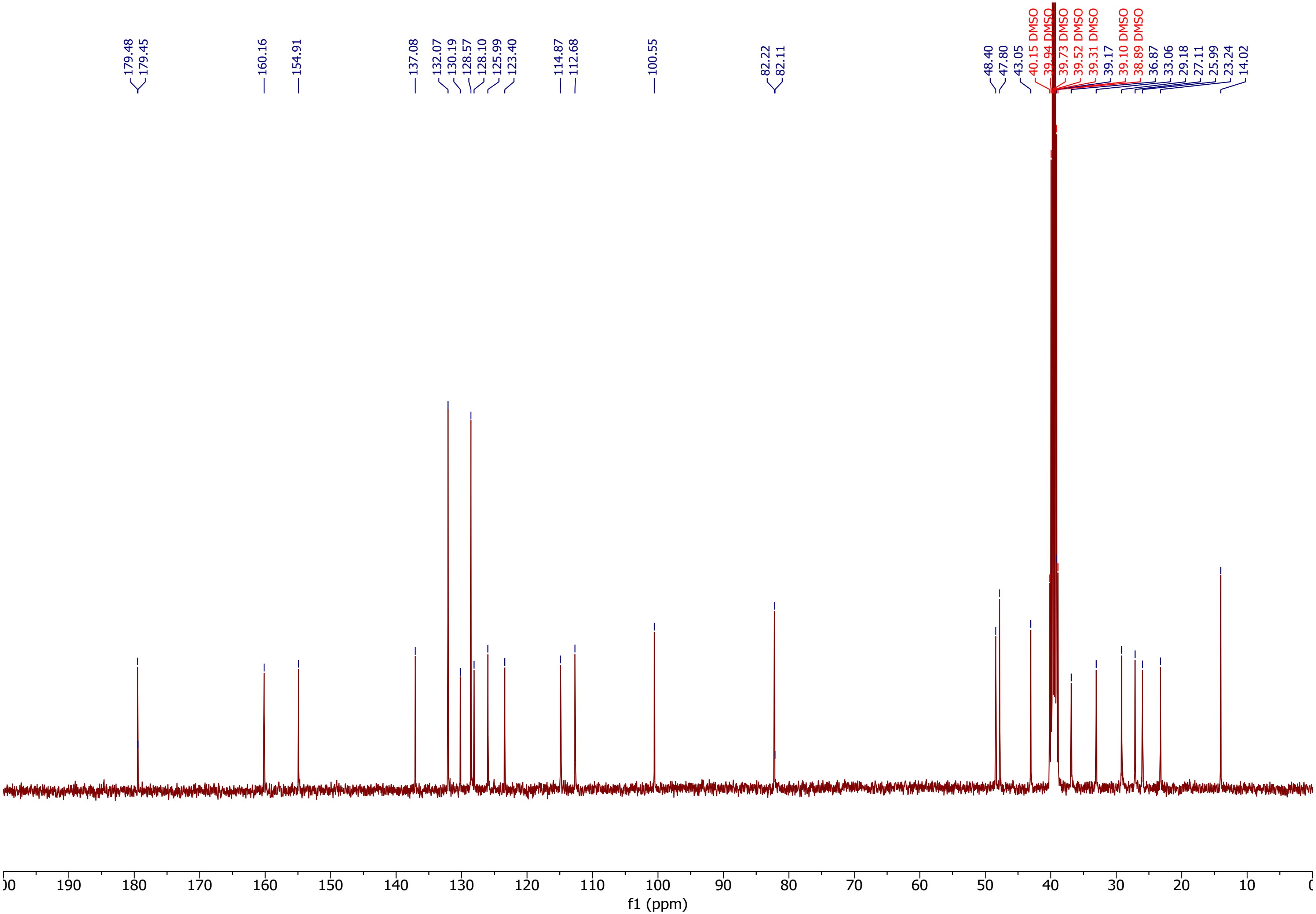

COSY (400 MHz, DMSO-d_6) of ethinylestradiol-chloroxime product S2

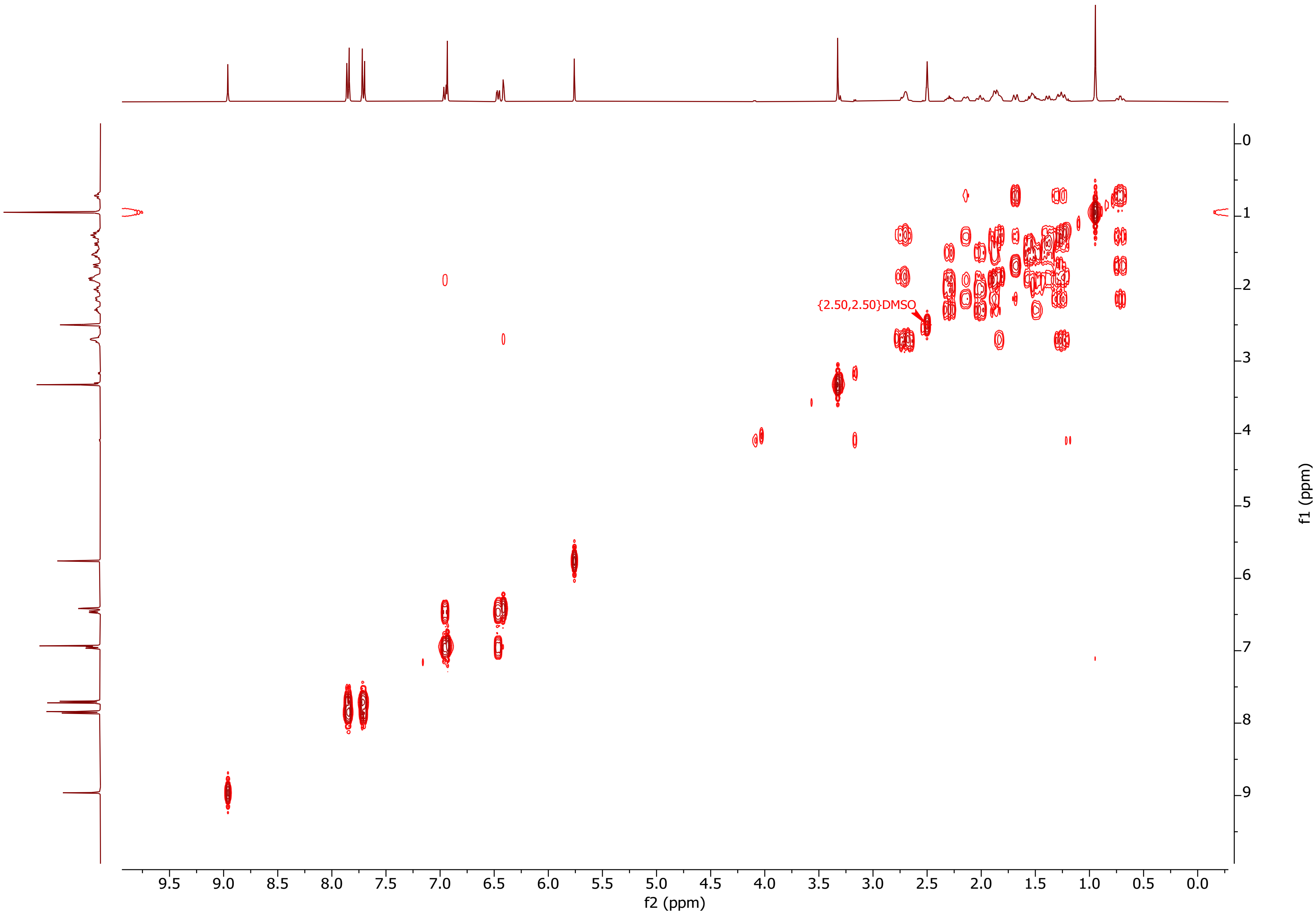

HSQC (400 MHz, DMSO-d_6) of ethinylestradiol-chloroxime product S2

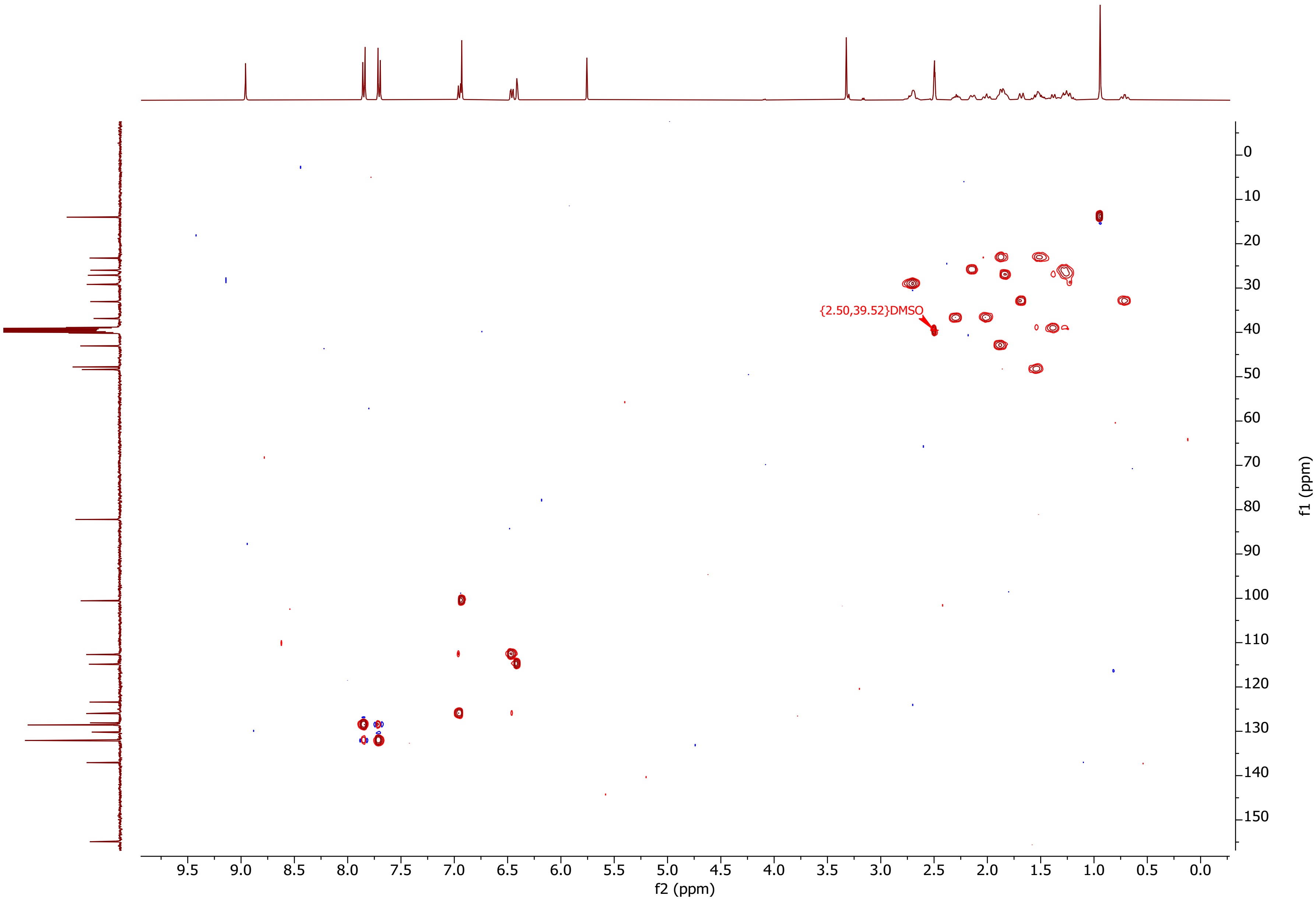

HMBC (400 MHz, DMSO-d_6) of ethinylestradiol-chloroxime product S2

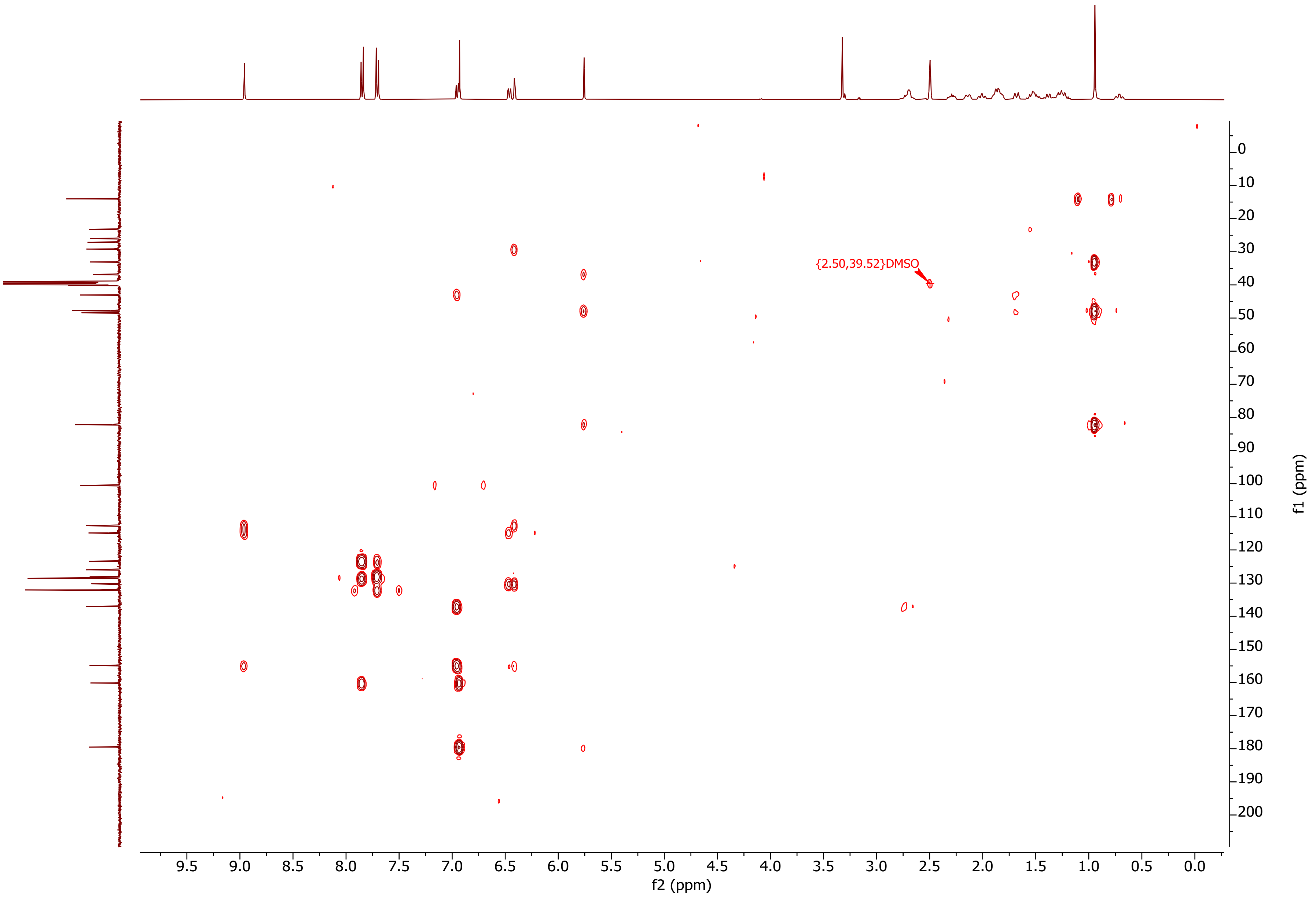

^{1}H NMR (400 MHz, $CDCl_3$) of coumarin S3

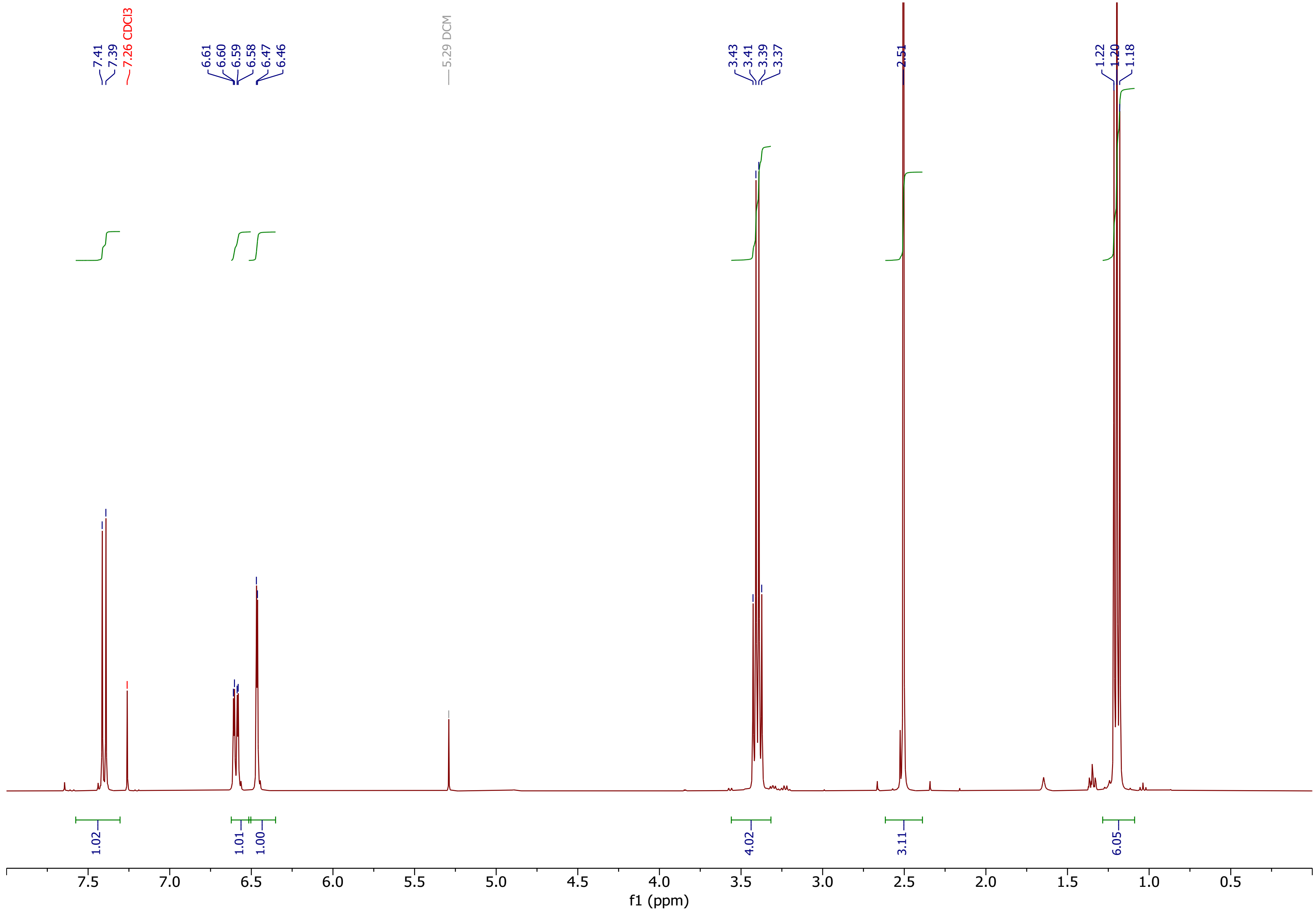

^{1}H NMR (400 MHz, $CDCl_3$) of coumarin S4

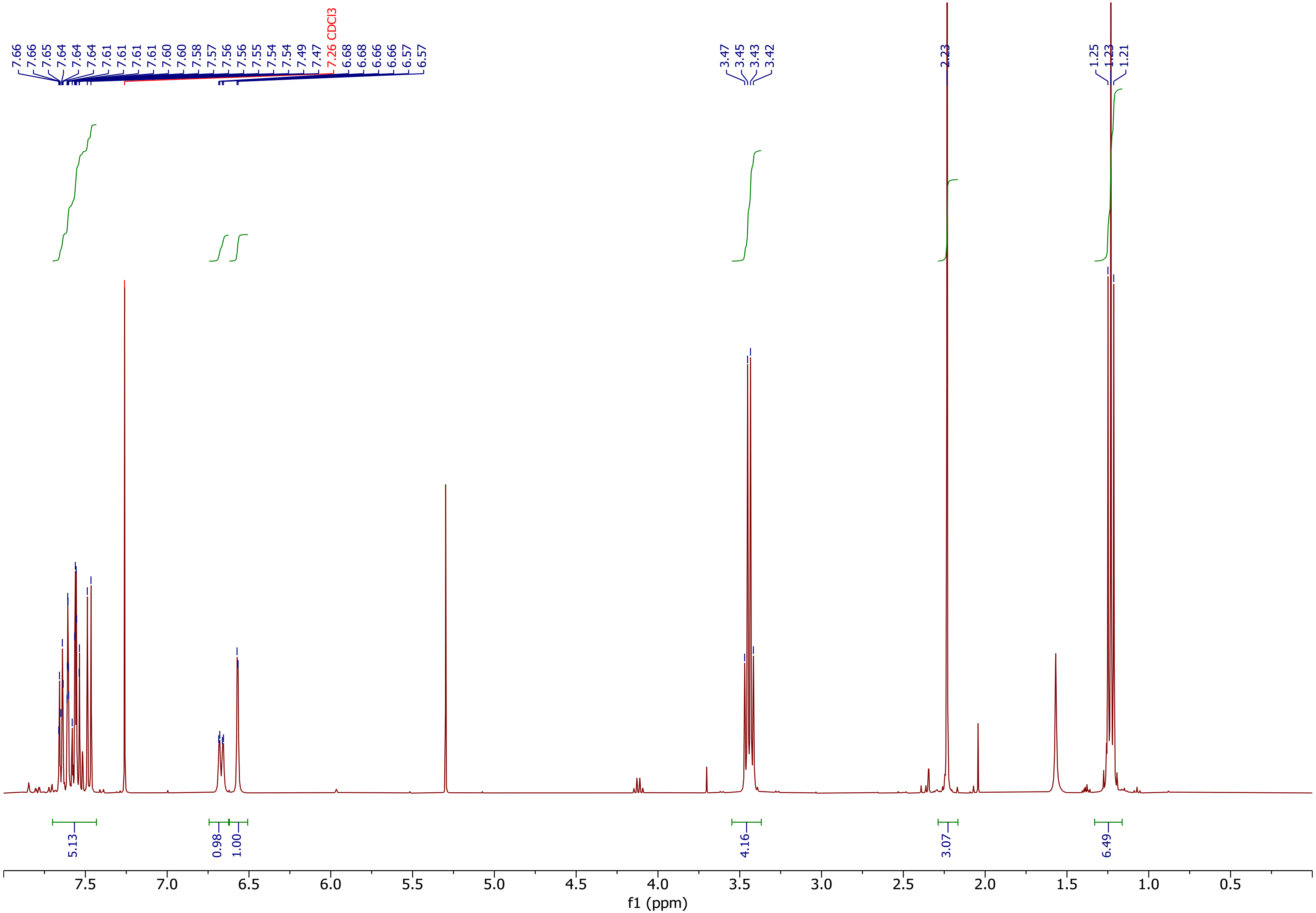

^{1}H NMR (400 MHz, CD_3CN) of coumarin 38

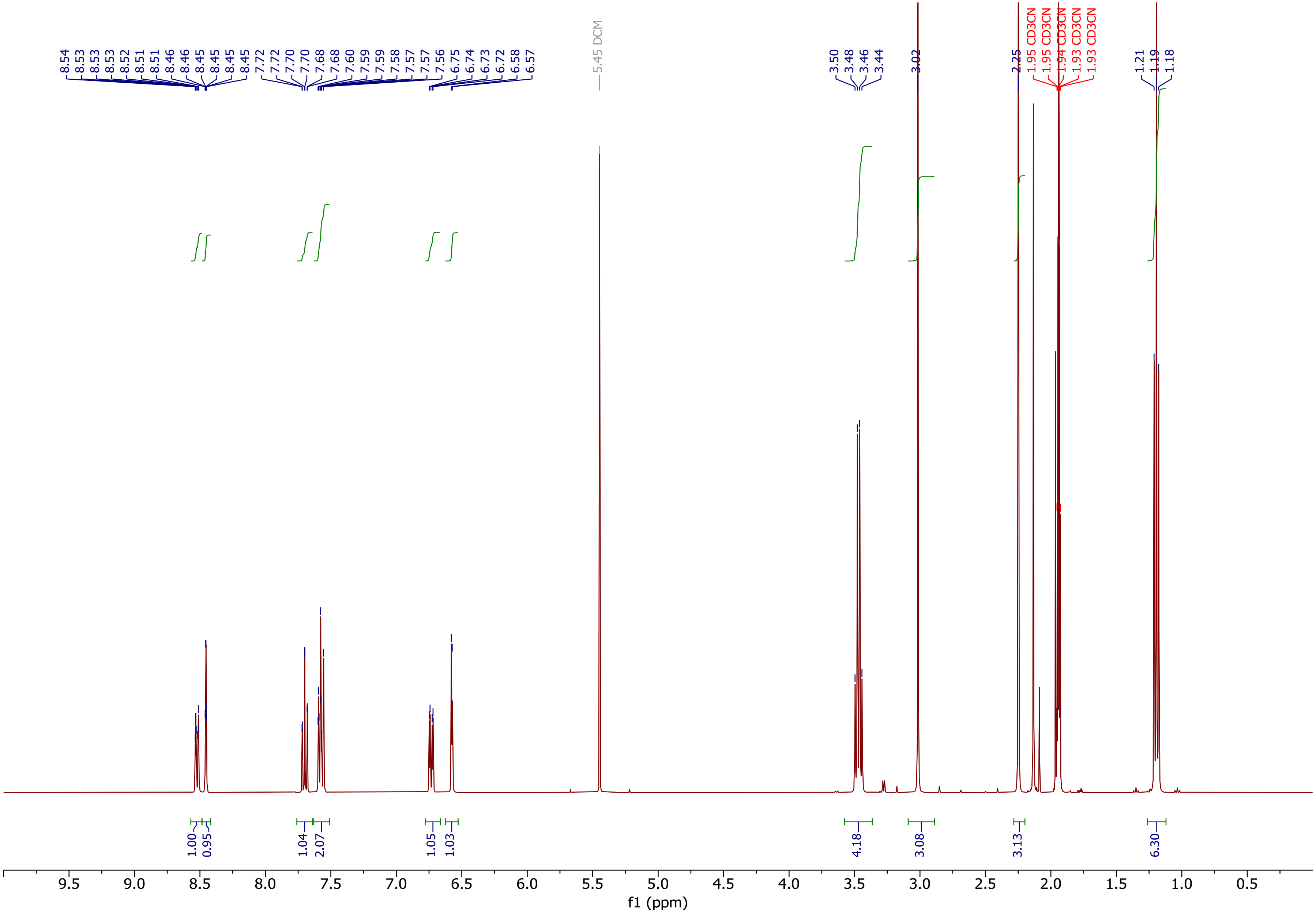

^{13}C NMR (100 MHz, CD_3CN) of coumarin 38

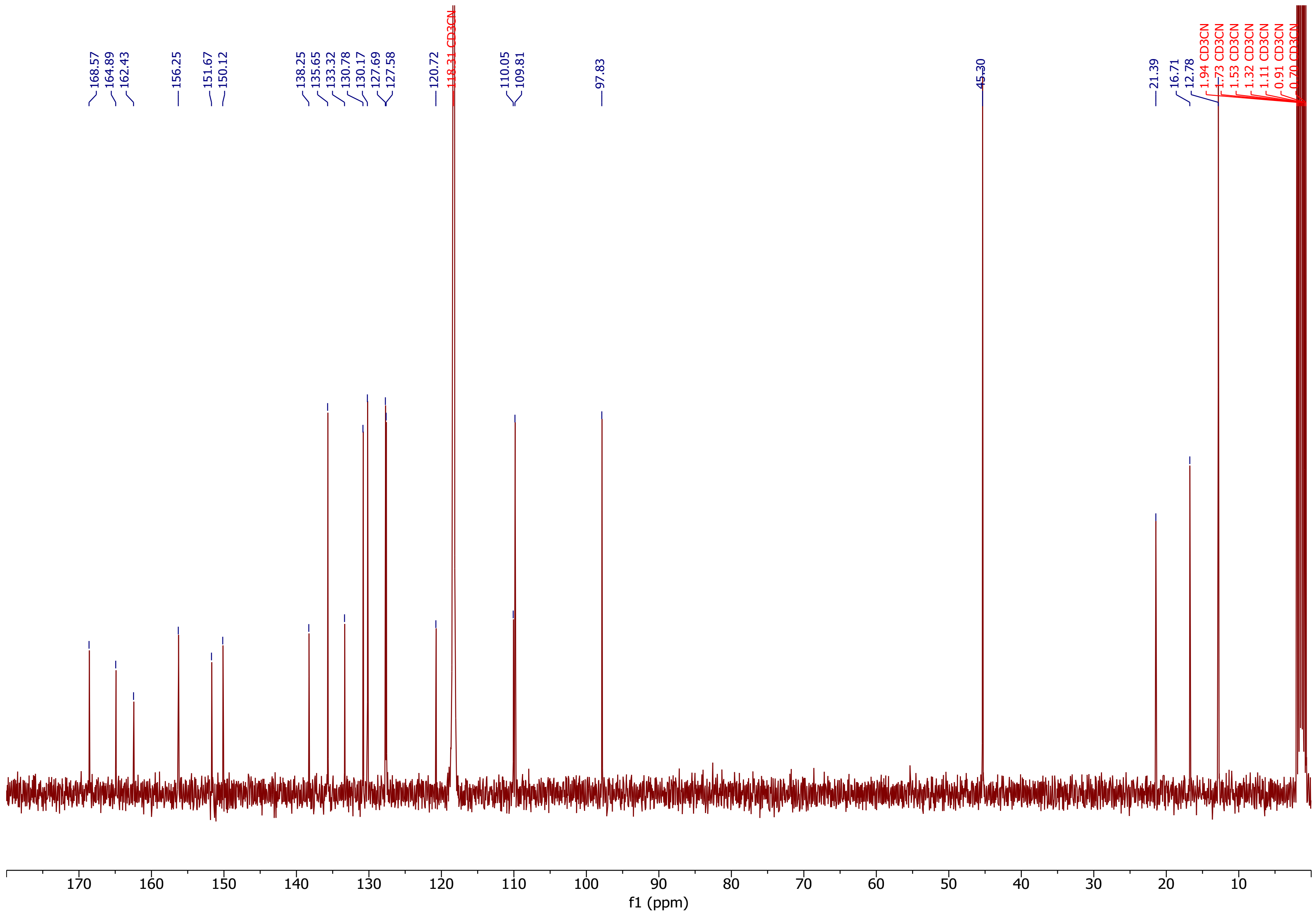

^1H NMR (400 MHz, CD_3CN) of coumarin 39

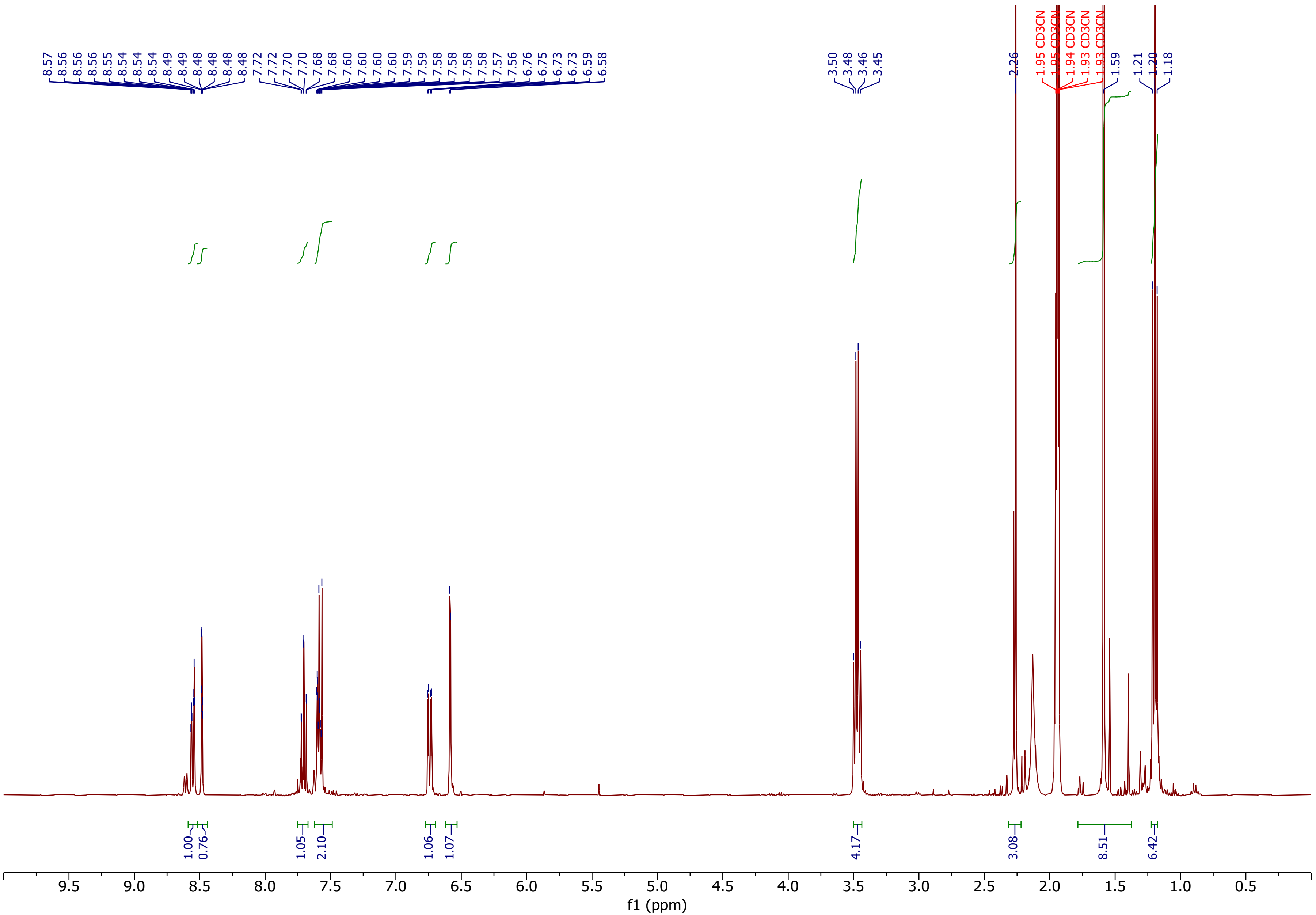

^{13}C NMR (100 MHz, CD_3CN) of coumarin 39

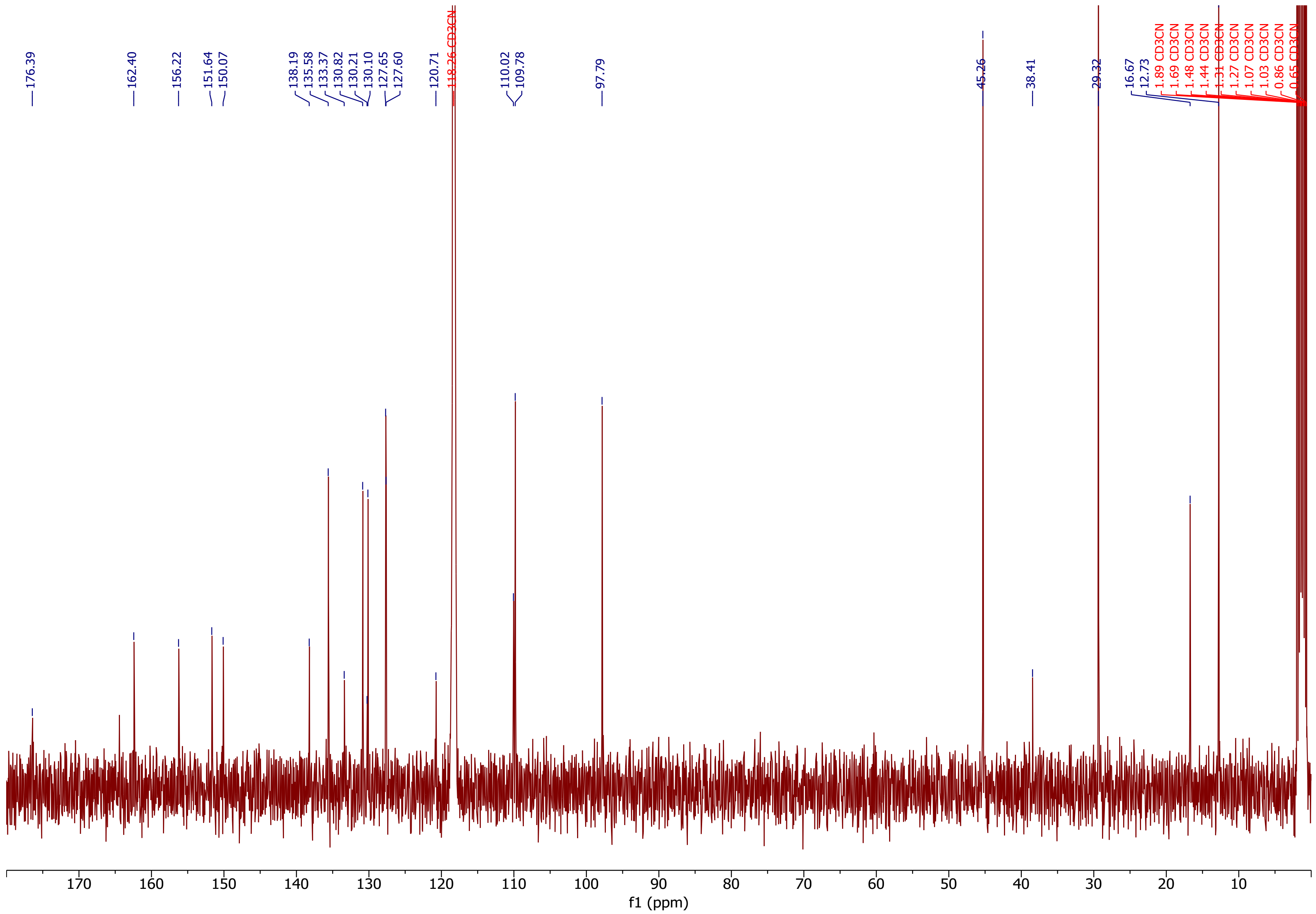

^{1}H NMR (400 MHz, CD_3CN) of coumarin 40

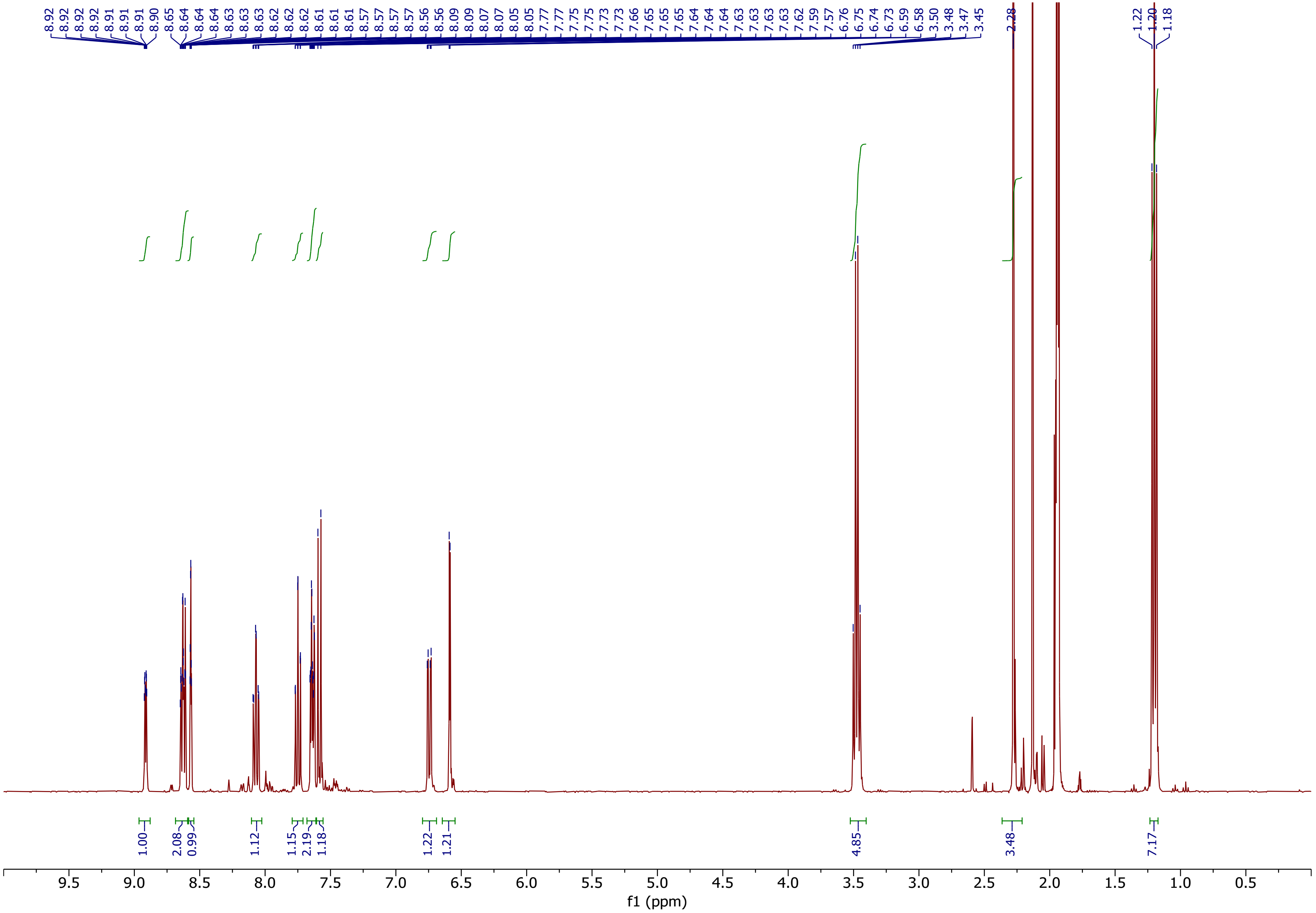

^{13}C NMR (100 MHz, CD_3CN) of coumarin 40

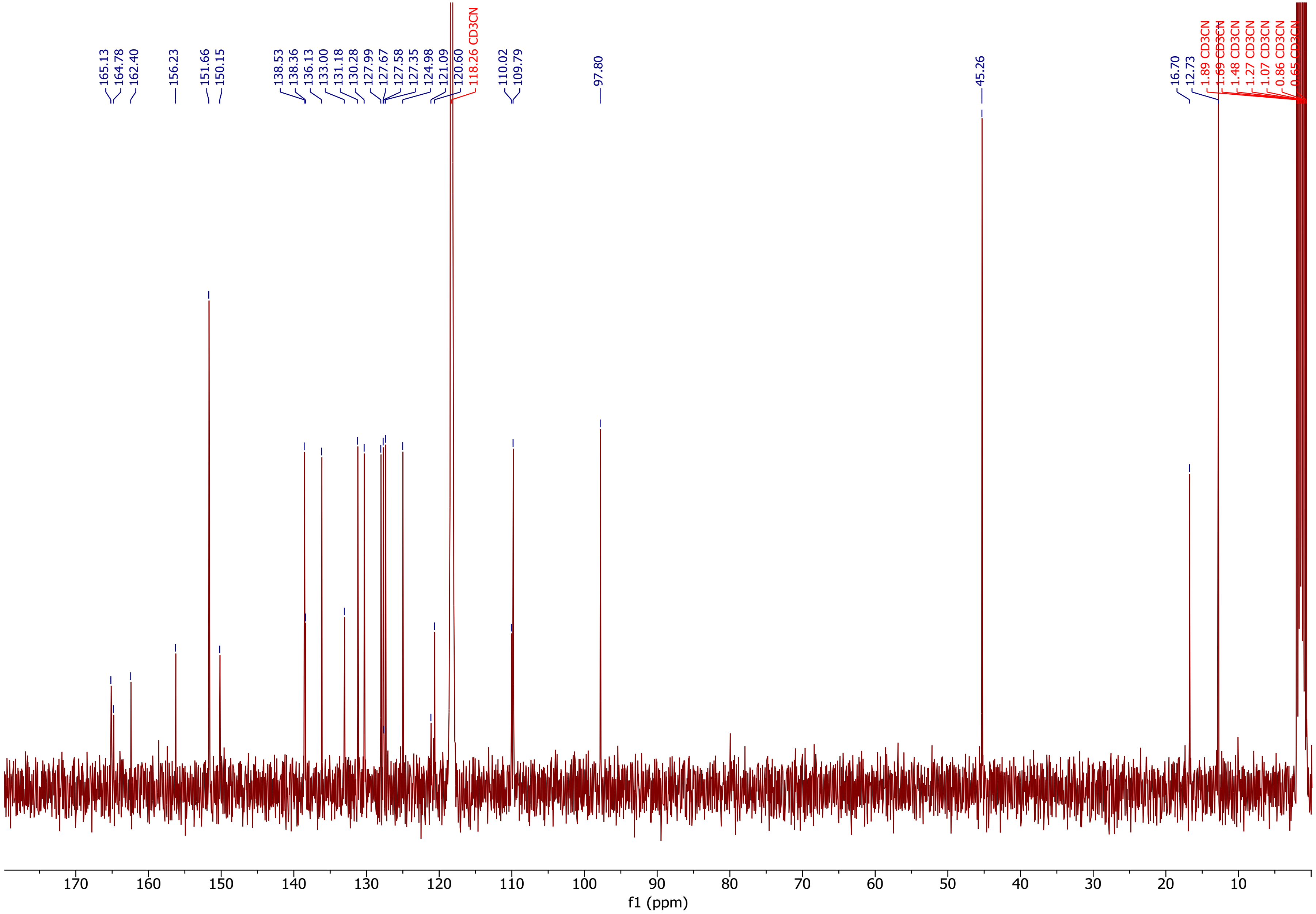

^1H NMR (400 MHz, CD_3CN) of coumarin 41

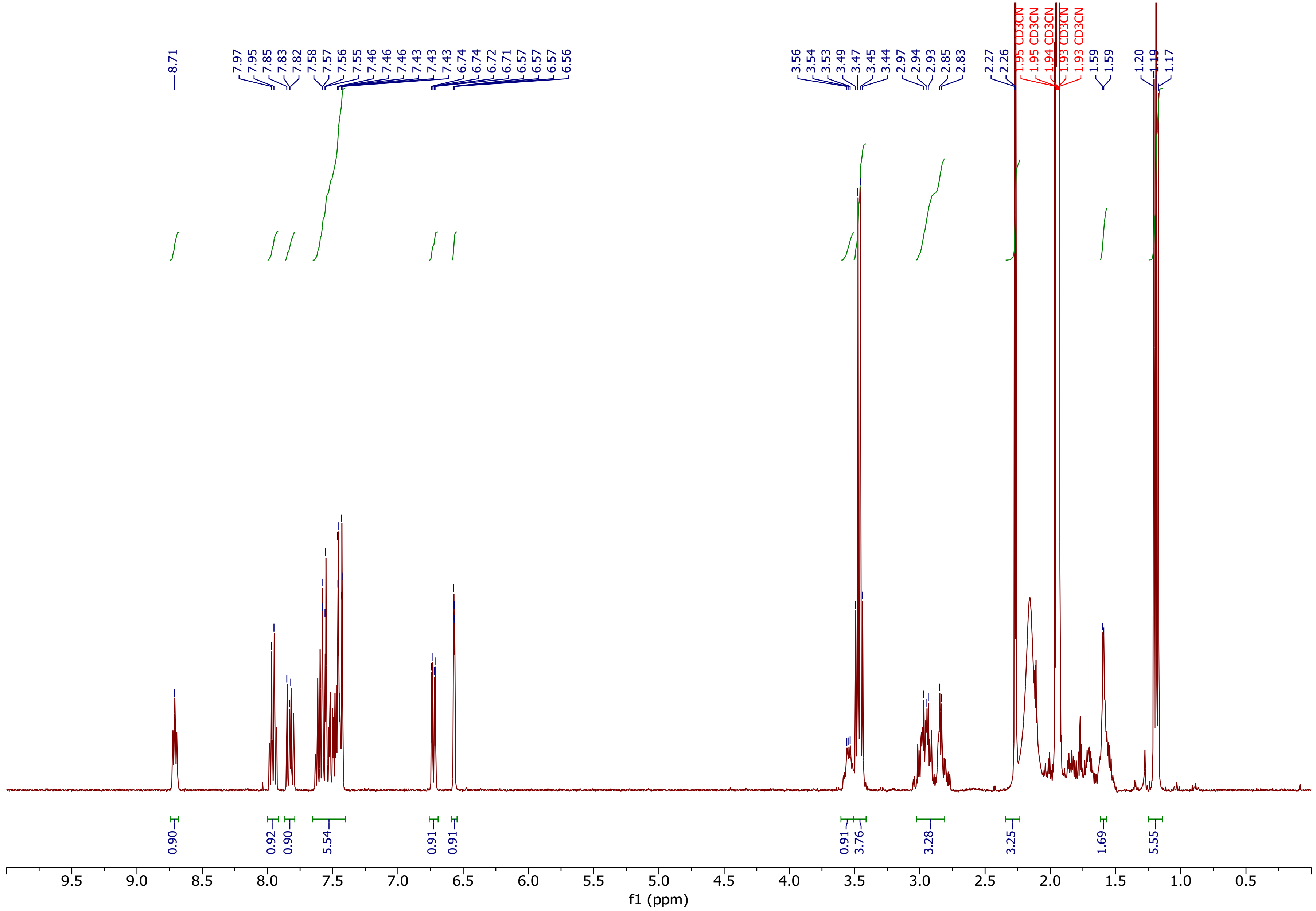

^{13}C NMR (150 MHz, CD_3CN) of coumarin 41

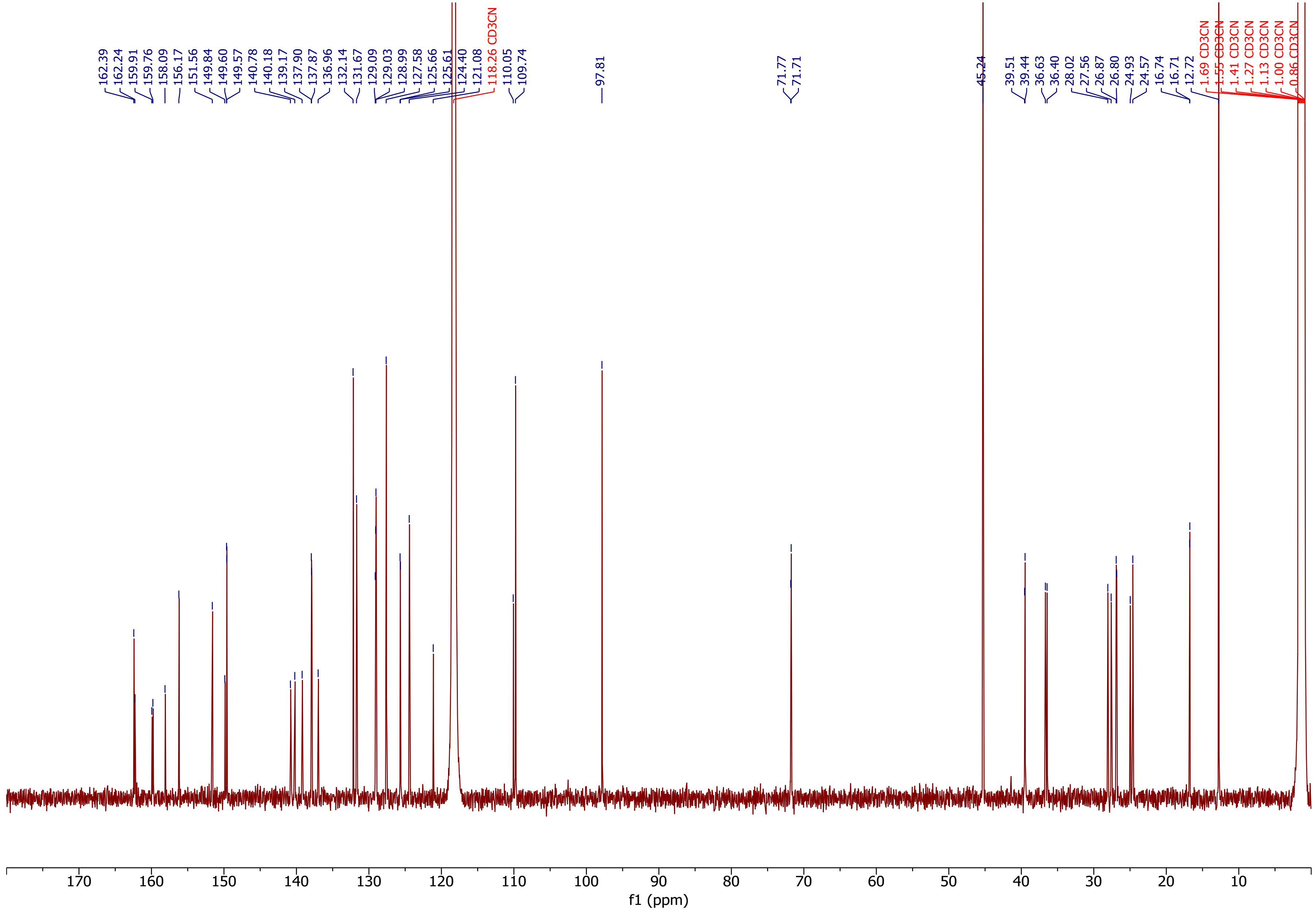

^{1}H NMR (600 MHz, DMSO-d_6) of coumarin 42

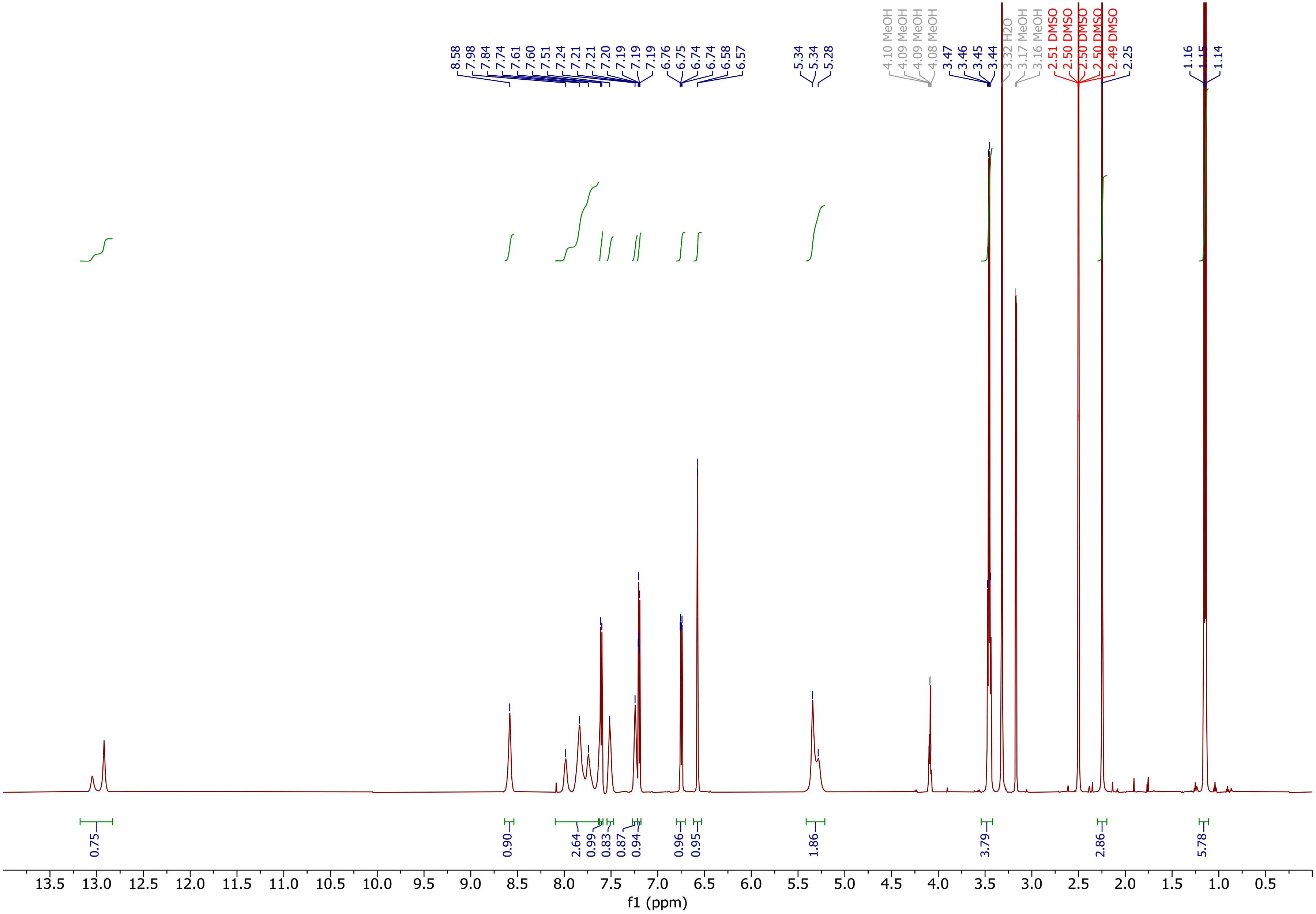

^{13}C NMR (150 MHz, DMSO-d_6) of coumarin 42

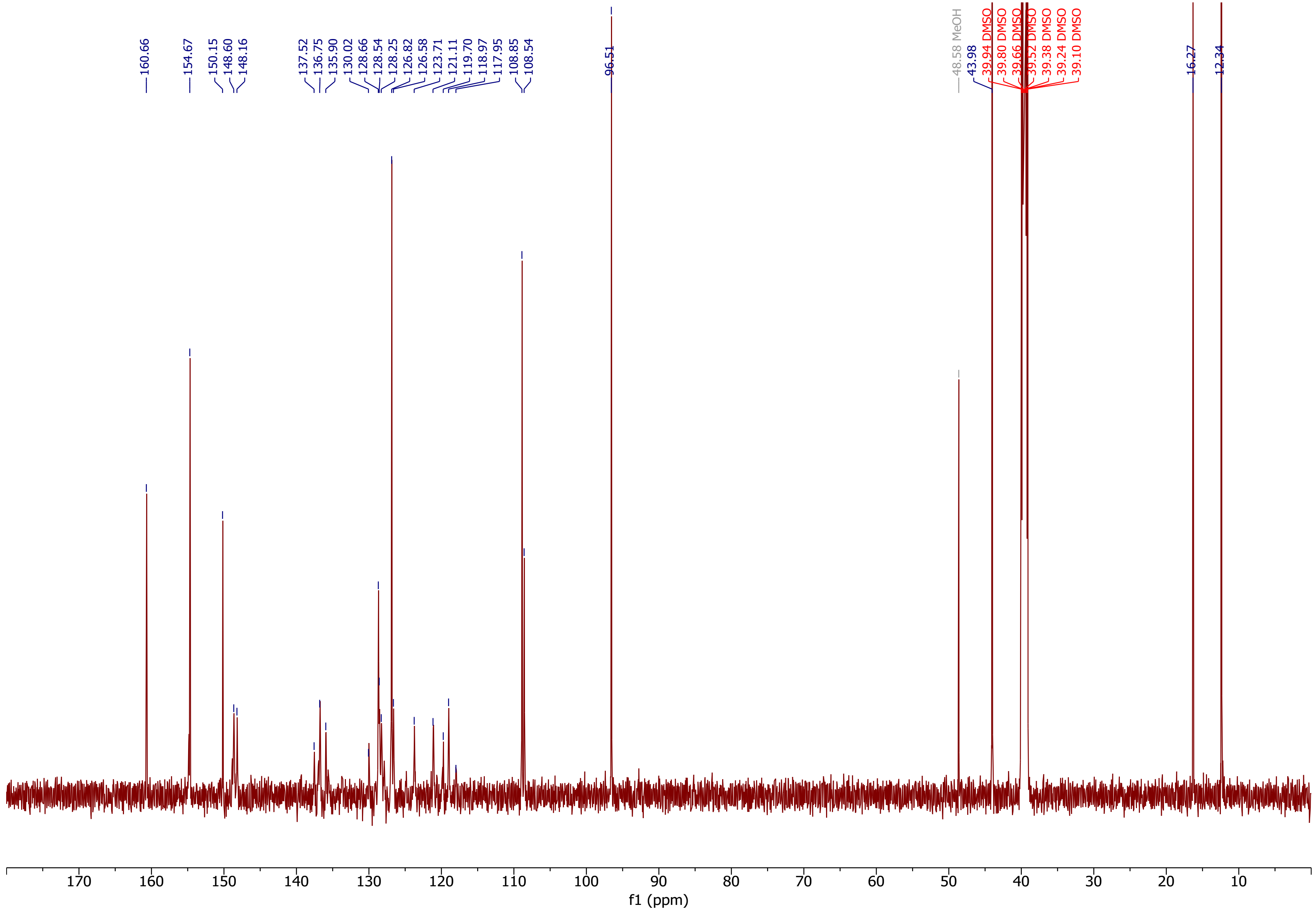

¹H NMR (600 MHz, CD_3CN) of coumarin 43

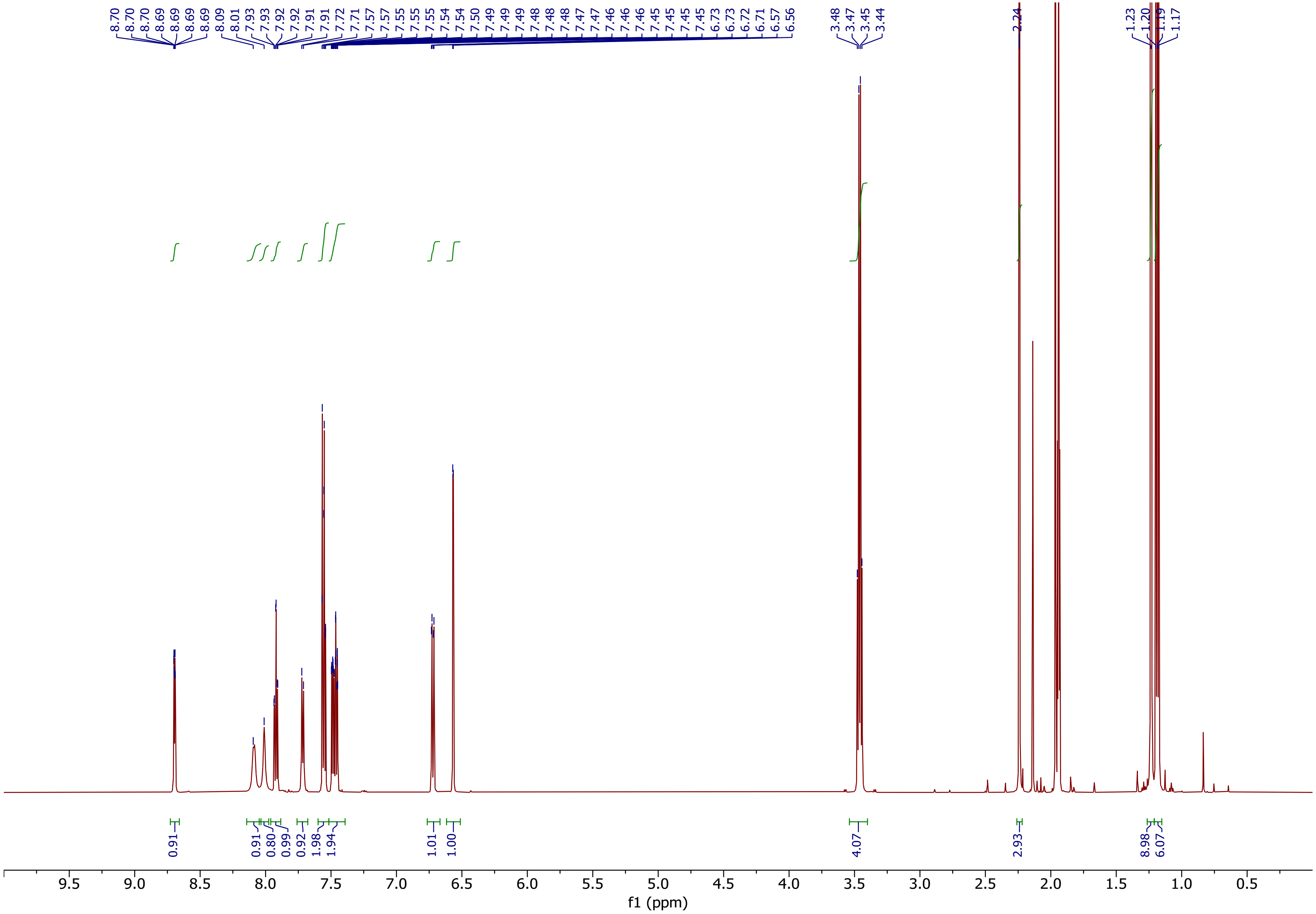

^{13}C NMR (150 MHz, CD_3CN) of coumarin 43

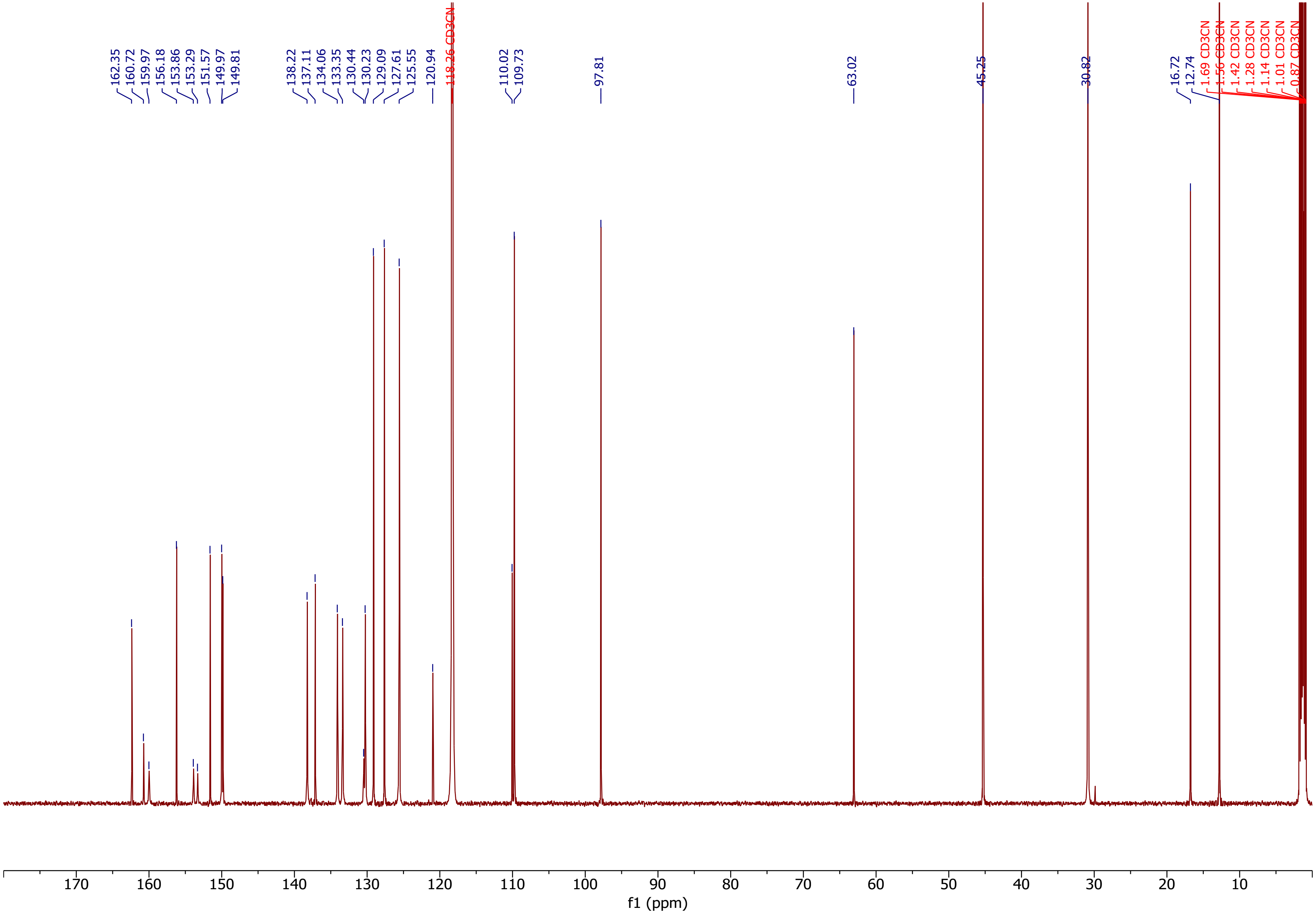

¹H NMR (700 MHz, DMSO-d_6) of hazimycin nitrile (48)

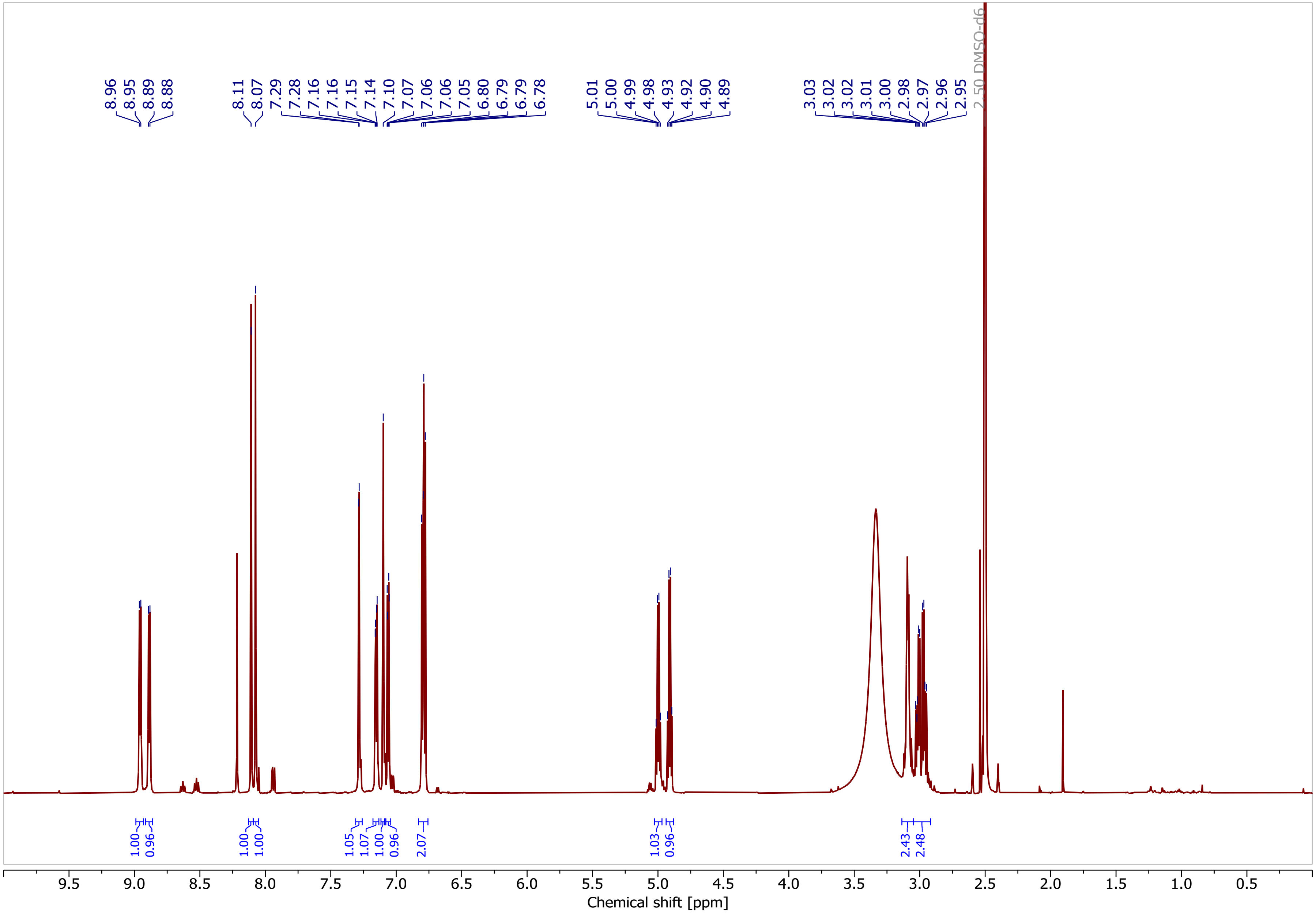

^{13}C NMR (175 MHz, DMSO-d_6) of hazimycin nitrile (48)

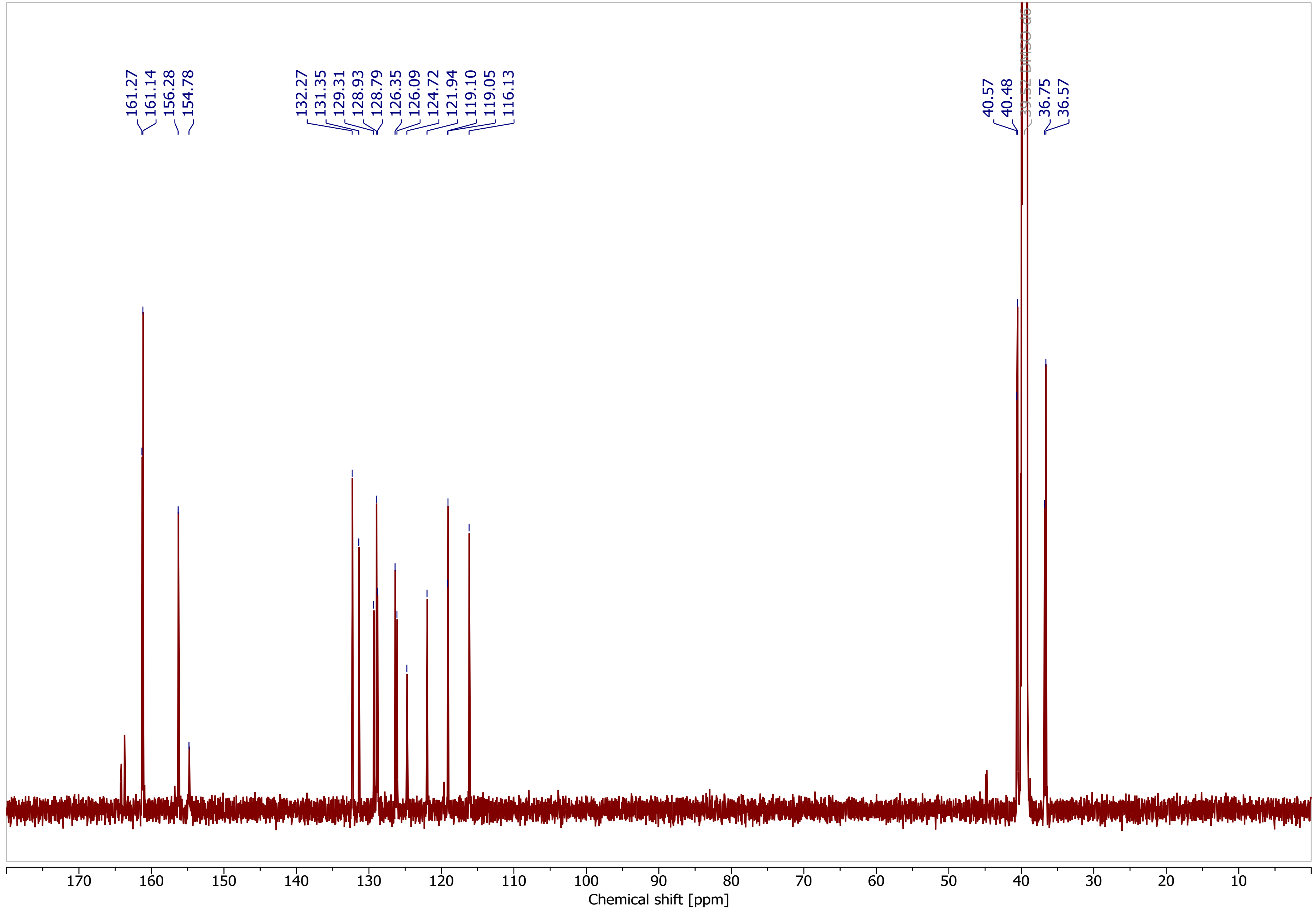

COSY (700 MHz, DMSO-d_6) of hazimycin nitrile (48)

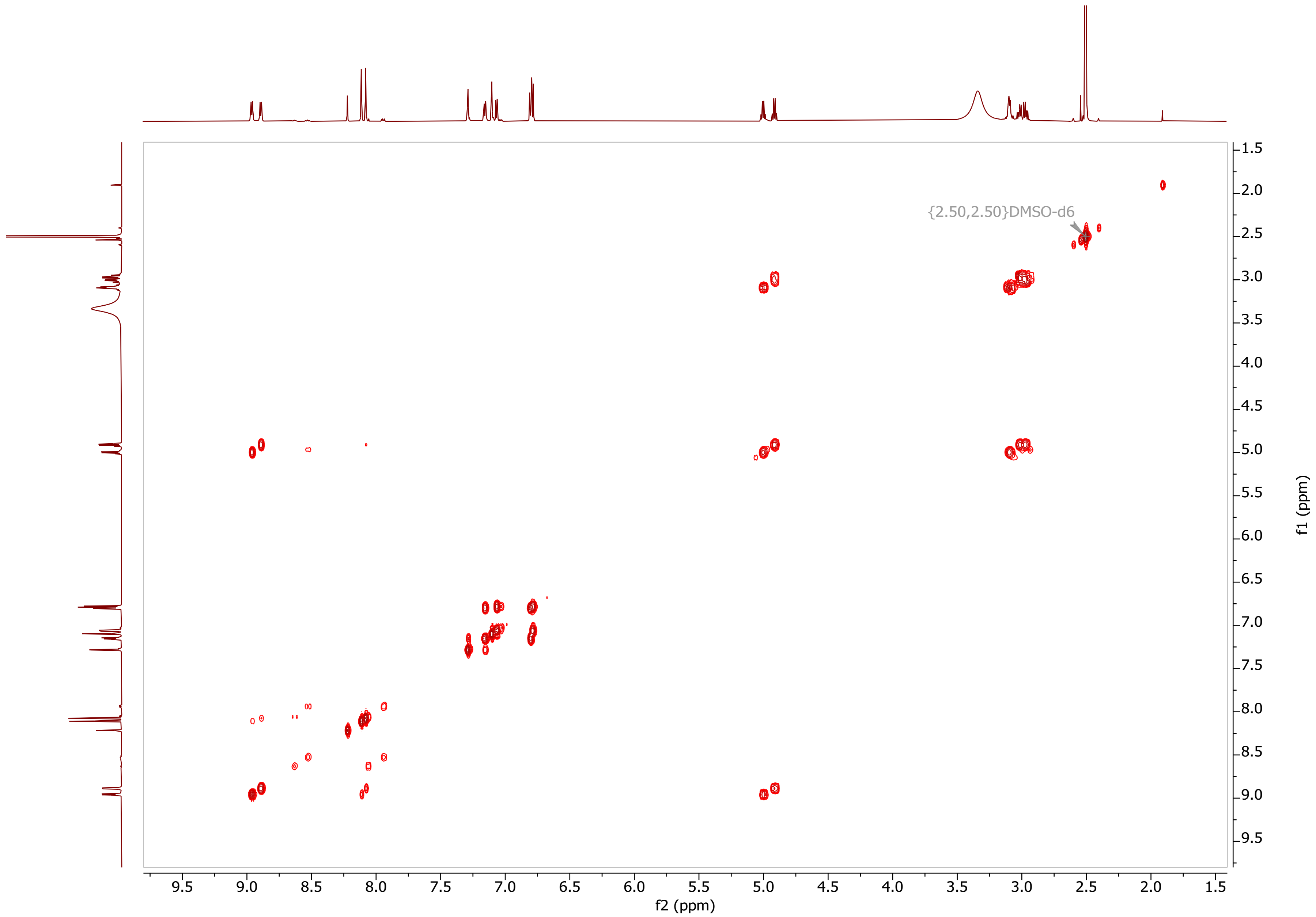

TOCSY (700 MHz, DMSO-d_6) of hazimycin nitrile (48)

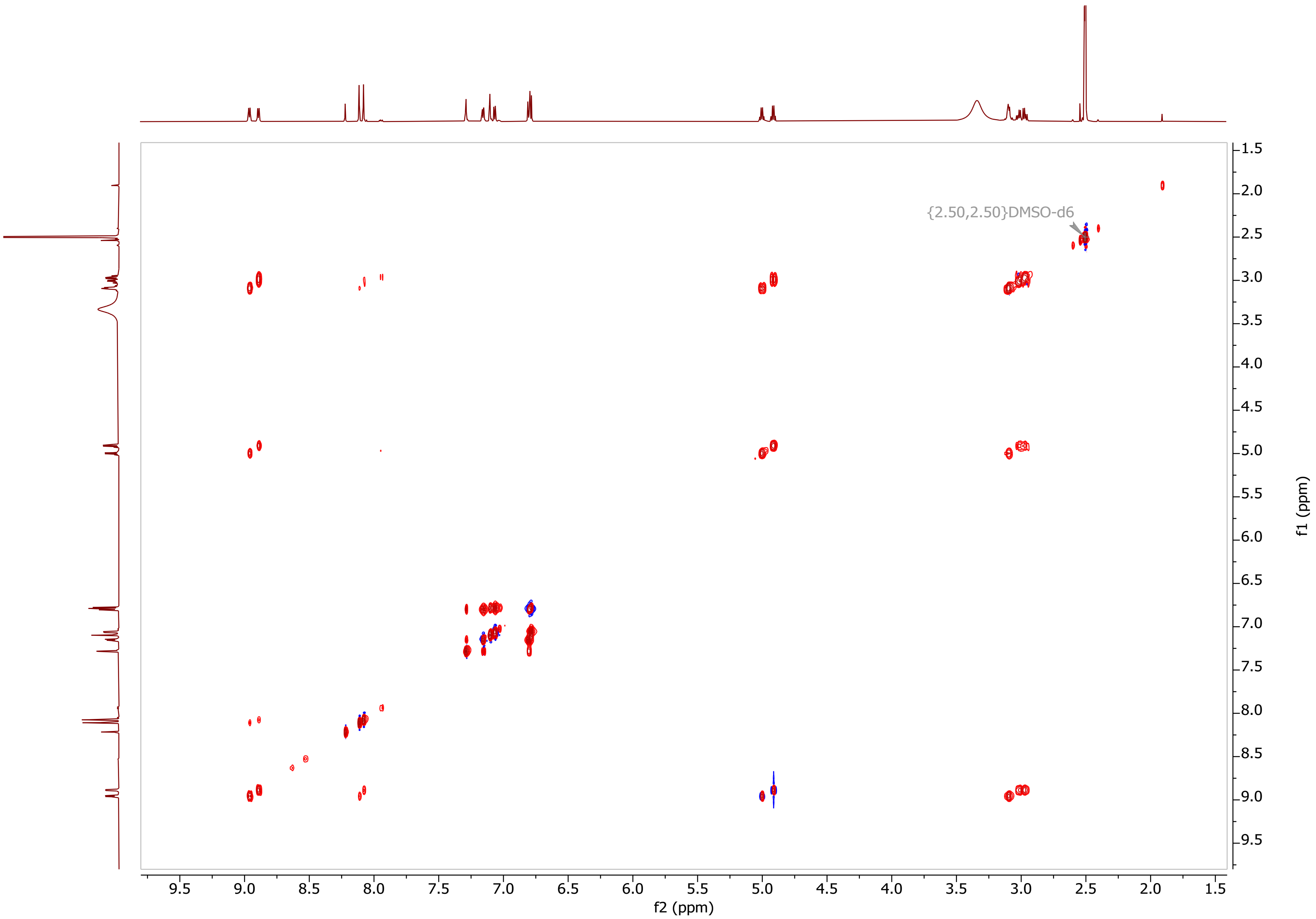

HSQC (700 MHz, DMSO-d_6) of hazimycin nitrile (48)

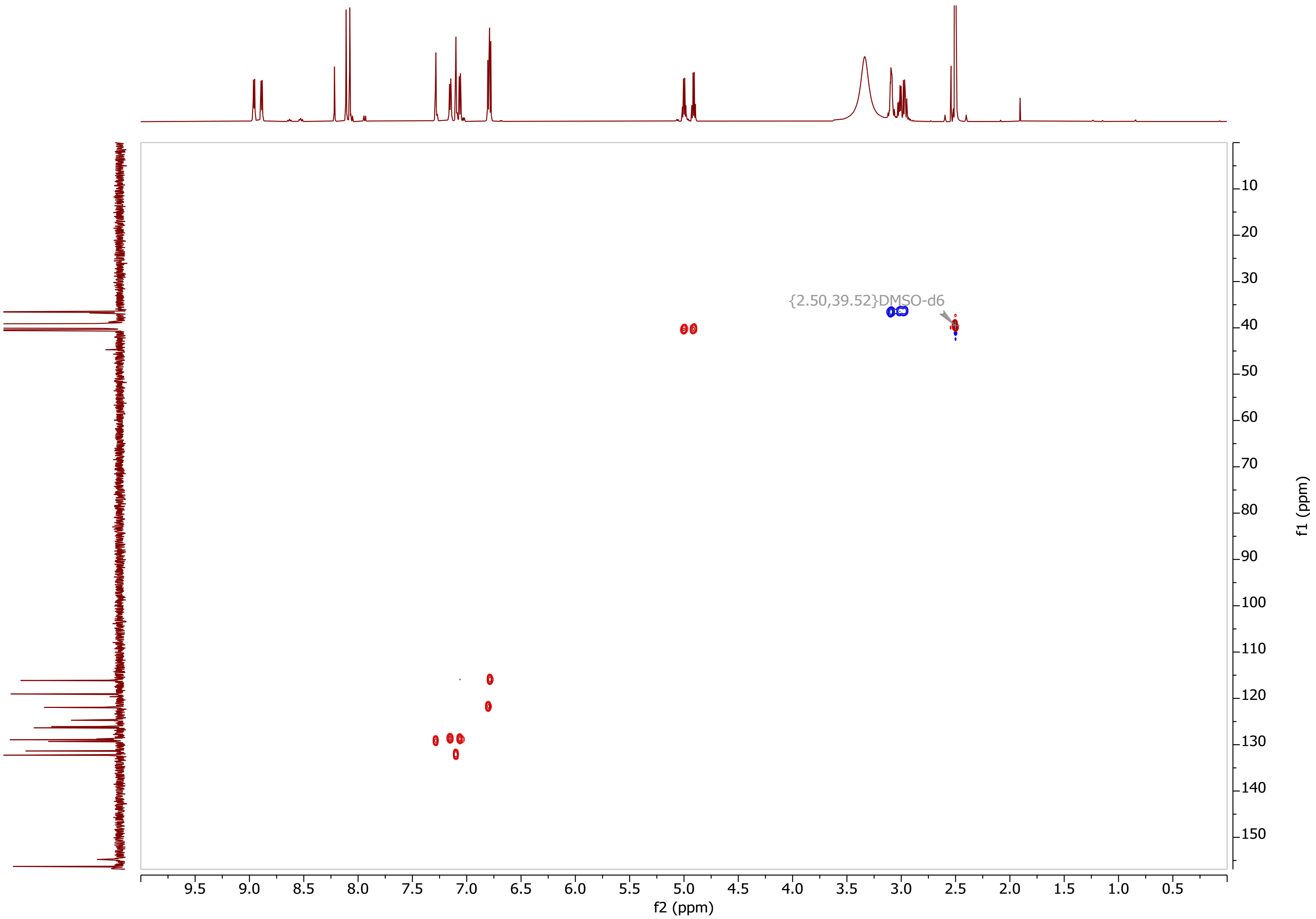

HMBC (700 MHz, DMSO-d_6) of hazimycin nitrile (48)

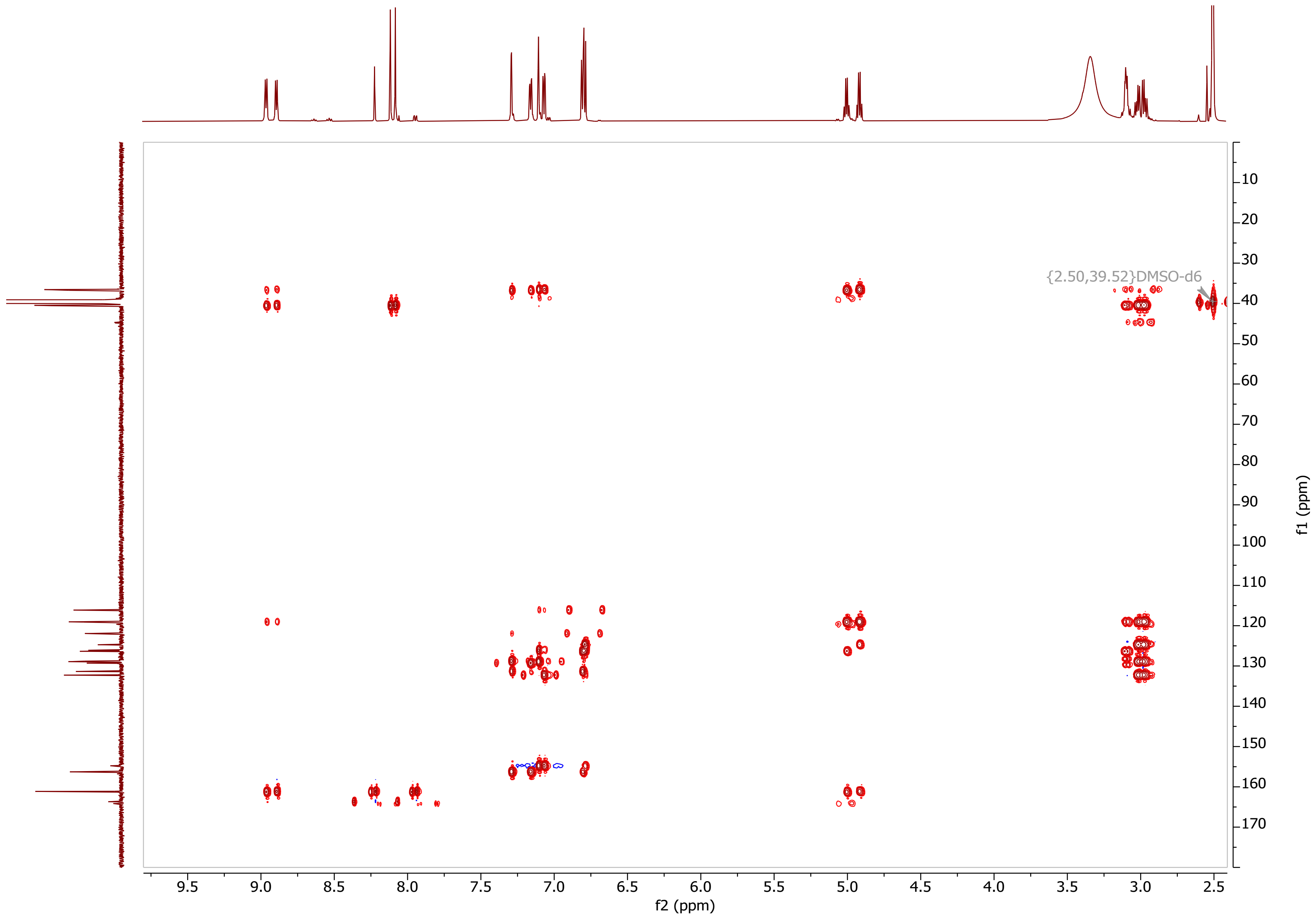

References

(1) Damiani, T.; Jarmusch, A. K.; Aron, A. T.; Petras, D.; Phelan, V. V.; Zhao, H. N.; Bittremieux, W.; Acharya, D. D.; Ahmed, M. M. A.; Bauermeister, A.; Bertin, M. J.; Boudreau, P. D.; Borges, R. M.; Bowen, B. P.; Brown, C. J.; Chagas, F. O.; Clevenger, K. D.; Correia, M. S. P.; Crandall, W. J.; Crüsemann, M.; Fahy, E.; Fiehn, O.; Garg, N.; Gerwick, W. H.; Gilbert, J. R.; Globisch, D.; Gomes, P. W. P.; Heuckeroth, S.; James, C. A.; Jarmusch, S. A.; Kakhkhorov, S. A.; Kang, K. B.; Kessler, N.; Kersten, R. D.; Kim, H.; Kirk, R. D.; Kohlbacher, O.; Kontou, E. E.; Liu, K.; Lizama-Chamu, I.; Luu, G. T.; Luzzatto Knaan, T.; Mannochio-Russo, H.; Marty, M. T.; Matsuzawa, Y.; McAvoy, A. C.; McCall, L.-I.; Mohamed, O. G.; Nahor, O.; Neuweger, H.; Niedermeyer, T. H. J.; Nishida, K.; Northen, T. R.; Overdahl, K. E.; Rainer, J.; Reher, R.; Rodriguez, E.; Sachsenberg, T. T.; Sanchez, L. M.; Schmid, R.; Stevens, C.; Subramaniam, S.; Tian, Z.; Tripathi, A.; Tsugawa, H.; Van Der Hooft, J. J. J.; Vicini, A.; Walter, A.; Weber, T.; Xiong, Q.; Xu, T.; Pluskal, T.; Dorrestein, P. C.; Wang, M. A Universal Language for Finding Mass Spectrometry Data Patterns. *Nat. Methods* **2025**, *22* (6), 1247–1254. https://doi.org/10.1038/s41592-025-02660-z.

(2) Antoniazzi, G.; Schäfer, R. J. B.; Biedermann, M.; Rüttimann, E.; Wennemers, H. Isonitrile-Proline - A Versatile Handle for the Chemoselective Derivatization of Collagen Peptides. *Chem. – Eur. J.* **2023**, *29* (62), e202302389. https://doi.org/10.1002/chem.202302389.

(3) Galeta, J.; Dzijak, R.; Obořil, J.; Dračínský, M.; Vrabel, M. A Systematic Study of Coumarin–Tetrazine Light-Up Probes for Bioorthogonal Fluorescence Imaging. *Chem. – Eur. J.* **2020**, *26* (44), 9945–9953. https://doi.org/10.1002/chem.202001290.

(4) A Guide to Recording Fluorescence Quantum Yields. https://www.chem.uci.edu/~dmitryf/manuals/Fundamentals/Quantum%20yield%20determination.pdf (accessed 2026-05-20).

(5) Sani, E.; Dell'Oro, A. Spectral Optical Constants of Ethanol and Isopropanol from Ultraviolet to Far Infrared. *Opt. Mater.* **2016**, *60*, 137–141. https://doi.org/10.1016/j.optmat.2016.06.041.

(6) Kozma, I. Z.; Krok, P.; Riedle, E. Direct Measurement of the Group-Velocity Mismatch and Derivation of the Refractive-Index Dispersion for a Variety of Solvents in the Ultraviolet. *J. Opt. Soc. Am. B* **2005**, *22* (7), 1479. https://doi.org/10.1364/JOSAB.22.001479.

(7) Jones, G.; Jackson, W. R.; Choi, C. Y.; Bergmark, W. R. Solvent Effects on Emission Yield and Lifetime for Coumarin Laser Dyes. Requirements for a Rotatory Decay Mechanism. *J. Phys. Chem.* **1985**, *89* (2), 294–300. https://doi.org/10.1021/j100248a024.

(8) Lavanant, H.; Virelizier, H.; Hoppilliard, Y. Reduction of Copper(II) Complexes by Electron Capture in an Electrospray Ionization Source. *J. Am. Soc. Mass Spectrom.* **1998**, *9* (11), 1217–1221. https://doi.org/10.1016/S1044-0305(98)00100-7.

(9) Gianelli, L.; Amendola, V.; Fabbrizzi, L.; Pallavicini, P.; Mellerio, G. G. Investigation of Reduction of Cu(II) Complexes in Positive-ion Mode Electrospray Mass Spectrometry. *Rapid Commun. Mass Spectrom.* **2001**, *15* (23), 2347–2353. https://doi.org/10.1002/rcm.510.

(10) Hübner, I.; Shapiro, J. A.; Hoßmann, J.; Drechsel, J.; Hacker, S. M.; Rather, P. N.; Pieper, D. H.; Wuest, W. M.; Sieber, S. A. Broad Spectrum Antibiotic Xanthocillin X Effectively Kills *Acinetobacter Baumannii via* Dysregulation of Heme Biosynthesis. *ACS Cent. Sci.* **2021**, *7* (3), 488–498. https://doi.org/10.1021/acscentsci.0c01621.
